# A review of advances in tribology in 2020–2021

**DOI:** 10.1007/s40544-022-0685-7

**Published:** 2022-10-11

**Authors:** Yonggang Meng, Jun Xu, Liran Ma, Zhongmin Jin, Braham Prakash, Tianbao Ma, Wenzhong Wang

**Affiliations:** 1 State Key Laboratory of Tribology in Advanced Equipment, Tsinghua University, Beijing, 100084 China; 2 School of Mechanical Engineering, Southwest Jiaotong University, Chengdu, 610031 China; 3 School of Mechanical Engineering, University of Leeds, Leeds, LS2 9JT UK; 4 School of Mechanical and Vehicle Engineering, Beijing Institute of Technology, Beijing, 100082 China

**Keywords:** lubrication, friction, wear, surface engineering, tribology, biotribology, high temperature, computer simulations

## Abstract

Around 1,000 peer-reviewed papers were selected from 3,450 articles published during 2020–2021, and reviewed as the representative advances in tribology research worldwide. The survey highlights the development in lubrication, wear and surface engineering, biotribology, high temperature tribology, and computational tribology, providing a show window of the achievements of recent fundamental and application researches in the field of tribology.

## Contents

1 Introduction ....................(P1445)
2 Lubrication ....................(P1446)
  2.1 Introduction of lubrication ....................(P1446)
  2.2 Approaches for revealing the lubrication mechanism ....................(P1446)
  2.3 Liquid lubrication ....................(P1446)
    2.3.1 Ionic liquid lubrication ....................(P1447)
    2.3.2 Water-based lubrication ....................(P1447)
      2.3.2.1 Additives in water-based lubrication ....................(P1448)
      2.3.2.2 Influence factors of water-based lubrication ....................(P1449)
    2.3.3 Emulsion lubrication ....................(P1450)
    2.3.4 Grease lubrication ....................(P1450)
    2.3.5 Lubricant Additives ....................(P1452)
  2.4 Solid lubrication ....................(P1454)
    2.4.1 Carbon-based materials ....................(P1454)
      2.4.1.1 Graphite and derivatives ....................(P1454)
      2.4.1.2 Diamond like carbon (DLC) films ....................(P1456)
      2.4.1.3 Other carbon-based materials ....................(P1459)
    2.4.2 Transition metal dichalcogenides (TMDs) ....................(P1459)
    2.4.3 Polymers and material modification ....................(P1460)
  2.5 Other lubrication ....................(P1460)
    2.5.1 Liquid metal ....................(P1461)
    2.5.2 Gas lubrication ....................(P1461)
    2.5.3 Surface texture ....................(P1461)
    2.5.4 Self-lubricating materials ....................(P1461)
    2.5.5 Tribofilm ....................(P1464)
    2.5.6 High/low temperature lubrication ....................(P1465)
  2.6 Application of lubrication ....................(P1466)
    2.6.1 Biotribology ....................(P1466)
    2.6.2 Lubrication of machinery manufacturing ....................(P1467)
    2.6.3 Lubrication in space or marine ....................(P1468)
3 Wear and surface engineering ....................(P1468)
  3.1 Introduction of wear and surface engineering ....................(P1468)
  3.2 Wear mechanisms and relationships with material structures and working conditions ....................(P1469)
    3.2.1 Catastrophic adhesive wear mechanisms ....................(P1469)

3.2.2 Rolling contact fatigue (RCF) and white etching crack (WEC) ......(P1470)
3.2.3 Wheel–rail wear modes............(P1471)
3.2.4 Cavitation erosion and corrosive wear ......................................................(P1472)
3.2.5 Fretting wear and subsurface damages ......................................................(P1474)
3.2.6 Wear of polymer, rubber, and hydrogels contacting with similar or harder surfaces .......................................(P1475)
3.2.7 Material transfer and wear debris dynamics in wear process ........(P1477)
3.2.8 Abrasive wear ............................(P1479)
3.2.9 Oxidative wear and tribochemical films ......................................................(P1480)
3.2.10 Wear in metalworking and polishing processes.....................................(P1481)
3.3 Physical and digital modellings, monitoring, prediction, and assessment of wear processes ...................................................................(P1482)
3.3.1 Running-in and surface structure evolution.....................................(P1482)
3.3.2 Physical modelling and testing... (P1483)
3.3.3 Wear monitoring .......................(P1483)
3.3.4 Numerical predictions of wear profile and wear rate .............................(P1484)
3.3.5 Big data, machine learning (ML), and artificial neural network (ANN) application for wear monitoring and analysis of tribosystems ...........(P1486)
3.3.6 Failure analysis, multi-dimensional assessment, and optimization of wear ......................................................(P1487)
3.4 Surface treatments and modifications...(P1489)
3.4.1 Ultrasonic nanocrystalline surface modification (UNSM) ...............(P1489)
3.4.2 Particle shot peening, laser shock peening, and plasma jet hardening.........(P1489)
3.4.3 Reductive surface texturing .....(P1490)
3.4.4 Heat treatment and cryogenic treatment ......................................................(P1490)
3.5 Additive surface engineering ..............(P1491)
3.5.1 Electro-depositions ...................(P1491)
3.5.2 Vacuum depositions .................(P1492)
3.5.3 Friction surfacing (FS) ..............(P1493)
3.5.4 Carbon films...............................(P1493)
3.5.5 Nitriding, carburizing, and other additive surface engineering technologies... (P1494)
3.6 New antiwear materials and friction materials .................................................................. (P1495)
3.6.1 High entropy alloys (HEAs) ... (P1495)
3.6.2 Metallic glasses (MGs) and superelastic metallic materials...................... (P1496)
3.6.3 3D printed materials and sintered materials.................................... (P1497)
3.6.4 New polymer composites........ (P1497)
3.6.5 Brake and friction materials .... (P1498)
4 Biotribology .................................................... (P1498)
4.1 Introduction of biotribology................ (P1498)
4.2 Natural synovial joints and artificial joint replacements.......................................... (P1499)
4.3 Oral ........................................................ (P1503)
4.4 Skin ........................................................ (P1504)
4.5 Others .................................................... (P1505)
5 High temperature tribology ......................... (P1505)
5.1 Introduction of high temperature tribology ................................................................. (P1505)
5.2 High temperature tribology in different applications ........................................... (P1506)
5.2.1 Manufacturing processes.......... (P1506)
5.2.2 Nuclear power generation........ (P1509)
5.2.3 Aero-engines ............................. (P1510)
5.3 Control of friction and wear at elevated temperatures ......................................... (P1511)
5.3.1 High temperature lubricants.... (P1511)
5.3.2 Materials..................................... (P1512)
5.4 Surface modification technologies ..... (P1516)
6 Computer simulations in tribology............ (P1521)
6.1 Introduction of computer simulations in tribology................................................. (P1521)
6.2 Simulation of EHL and mixed lubrication ................................................................. (P1521)
6.2.1 Point contact lubrication problem ... (P1521)
6.2.2 Line contact lubrication problem ... (P1524)
6.2.3 Lubricant theory and design..... (P1527)
6.3 Simulation in contact problem........... (P1529)
6.3.1 Extension of contact model ..... (P1529)
6.3.2 Contact problems of inhomogeneous and layered materials............... (P1533)
6.3.2.1 Inhomogeneous materials .................................... (P1533)
6.3.2.2 Layered materials ...... (P1533)
6.3.3 Contact problems involving multipysical fields ......................................... (P1535)
6.3.3.1 Thermal contact ......... (P1535)
6.3.3.2 Electrical contact ........ (P1536)

6.3.3.3 Magneto-electro-elastic contact..........................(P1538)
6.4 Simulation in wear problem ................(P1539)
6.4.1 Finite element method..............(P1539)
6.4.2 Atomic simulations...................(P1540)
6.4.2.1 Fundamentals of solid friction and wear......................(P1540)
6.4.2.2 Friction and wear of polymer matrix composites......(P1543)
6.4.2.3 Friction and wear of adhesive contact..........................(P1545)
6.4.3 Discrete element method .........(P1546)
6.4.4 Phase-field approach ................(P1546)
6.4.5 Wear simulation of rough surface ...(P1546)
6.4.6 Wear prediction with machine learning algorithms ..................................(P1547)
6.5 Simulation in rough surface.................(P1547)
6.6 Simulation in micro-friction and superlubricity ..................................................................(P1548)
6.7 Tribochemistry.......................................(P1550)
6.8 New simulation methods in tribology—Peridynamics (PD) .................................(P1552)
7 Concluding remarks and prospectives .......(P1553)

## 1 Introduction

The science and technology of friction, wear, and lubrication on interacting surfaces have been described with the term "Tribology" since 1966. The progress in tribology has witnessed heave in the interest from both academic and industrial communities, which plays a significant role in the development of national economy and human civilization. In the pursuit of a comprehensive understanding of recent advances in tribology, this review paper goes through the experiment and fundamental theories governing in lubrication, wear, surface engineering, biotribology, high temperature tribology, and computational tribology, based on the journal papers published in the period of 2020–2021, following previous highlights in the importance, complexities and rapidly growing research interest in tribology during 2018–2019 [1]. The research in tribology is immense and vast, we searched the published articles during 2020–2021 using the keywords such as friction, lubrication, wear, and tribology through the Web of Science, based on the identification of the main journals in tribology and the relevant papers related to theoretical simulation and biological applications of tribological principles. The resulting 3,450 articles were selected, and after looking through, classifying, comparing, and screening, a total of 1,001 articles were accepted as the base materials of this survey, highlighting the development in lubrication, wear, surface engineering, biotribology, high temperature tribology, and computational tribology, as shown in Fig. 1. While this review paper is extraordinarily long, it has been divided into different

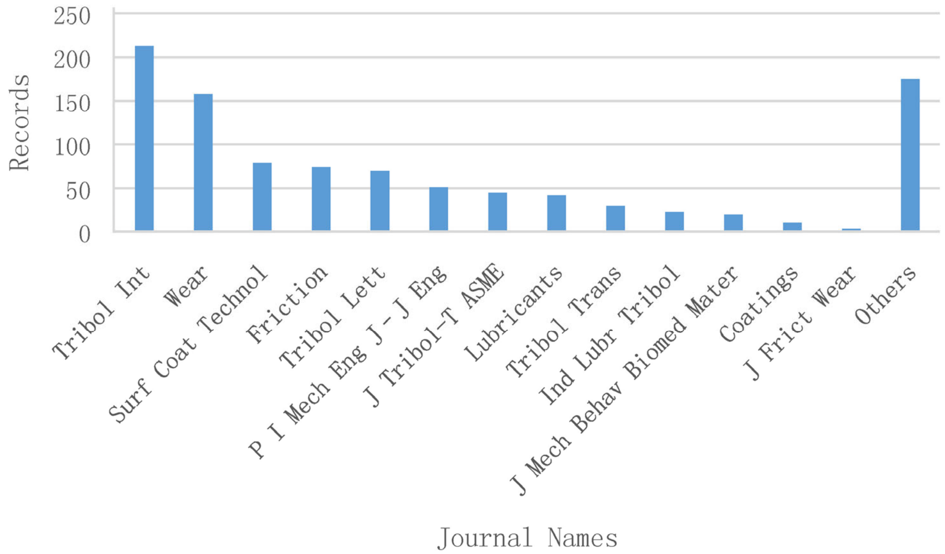

**Fig. 1** Number of references in the main journals in tribology and the related to theoretical simulation and biological applications of tribological principles.

sections and each of its sections has focused on a particular topic (see Contents). Accordingly, the readers may read selectively because it is hardly to read the entire review from the beginning to the end, i.e. preferably reading the parts they are most interested in.

## 2 Lubrication

### 2.1 Introduction of lubrication

In this section, the progresses in researches on lubrication will be exhibited and reviewed. The researches and approaches focused on revealing the lubrication mechanism are overviewed in Section 2.2. The properties of liquid lubrication and solid lubrication including various lubricants are reviewed separately in Sections 2.3 and 2.4. Some special and new type of lubrication materials are also introduced in Section 2.5. In the last part, the studies on applications of lubrication are reviewed.

### 2.2 Approaches for revealing the lubrication mechanism

To look into the lubricating mechanism, many approaches and devices have been developed and utilized, including acoustic emission, optical interference, visualization, sum-frequency generation (SFG) spectroscopy, etc. The influence of pressure, elastic modulus, and surface topographies on lubricating film were analyzed.

Jlaiel et al. [2] established the correlation between acoustic emission signals and elementary tribological mechanisms by an apparatus, equipping with high-speed camera, acoustic emission acquisition system, and force sensors, on transparent materials in ball-on-flat contact. Buse et al. [3] revealed lubricating mechanisms of the calcium hydroxide which can avoid the fretting wear by using a new time-lapse animation visualization technique. Zhang and Glovnea [4] firstly measured lubricant film thickness at the inner and outer raceways in grease lubricated rolling bearings by an original test rig using electrical techniques. Liu et al. [5] reviewed the application of SFG vibrational spectroscopy on experimental configurations, contact and transfer of organic film analysis, molecular orientation alterations analysis, and molecular ordering alterations analysis on the friction interfaces. Bair [6] explored the rheological properties of the liquid film through the quantitative approach in which viscometers and rheometers were used at high pressure and classical approach where the same averaged properties were extracted from the contact itself, and they are essential for understanding and predicting the friction and the film thickness during elastohydrodynamic lubrication.

Based on the optical interference technology, many researches on revealing the lubricating status of the contact area were achieved. Otsu et al. [7] indicated that the film thickness was greater than the surface roughness of specimens in the full-starvation condition, even in low speed cases. Hansen et al. [8] revealed the influence of topographies on elasto-hydrodynamic lubrication (EHL) and pointed out that the micro-conformity play a key-role for EHL lift-off that precedes the completion of running-in. The surface roughness acquisition procedure for re-location of topographies before and after test is shown in Fig. 2.

Macián et al. [9] designed a novel test rig based on the floating liner principle, which can develop multiple parameters tests in a relatively quick and easy way, and presented a theoretical model for the piston compression ring using the finite differences method. Chmelar et al. [10] proposed a method to predict the formation of elastodydrodynamic lubrication regime based on the surface acoustic waves (SAW). It used the sensitivity of Lamb and Rayleigh waves propagation speed to changes caused by the presence of a lubrication film in the roller race contact. Bouchot et al. [11] proposed a new method for quantitative prediction of dry friction based on image processing. The method is carried out in four steps: Pin-on-disk experiment, particle image acquisition, image processing, and quantitative feature extraction of third-body particles. They extracted descriptors for describing contours and textures, which provided a preliminary basis for the subsequent use of machine learning algorithms to better understand the dry contact mechanism.

### 2.3 Liquid lubrication

Liquid lubrication refers to the phenomenon that the

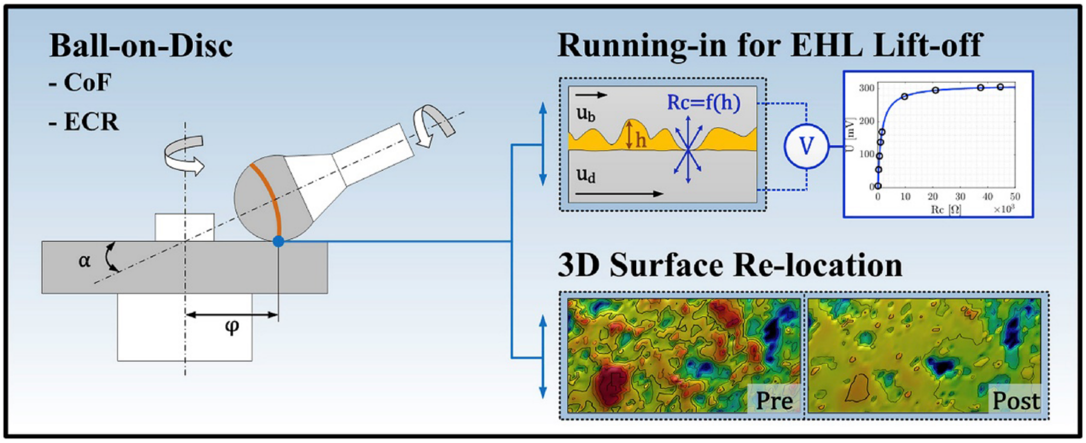

**Fig. 2** Surface roughness acquisition procedure for re-location of topographies before and after test [8]. Reproduced with permission from Ref. [8], © Elsevier Ltd., 2020.

friction force between two surfaces can be reduced through the liquid lubricating film between them, which can also reduce corrosion and protect the surfaces. Here, in this section, liquid lubrication is highlighted and reviewed in several parts, including ionic liquid lubrication, water-based lubrication, emulsion lubrication, and lubricate additives separately.

#### 2.3.1 *Ionic liquid lubrication*

Ionic liquids (ILs) have been known as potential lubricants. ILs can be adsorbed on various interfaces. The adsorbed ILs can avoid the direct "solid–solid" contact, resulting in low friction and wear. It is generally accepted that the lubricity of ILs is determined by their adsorption on the interface and tribochemical reactivity. ILs are used as one type of lubricant and they are also a kind of lubricant additives which can be added into water, greases, drilling mud, and base oil. ILs can be used to improve the suspension of other additives, such as graphene.

ILs can be added into other lubricants as additives to reduce coefficient of friction (COF) and wear rate [12], such as bio-based lubricant [13], water-based lubricants [14], and oil-based lubricants [15], even at relatively high temperature [16]. ILs can increase the stability of lubricant additives [16], such as NiAl particles and trihexyl(tetradecyl)phosphonium cation [17], and improve their tribological performance. ILs can exhibit excellent lubricating performance under different working condition, e.g., under extreme temperature [18], electric field [19], etc.

Gan et al. [20] functionalized multilayer graphene oxide (MGO) using hydroxy-ended ionic liquid coupling agent (ILCAs), and the prepared functionalized GO (ILCAS-GO) showed better dispersion and lubricity in water than the original MGO, the ILCAs-GO on the wear surface provided effective protection and reduced the wear of the material. Schematic about the preparation and modification mechanisms of was shown in Fig. 3.

Aviles et al. [21] prepared a single layer new epoxy resin (RE) coating modified by graphene in aprotic ionic liquid 1-octyl-3-methylimidazolium tetrafluoroborate, RE+9 wt%([OMIM]BF4+0.1 wt%G), protected against wear and reduced the coefficient of friction relative to RE by 60%. Li et al. [22] compared the lubrication performance of ILs under sub-ambient pressures and tribo-degradation performance of two imidazolium tetrafluoroborate ionic liquids (LAB103 and LB104), and found that LAB103 presented better lubricity and lower corrosivity than LB104, due to its strong adsorption layers and robust tribofilms on worn surfaces. Al-Sallami et al. [23] reported the effect of ILs' chemistry on their tribological performance derived from five different types of ILs, and found that increasing the anion/cation chain length reduced the wear and friction coefficients, and the influence of anion and cation types appeared to vary depending on the contact pressure.

#### 2.3.2 *Water-based lubrication*

Water-based lubricants have economic and

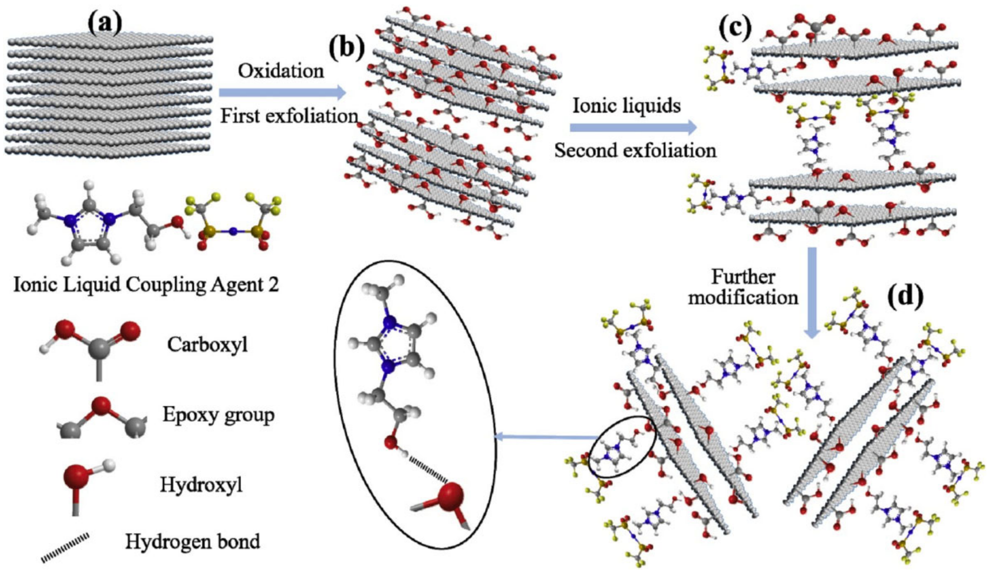

**Fig. 3** Schematic about the preparation and modification mechanisms of ILCAs-GO. (a) Natural flake graphite, (b) MGO sheets, (c) ILCAs-GO sheets, (d) partial enlargement image of junction at ILCAs and oxygen-containing group of GO (take the case of epoxy group) [20]. Reproduced with permission from Ref. [20], © Elsevier Ltd., 2020.

environmentally friendly characteristics and have attracted great attention and expectation. However, they also have drawbacks in term of friction reduction and load bearing capacity. Many schemes have been proposed to enhance the performance of water lubrication. Surface modification and the addition of nanoparticles are the most promising ways because of its low cost, environmental friendliness, and superior improvement in lubrication.

#### 2.3.2.1 Additives in water-based lubrication

The researches on additives used in water-based lubrication were focused on different materials, geometric shape, particle size, and concentration. Chen et al. [24] found that the COF of 100Cr6 ring on 100Cr6 plate reduced by more than 95%, when alkyl glucopyranosides (AGPs) are added into water-based lubricants, which are attributed to the boundary lubrication layer formed by alkyl sugar on the surface. Guo et al. [25] achieved low-friction coefficients (0.01–0.03) by employing self-mated ceramics, self-mated cemented carbide, and cemented carbide/ceramic under water lubrication. Feng et al. [26] found that the deionized water (DI water) containing an electroosmotic promoter (sodium lauriminodipropionate (SLI)) reduced the coefficient of friction and wear scar diameter, whereas, that with an electroosmotic suppressant (cetyltrimethylammonium bromide (CTAB)), exhibited an opposite behavior. Ji et al. [27] reported that glyceryl monostearate (GMS)-filled microcapsules largely improved the tribological properties of drilling mud. Liu et al. [28] found that after normalizing the friction force by the contact area, the shear stress of hydrogels with either crosslinked or brushy surfaces was similar for both contact geometries at low speeds, indicating that hydrogel friction is unaffected by the contact geometry at these speeds. Tang et al. [29] prepared a water-based lubricant with 0.005 wt% black phosphorus quantum dots (BPQDs), which reduced the friction coefficient and wear volume by 32.3% and 56.4%, respectively, and enhanced the load-carrying capacity and lifetime, ascribed to the small and uniform particle size of the BPQDs, excellent dispersion stability in water, and high reactive tribo-chemical film during rubbing.

Wang et al. [30] found that the 70 mg/L BP nanosheets as water-based lubrication additives (BP-WL) exhibited optimal friction-reducing and anti-wear performance, which were closely associated with their lamellar structure, and the adsorption and tribochemical films formed on the worn surfaces, respectively. Zhang et al. [31] found that the film-forming ability of water and tribological behaviors of water-lubricated contacts can be improved by injecting of #5 white oil. They provide a new method to reduce the friction coefficient and wear rate of water-lubricated bearings. Jiang et al. [32] demonstrated whether the superlubricity of $Si_3N_4$/glass interface lubricated with $H_3PO_4$ degenerates depends on the substrate surface topographies induced by different shearing behavior, and established the relationship between lubrication state and surface roughness of superlubricity system. The evolution of surface roughness in worn region on glass substrate was characterized using atomic force microscope (AFM), as shown in Fig. 4.

Li et al. [33] found that the added silicon nanoparticles into water significantly reduce the coefficient of friction and the time of running-in. Lubricant additives are added at different time points to ensure the effect on running-in duration, which attribute to surface film formed by the depositing of nanoparticles. Wei et al. [34] prepared GO/chitosan-g-PNIPAM nanohybrids by combining GO and brush-like chitosan-based copolymer (chitosan-graft-poly (N-isopropylacrylamide), chitosan-g-PNIPAM) for water-based lubrication and found that it reduced the average COF by 40% compared to raw GO and by 84% compared to a single copolymer, and reduced wear rates by 15% and 47%, respectively. Bin Abdollah et al. [35] proposed that increasing the amount of hexagonal boron nitride additive may cause the lubricating state to change from mixed lubrication to hydrodynamic, allowing nanoparticles to aggregate, resulting in increased friction. Liu et al. [36] found the surface modification endowed $SiO_2$ with smaller particle size and better dispersibility, significantly reduced the wear of $SiO_{2(TESPT)}$/NBR.

#### 2.3.2.2 Influence factors of water-based lubrication

In addition, influence factors, including the load, surface treatment, and velocity on water-based lubrication have been studied. Xie and Zhu [37]

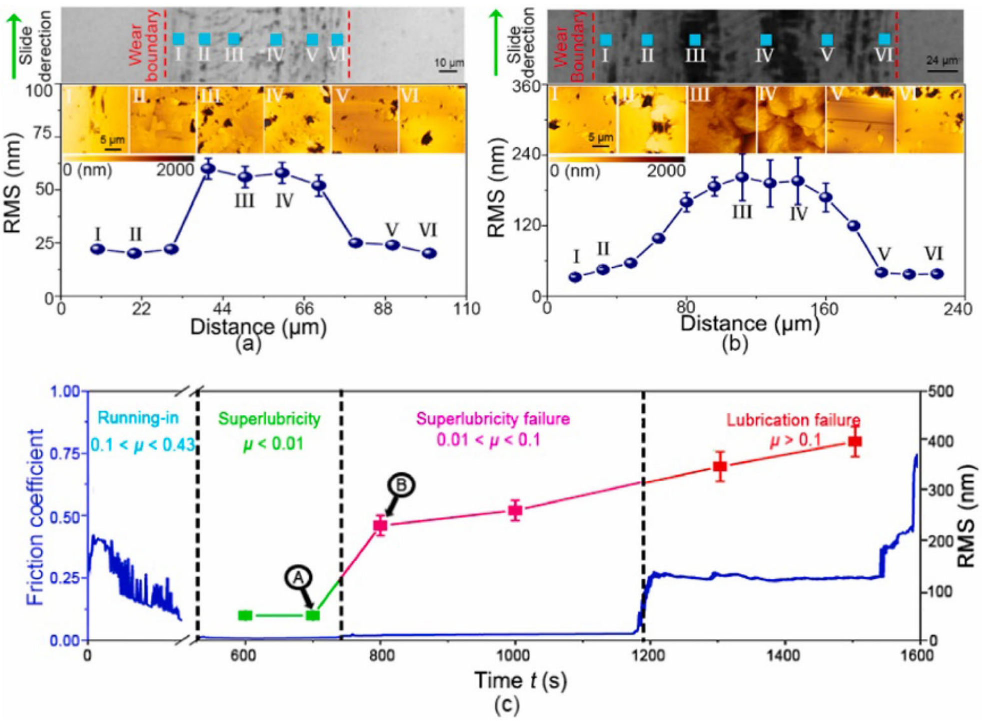

**Fig. 4** Evolution of surface roughness in worn region on glass substrate characterized using AFM. (a) and (b) respectively show the variations of surface roughness in each AFM scanning area of 20 μm × 20 μm along the cross-section of wear scars formed at the sliding time of 700 s (marked with A in (c)) and 800 s (marked with B in (c)) corresponding to the stages of superlubricity and superlubricity failure. The upper optical pictures in (a) and (b) show the wear tracks with the widths of 100 and 200 μm formed under these two conditions. The middle insets of (a) and (b) show the representative AFM images (20 μm × 20 μm) of the worn regions marked in the upper optical pictures. (c) Root mean square (RMS) roughness of the center area (within 40 μm of scratch center) of wear track on glass substrate under different lubrication states obtained using AFM. $F$ = 6 N and $v$ = 0.5 m/s. The error band represents a standard deviation [32]. Reproduced with permission from Ref. [32], © Elsevier Ltd., 2021.

investigated the micro asperity contact load ratios and lubrication states of the water-lubricated bearing, proposed a new coupled models with consideration of micro-scale asperity contact effects, and pointed out that the asperity contact load ratios can be used as the judging criterion for the interface lubrication regimes transition for water lubricated stern-tube bearings. Cao et al. [38] found that the carbon fiber reinforced glass fabric/phenolic laminate composite with a 0.2 wt% graphene oxide modified on the carbon fiber shows the lowest friction coefficient and specific wear rate when siding against the stainless steel under water lubrication. Lin et al. [39] predicted dynamic characteristics of high-speed water-lubricated spiral groove thrust bearing (SGTB) considering cavitating effect. Bahrami et al. [40] prepared a surface-attached hydrogel layer by coating the substrate with a copolymer containing benzophenone groups and a subsequent UV irradiation. Yang et al. [41] showed macroscale liquid superlubricity on steel tribo-pair lubricated by glycerol aqueous solutions with water/glycerol weight ratio of 0.2 within 40 s.

#### 2.3.3 *Emulsion lubrication*

Emulsions are widely used in tribological applications in order to facilitate operation (cutting or shaping) of metals. The O/W emulsions are mixtures of water, base oils, and additives. The oil phase acts as a lubricant, reducing the friction between workpiece and tool, while the water phase promotes the cooling for different machining processes. This fluid is suitable for turning, milling, and grinding process due to the use of new cutting tool materials, such as hard metals and high cutting speeds. The application of emulsion in tribological field is briefly introduced in this section.

Many researchers have studied the effect of emulsion composition on tribological properties. Yang et al. [42] investigated the effect of amines on the lubricity of castor oil-sulfated sodium salt (CSS) emulsion solution for titanium alloys and found that the amines with hydrophilic parts such as diglycolamine, have a negative effect on the lubrication while those with hydrophobic parts such as dicyclohexylamine (DCHA), show a synergistic effect when mixed with the CSS. Xue et al. [43] studied the tribological behaviors of different interfaces under dry friction and oil lubrication. They proposed that the reduction of fluctuation amplitudes of friction force and the improvement of bearing capacity of oil film are attributed to the synergistic effect of Sn–Ag–Cu–$Ti_3C_2$, oil, and grooves fabricated on the surface. Yang et al. [44] found that the Oct–O–GO (oxidation degrees 1) emulsion exhibited the best load-carrying capacity, and the best friction-reducing and anti-wear properties compared to other emulsions. Chen et al. [45] studied the relationship between the tribological properties and oxidation degree of graphene derivatives in O/W emulsion, and found that the tribological properties of MGO1-emulsion (graphene oxide modified with octadecylamine on a single side) were better than that of MGO2-emulsion (graphene oxide modified with octadecylamine on double side).

The influence of emulsion on workpiece was also studied. The cleaning of food production equipment using cleaning detergents may contaminate the lubricant of the bearings, thereby reducing the bearing service life. Chen et al. [45] also investigated the cause and mechanism of such damage of bearings lubricated by cleaning detergent/water-in-oil emulsions. The emulsion can induce damages on the soft surface in the startup mainly due to the presence of water around the contact.

#### 2.3.4 *Grease lubrication*

Unless maintained in a sealed enclosure, fluid lubricants are difficult to retain at the point of application and must be replenished frequently. If, however, the fluid lubricant is thickened, its retention is improved and the lubrication intervals can be extended. This is exactly grease, which can simply be thought of as a lubricating fluid that has been gelled with a thickening agent so that the lubricant can be retained more readily in the required area. Lubricating greases have unique advantages over lubricating fluids in some cases. The behaviors and properties of grease lubrication are reviewed in this section.

Ghezzi et al. [46] found that the rheological and physiochemical interactions between the thickeners, the additives, and the base oil of grease lead to different frictional trends. Their results of grease lithium complex PAO (LiCPAO) and base oil PAO

showed the contribution of thickeners and additives affects mainly the frictional response in static conditions and within the low-velocity range. At the same time, Feng et al. [47] established the relationship between rheological and the frictional properties, which exhibited that the loss modulus of friction-enhancing grease is proportional to the friction coefficient. In terms of the impact of base oils, the investigation of Buyanovskii et al. [48] showed that bentonite lubricants prepared on the basis of industrial oil I-40A have a higher lubricating ability than compared greases based on polyalphaolefine oil PAO-10, including the temperature stability and the friction reduction effect of some additives, such as polytetrafluoroethylene (PTFE) and BN, can be more significant on the grease based on I-40A.

On further investigation of grease film formation, Li et al. [49] found that the evolution patterns of the grease films were highly related to the speed ranges and grease structures in rolling EHL contacts, and they clarified the primary mechanisms that dominate grease film formation in different lubricated contacts (Fig. 5). Han et al. [50] found that the grease film thickness did not become nearly constant until the stroke ends, because the reciprocating motion reduced the accumulation of the thickener fiber gradually with time and after enough working cycles, very severe starvation occurred. For situations where a boundary lubrication regime predominates in high load oscillation motion, Frache et al. [51] studied the mechanisms involved in protecting contact surfaces of commercial lubricated deep groove ball bearing. The analyses revealed a modified layer at the contacts generated by grease interactions.

On the other hand, the friction performance of greases is closely related to temperature. Georgiou et al. [52] measured the adhesion and tackiness of greases with the indentation retraction approach and found that the ability of a grease to adhere to a surface (pull-off force) and to form threads when it is being pulled apart (tackiness) strongly depended on the temperature. Joysula et al. [53] improved antiseizure and friction performance of grease without modifying the grease chemistry. The mechanism can be related to the delay in rise of local temperature, which was theoretically attributed to an increase in heat loss as the speed ramp-up time was increased. Kunishima et al. [54] found that temperature and addition of zinc carboxylate to grease promoted the formation of a carboxylate tribofilm on the steel surface and a zinc sulfide reactive film on the polyamide 66 (PA66) surface under boundary lubrication, which contributed to an improvement of the tribological properties, particularly at 80 °C (see Fig. 6). Qi et al. [55] found that adding antioxidants to additives to reduce the antioxidant reaction of additives can enhance the temperature stability and antiwear properties of lubricating greases.

The friction performance of greases can be significantly improved by the addition of nanoparticles. Kumar et al. [56] reported the potential of nanoparticles (NPs) of Talc to improve the tribo-performance of Li-based grease in antiwear and extreme-pressure. What's more, Rylski and Siczek [57] and Zhang et al. [58] found that the improvement of the dispersibility of nanoparticles can effectively improve the tribology performance of lithium grease with preventing the agglomeration of nanoparticles. These are consistent with the study of Wang et al. [59] about the influence of the variation of morphology on the tribological performance of layered double hydroxide (LDH). They found that the variation of morphology, instead

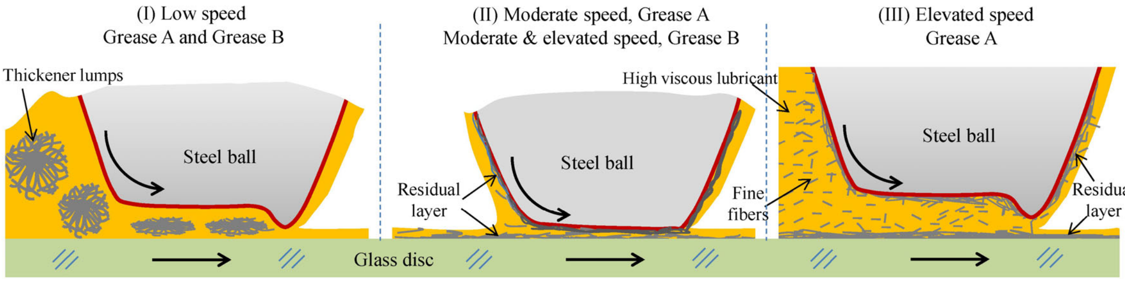

**Fig. 5** Schematic of lubrication states over different speed ranges for grease A and B [49]. Reproduced with permission from Ref. [49]. © The author(s), 2020.

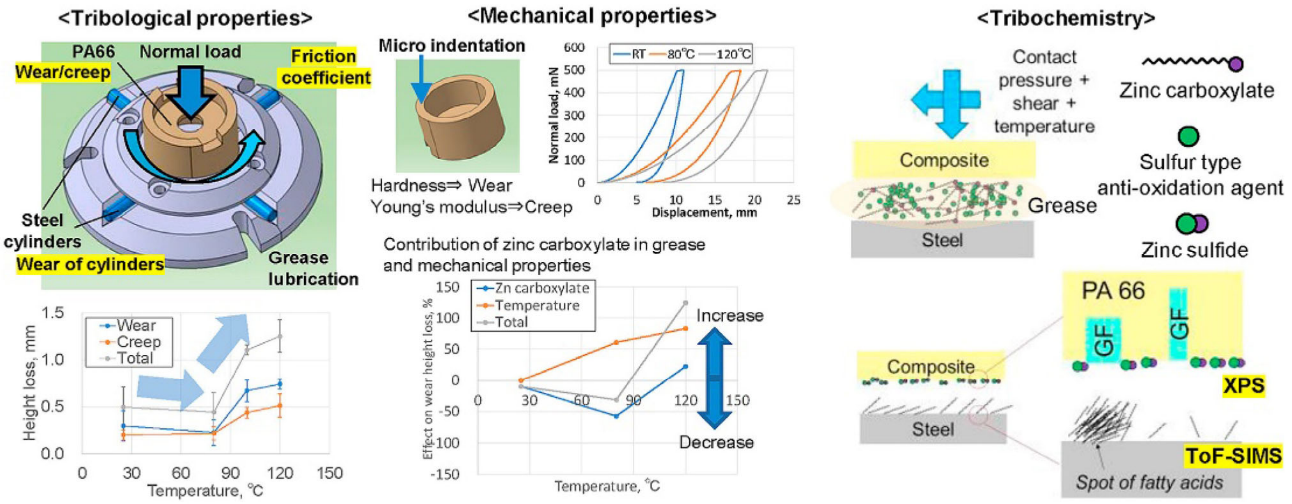

**Fig. 6** Properties of glass-fiber reinforced PA66 using as friction pair [54]. Reproduced with permission from Ref. [54], © Elsevier Ltd., 2021.

of chemical composition, had great influence on the tribological performance of LDHs, and the sample with high specific surface area demonstrated the best performance.

For evaluation of grease performance, Sanchez et al. [60] and Kanazawa et al. [61] showed that analysis of single-contact ball-on-disc tests can provide a fast tribological evaluation of grease performance.

### 2.3.5 *Lubricant Additives*

Developing additives with specific functions are one of the most effective ways to reduce friction and wear. 2D materials, such as graphite, nanocarbons, molybdenum disulfide, sodium tetraborate, cadmium chloride, boron nitride, lead iodide, cobalt chloride, silver sulfate and zinc stearate, and sulfides, tellurides of metals, such as $MoS_2$, $WSe_2$, $WS_2$, $NbS_2$, $NbSe_2$, $TaS_2$, $TaSe_2$ are good additives to reduce friction energy losses. In this part, the friction and wear reducing effect of these additives are summarized.

Graphene-based materials are typically used as a lubricant additive because of their layered structure, such as GO-$Fe_3O_4$ nanohybrids [62], functionalized graphene/montmorillonite (FG/MTT) nanosheet [63], titanium dioxide ($TiO_2$) and graphene(GnP) nanoparticles [64], graphene/ionic liquid (G/IL) composite material [65], graphene modified by octadecylamine and dicyclohexylcarbodiimide [66], detonation nanodiamond and GO [67], graphene nanoparticles [68], graphene/triangular copper nanoplate composites (GN/Cu nanoplates) [69], and nanomaterials graphene nanoplatelets [70], etc.

Oxide and metallic nanoparticles can also be used as effective lubricating additives, such as Ni nanoparticles [71], $SiO_2$ nanoparticles [72–74], CuO nanoparticles [75], oleylamine (OM)-modified $CeO_2$ nanoparticles [76], $SiO_2$@Cu microspheres [77], $ZrO_2$ nanoparticles [78], carbon quantum dot (CQD) and Ni-CQD particles [79], Cu nanoparticles [80], cubic nickel nanoparticles modified with oleymine [81], some magnetic nanoparticles [82], nano-silver [83], etc. Nanofluids formed from metal compounds also work well as lubricant additives [84–87]. Lubrication additives can also be obtained by modifying Zn/ZnO (zinc dialkyl dithiophosphates, ZDDP) [88–94]. Wu et al. [95] synthesized lithium complex grease (LCG) added with carbon black (C), $Fe_3O_4$, and $Al_2O_3$ nanoparticles on rolling bearing and found that nanoparticles were beneficial to enhance the friction and wear performance (see Fig. 7).

Organic additives can also provide effective lubrication, such as fatty acid methyl esters [96], boron-nitrogen modified soybean oil [97], moringa oil [98], organic friction modifiers (OFMs) [99], isooctyl furan dicarboxylate (isooctyl-FD) [100], N-octadecyl-D-gluconamides (NOG) [101], diblock polymeric friction modifier (PFM) [102], transesterified olax scandens oil [103], polyisobutylene (PIB) [104], thermo-oxidized Jatropha bio-oil [105], methyl oleate derived multifunctional additive [106], oleic acid

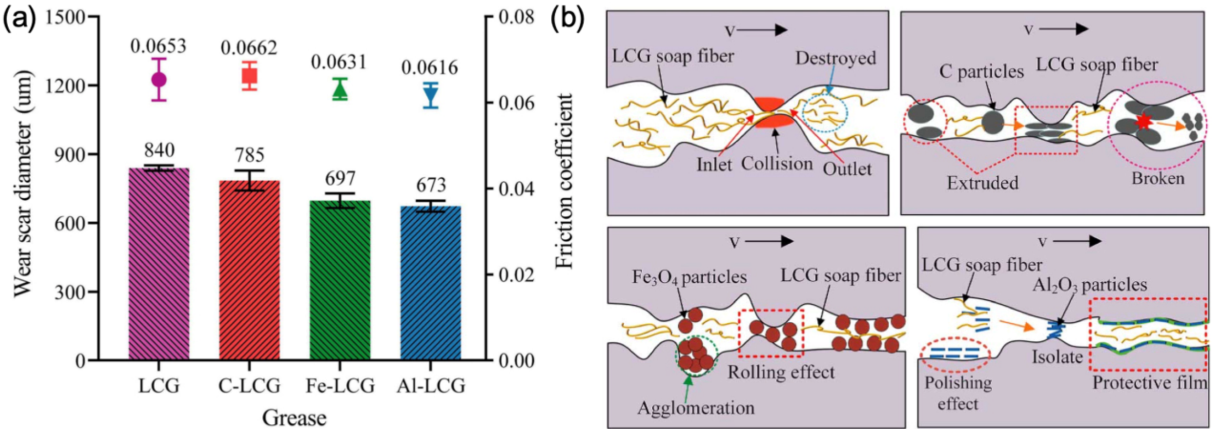

**Fig. 7** (a) Friction coefficient and wear scar diameter of LCG, C-LCG, Fe- LCG, and Al-LCG (four-ball, 1,200 rpm, 392 N, 60 min). (b) Schematic diagrams of the lubricating mechanism of four kinds of greases [95]. Reproduced with permission from Ref. [95], © Elsevier Ltd., 2021.

cholesterol ester [107], glycol alkanes [108], 1,3-diketone EPND (1-(4-ethyl phenyl) nonane-1,3-dione) fluid [82], pyrone esters (PEs) novel lubricant additives [109], fructose consisting of ethylene glycol (EG) and 1,3-propanediol (13-PD) [110], glycerol monooleate (GMO) [111], oleic acids (OA) [75, 85, 111, 112], lauric acid methyl ester [113], and microcrystalline cellulose [114], etc. Friction behavior of pure EG and fructose/EG solutions was shown in Fig. 8 [110].

In addition, sulfide can also be used as lubricating additives. $MoS_2$ is a typical sulfide used as a lubricant additive [115–121]. Liu et al. [122] achieved the active control of boundary lubrication in $MoS_2$ particle suspensions by modifying the surface charging state of the particles and applying an electric field and found that the applied potential increased the thickness of the tribofilm with a relatively thick and loose structure, which played a key role in reducing the shear force.

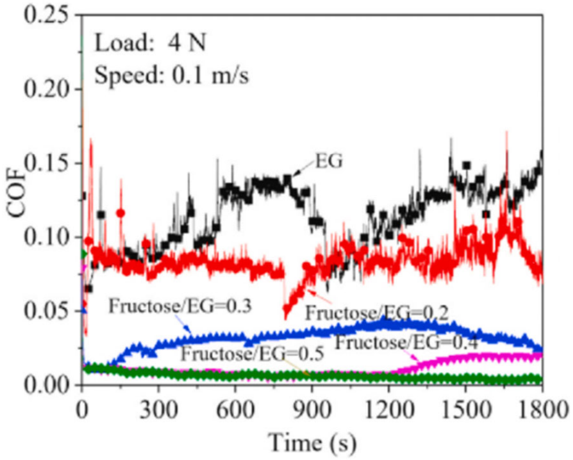

**Fig. 8** Friction behavior of pure EG and fructose/EG solutions [110]. Reproduced with permission from Ref. [110], © Elsevier B.V., 2021.

ILs can be used as lubricating additives. Jiang et al. [123] synthesized an oil soluble multifunctional protic ionic liquid, which significantly reduced friction and wear of PAO4. Syahir et al. [124] found that adding ILs into PAO8 and trimethylolpropane trioleate (TMPTO) can further reduce friction coefficient, lower overall surface wear, and improve surface finishing. Kawada et al. [125] confirmed the ionic liquids underwent tribo-decomposition on nascent steel surfaces, and a stable tribofilm was formed. Yue et al. [126] synthesized two kinds of novel halogen-free liquids additives [DHB][BScB] and [MA-BIm][bis(salicylato)-borate, BScB].

Nanomaterials are effective lubricant additives, such as lanthanum borate nanospheres (LBN) [127], core-shell nanocomposites [128], 2D layered α-zirconium phosphate (α-ZrP) nanosheets intercalated with different amines [129], novel palygorskite nanoplatelets [130], biocarbon-based magnesium silicate hydroxide nanocomposite [131], COOH–functionalized multiwalled carbon nanotubes [132], and nano-PTFE particles [133], etc.

In addition, there are other lubrication additives, such as magnetic fluids [134], h-BN [135], and liquid crystal additive [136]. Wang et al. [137] prepared two kinds of novel functionalized phosphate IL additives, which can realize transition from mixed lubrication

(ML) to EHL at a smaller entrainment velocity (*U*e) and improve lubricating properties (see Fig. 9).

## 2.4 Solid lubrication

Due to the limitations of liquid lubrication in certain applications, solid lubrication has also developed considerably. Two-dimensional materials are crystalline or amorphous material with thickness of only one single or multiple layers of atoms. The excellent physical, mechanical, and chemical properties of two-dimensional materials bring new opportunities to the design and development of nano-scale lubricating materials.

### 2.4.1 *Carbon-based materials*

#### 2.4.1.1 Graphite and derivatives

Graphite is a traditional solid lubricating material with layered crystal structures. Layer by layer van der Waal force bonding has a very low interlaminar shear strength, resulting in good lubrication performance. Feng et al. [138] achieved the state of superlubricity steadily on a highly oriented pyrolytic graphite (HOPG) surface at the atomic-scale by an appropriate combination of amplitude and frequency. However, due to the topographic height change, there was a resistive force during the step-up motion and an assistive force during the step-down motion when the tip slid over a buried graphene step edge [139]. The mechanistic model of graphite- and graphene-based surface protective films absorbed on the metal substrates [140] and the role of graphite/matrix interface in low friction behavior were investigated. Fu and Duan [141] carried out molecular dynamics simulations to study the effects of environmental moisture and functional groups on the sliding adhesive behaviour of graphene steps at the atomic scale. The abrasion resistance of graphene sheets in a water environment is better than that in a vacuum environment. Motozuka et al. [142] demonstrated

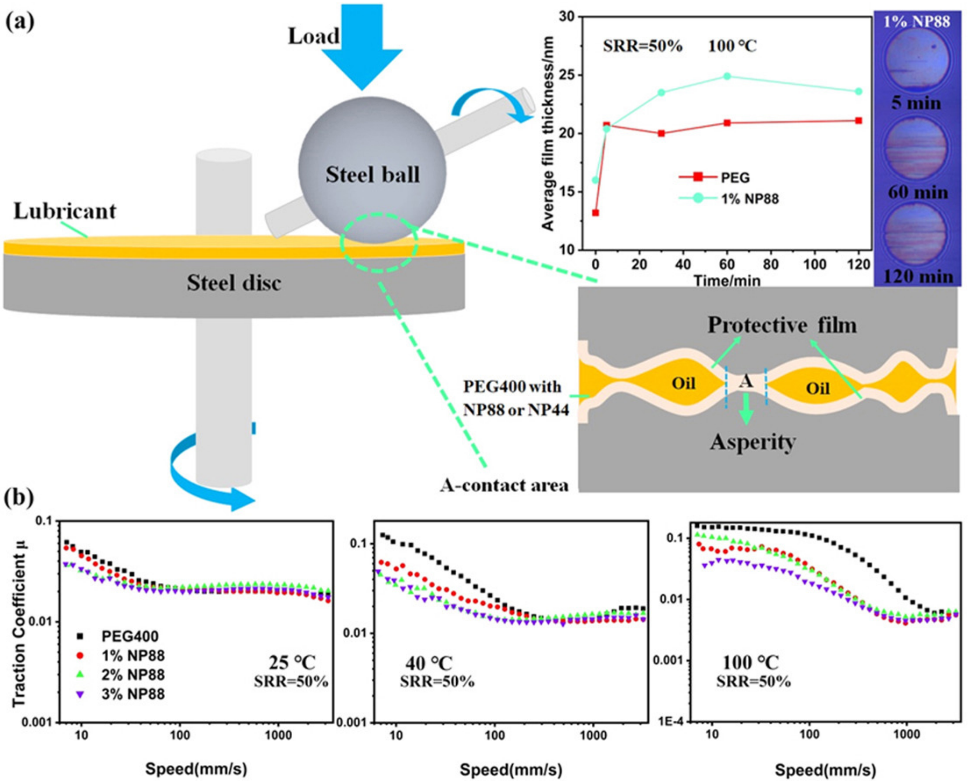

**Fig. 9** (a) Schematic of the proposed mixed lubrication mechanism of polyethylene glycol (PEG)400 with NP44 or NP88 and the variation of average film thickness with rubbing time under the lubrication of PEG400 and 1% NP88 at *U*e of 50 mm/s, load of 20 N, slide-to-roll ratios (SRR) of 50% and temperature of 100 °C. (b) Stribeck curves of PEG400 and PEG400 with different concentrations of the NP88 at temperatures of 25, 40, and 100 °C, SRRs of 50%, and load of 20 N [137]. Reproduced with permission from Ref. [137], © Elsevier Ltd., 2021.

that the graphite base plane was oriented along the oxidized surface, but not along the exposed iron surface, which explained the obviously increased friction in Ar–$H_2$ atmosphere (Fig. 10).

Graphene or graphene derivatives have a significant effect of reducing friction and wear of the composites [143] with different contents, as well as in other friction pairs such as steels, ceramics, and alloys [144]. The lubrication mechanism is mainly attributed to the fact that graphene or graphene derivatives enter the contact area to form a friction protective film, which reduces wear and reduces friction significantly due to the weak interlayer force of graphene nanosheets. Chen et al. [145] fabricated a series of graphene oxide composite lubricating films, which exhibit excellent tribological properties under harsh environment. Zhen et al. [146] concluded that a high content of graphite was readily oxidized and formed tribolayer films of more high-strength carbides in high temperature, thus improving the tribological behavior in the range of 24–400 °C. Feng et al. [147] found that the dense microstructure in the graphene and oxide (GNs/AT13) composite coating can form a transfer layer to significantly protect and lubricate the friction interfaces. Similarly, Wu et al. [148] observed that the reduced graphene oxide containing nanocomposite on the polyimide (PI) can be slowly released to the interface during the sliding friction process to form a protective film, which not only solves the aggregation problem of PI, but also reduces the wear rate of the PI surface. What's more, the increasing amount of graphene contents in the composite matrix can improve the mechanical properties, which changes the dominant wear mechanisms [149] and increases the abrasion resistance of the woven glass fiber reinforced epoxy resin (GFRE) composites [150]. Zhao et al. [151] found the stone-wales defect on the top layer of thin film contributes the most to lubrication, while single vacancy defect is the key point to promote the adhesive wear of the thin film. They provide an atomic-level evidence to the tribological characteristics of film composed of graphene and h-BN. Zhang et al. [152] used three typical surfactants and saccharin to disperse GO nanoparticles and refine crystallite size of nickel/GO composite coating. The tribological test indicates that the PEG–GO coating can achieve the maximum reduction of friction coefficient with 0.15 with the addition of saccharin of 0.2 g/L. Shi and Jiang [153] investigated the tribological properties of graphene/

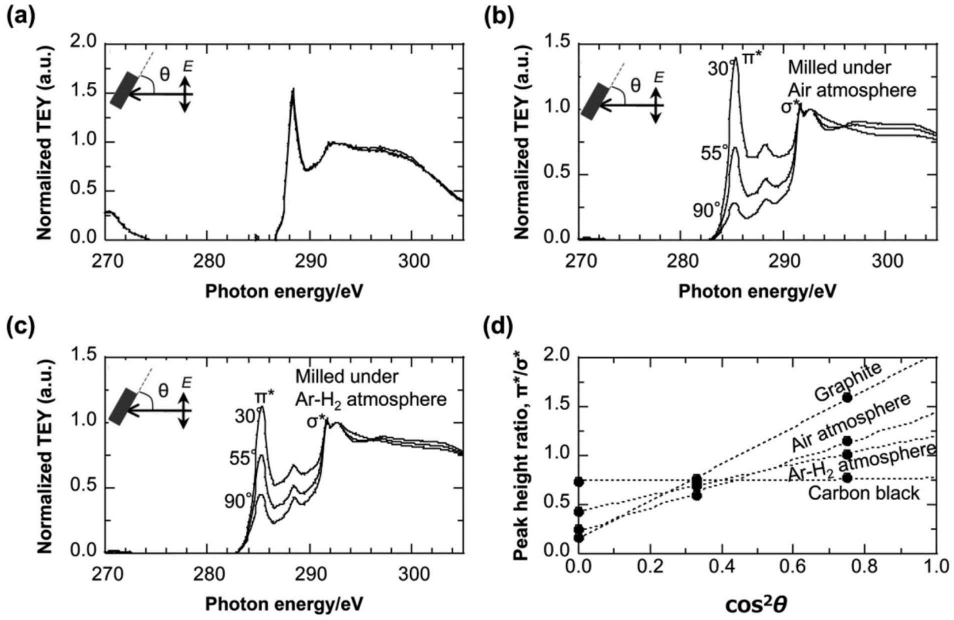

**Fig. 10** Incident-angle dependent C K-edge X-ray adsorption near-edge structure (XANES) of iron powders milled (a) without graphite, and with graphite (b) in air and (c) in an Ar–$H_2$ gas, and (d) variation in the peak height ratio of π*/σ* among four specimens; graphite, two iron powders milled in air and in an Ar–$H_2$ gas and carbon black [142]. Reproduced with permission from Ref. [142], © Elsevier Ltd., 2020.

copper hybrid particles and hydroxypropyl methylcellulose (HPMC) prepared into composite solid lubricating coatings. The addition of hybrid particles to the solid lubricant effectively reduced the friction coefficient by 35% and the wear volume by at least 50%. Avilés et al. [154] found that the tribological properties of epoxy resins modified by graphene and ionic liquids were greatly improved. A single layer coating of RE modified by graphene and aprotic ionic liquid 1-octyl-3-methylimidazolium tetrafluoroborate, prevented wear and reduced the coefficient of friction.

In addition, many efforts have also been made to improve the tribological performance by adding the graphene and its derivatives as lubricating additives into water, oils [155], greases [156], and so on. For example, Xie et al. [157] achieved better load-carrying capacity and better high temperature lubrication ability of wax with the addition of multilayer graphene. Further, many scholars choose to modify graphene, which is mainly to improve the solid lubrication as well as the dispersity and stability of graphene in lubricants as lubrication additives. The influences of the treatment methods [158] and the functionalized graphene coating on the tribological performance of steels were investigated, and found that friction layer formed *in situ* can effectively prevent the substrate from oxidation and improve the anti-wear strength greatly [159]. Li et al. [160] found that incorporated nano-$SiO_2$ particles have roles in promoting the formation of graphene nanoscrolls and exhibiting load-carrying capacity for achieving macroscale superlubricity. Gan et al. [159] prepared the functionalized GO (GO-EmimN(CN)2) with extraordinary dispersity and high exfoliation degree to dramatically decrease the wear of GCr15 steel as water-based lubricant additives. Han et al. [161] found the modified graphene can be steadily dispersed in PEG200 and reduce friction coefficient and wear, attributed to protective layers on the interface (Fig. 11).

#### 2.4.1.2 Diamond like carbon (DLC) films

Among many types of carbon materials, DLC films have attracted wide attention worldwide due to their excellent properties. The structure of DLC film is between diamond and graphite structures, which is mainly composed of complex three-dimensional network structure formed by mixing $sp^3$ hybrid carbon atoms of diamond structure and $sp^2$ hybrid carbon atoms of graphite structure. Therefore, DLC film not only presents good tribological properties, but also has good corrosion resistance and anti-wear performance, with high hardness, high thermal conductivity, low dielectric constant, wide band gap, good optical transmittance, excellent chemical inertia, and good biocompatibility.

There are many methods for preparing DLC films, and related technologies emerge endlessly, which help to prepare films with corresponding performance requirements for different application occasions. It has been reported that the plasma-enhanced techniques [162] are effective to deposit DLC coatings to improve adhesion with the surfaces of friction pairs, mechanical properties, and thereby tribological behavior, including high power impulse magnetron sputtering (HiPIMS) [163], plasma enhanced chemical vapor deposition (PECVD) [164], and plasma ion immersion deposition (PIID) [165]. The DLC that presented the best tribological behavior was the one deposited by PECVD due to its highest $H/E$ ratio. In addition, the ultra-short pulsed laser irradiation was used by Dorner-Reisel et al. [166] to prepare the μ-patterned DLC with ultralow wear rate of $0.40\times10^{-7}$ $mm^3/(N\cdot m)$, if there is hyaluronic gel lubrication.

DLC films usually present amorphous or amorphous nanocrystalline composite structures based on the characteristics of crystal materials. According to the presence or absence of hydrogen, it can be divided into DLC films containing hydrogen (a-C:H) with different hydrogen content and the ratio of $sp^3$ to $sp^2$ hybridization bond and DLC films without hydrogen (a-C). The DLC coatings of hydrogen-free a-C and hydrogenated a-C:H [167] can significantly reduce friction and increase wear resistance of substrates (Fig. 12). Among them, hydrogen incorporation played positive roles in reducing surface roughness and achieving better tribological performance of DLC films [168], which was governed by the interfacial adsorbates and carbon dangling bonds, leading to reduced interfacial interactions [169]. And the removal of the oxide layer with decreased oxygen concentrations in H-DLC was observed to result in friction reduction during running-in [170].

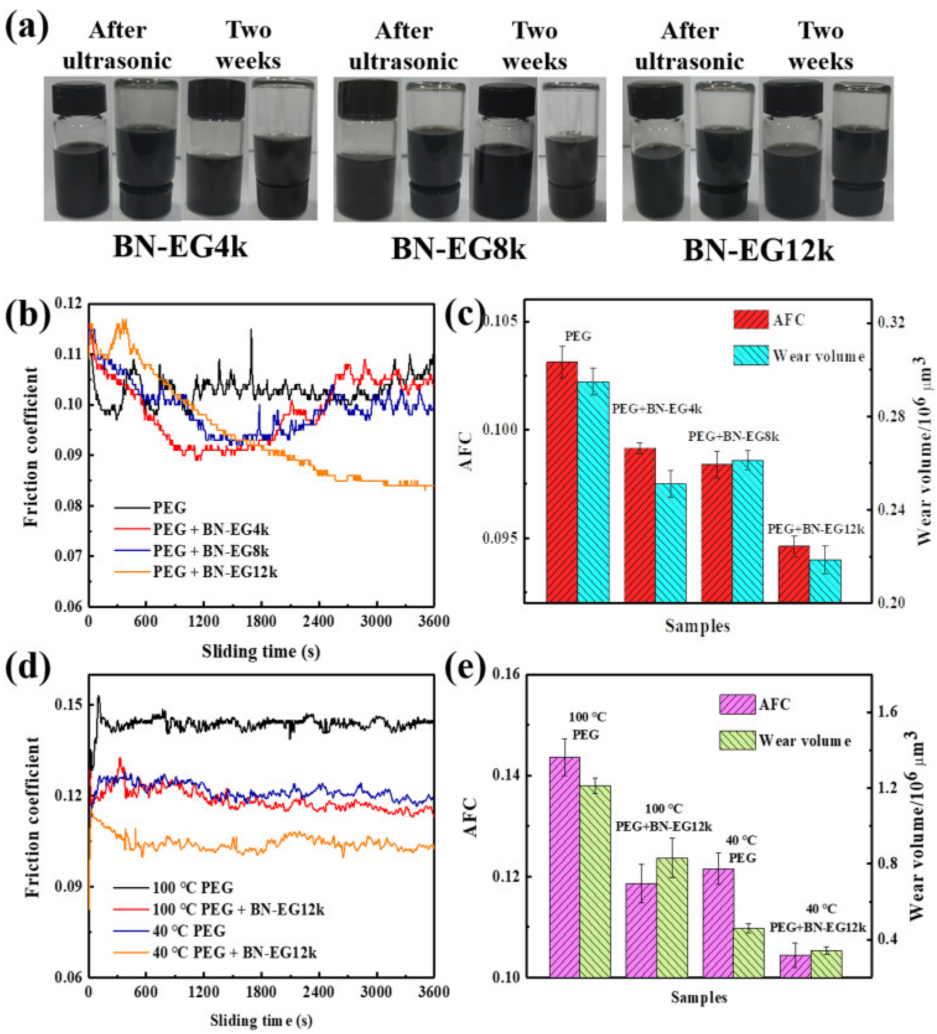

**Fig. 11** (a) Images of BN-EG4k, BN-EG8k, and BN-EG12k dispersing in PEG200 and comparison after two weeks. (b) Curve of their friction coefficient with sliding time at RT; (c) histograms of average friction coefficient (AFC) and wear volume. (d) Curve of their friction coefficient of the BN-EG12k-containing lubricant and pure PEG200 at temperature of 40 and 100 °C; (e) histograms of AFC and wear volume [161]. Reproduced with permission from Ref. [161], © Elsevier Ltd., 2021.

For DLC films serving in special environments, such as parts with high load and high speed, appropriate doping will be carried out on DLC films to change the bonding mode of the cross-linked carbon matrix hybrid network inside the film and the chemical state of the film surface, as well as the surface texturing, so as to improve the performance of the film and realize the application in actual working conditions. Doping of hydrogenated amorphous carbon DLC exhibited significant improvement of tribological properties further, such as single-layer tungsten-doped (a-C:H:W) [171] and multilayer silicon oxide containing $(a\text{-}C:H:Si:O/a\text{-}C:H)_{25}$ amorphous carbon coatings [172], silicon-containing hydrogenated amorphous carbon DLC (a-C:H:Si) [173], and so on. The friction and wear behavior of DLC was revealed to mainly depend on their surface topography [174], and a considerably lower friction coefficient was achieved under severe working conditions by DLC coatings combination with the texturing [175].

What's more, the doping of metal elements in DLC films, including Ti-doped (Ti-DLC) [176], Al and Ti co-doped (Al/Ti-DLC) [176] (see Fig. 13), Ag-doped (Ag-DLC) [177], Ni and B co-doped (Ni/B-DLC) [178], improved tribocorrosion resistance and wear resistance performance of DLC films, mainly originating from the reduction of pure mechanical wear with the increase of hardness and elastic modulus. However,

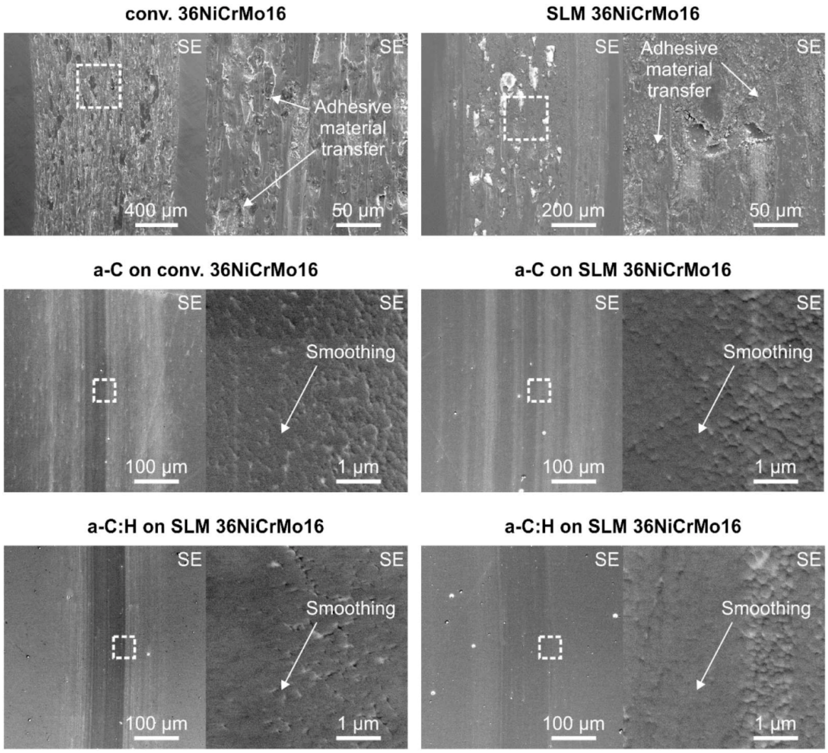

**Fig. 12** SEM micrographs of the wear tracks of uncoated 36NiCrMo16 and a-C(:H) films on 36NiCrMo16, produced by conventional technique and selective laser melting (SLM) [167]. Reproduced with permission from Ref. [167], © Elsevier B.V., 2020.

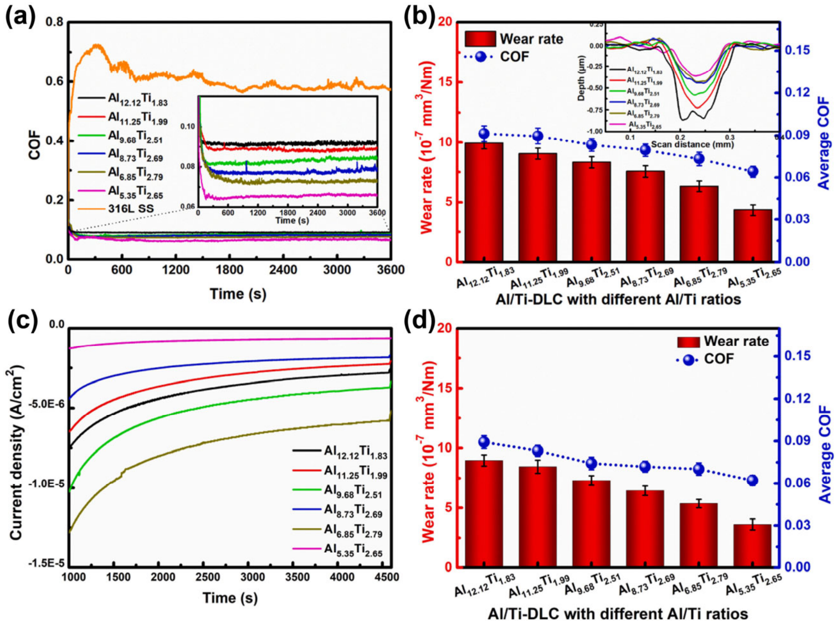

**Fig. 13** (a) Friction curves and (b) average wear rate and COF values of films in 3.5 wt% NaCl solution, in which the insets are enlarged friction curves and depth profiles of the wear tracks, respectively. (c) Current evolution curves and (d) average wear rate and COF values of films under cathodic protection conditions [176]. Reproduced with permission from Ref. [176], © Elsevier B.V., 2020.

some doping or modification may lead to severe wear of the friction pair material. Zhang et al. [179] reported that the biomolecules adsorbed on the fluorinated DLC films inhibited the formation of the tribofilm, which improved the friction coefficient and wear of the Ti6Al4V counterpart.

2.4.1.3 Other carbon-based materials

Carbon nanotubes (CNTs) are tubular structure molecules formed by carbon atoms. Their structure is a hollow cylinder surrounded by graphite surfaces, which has excellent self-lubricating performance. As additives, CNTs can not only significantly reduce the friction coefficient of materials, but also effectively improve the wear resistance [180]. Kałużny et al. [181] observed a large friction reduction of 6% with a trace amount of CNT (about 0.03%), which is dispersed steadily in oil compared with high concentration of CNTs.

Apart from that, other carbon-based materials also exhibited superior tribological properties, such as fullerene-like carbons [182], carbon fibers [183], and carbon-coating coatings [184]. Liu et al. [182] found that the lower hydrogen concentration in the preparation process of fullerene-like carbons was beneficial to enter the low friction state. Wang et al. [183] indicated that non-smooth carbon-fiber-reinforced polyetheretherketone (CFRPEEK) surfaces can produce hydrodynamic lubrication effect and perform better seawater lubrication properties. The excellent lubricating properties of the prepared carbon-containing coatings, which were not limited to specific working conditions [185], originated from the rearrangement of the carbon structures and stability of adsorbed films [147].

2.4.2 *Transition metal dichalcogenides (TMDs)*

Among the various members of the TMD family of compounds, molybdenum disulfide($MoS_2$) and tungsten disulfide ($WS_2$) both have a similar layered structure to graphene, and their weak interlaminar shear forces make them an excellent lubricant additive. Fu et al. [186] modified $WS_2$ using silane encapsulated in the dimples fabricated on the surface of cylinder liner samples and reached a conclusion that the anti-friction and sustained mechanisms can be explained by the synergistic effect of dimples, solid lubricant, and silane coupling agent. Ru et al. [187] investigated the relationship between the torsion angle and the tribological properties of bilayer graphene and $MoS_2$/$MoSe_2$ van der Waals heterogeneous structures by molecular dynamics simulations. The results show that for the twisted bilayer graphene, the friction is significantly reduced and the super-lubricity ($\mu \leq 0.001$) occurs during the uneven stacking process.

Many efforts have been made to reduce friction and wear using TMDs. Yadav et al. [188] combined AFM and density functional theory (DFT) to identify the interfacial interaction and established a correlation between tribological behavior, interfacial charge distribution, and variations in the potential energy profile with sliding along the metal/2D-materials interfaces. Arif et al. [189] believed that while water increases the friction and adhesion at the steel/ultra-thin graphite interface, an opposite trend is observed for the steel/molybdenum disulfide interface. Water acts as a temporary protective film to inhibit chemical reactions. Liu et al. [190] found that by introducing the loose $MoS_2$ powder in the gap of a point contact friction pair, the friction and wear rate between the friction pair of the GCr15 ball and Q235 disk were reduced by one-fifth compared with the values without powder lubrication. He et al. [191] prepared a 350–400 nm-thick $MoS_2$ film with nano-flowers structure on porous anodic aluminum oxide (AAO)/Al substrate by magnetron sputtering. Significant improvement in the lubrication performance at low friction coefficient (0.15) was realized compared with $MoS_2$/Al structure, and the wear rate decreased by ~98%. Fleming [192] did a study of physical vapor deposition (PVD) coatings, suspensions, and an ashless phosphate ester in oil at the interface of the upper die-workpiece and found that the mixing of $MoS_2$ micro-platelets and an ashless phosphate ester in oil showed tribological synergistic effect, which provided higher quality surface finish and greater protection against wear. Slapnik et al. [193] improved tribological performance of the photopolymer-based material for additive manufacturing (AM) using vat photopolymerization (VPP) by modifying photocurable acrylates with different solid lubricants, PTFE, graphite, and $MoS_2$. Lu et al. [194] prepared a kind of self-lubricating composites that can adapt to different environments by adding reduced graphene oxide(RGO) and $WS_2$ into the Cu matrix. The tribological behaviors were obtained to provide a theoretical basis for preparing Cu-based self-lubricating composites that can adapt to different environments. Nautiyal et al. [195] demonstrated nanostructured $MoS_2$ grown on reduced graphene oxide (rGO–$MoS_2$) as a lubricating reinforcement material for copper matrix composite in dry sliding. The high mechanical strength and low shearing properties driven by the lamellar structure of rGO–$MoS_2$ furnished the self-lubricating properties to Cu–rGO–$MoS_2$ nanocomposites.

#### 2.4.3 Polymers and material modification

Among polymeric materials, PTFE is well known for its anti-friction property. It is a commonly used lubricating material and many researchers have done works about its preparation and modification. Without sacrificing the characteristic low friction of PTFE, its wear resistance can be improved by adding fillers to the PTFE matrix. Zuo et al. [196] effectively improved the abrasion resistance of polyethersulfone (PES)/PTFE-based polymers by using lamellar sodium-montmorillonite (NaMMT) as reinforcing filler, due to the load bearing of NaMMT on the worn surface. Levin et al. [197] successfully enhanced the friction and wear reduction properties of $WS_2$- inorganic nanotubes (INTs) with the addition of PTFE nanospheres using bath sonication. Abe et al. [198] found that by incorporating proper amount $SiO_2$ nanoparticle dispersion into the polydopamine (PDA) underlayer, the durability of the PDA/PTFE coating can be improved by 70%, and this effect was attributed to the increased adhesion of the PTFE coating to the PDA underlayer. Chen et al. [199] prepared a composite coating of PTFE filled with silane coupling agent modified graphene oxide (mGO/PTFE) on textured stainless steel (SS) substrate. The groove texture processed by laser surface texturing technology greatly improved the load-carrying capacity and anti-wear life of the PTFE coatings. The wear scar width and the friction coefficient are significantly reduced, as the PTFE coating was filled by mGO. Liang et al. [200] employed fluorinated graphene (FG) with various fluorine/carbon (F/C) ratios and graphene (G) as additions incorporating into the PTFE matrix. The performances of self-lubricity and wear resistance for four fabricated FG/PTFE composites were superior to those of G/PTFE composite.

With the progress of modern science and technology, in order to solve the mechanical lubrication problems under special working conditions such as high load, high vacuum, high and low temperatures, strong radiation, and strong corrosion, solid lubrication materials have been developed from a single powder, film into composite materials composed of a variety of components. The research on the mechanism of action and application of these materials has also been developed rapidly, and many new processes and technologies for the preparation and application of these materials have emerged. He et al. [201] prepared novel composites of ultrahigh molecular weight polyethylene (UHMWPE) by adding multi-layer directional structure polyester fibers (PETF) with different orientations. The PETF can improve the ultimate flexural strength and reduce friction coefficient of the composites. The oriented fibers could further enhance the composites and solve the high wear volume problem after the addition of PETF. Hausberger et al. [202] studied the tribological properties of PEEK and PPA-based compounds and the influence of alternative metal sulfide solid lubricants in combination with different glass fiber contents. They found the beneficial effect of adding metal sulfide filler systems to different matrices on tribological performance, in which the matrix-filler-bonding has a crucial impact. Tadokoro et al. [203] developed a homemade apparatus modelling reciprocating seals consisted of an inner steel rod. The concentrated polymer brushes (CPB) film narrowed the clearance between the two steel surfaces, which improved its sealing performance, which provides another avenue for green technologies toward reciprocating seals with ultralow leakage and ultralow friction. Ebersbach et al. [204] studied the tribological behavior of surfaces obtained by turning in sintered self-lubricating composites. The turned surfaces did not present friction coefficient values within the lubricity limit ($m < 0.2$). The friction coefficient and the wear rate of the turned surfaces were around 320% and 40% higher, respectively, than those of the sintered surfaces.

### 2.5 Other lubrication

In addition to the classic liquid and solid lubrication, there are some special and new forms of lubrication. The state of grease lubrication and liquid metal is between liquid and solid, while gas lubrication is outside of liquid-solid state. In addition, there are surface textures using fluid effects, self-lubricating materials without additional lubricants, tribofilm formed in boundary, and high-temperature lubrication under special operating conditions.

*2.5.1 Liquid metal*

Liquid metal is a low melting point, amorphous, flowing metal. It can be liquid at room temperature, so that it has good liquidity, thermal conductivity, electrical conductivity, and thermal stability. The most common liquid metals are mercury and gallium, but mercury's highly toxic and volatile nature limits its use. Unlike mercury, gallium is non-toxic and recyclable. Gallium has a melting point of 29.8 °C, slightly higher than room temperature, which can be reduced by alloying.

Bai et al. [205] found that the friction coefficient and wear rate of steel lubricated with gallium-based liquid metal (GBLM) in aerobic environment are lower than that in anaerobic environment. In air, wear rates and COFs increase with humidity. In the aerobic environment, GBLM gradually changes from liquid to paste due to the formation of GBLM oxidation, and GBLM has strong adhesion on the metal surface (Fig. 14).

Li et al. [206, 207] proposed fabricating liquid metal by adding water-based lubricant additive with good dispersion stability and chemical stability or hexagonal boron nitride (h-BN) nanosheets to reduce the COF and wear rate. It can be attributed to the formation of gallium-contained tribofilms, the high heat dissipation efficiency of additives.

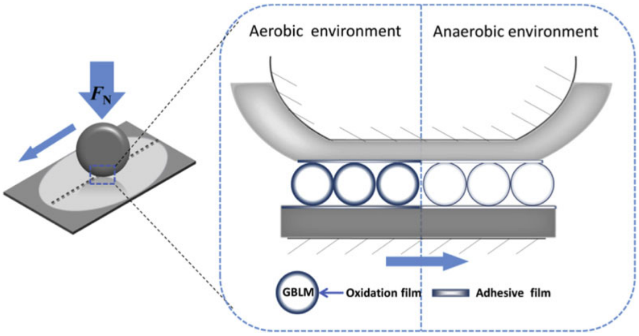

**Fig. 14** Schematic of the proposed lubrication model of steel with GBLM lubrication in aerobic and anaerobic environments [205]. Reproduced with permission from Ref. [205], © Elsevier Ltd., 2020.

*2.5.2 Gas lubrication*

Hydrostatic-dynamic gas lubrication can realize the non-contact operation of the end face of friction pair, and it has the excellent characteristics of small speed limit of equipment, strong stability of gas film, and high bearing capacity, and has been applied more and more in the fields of sealing, bearing, and so on.

Considering a more complete study of the influence of temperature and thermodynamic gas properties in the contact, Bouchehit et al. [208] found that it is possible to use surrounding gases as a lubricant and they have studied the behavior of different gases in static and linear dynamic conditions of a lobed bearing, coupled with nonlinear phenomena, as shown in Fig. 15.

*2.5.3 Surface texture*

Surface texture technology is called the technique of accurately producing various surface geometry on relatively smooth surfaces. This type of surface texture usually has a regular distribution, usually small depressions, or bumps. However, it can also be designed as grooves in some special environments.

With investigations about the influence of different surface morphologies on friction performance, significant decrease in COF and wear ratio has been found in the textured sample compared with smooth sample, especially in starved hydrodynamic lubrication [209–214]. The comparison of the variations in the friction coefficient among the three types of discs at different sliding speeds was shown in Fig. 16 [214].

The simulation work of Wang et al. [215] revealed that the loading capacity can be enhanced by the effects of turbulence and blocking.

*2.5.4 Self-lubricating materials*

Self-lubricating materials refer to those solid materials with good tribological performance, which can be used for reducing friction and wear between two interacting surfaces in relative motion. Self-lubricating materials are generally used in a wide range of operating temperature and special environmental conditions (such as radioactive, high vacuum environment, and products shall not be contaminated or there can be no lubricating liquid), including oil-loaded microcapsules, solid powder lubricant,

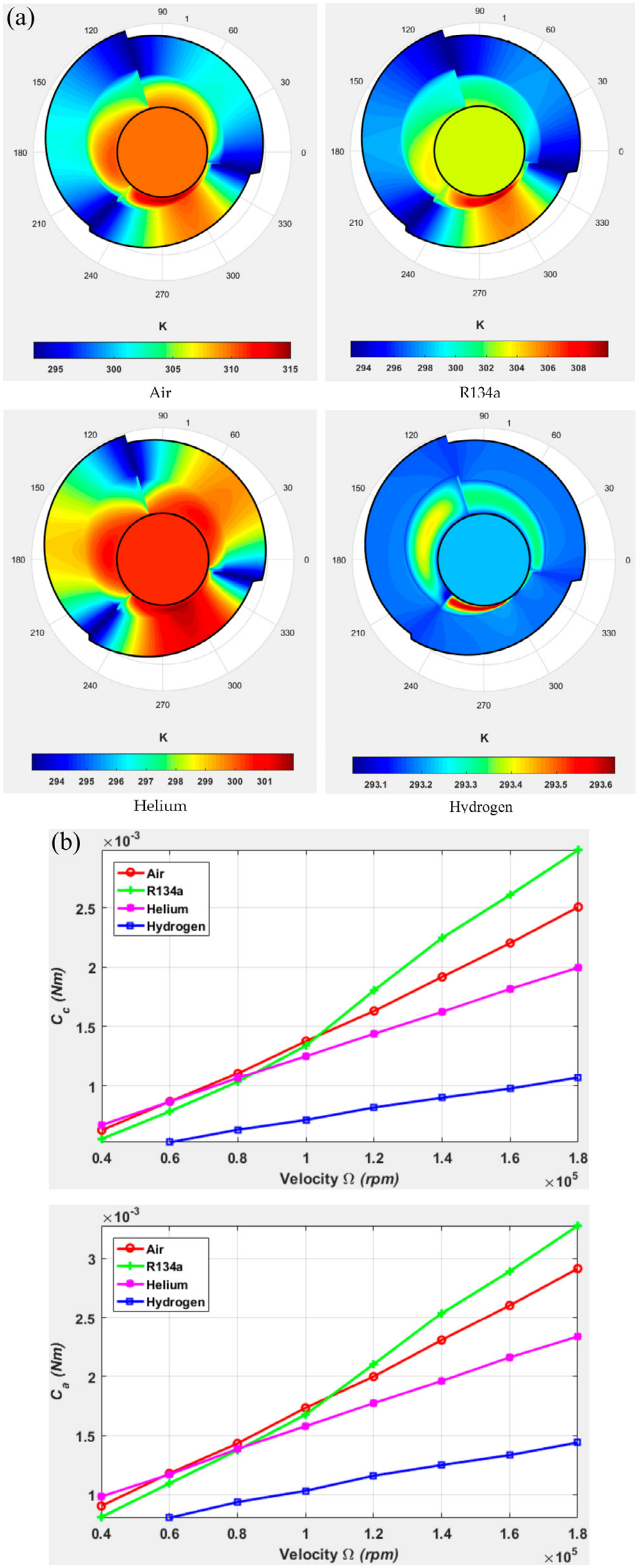

**Fig. 15** (a) Temperature distribution in the solids (shaft and housing). (b) Housing frictional torque *Cc* and (c) shaft frictional torque *Ca* versus shaft speed, bearing load 10 N, preload $m$ = 0.2 [208]. Reproduced with permission from Ref. [208], © The authors, 2021.

solid self-lubricating film or coating, and composite materials.

Meng et al. [216] and Yin et al. [217] found the presence of the liquid lubricants significantly reduces the friction coefficient and the wear rate of the pure coating. Wang et al. [218] prepared ultralow friction polymer composites by adding oil-loaded microcapsules into epoxy (EP) resin. With the formation of a boundary lubricating film formed by released PAO oil from the microcapsules during the friction process, which could prevent the direct contact of two rubbing surfaces, they realized an extremely low friction coefficient and wear rate.

Vieira et al. [219] found that the role of the $MoS_2$ particles in prevention the formation of adhesion between metals can improve the tribological performance of the steel–bronze friction pair and the mixture of the best combination of satisfactory hardness, low friction coefficient and low wear rates was tested. Xiao et al. [220] found that the $MoS_2$ film coverage exhibited an exponential relationship with the volume fraction of the $MoS_2$, which verified that adding excess lubricant does not contribute to reduce the friction coefficient.

Miao et al. [221] investigated the lubricating behavior of NbN coating in a three-phase contact environment (water, air, and NbN). Oxidation and hydrolytic reactions of NbN lead to the formation of "colloidal solutions", which contains $Nb_2O_5$ colloidal particles in the friction pair. The low friction coefficient and wear of NbN can be achieved by the weak shear of the "colloidal solutions" and the double electric layer repulsion (see Fig. 17).

With improvement of related fabrication methods, the performance of self-lubricating coatings can also be greatly improved. Yan et al. [222] combined carrier transport and mechanical milling to fabricate nickel–aluminum (Ni–Al) based self-lubricating composite coating on aluminum (Al) alloy, which made the Ni–Al composite coating exhibit lower friction coefficient and wear rate due to the more uniformly distributed granular Ag enriched areas in the coating, which can effectively avoid the gliding fracture of the coating. Wang et al. [218] developed hard self-lubricating coatings for dry cutting and die casting using a cathodic multi-arc ion plating process, which can

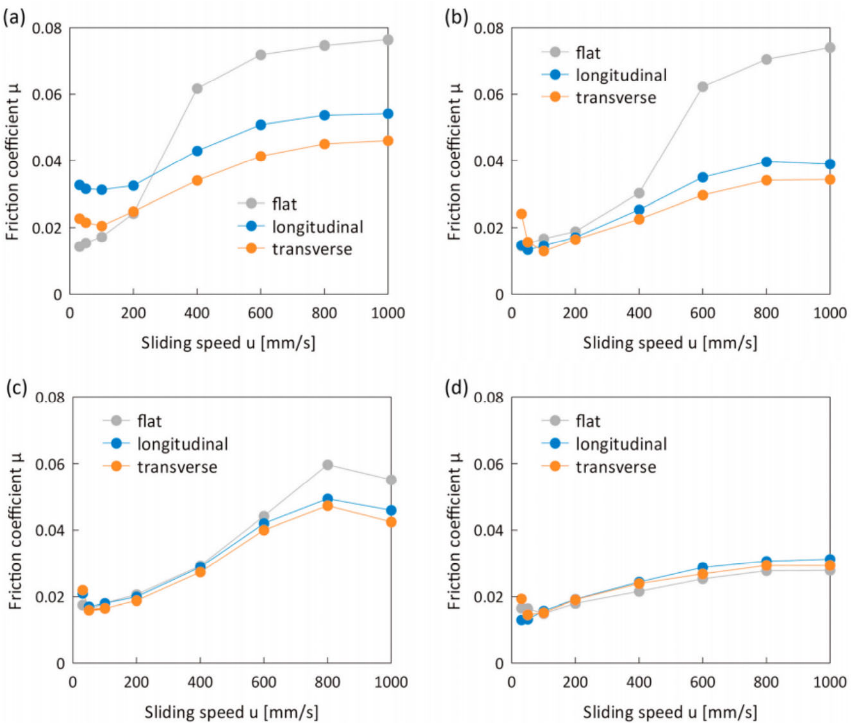

**Fig. 16** Comparison of the variations in the friction coefficient among the three types of discs at different sliding speeds for lubricant quantities of (a) 50 μL, (b) 100 μL, (c) 150 μL, and (d) fully flooded conditions [214]. Reproduced with permission from Ref. [214], © Elsevier Ltd., 2021.

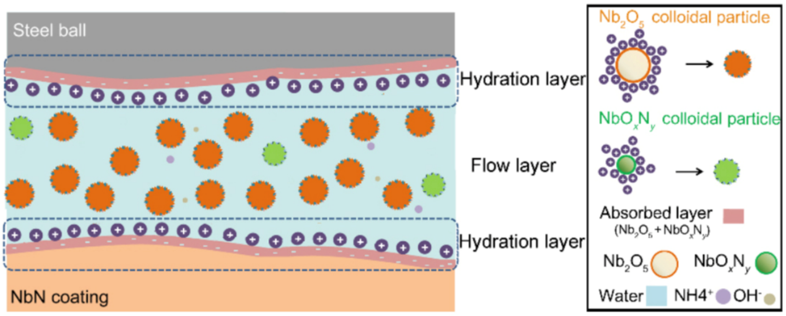

**Fig. 17** Schematic of the friction model of NbN in the three-phase contact environment [221]. Reproduced with permission from Ref. [221], © The author(s), 2022.

significantly improve the efficiency of characterizing multilayer nanostructures. Evaristo et al. [223] improved carbon-based coatings to reduce friction and wear in sliding contacts with direct current magnetron sputtering (DCMS). The as-prepared HiPIMS/DCMS thin films showed a low-order tungsten carbide structure, and the morphology was denser and can effectively avoid scratches.

Bashandeh et al. [224] reported on tribological performance of polymeric-based coatings, which showed excellent tribological performance with low friction coefficient and unmeasurable wear within a wide temperature range and constant contact pressure. Additives to common polymers matrices also help to improve friction performance. With investigation of Li et al. [225] on adding certain g-$C_3N_4$ to the PTFE composites and study of Chen et al. [226] about adding 0.3 wt% of g-$C_3N_4$/Ag to PPESK composites, the mechanisms of leading to better tribological performance were revealed, which are the stronger bonding of g-$C_3N_4$ derived from hydrogen bonds, self-lubricating effect of g-$C_3N_4$ nanosheet and Ag nanoparticles, the limited effect of g-$C_3N_4$/Ag on the shear deformation of PPESK film, and the formation of transfer film. Ren et al. [227] compared the heat resistance, and the tribological and mechanical properties of common polymer matrices, as well as the categories of functional fillers that improve the coating performance (Fig. 18).

### 2.5.5 *Tribofilm*

In the process of friction, a film is formed, usually in the form of a solid film on the friction surface. Its formation is a complex process, which depends on the friction conditions, and the properties of film forming materials and substrates. Tribofilm can effectively play the role of anti-wear, extreme pressure, and anti-abrasion [228]. Evolution of friction coefficient versus entrainment speed curves during rolling-sliding tests for solutions of four primary ZDDPs was shown in Fig. 19.

The formation of tribofilm can significantly decrease the COF and wear ratio. Bai et al. [229] found that under sulfur-containing boundary lubrication condition, the tribofilm layer formed on the worn surface of 15 wt% VN/7075 composite significantly decreased the friction coefficient compared with the Al7075 alloy. Zhao et al. [230] proposed that the excellent tribological properties under high temperature are attributed to the formation of physical adsorption

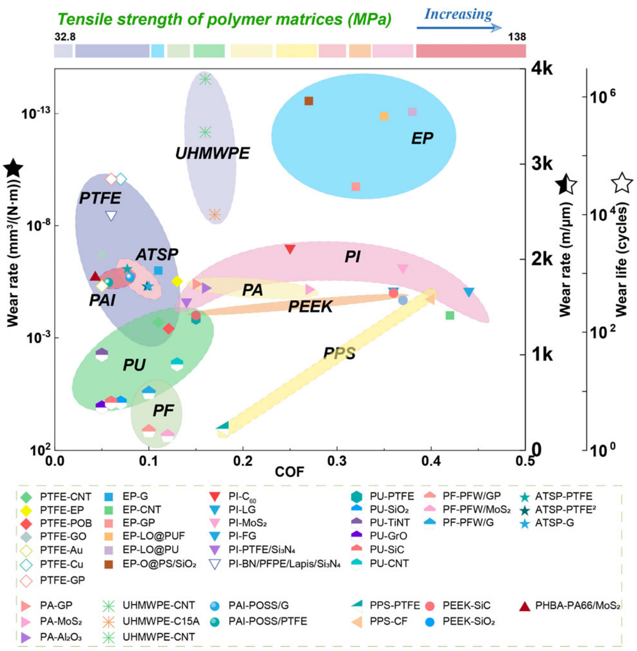

**Fig. 18** Comparison of the tribological properties of different polymer matrices and different fillers [227]. Reproduced with permission from Ref. [227], © The author(s), 2021.

films and boundary tribochemical films of ILs in diisopropylether (DIPE) esters under high temperature and high load. Yi et al. [231] attributed the acquisition of a stable macro hyperlubrication state with a COF of 0.002 to the *in situ* formation of tribofilm, which reduced shear stress and wear between surface concave-convex bodies, when they introduced $Ti_3C_2T_x$ MXene nanosheets into glycerol at $Si_3N_4$/sapphire interface.

Erck et.al. [232] proposed the strength of contact or bond during tribofilm formation can be considered to have a significant positive effect on the antiwear properties. Similarly, Zhang et al. [228] found the COF of tribofilms in the boundary friction is controlled by the adsorbing performance of alkyl structure for primary ZDDPs on contact surface. When the surface changed, such as ion-implantation in the steel substrate [233], or addition of some additives [234, 235], the formation of the tribofilm is affected, which in turn affects its tribological performance. Choudhury et al. [236] found that the adhesion of thin tribofilm to steel can be improved by rougher film, denser PTFE fiber network and enhanced transferred film with the mixing of Ag nanoparticles into polydopamine underlayer.

Zhao et al. [237] found that the polysiloxane/$MoS_2$ lubricating coatings has good wear resistance with the formation of smooth transfer film due to the synergistic effect of low surface energy polysiloxane and $MoS_2$. In addition, the polysiloxane coating has excellent atomic oxygen (AO) resistance due to the generation of $SiO_2$ protective layer, which can maintained low friction coefficient and good wear resistance under vacuum conditions. The form process of transfer film was shown in Fig. 20.

The addition of some additives to the base oil can also promote tribofilm formation. Yu et al. [238] studied the tribological behavior of lubricating oil added with natural attapulgite nanofibers and found the tribolayer with good lubricity and high hardness, and hardness/elastic modulus ratio is attributed to the tribological behavior of natural attapulgite nanofibes. Wang et al. [239] significantly reduced the friction coefficient and improved the wear resistance and extreme pressure performances of biodegradable base oils by synthesizing mercaptotriazine-containing organic molybdenum to form the tribofilm with a layered structure. It is consistent with the study of

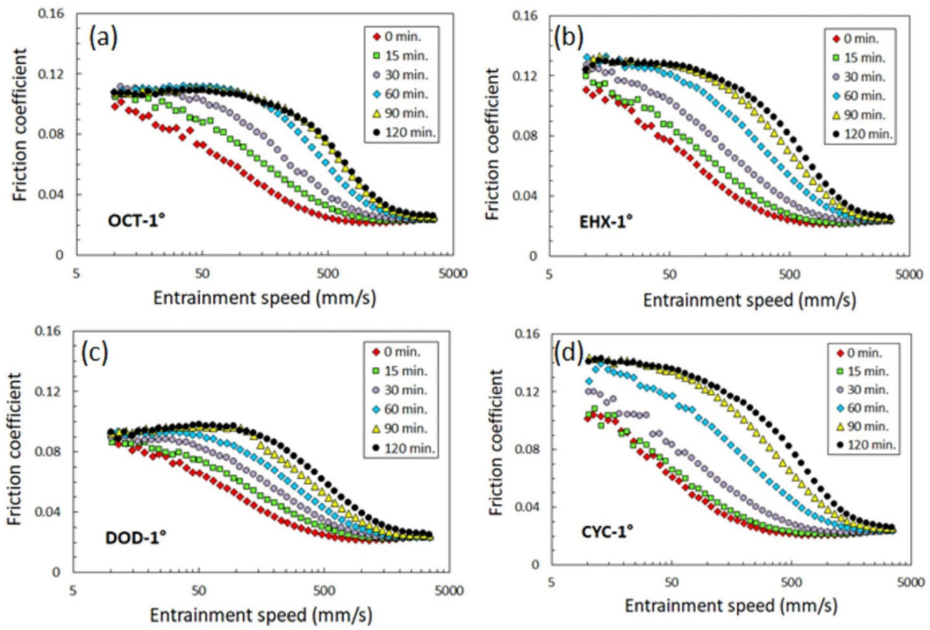

**Fig. 19** Evolution of friction coefficient versus entrainment speed curves during 2-h slow speed rolling-sliding tests for solutions of four primary ZDDPs. (a) Oct-1-yl ZDDP, (b) 2-ethylhexyl ZDDP, (c) dodec-1-yl ZDDP, and (d) cyclohexylethyl ZDDP [228]. Reproduced with permission from Ref. [228], © The author(s), 2021.

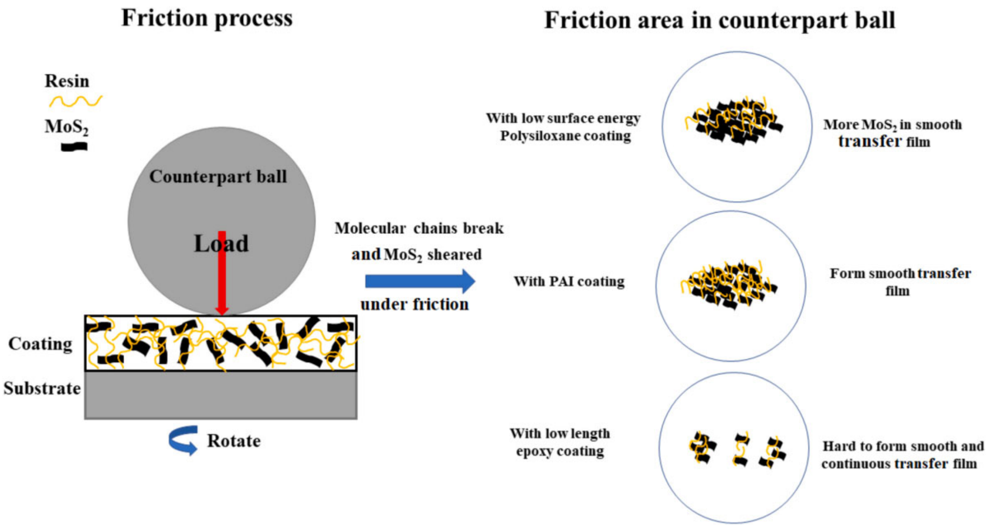

**Fig. 20** Form process of transfer film in counterpart ball corresponding to three different lubricating coatings [237]. Reproduced with permission from Ref. [237], © Elsevier Ltd., 2021.

Shaigan et al. [240], which indicated that the boundary film adsorbed for certain additives in ultra-low sulfur diesel (ULSD) fuels is multilayer.

What's more, the peak area of the $CF_2$ antisymmetric stretching vibrational absorption peak at 1,205 $cm^{-1}$ was found to be linearly correlated with transfer film thickness which follows the Lambert–Beer Law. Gu et al. [241] proposed a method of quantitatively characterizing the thickness of transfer films of PTFE/$\alpha$-alumina composites.

### 2.5.6 *High/low temperature lubrication*

In order to lay the foundation for the scientific development of aerospace engineering and industry application, it is necessary to reveal the nature of law between the friction pair surface structure evolution, lubrication performance, lubrication and friction pair damage failure behavior caused by the extreme temperature environment, such as high temperature, low temperature, alternating temperature, and high-speed overloading, and establish the design and evaluation method of lubrication and high wear resistant materials.

Yang et al. [242] studied the effects of temperature changes, rapid load and speed changes on the COF and lubricant breakdown phenomenon. COF evolution at the elevated temperature of 300 °C was demonstrated as shown in Fig. 21. Results showed three distinct stages, indicating the transformation from boundary lubrication condition to dry sliding condition, which reveals that the increase of temperature, contact load, and sliding speed caused an earlier breakdown of lubricant.

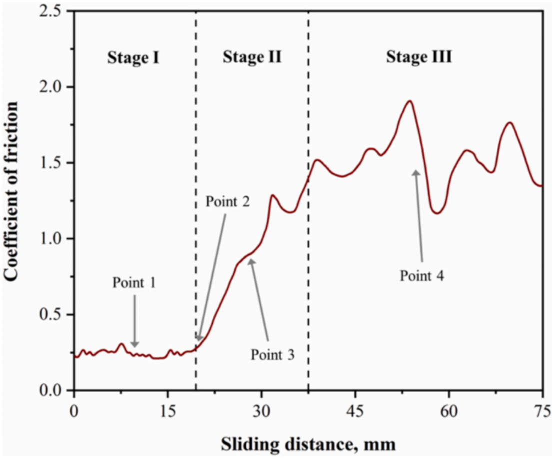

**Fig. 21** COF evolution at the elevated temperature of 300 ℃ with the speed of 50 mm/s and contact load of 5 N [242]. Reproduced with permission from Ref. [242], © Elsevier Ltd., 2021.

## 2.6 Application of lubrication

The essential function of any lubricant is to prolong the life and increase the efficiency of mechanical devices by reducing friction and wear. In this part, we reviewed applications of lubrication on biology, machinery fabrication, space, and marine environment.

### 2.6.1 *Biotribology*

Biological tribology is a new discipline of organization tribology, material science, biology, and medicine. In-depth study of biological tribology is an important requirement for science and technology to benefit mankind. Rebenda et al. [243] investigated the COF of five commercially available viscosupplements and their mixtures with synovial fluid (Fig. 22) to explore the effect of viscosupplementation on the friction of articular cartilage depending on the rheology of viscosupplements. Results showed that mixtures of synovial fluid and viscosupplement showed similar friction as clear viscosupplements.

Lin et al. [244] developed poly-L-lysine (PLL) and hyaluronan (HA) lubricious coating by the method of layer by layer to maintain low friction and prevent wear of endothelial glycocalyx layer (EGL). At the same time, Wang et al. [245] improved the compressive stress by almost double and considerably lowered COF to approximately 0.011 by introducing 2-methacryloyloxyethyl phosphorylcholine (MPC) polymers into the sulfobetaine methacrylate (SBMA) hydrogel under water lubrication.

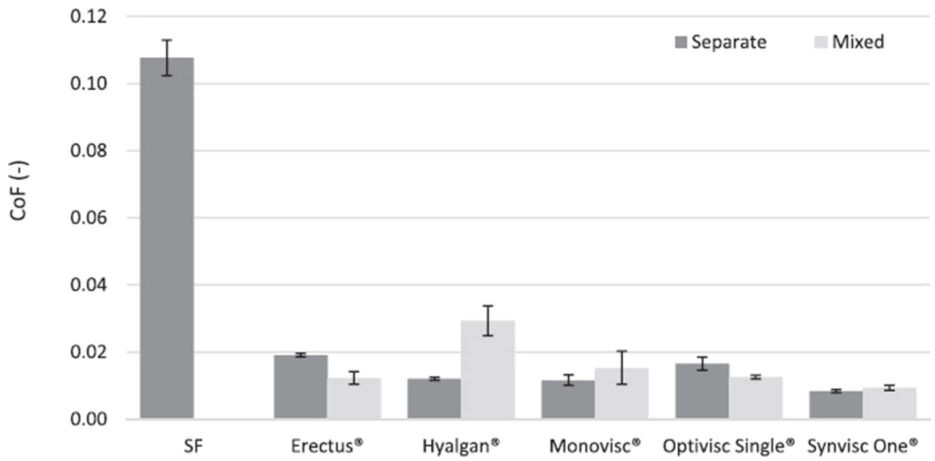

**Fig. 22** COF at the end of measurements for all tested lubricants [243]. Reproduced with permission from Ref. [243], © Elsevier Ltd., 2021.

*2.6.2 Lubrication of machinery manufacturing*

Tribology is one of the research fields of mechanical engineering. It is the theory and application of the study of friction, lubrication, and wear between surfaces acting on relative motion, as well as the relationship among the three. Tribological problems of mechanical manufacturing processes, such as metal forming, cutting, and ultra-finishing, are facing great challenges and need further research.

Duc et al. [246] studied the effects of nanoparticles used as additive in nanofluid on cutting performance and surface roughness in the minimum quantity cooling lubrication (MQCL) technique. They found the outstanding lubrication and cooling effects of $Al_2O_3/MoS_2$ hybrid nanofluid with the evaluation of different nanoparticle concentrations, cutting speeds, and feed rates. Bertolini et al. [247] used graphene nanoplatelets (GNP) as an additive to prepare nanofluid (NF) to limit the temperature increase in the cleavage region of Inconel 718. Wu et al. [248] found that compared with the traditional cold rolling oil, the friction coefficient of the microemulsion during cold-rolling experiment was reduced, and the finished surface roughness had been decreased, and the anticorrosion property of the copper surface had been improved. Sethupathi and Chandradass [249] compared the influence of different kinds of solid lubricants, namely, non-asbestos brake pad with sulfide mix (NASM), non-asbestos brake pad with bismuth sulfide (NABS), and non-asbestos brake pad with molybdenum disulfide (NAMO), on the friction stability of a non-asbestos disc brake pad, and NASM was excellent in terms of fade as well as wear resistance. Gupta et al. [121] utilized CVD coated carbide insert for turning Ti–6Al–4V alloy with sustainable hybrid cryogenic minimum quantity lubrication (MQL) cooling/lubrication techniques viz. Ranque–Hilsch Vortex tube plus MQL (RHVT + MQL), liquid nitrogen plus minimum quantity lubrication ($N_2$+MQL), and liquid nitrogen ($N_2$), and tribological aspects were evaluated in terms of tool wear, surface roughness, micro hardness, specific cutting energy, and chip observations, as shown in Fig. 23. Shah et al. [250] presented the comparison of a newly developed ultra-high voltage electrostatic minimum quantity lubrication (EMQL) using a customized nozzle with the MQL technique as an alternative cooling/lubricating method in turning processes of 15-5 precipitated hardened stainless steel (PHSS). Karagöz et al. [251] studied the friction and wear characteristics of graphene and graphene coating deposited by CVD process on Honda GX270 engine (nodular cast iron) piston rings. These features were investigated experimentally under boundary lubricated conditions.

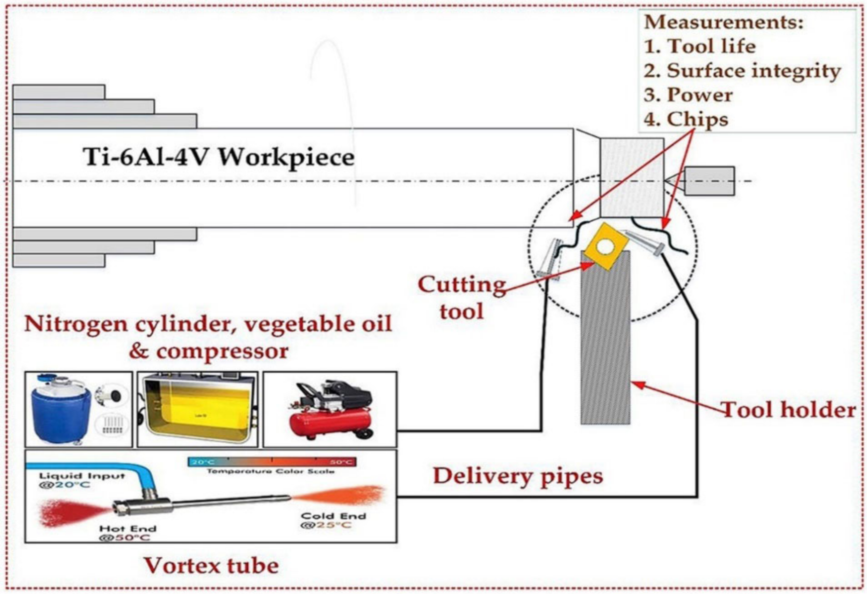

**Fig. 23** Mechanism of the performance of hybrid cryo-lubrications in the turning of Ti–6Al–4V alloy in terms of tool wear, surface roughness, and micro hardness [121]. Reproduced with permission from Ref. [121], © Elsevier Ltd., 2021.

*2.6.3 Lubrication in space or marine*

Due to the universality of tribology problems in marine machinery and the increasing attention to material and energy conservation, the research of tribology in marine machinery has been developed greatly. The tribology problem in marine machinery mainly exists in marine power plant and some auxiliary machinery.

Zhang et al. [252] established a turbulent flow lubrication model for the spiral-groove thrust bearing (SGTB) based on gas-liquid two phase flow to study the static and dynamic characteristics of high-speed water-lubricated SGTB, which was measured by an experiment setup in Fig. 24. It is pointed out that cavitating and centrifugal effects should be considered in turbulent flow conditions. Zhang et al. [253] used the compatibility of dimethyl silicone oil (PMX-200) and polydimethylsiloxane (PDMS) to prepare lubricant-infused surface (LIS). The surface silicone oil of the cured polymer LIS has self-replenishing properties, that is, when the silicone oil film on the surface decreases or disappears, the silicone oil film inside the PDMS polymer will quickly and automatically diffuse to the surface to form a new silicone oil film, which makes the LIS. It has a small water-slip angle and high lubricating ability, and is expected to be widely used in anti-icing and other occasions. Xiao et al. [254] pointed that the surface of water-lubricated rubber bearings has a more alternative friction coefficient and wear rate under seawater than in other lubrication conditions. The seawater not only acts as a lubricating medium but also brings microstructure while corroding the rubber interface, thereby further enhancing the lubricating effect and storing abrasive debris.

## 3 Wear and surface engineering

### 3.1 Introduction of wear and surface engineering

The review of the progresses in researches on wear and surface engineering is organized in the following 5 parts. Studies on the major wear mechanisms are overviewed in the first part, paying special attention on the relationship between wear rate and wear debris formation with the evolution of microstructures of subsurfaces of rubbing bodies under various working conditions. In the second part, progresses in physical and digital modelling, monitoring, and prediction and assessment of wear processes are reviewed. Papers on surface treatments and modifications are briefly described in the third part, followed by the published research work on additive surface engineering. In the last part, reviewing on a portion of papers on brake and friction materials is

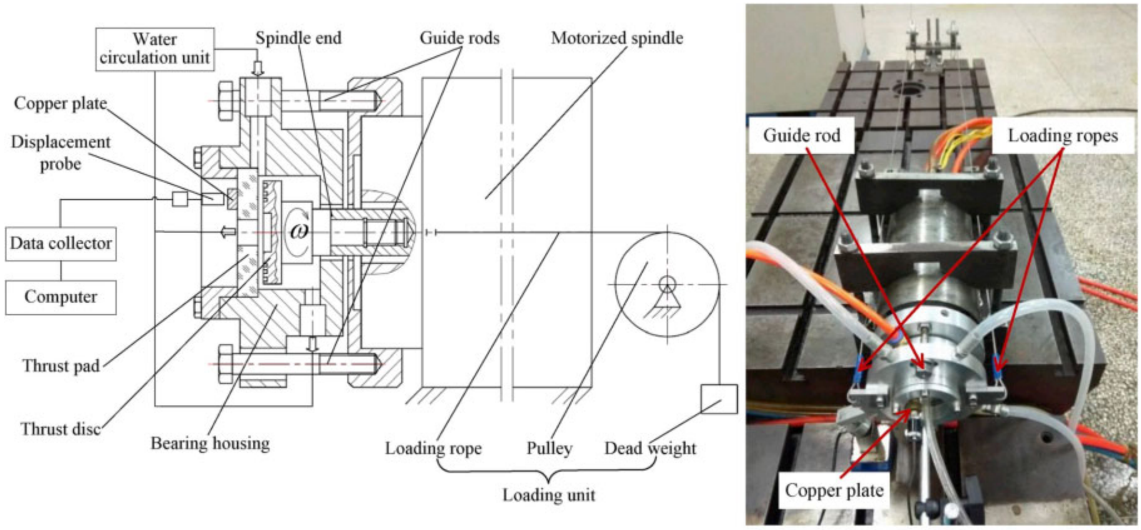

(a) Schematic view (b) Real scene image

**Fig. 24** Experiment setup to measure the characteristics of the spiral-groove thrust bearing (SGTB) [252]. Reproduced with permission from Ref. [252], © Elsevier Ltd., 2020.

presented. Total of 367 papers were selected from 530+ paper retrieved in the databases in preparation of this Section.

## 3.2 Wear mechanisms and relationships with material structures and working conditions

### *3.2.1 Catastrophic adhesive wear mechanisms*

In literatures, there are various different terms, including scuffing, scoring, seizure, galling, and gouging, to describe catastrophic failures frequently happened on tribo-contacts in a wide range of engineering applications. For lack of clear distinctions and thorough understanding of the failure mechanisms, different researchers might often use different terms to describe the same type of such failures, or different types of failures might be described by the same terms sometimes. Common features of these catastrophic wear phenomena are rapid and irreversible development, adhesion-initiated and severe selective material transfer, and hence wear rate or wear coefficient, the parameters used for characterization of steady wear, are no longer meaningful in such a case. Experimental and theoretical investigations into mechanisms of catastrophic adhesive wear have been doing for many decades, and some progresses and findings have been published in the past two years.

Rogers et al. [255] performed galling tests of 316L stainless steel (SS) at ambient condition on an ASTM G196 standard tester. The purpose of the experiments was to clarify the effect of the oxide layer pre-formed on the SS sample surfaces by exposure in simulated pressurized water reactor water for 850 h. They found that the degree of galling, quantified by surface roughening parameters, was suppressed remarkably by the pre-formed oxide layer due to the weaker adhesion than the non-oxidized samples. The subsurface microstructure of the tested SS samples changed greatly, and a thin nanocrystalline layer with transformed martensite was induced by severe plastic deformation undergone during the tests. Yagi et al. [256] constructed an *in-situ* test rig (Fig. 25), which combined a synchrotron X-ray diffraction detector with a visible camera and a near-infrared thermometer, to observe the changes in crystal grain orientation, surface temperature, heated area, as well as friction coefficient. They successfully captured the crystal grain orientation inclination under shear, phase transformation from martensite to austenite and about 1,000 °C temperature rise simultaneously during the scuffing of a tribo-contact of a stationary crowned steel pin against a rotating quartz glass disc. A rotating ball-on-disc tribometer was used to investigate the effectiveness of a top soft transition metal dichalcogenide (TMD) film deposited on a hard TiCN sublayer for enhancing scuffing resistance under starved lubrication conditions [257]. Their experiment results indicated that the elapsed time between the scuff initiation and the oil supply removal was extended by the application of the softer lubricious TMD film on the hard sublayer. The goal of the study was to extend the survival time of helicopter gears subjected to cut-off of lubricating oil supply. The scuffing of gears under rolling and sliding speed up to 40 m/s was also simulated by using a high speed two-disc tribometer [258], which correlated the occurrence of scuffing with abrupt increases in disc surface temperature and friction coefficient. Scuffing experiments on metallic powder

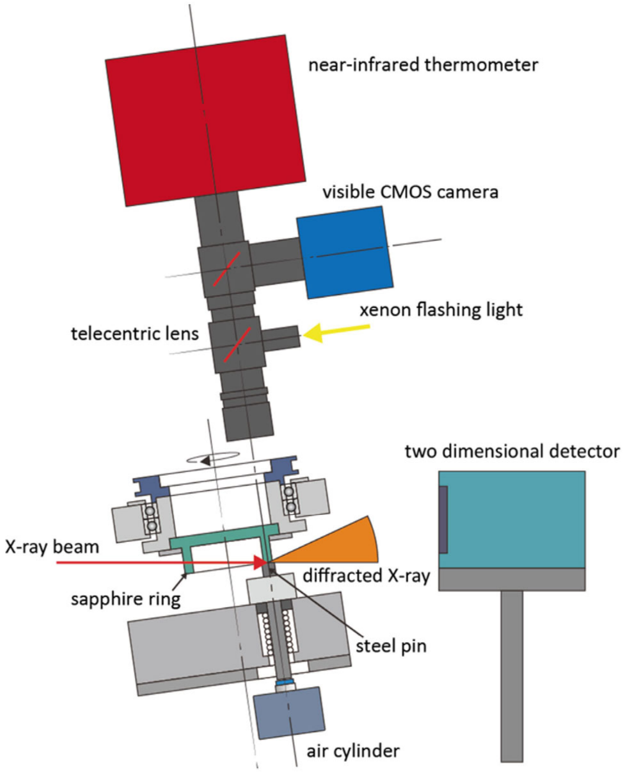

**Fig. 25** A multidimensional *in-situ* observation system [256]. Reproduced with permission from Ref. [256], © Springer Science+Business Media, LLC, part of Springer Nature, 2020.

journal bearings lubricated with two kinds of lubricant, PAO and ester, were done in a wide range of material-lubricant combinations. To obtain statistically meaningful test results with a shorter testing time, a 5-bench bearing tester was developed, which has the functions of monitoring the variations of friction coefficient and temperature rise under various prescribed loading and rotating speed cycles [259].

Besides of the above experimental studies, numerical modelling of local thermal damage and adhesive wear was also conducted. Morales-Espejel and Brizmer [260] simulated the surface fatigue, mild wear, and thermal damage for ball bearings under high speed and high load conditions. They regarded the scuffing at the ball–race contact as a thermal creep process and incorporated a modified thermal creep model with the mixed thermo-elastohydrodynamic lubrication analysis and the Archard wear equation. Considering that adhesive wear always accompanies the occurrence of selective material transfer, which manifests fracture of material inside the subsurface layer instead of breaking of adhesive junctions. Cao et al. [261] proposed a fracture-induced adhesive wear criterion, as Eq. (1), for making judgement of whether and where adhesive wear happens in numerical simulation. They viewed the adhesive wear as a competition between external friction (no wear) and internal friction (fracture-induced material transfer and wear). The key factor $\theta$ is the ratio of the change in surface energy, $A_c W_{12}(x,y,t)$ due to the separation the adhered junction area to the increase in surface energy, $2A_w$ ($\gamma(x,y,t) - \alpha W_f(x,y)$), where $W_f = \int_0^t \mu_c p_c |u_2 - u_1| \mathrm{d}t$, the frictional work done till the moment *t*. Adhesive wear happens if $\theta \geq 1$.

$$\theta = \frac{A_c W_{12}(x,y,t)}{2\left[\gamma(x,y) - \int_0^t \alpha \mu_c p_c(x,y,t)|u_2 - u_1|\mathrm{d}t\right] A_\mathrm{w}} \quad (1)$$

here $A_c$ and $A_w$ represent the projection area and the crown surface area of the wear element respectively, $\gamma(x,y)$ and $W_{12}(x,y,t)$ denote the surface energy and work of adhesion per unit area at the position (*x*, *y*) at time *t*, and $\alpha$ refers as conversion coefficient, means how much of the frictional work dissipates as the surface energy degradation.

### 3.2.2 *Rolling contact fatigue (RCF) and white etching crack (WEC)*

RCF is another type of catastrophic failures encountered in concentrated contacts in motions of pure rolling or rolling and sliding, especially under heavy loads. Unlike seizure or scuffing failures, it is the fatigue property of materials that dominates the RCF rather than the surface properties such as work of adhesion and surface energy. Material microstructures, especially internal defects, hard inclusions, and surface integrity are the major inherent factors determining the RCF life, which rely mainly on the metallurgical and manufacturing processes. Lubrication is considered to be detrimental to RCF in contrast to the positive effect of adhesion resistant. Experimental and theoretical researches on RCF have been widely conducted in relation to the life of rolling element bearings, gears, and wheel–rail contacts under lubricated or dry operation condition. Ueda and Masuda [262] investigated the effects of carbon contents and hardness on RCF resistance in heavily-loaded pearlitic rail steels on a two-disc tribometer, and they concluded that increase in carbon content resulted in not only a higher hardness but also less spalls and shallower cracks. Hu et al. [263] compared the wear and RCF behaviors of bainitic (BH) and pearlitic (PH) rails by using a laboratory wheel–rail simulation tester under rolling-sliding conditions. Their experiment results indicated that the RCF damage of BH steel was more severe than that of PH steel and the wear and RCF damage of both PH and BH steels increased with the increase in creepages and pressures. Zeng et al. [264] used the same simulation tester to perform the RCF tests of wheel steels with different sizes of artificial surface dents, and they found that the critical defect dimension resulting in the RCF crack initiation of the pre-rolled wheel disc was 100–200 μm. RCF cracks initiated from the region near the middle edge of the defect, where the most significant stress concentration appears. Liu et al. [265] investigated the evolution of surface microstructure of D2 wheel steel during RCF by using the electron backscattering diffraction (EBSD) technique, and their inspections revealed that RCF cracks were initiated in the third stage, before which the misorientation of the proeutectoid ferrite increased from less than 2° to more than 10°, and dislocation density also increased gradually, and propagated primarily at the interface of pearlite/proeutectoid ferrite and in proeutectoid ferrite. Lorentz et al. [266] numerically simulated the RCF development process of non-conformal contacts like in rolling element bearings by using the finite element method (FEM). They adopted a continuum damage mechanics (CDM) model which has the same root of the thermal damage model in Ref. [260]. Their simulations predicted that refinement of grain size can improve the RCF resistance, which exhibits good corroboration with existing experimental results found in literature.

WEC, or white etching areas (WEAs), or white structure flaking (WSF) in other words, is a special type of RCF failure, often found in transmissions of electric motor power trains, especially controlled with a modern converter. WEC failures have a much shorter fatigue life compared with those of non-WEC fatigue failures, and have cross-sectional features which are distinctive from RCF [267].

The trigger of WEC is attributed to the stray current and shaft voltage existing on the transmissions in which rolling element bearings are installed. Many research results have shown that WEC correlates strongly with the hydrogen generation from the base oils and/or additives involved under frequent discharging. Influence of high electrical currents on WEC failures in a number of oil-lubricated cylindrical roller bearings and grease-lubricated ball bearings was investigated under different external voltage applications on a bearing tester [267]. It is found that current intensity, electrical polarity, and load type have strong influence on WEC formation while the effect of lubricant is relatively weak. The authors in Ref. [267] proposed an "energetic WEC fatigue" hypothesis, shown in Fig. 26, to explain their observations. Linzmayer et al. [268] tried to establish a WEC testing standard based on the FE8 wear life test of thrust roller bearings.

### 3.2.3 *Wheel–rail wear modes*

Along with the rapid expanding of high-speed railway networks, especially in the mainland of China in the past decade, researches on wheel–rail wear become more and more active in recent years. Apart from RCF, wheel–rail contacts experience progressive wear, which could suppress the growth rate of surface-initiated cracks. Two types of wear, unique about the wheel–rail rolling contacts, are rail corrugation and wheel polygonization, which have been attracting much attention because they degrade stability, comfortableness, and safety of railway vehicles. Liu and Wang [269] proposed a friction-induced torsional vibration model for interpretation of rail corrugation generation. They adopted a rate-dependent rolling friction law and combined it with wheel torsional dynamics to predict the rail corrugation amplitude and frequency. The predictions were compared with the field data obtained in Wuhan Metro Line 2, and reasonable agreement was achieved. In addition,

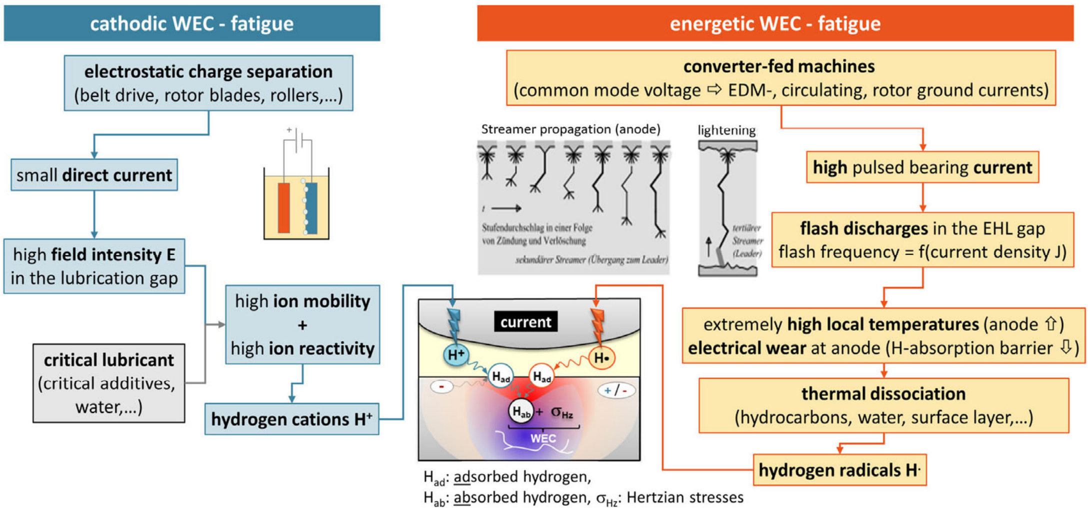

**Fig. 26** Root cause hypothesis for WEC formation due to bearing currents [267]. Reproduced with permission from Ref. [267], © Society of Tribologists and Lubrication Engineers, 2021.

their model can explain the field observations such as why corrugations mostly occur at curves of rail and why corrugations mostly occur on the top surface of inner rail. The model can couple with the vertical dynamics of wheel–rail contacts and a wheel wear model for a better simulation, as shown in Fig. 27. In the future, the rate-dependent rolling friction law can be refined further to be a rate-, state-, and temperature-dependent friction law, taking the effects of tread contamination [270], microstructure evolution [271], and thermal damage into account. On the other hand, Zhu et al. [272] used a friction-induced vibration model for wheel-track-disc brake system to predict the polygonal wear of wheel, combining with the complex eigenvalue analysis function of ABAQUS software. According to the analysis results, they attributed the wheel polygonal wear to the unstable vibration induced by the disc brake. They suggested that the effective damping ratio of unstable vibration decreases with increase in wheel diameter from 860 to 920 mm with an oblique web plate. In addition, the occurrence of polygonal wear increases with an increase in the tread wear depth, which was investigated by Faccoli et al. [273] with a small-scale two-disc test rig experimentally. The study by Zhang et al. [274] showed that ER8 wheel steel with a nonuniform microstructure composed of pearlite, proeutectoid ferrite, and upper bainite is less wear resistant than that with a uniform microstructure composed of pearlite and proeutectoid ferrite, because of the incompatible plastic deformation between the bainite and pearlite. In addition to the corrugation wear, the formation mechanism of squat-type defects on rails was analyzed by Steenbergen [275].

#### 3.2.4 *Cavitation erosion and corrosive wear*

Cavitation erosion is a type of accumulative surface damages caused by the impacting of bubbles and/or droplets on the solid body. It is found in a wide range of natural and industrial solid–liquid systems, and its mechanism is complicated owing to the ultrafast phase transformation processes and multiphysics interactions involved. When the liquid phase is corrosive to the solid, corrosive and/or electrochemical corrosive wear accompany with the cavitation erosion. Figures 28 and 29 depict the images of the cavitation

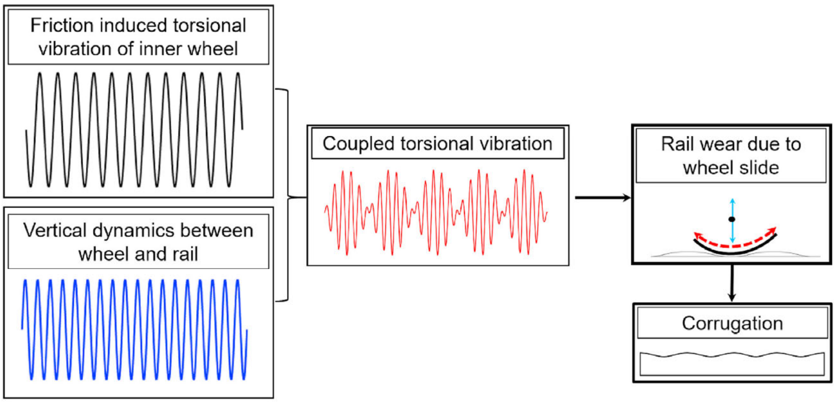

**Fig. 27** Full description of dynamic friction and wear of wheel–rail contacts [269]. Reproduced with permission from Ref. [269], © Elsevier B.V., 2021.

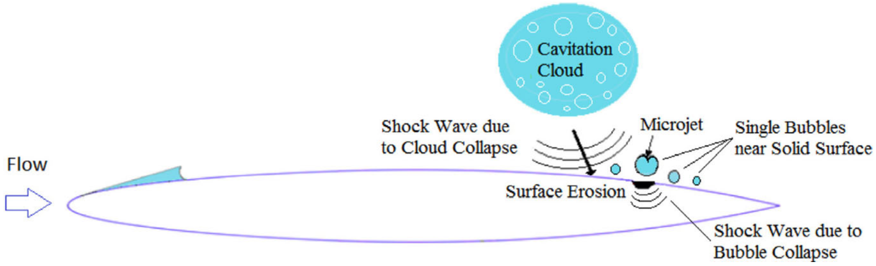

**Fig. 28** An image of cavitation erosion near the trailing edge of a hydrofoil due to cloud cavitation dynamics [276]. Reproduced with permission from Ref. [276], © Elsevier B.V., 2021.

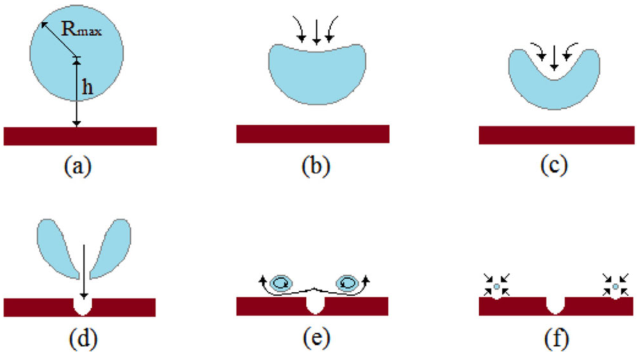

**Fig. 29** An image of the impacting actions of a single bubble on solid surface [276]. Reproduced with permission from Ref. [276], © Elsevier B.V., 2021.

erosion occurred near the trailing edge of a hydrofoil at macroscale and the impacting actions of a single bubble on solid surface at microscale respectively [276]. It should be noted that the impact pressure and flash temperature at the moment of bubble collapsing on solid surface are extremely high.

Kadivar et al. [276] used the laser-generated single bubble technique and a fast-imaging capture system to reveal the details of the dynamic process of a single cavitation bubble near a smooth surface or a textured surface with V-shaped microriblets. Their results showed that the bubble dynamics collapsing near the riblet surface differed significantly from that near the smooth one. The interaction of microbubbles formed between the bubble and the riblet surface during the first collapse mitigated the momentum of the microjet. The riblet structure reduced the cavitation erosion area on the rigid surface with the micro structured riblet. Cavitation erosion was found the major failure mechanism of a high chromium carbon steel impeller operating in phosphoric acid slurry [277], of the shoe-guide assembly in a high-pressure fuel pump [278], and of AISI 1018 steel elbows in potash brine-sand slurry [279]. X-ray diffraction study showed that the pristine microstructure of ~93 vol% $\gamma$ + ~7 vol% $\alpha'$ (austenite ($\gamma$), martensite ($\alpha'$, $\varepsilon$)) of the smooth solution-treated AISI 304 austenitic stainless steel samples experienced $\gamma \rightarrow \alpha'$ transformation fraction of ~89 vol% in the first 3 h of ASTM G32-10 ultrasonic-induced cavitation test without visible mass loss, and then started to loss the mass almost linearly with increase in testing time, keeping the austenite volume fraction of 11% constant [280]. The ASTM G32-92 cavitation erosion test was also used to evaluate the anti-erosion performance of chemical vacuum deposition (CVD) W/WC coatings, applied on Ti6Al4V alloy aircraft parts to prevent from water droplet erosion (WDE). It was found that a hierarchical coating microstructure demonstrated better performance compared to a monotonic columnar grain structure [281].

Most of metals and alloys suffer tribocorrosion or corrosive wear, in which rate of material loss is much larger than pure corrosion, in environment of reactive gaseous atmosphere, salty water, and corrosive chemicals, particularly at high temperature. To prevent metallic parts from corrosion and tribocorrosion, anti-corrosion and anti-wear coatings are often applied. $CuSn_5$–$Al_2O_3$ composite coatings were deposited on aluminum alloy 7075-T651 substrate by the low-pressure cold spraying technique. In Ref. [282], experiment results showed that the wear rate of the coatings decreased when rubbing against a 316L steel ball or a $Al_2O_3$ ball in artificial seawater on a ball-on-disc tester, and the wear mechanisms were abrasive and corrosive wear. The anti-tribocorrosion property of two cement coatings, WC–CoCr deposited by high velocity oxygen fuel (HVOF) and high velocity air fuel (HVAF), and $Cr_3C_2$–WC–NiCrCo applied by HVAF, was examined in paper machine environment on a pin-on-disc tribometer under the load of 20 N in electrolyte containing chlorides and sulphates (pH 4.5) [283]. It was reported that wear was the dominant degradation mechanism for all coatings, followed by corrosion-induced wear, the importance of which increased with rising potential. The overall material losses were the lowest for HVAF WC–CoCr and the highest for HVAF $Cr_3C_2$– WC–NiCrCo, the latter being related to the evident abrasive wear of the $Cr_3C_2$ phase and cracking along $Cr_3C_2$- matrix interface in the near-surface areas. Another widely-used technique to protect metallic parts from corrosion is to apply an anodic potential. Sun and Bailey [284] tried to apply a cathodic potential on a CoCrMo alloy, which is used in biomedical implants, in NaCl solution. They found that low friction and low wear were observed at potentials more cathodic than the hydrogen charging potential. The coefficient of friction and total material loss decreased with increasing cathodic potential, such that at −1,000 mV, extremely low coefficient of friction (COF) value of 0.02 and negligible material loss were obtained. Such reduction in friction and wear was attributed to the hydrogen charging and hydrogen segregated layer formation at the surface. Tribocorrosion is also a problem at fretting condition. Du et al. [285] conducted fretting wear tests of 22MnCrNiMo steel in pure water and artificial seawater using a pin-on-flat configuration at room temperature for 2 h. They reported that the COFs of 22MnCrNiMo friction pairs were in general smaller in seawater compared with that in pure water and wear volume loss of the steel in seawater was lower than that in pure water. There was a transformation of positive to negative interactions between corrosion and wear in the fretting corrosion and wear processes. The wear mechanism of 22MnCrNiMo steel was abrasive wear in pure water, whereas it was corrosion fatigue in seawater.

### 3.2.5 *Fretting wear and subsurface damages*

In recent years, publications on fretting wear have increased rapidly. A number of papers, including those focused on tribology transformed structures, have to be omitted here because of limited length of the review article. The selected papers are organized into three categories: Laboratory bench/simulation tests, standard tests, and mechanism analysis in the following review. To evaluate the cold welding in the so-called hold down and release mechanism (HDRMs) of spacecraft and satellites due to fretting wear during a launch and under various vacuum and lubrication conditions, Merstallinger et al. [286] developed a new fretting wear tester and a testing sequence, which can measure both adhesion force in cyclic vertical separations and static/kinetic friction in fretting, shown in Fig. 30. Through a series of simulation tests, they concluded that behavior in high vacuum is influenced by the "pre-life" on ground (fretting in ambient, e.g., when doing vibration tests), and replacement of grease in HDRMs could more likely cause failure. Some alloys did not show high adhesion forces in the high vacuum phase of a launch test. The fretting in air lead to tribo-corrosion, forming reaction layers that inhibit cold welding. Blades et al. [287] used a simulation tester for evaluation of fretting wear of nuclear fuel rods made of Zr–1Nb–1Sn-0.1Fe alloy, and they revealed that misalignment promoted premature wear because the intensification of fretting wear occurred by the appearance of complementary efforts, and wear residues under dry conditions remained adhered to the fretting surface. Lee et al. [288] used a grid-to-rod assembly to investigate the effect of $ZrO_2$ oxide layer of Zr-based fuel claddings on fretting wear of grid–rod contacts in normal pressurized water reactor (PWR) operation. Liu et al. [289] designed and used an experimental setup to investigate loosening behavior of threaded fasteners under cyclic shear displacement. Their experiment results showed that the looseness of threaded fasteners was small under high initial preload and low amplitude of shear displacement. For DLC-coated bolts, the thread damage was slight, exhibiting a better anti-loosening property comparing with zinc-coated bolts. The

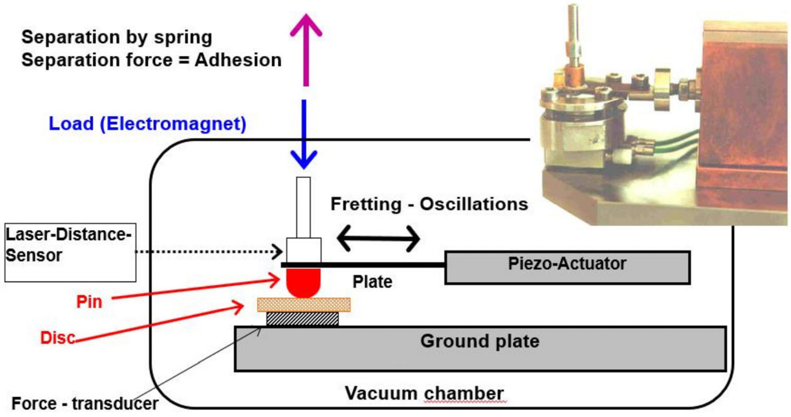

**Fig. 30** A fretting tester for simulating cold welding in HDRMs [286]. Reproduced with permission from Ref. [286], © The author(s), 2021.

blade-disk connecting structure (so-called tenon/mortise structure or dovetail attachment) in gas turbine engines is prone to fretting fatigue damage due to high contact stresses and small displacements on the blade/disk interfaces induced by varying centrifugal load and vibration at high temperature. A uniaxial fatigue test system equipped with a high temperature furnace was used to simulate the centrifugal load and cyclic load conditions of dovetail attachments made of DD6 nickel-based single crystal superalloys. Fretting test results showed that all of tested dovetail specimens failed at the contact leading edge area and along (111) [110] and (111) [110] slip systems, which were resulted from the large resolved shear stress at the contact edge [290]. A flat-on-flat annular contact test device was used to investigate the initial adhesive friction peak observed in dry self-mated quenched and tempered steel fretting contacts, and experiments under oil, grease, or paste lubrication showed that the adhesive peak could be eliminated effectively [291]. In Ref. [292], a special shaker-driven fretting tester was built to test the durability of electrical connectors under the combined stress of electric current, temperature, and high frequency lateral vibration. The experiment results showed that the service life of electrical contacts could be extended by a surface design approach that controls the metal interface and ensures low contact resistance. A strong relationship between surface roughness and electrical contact resistance was observed and surface texturing was identified as a viable option to increase durability of automotive electrical connectors by applying the theory of inventive problems solving (TRIZ).

Fretting wear is a mixed mode of adhesive, oxidative, abrasive, and fatigue wear. Shipway identified that with the increase of wear scar width wear rate transited from debris formation controlled mode to debris ejection controlled mode, and thus was time dependent [293]. Baêta et al. [294] also investigated the debris types and their influence on the rate of fretting wear of EN24-T/Ti–6Al–4V contacts in oxidative and non-oxidative atmospheres. Vast differences were observed in the effects of oxygen on the wear rates of the two materials. Xu et al. [295] studied on tribochemical and fatigue behavior of 316L stainless steel by using a torsional LFV100-T500-HH servo fatigue tester. By XPS analysis, they found that the extent of oxidation in the fretting damage zone was affected by the amplitude of relative displacement. The tribochemical reaction in the slip regime was more activated, and hence more severe wear than that in partial slip regime. More reports on oxidative wear and abrasive wear will be described later. On crack initiation in fretting, three critical plane fatigue parameters were combined with different stress averaging methods to estimate the crack initiation direction. It was found that the normal stress-based Smith–Watson–Topper parameter provided better crack orientation estimates than the shear stress-based parameters [296]. An interesting fretting wear model was proposed, which accounts random damage accumulation due to random excitation force [297].

#### 3.2.6 *Wear of polymer, rubber, and hydrogels contacting with similar or harder surfaces*

Adhesive friction and wear are prominent in the contacts of polymer, rubber, or hydrogel with similar or harder surfaces. These softer and elastic materials are widely used in seals, tyres, and light-load bearings, the life of which depends on the wear resistance of the contacts. Polytetrafluoroethylene (PTFE), polyimide, and polyvinyl chloride (PVC) or their composites, usually reinforced with hard particulate or fiber fillers for enhancement of wear resistance, are common engineering polymeric tribomaterials and hence have been mostly studied. Johansson et al. [298] investigated the effect of humidity and counterface material on the tribological behavior of carbon fiber reinforced PTFE composites in a dry nitrogen environment at different humidity levels and compared with that in laboratory air condition. Their experiment results indicated that the tribological performance of the PTFE composites were sensitive to environmental changes. The impact of humidity on both the coefficient of friction and specific wear rate was up to about 40%. To elucidate the mechanism of the ultralow wear (wear rate ~ $10^{-7}$ $mm^3/(N\cdot m)$) of PTFE filled with 5 wt% nano-alumina aggregates, Alam et al. [299] prepared the PTFE samples with different particle aggregate strengths and tested on a pin-on-flat reciprocating tribometer under a constant pressure of 6.3 MPa and sliding speed of 50 mm/s. Their experiments revealed that stable transfer film, induced by small debris, was responsible for the ultralow wear, and fillers directly reduced debris size even without a protective transfer film. Sun et al. [300] found that ultralow wear of PTFE composites could achieved only when 10 wt% nanosized (< 300 nm) $\alpha$-phase $Al_2O_3$ or micro-sized (> 3 μm) $\gamma$-phase $Al_2O_3$ particles were filled, whereas nanosized $\gamma$-phase (< 60 nm) or micro-sized $\alpha$-phase $Al_2O_3$ (1 μm) only mildly reduced PTFE wear rate to ~ 0.1–1× $10^{-4}$ $mm^3/(N \cdot m)$ at identical loading, dispersion, and testing conditions. The paradoxical filler size effect was explained by the authors as below. 1 μm $\alpha$-$Al_2O_3$ provided the least filler–matrix interface and largest unreinforced polymer domain in PTFE, which lead to the least crosslinked and compartmentalized tribofilms, whereas the 20 nm $\gamma$-$Al_2O_3$ provided the most filler–matrix interface which leads to excessive aluminum fluoride that blocked the filler–matrix chelation. In either case, tribofilms lacked enough crosslinking through filler–matrix chelation and became more susceptible to wear. When the softer polymer parts slide against a harder counterpart, not only the polymer itself but also the harder side wears off and becomes smoother, meaning that hard surfaces are polished by softer ones. The phenomenon has been noticed since 1960s, and several different hypotheses have been proposed to interpret the abnormal wear phenomena. Recently, it has been reported that contact forms between polymer parts and its harder counterpart also affects the wear behavior in addition to other effects. Six kinds of different polymer spheres, polystyrene (PS), polyoxymethylene (POM), polyamide 66 (PA 66), polypropylene (PP), high density polyethylene (HDPE), and PTFE, were rubbed on an inclined monocrystalline silicon plate in four different contact forms: (a) Sliding between a polymer sphere and a smooth silicon surface; (b) sliding between a polymer sphere and a rough silicon surface; (c) sliding between a polymer sphere and a smooth inclined silicon surface; and (d) rotation of a polymer sphere on a smooth inclined silicon surface. The contact form (c) showed the most severe wear comparing with other contact forms under same load conditions. The observed differences of the silicon surface under different contact modes were attributed to different stress states undergone. In addition to kinetic friction force, an additional static friction force, bending moment, or torque acted on the hard surface under certain contact forms. The polymer that produces layered wear debris can more effectively protect the hard surface from wear, compared to those produced granular debris [301]. The harder surface of the aerospace grade case-hardened sleeve was also worn off by 300 μm thick PTFE-lined radial lip in an oil seals tested for 72 h [302]. In Ref. [303], the tribological behaviors of polyimide composite against steel ball were studied under a motion compounded with rolling and sliding in comparison with pure sliding. The rolling-sliding motion has a strong influence on the tribological properties of polyimide composite depending on lubrication conditions. Especially under PAO4 oil lubrication, the rolling-sliding motion can facilitate the degradation of polyimide composite, resulting in severe surface wear and abundant black products forming. These results are meaningful for understanding the friction and wear mechanisms between steel balls and polyimide retainer in ball bearings.

Rubber is a kind of unique elastomers widely used in airplane and vehicle tyres, O-ring seals, footwear, etc. Three shapes of rubber samples were tested on the modified UMT tribometer under dry condition at speeds of 10 or 0.5 mm/s. Experiment results indicated that longer shapes (parallel to the slide direction) have lower kinetic friction coefficient $\mu_k$ due to frictional heating at velocities greater than 1 mm/s. A lesser but still significant frictional difference was also found at a velocity below 1 mm/s (0.5 mm/s). Investigating the wear rate and locations revealed that the leading edge length also has an effect, with longer front edged shapes producing the highest $\mu_k$ through additionally wearing. The behavior is definitely contrary to the classical Coulomb friction law [304]. The frictional heating leads thermal degradation of rubber and change in wear behavior during a long period of sliding. The tribological behavior of fluororubber (FKM) O-rings were traced by tribo-test and compared at different stages of the wear process [305]. Results showed that under the influence of thermal degradation of FKM, the wear rate and friction coefficient decreased, while the friction instability increased, associated with the wear tongue development. Rubber friction is also sensitive to the interfacial properties. By changing the lubricant of mixtures of water, ethanol, and glycerol, the influences of unforced dewetting and enforced wetting on real contact formation and friction behavior between a rubber hemisphere and a glass plate were analyzed during contacting and sliding processes. Unforced dewetting was quantified by the characteristic dewetting velocity that related to the spreading coefficient and lubricant viscosity, while enforced wetting was determined by the contacting and sliding velocities. The real contact area and friction coefficient correlated positively with the ratios of the characteristic dewetting velocity to the contacting and sliding velocities, respectively, depending on the time period [306]. A study of the dynamic contact between a rubber block and the abrasive surface at conditions close to those in a tire footprint was conducted [307]. The test samples were a miniaturized tread block allowing to reproduce the intermittent tire contact including correct contact timings, pressure, and friction coefficients in dependence on the driving maneuver to be tested. An analysis and comparison of friction and wear mechanisms for continuous contact and defined contact times were presented surpassing the characterization capabilities of DIN abrasion testers. In fluid machinery, abrasive particles included in the contaminated fluid cause abrasive wear of rubber seals, made of acrylonitrile–butadiene rubber (NBR). Effect of $Al_2O_3$ particle size on tribological behavior of rubber/steel seal pairs under contaminated water lubrication conditions was investigated experimentally. It was shown that the presence of abrasive particles dispersed in water deteriorated the tribological performance of the seal pairs. Two threshold values (approximately 7.5 and 75 μm) related to the "particle size effect" were obtained [308].

Hydrogels are chemically or physically cross-linked water absorbing networks of polymers, and its friction and wear behaviors depends on Young's modulus, porosity, and surface roughness strongly. A hydrogel hemisphere-on-hydrogel plate tribotool was used to investigate the friction characteristics of the hydrogel–hydrogel contacts [309]. A drop in friction coefficient is found when increasing the normal force, defining two distinct frictional regimes. The first frictional regime was sensitive to the nature and the length scale of the asperities on the hydrogel surface. The friction coefficient in the second regime appeared to be a material property. The decrease in friction coefficient was likely to be induced by the deformation of the soft surface asperities leading to a smoother surface. The potential of double network (DN) gel as a covering film for tribo-system applications was studied, focusing on the role of the DN gel film thickness on the friction property and the evolution of friction coefficient over distance [310]. Experiments showed that friction of DN gel film sliding against SiC in water can be classified into two types as "friction region I: friction remains substantially unchanged during the test" and "friction region II: friction decreases due to run-in". Friction region transition can be related to the Hersey number region, which consists of viscosity of lubricant, sliding speed, and apparent contact pressure. Hersey number larger than $1.5\times10^{-10}$ is a necessary condition for friction region II where different lubrication regimes can be found. 1.5 and 6 mm thick DN gel films had exhibited super low friction ($\mu$ < 0.01) under a wide range of friction conditions as friction region II, and especially 1.5 mm thick DN gel film, super low friction is achieved by twice run-in phenomena.

### *3.2.7 Material transfer and wear debris dynamics in wear process*

Material transfer and wear debris exist in friction and wear process not only in tribopairs of polymer and rubber, but also in most of tribosystems composed of any solids. They alter the interface structure and interaction partially and irreversibly and affect tribology performance greatly. When material transfer happens severely and abruptly, it would give rise to seizure, scuffing, scoring, or galling, which has been described in Section 3.2.1. If material transfer is selective, stable, and mild, it brings along an improved interface as seen in many of soft-on-hard tribopairs. A necessary condition for selective material transfer is that adhesion strength at the real junctions of a soft–hard contacts is stronger than the fracture strength of the softer body. The adhesion strength is usually characterized by the magnitude of work of adhesion for contacts between idea and perfect smooth surfaces. However, most of natural and engineering surfaces are rough, fractal rough in nature, and hence effective values of surface energy and work of adhesion are much deviated from the theoretical ones. Considering the inevitable contamination of surfaces in practice, evaluations of effective surface energy and work of adhesion are extremely difficult and often non-repeatable, especially for contacts with high stiffness. Based on the Persson and Tosatti's equation for effective surface energy of rough contact, which is applicable for rubber–rigid rough surface contacts, Ciavarella [311] proposed a new Persson–Tosatti stickness criterion and a new bearing area model (BAM) stickness criterion, and compared with the stickness criteria proposed by Violano as well as by Pastewka and Robbins previously. He concluded that the two new criteria and the Violano criterion show the same main factors for stickiness, which are the low wavevector cutoff of roughness, the rms amplitude of roughness and the ratio between the work of adhesion and the plane strain Young modulus, differing only by pre-factors or by a small difference in the power laws exponent. The stickness criterions state a necessary condition for re-separation of a rough contact elastically without internal fracture induced material transfer, while the adhesive wear criterion proposed by Cao et al. (see Eq. (1)) defines a necessary condition for adhesive and selective material transfer, if the parameters of $W_{12}$ and $\gamma$ are interpreted as the effective values. In heavy load conditions, the subsurface of contacting junctions undergoes plastic deformation, which results in alterations in orientation of crystalline due to slip of dislocations and roughening of surfaces, and thus affects the adhesion and wear behavior. Nakano et al. [312] studied the orientation dependence of the wear resistance in Co–Cr–Mo single crystal. They found that the wear resistance of the Co–Cr–Mo alloy with face-centered cubic (fcc) structure was higher on the planes in order of {110}, {001}, and {111}. The wear resistance on {110} is 1.5 times larger than that on {111}. The volume fraction of the hexagonal close-packed (hcp)-phase, which was formed as the strain-induced martensite during the wear test, was larger in the same order of the wear resistance. The variation in formation frequency of the hcp-phase during the wear test can be explained by focusing on the Schmid factor along the resultant direction of the applied stress and the friction stress, manifesting the stacking fault energy (SFE) effect on wear. Material transfer is found even in proximity contact in the head–disc interface of hard disc drives. Xiong et al. [313] investigated the material pick up on heat-assisted magnetic recording (HARM) heads experimentally, and identified at least two different types of smear and the critical temperatures for the lubricant smear formation and transfer from disc surface to head. It was shown that optimizing the media process, particularly the magnetic layer and lubricant, could help to reduce the smear.

In friction and wear process, a fraction of frictional work is dissipated not by heat, but by microstructural evolution, even phase transformation, and damage accumulation continuously or abruptly, within sublayers of the tribo-contacts. Dissipative mechanisms include highly-deformed layer (HDL) formation, deformation twinning, recrystallization, amorphization, and texturing. In some tribosystems, a portion of the frictional work is dissipated in third-body layers [314]. HDL thickness can vary from grain-to-grain, depending on crystallographic orientation relative to the sliding direction. Thus, the energy used in texturing can also vary grain-by-grain. Composition and surface processing methods affect the manner and degree of friction/wear correlation. Transitions in wear mechanisms were investigated in a series of Cu–Al binary alloys ranging from 0 to 15 wt% Al that were processed by different routes. These bronzes were worn against bearing steels in a non-lubricated block-on-ring configuration. Application of established and less common imaging methods supports the transition from sharpened dislocation cell formation at higher SFE to deformation twinning at lower SFE. At the wear process level, primary and secondary wear debris affect model development because their relative influence changes over time [314]. Takagi et al. [315] correlated wear behavior and microstructural evolution in Mg–Zn–Y alloy with long-period stacking ordered phase (LPSO). The Mg96Zn2Y2 alloy with uneven LPSO-phase layers and $\alpha$-Mg grains, including fine plate-like LPSO phase, was found to exhibit smaller wear mass loss compared to that of the Mg97Zn1Y2 alloy without the fine plate-like LPSO phase. The dynamic crystallisation of the $\alpha$-Mg grains increased the wear mass loss. The presence of a fine plate-like LPSO phase inside the $\alpha$-Mg delayed the dynamic crystallisation of $\alpha$-Mg grains under the lower normal load while it reduced the thickness of the dynamically crystallised layer after sliding deformation under the higher normal load in the Mg96Zn2Y2 alloy. Zugelj and Kalin [316] investigated the multi-asperity contact behavior of various steels, an aluminum alloy, and a polymer by using an in-house designed test apparatus which enables *in-situ* submicron-scale analyses of the entire multi-scale asperity contacts between the sapphire flat surface and the tested surface. They reported low contact areas, at most 12%–25% of the nominal value, and small asperity deformations of 40%–57% of the maximum peak height, at yield stress, both depending on roughness. The polymer contact area is up to 70% of the nominal value at the yield, independent of the roughness. Wear debris entrapment in annular contacts [317], particle dynamics in reciprocated sliding [318], and particle emission from Cu-free brake materials [319] are investigated respectively. These experiments showed that wear decreased with the ring width for aluminum samples while no clear correlation between ring width and total mass loss or steady-state wear rate for steel samples, non-repeated sliding gave rise to a reduced debris formation instead a steady increase in friction, and Cu-free brake pads generated more airborne particles than Cu-contained brake pad. Cappella and Reichelt [320] correlated the lognormal distribution of wear coefficient values measured in the three tribosystems (100Cr6 spheres on 100Cr6 discs at relative humidity rH = 10%–70%, 100Cr6 spheres on 100Cr6 discs at rH = 91%–100%, and SiC spheres on SiC discs) with the variable wear coefficient, statistical wear debris size, and real contact junction number.

### 3.2.8 Abrasive wear

Scratching or plowing by a single rigid asperity on a softer solid surface has been tested on precision scratch testers or atomic force microscope (AFM) at macro-micro-nano-scales, and has been numerically simulated by using finite element method (FEM), discrete element method (DEM), molecular dynamics (MD) simulation, or coarse grain molecular dynamics (CGMD) simulation. These experimental and theoretical studies are driven by the needs and interests for in-depth understanding plowing component of friction force, abrasive wear and/or precision manufacturing. Pereira et al. [321] conducted multi-pass scratch test on pearlitic steel by using a Vickers indenter installed on a UMT-2 tribometer under different loads (4 and 18 N) and numbers of sliding cycles (1–12 cycles). They reported that the successive sliding cycles led to an increase in the plastically affected region, surface crack nucleation and propagation, and brittle delamination. The initial random orientation of the pearlite colonies did not influence the new grain (colony) orientation produced during the test. The pearlitic steel work-hardened after a few passes. Xia et al. [322] investigated the single asperity wear of an austenitic steel by using an Agilent/KLA G200 nanoindenter with a spherical diamond tip (nominal radius 5 um). The applied normal force for indentation and wear tests was 40 mN and the loading/unloading rate was set as 0.5 mN/s. They divided the ploughing process into four stages: indentation, transition, steady-state, and unloading. During the transition stage, the center of slip-step pattern and pile-up tended to remain in the same direction with respect to the wear direction. The symmetry of the pile-up was broken by the wear motion. The relative activity of slip systems changed depending on the grain orientation, and dissociated dislocations occurred during wear while indentation is dominated by full dislocations. They compared the austenitic steel results with those of cupper and cementite steel, and observed that the conservation of contact area, elastic recovery, and the front pile-up development were the dominant mechanisms [323]. To simulate the grinding process of single crystal silicon carbide (SiC) with diamond grit, single scratch experiments with a simulative grinding test rig were performed [324]. It is reported that the key damage parameters of diamond grit are the contact width and the breaking angle, and the key factor to reduce surface damage is the state of the micro-edges that appear on the crystal plane of the diamond grit in contact with the workpiece during the scratching process. AFM tip-based nanoscratching was used to study the formation mechanism of bundle structure on polycarbonate (PC) surfaces. The formation of bundle structures was determined by a critical friction force, and a stick–slip process was used to explain the formation mechanism [325]. Mishra et al. [326] applied a material point method (MPM) based plowing model to study the effect of asperity geometry on the ploughing behaviour of an elliptical asperity sliding through a rigid-plastic substrate, and the simulation model was validated to be in good agreement with the friction forces and ploughing depths measured from the ploughing experiments on lubricated steel sheets with ellipsoidal indenters up to certain sizes and orientations.

Abrasive property of hard ZnO powder with different nanoforms was evaluated on a pin-on-disc tribometer. It is reported that the abrasive nature of ZnO nanoforms is correlated with the crystallite dimensions. Spherical particles cause a higher damage on surfaces at the interface, with a consequent major change in the roughness [327]. Effect of SiC abrasive particle size distribution and ball material (304 stainless steel and ANSI 52100 bearing steel) on wear coefficient was investigated by using micro-abrasion wear tests. Results indicated that a change in ball material, with consequential modification in the ratio between the hardness of the body and the counter-body, enabled different behaviors of the wear coefficient with the variation of the granulometric distribution. The wear coefficients were analyzed in each case, resulting in a map to describe the combined influence of granulometric distributions of abrasive particles and ball material on the wear coefficient [328]. On the other hand, abrasive wear resistances of low carbon steels [329], martensitic steels [330, 331], 42CrMo4 journal bearing steel with a AlSn20Cu liner [332], and Ni–Cr–B–Si coating [333] by hard abrasives of gravel (size 2–10 mm), abrasive paper, natural granite abrasives, $Al_2O_3$ powders, and sand/rubber wheel were evaluated respectively.

#### 3.2.9 *Oxidative wear and tribochemical films*

Natural oxidation and tribo-oxidation have important impact on wear of metallic materials. Moghaddam et al. [334] investigated the effect of oxidation at low temperature on dry sliding wear of self-mated nanostructured carbide-free bainitic steels and compared with those with quenched and tempered martensitic microstructure. They reported that wear resistance of the carbide-free bainitic steel was better than the quenched and tempered martensitic steels, the reason of which is attributed to the formation of a thin and mechanically stable compositional mixed layer on top of the surface together with a hard underlying substrate. Oxidative wear is more serious at high temperatures and/or high sliding speeds. $ZrB_2$–20 vol% SiC ultra high-temperature ceramics pin-on-high carbon steel disk tests were done at speeds up to 37 m/s [335]. It was found that two subsurface layers were formed after wear test. The first one was a mechanically mixed (transfer) layer composed of an iron-containing borosilicate glass mixed with the tribo-oxidation products resulted from oxidizing both ceramics and steel components. The second underlying layer was a partially oxidized zirconium diboride. Such a combination of quasi-viscous transfer layer and partially ductile underlying layer allowed reducing friction, forming a smooth worn surface, and protecting the ceramics against brittle subsurface fracture. Senge et al. [336] developed a mathematical model to predict the damage scenario of nickel-based superalloy subjected to high pressure and high temperature as in turbines.

Various kinds of lubricants and additives can form tribochemical films, which protect the rubbing surface from severe wear. The chemical tribofilm of ionic liquids added PAO2 oil formed on 100Cr bearings in friction test was analyzed by using ToM-SIMS [337]. It was reported that metal surfaces that have been immersed at high temperatures in phosphonium phosphate-containing oils contained at least some characteristic signals for phosphate containing anti-wear layers. Tribochemical film of natural serpentine mineral particles in oil were formed in SRV4 wear test of tin bronze disk, and the tribofilm was wear resistant, non-conductive, and composed of metal oxides, oxide ceramic particles, graphite, and organics [338]. It is reported that the wear resistance of titanium-matrix composite (TMC) against M35 steel was improved by adding multilayer graphene (MLG)/$Fe_2O_3$ nanocomposite and MLG + $Fe_2O_3$ mechanical mixture into base oil, but the addition of MLG or $Fe_2O_3$ had no effect [339]. Esfahani et al. [340] found that the tribofilm formation of zinc dialkyldithiophosphate (ZDDP) and molybdenum dithiocarbamate (MoDTC), as well as water contamination in oil can impede hydrogen ingress in the steel substrate, by impeding the formation of fresh metal surfaces. This reduces the generation of hydrogen atoms by preventing the decomposition of hydrocarbon molecules. A uniform tribofilm across the wear track also acts as a physical barrier for hydrogen permeating through the tribo-contact. The water contamination in the oil negatively influenced tribofilm properties and therefore led to higher permeation of hydrogen into the steel.

#### 3.2.10 *Wear in metalworking and polishing processes*

In metal-forming processes, hard tools contact with and enforce the workpiece with a lower hardness to plastically deform to a prescribed shape. The hard tools are usually made of high-cost materials and engineered through sophisticated manufacturing processes. Hence the wear of tools is desired to be as less as possible to ensure precision forming, and the tool life is demanded to be as much long as possible to save cost. Effect of lubrication with different lubricants on friction and wear in punching and blanking operations was investigated experimentally, combined with FEM simulation. The experiments revealed that an applicable temperature range and a high load bearing capacity are central lubricant properties necessary for ensuring sufficient lubricating ability for punching and blanking operations. Additive package based on Ca-, P-, and S-additives was suggested to replace the hazardous chlorinated paraffins EP additive in mineral oils [341]. Wear particles generated due to the relative sliding of the roll and the sheet metal in the roll bite are one of the main factors that contaminate the surface of a cold rolled steel sheet. Micro-tribology experiments in the form of single asperity scratch testing were done by Mekicha et al. [342], and they found that ploughing was the dominant wear mechanism in lubricated conditions, while wedge forming was the main wear mechanism in the absence of lubricant. The beneficial influence of chrome plating rolls on wear particles formation was found to stem from its interaction with the lubricant as the same influence was not observed in non-lubricated test conditions. Material point method (MPM) scratch simulations were also performed, and the simulations showed that almost all the frictional resistance arised from deforming the substrate in the case of an interfacial shear strength corresponding to the lubricated contact. In unlubricated sliding, however, the frictional resistance was primarily due to shearing of the adhesion junction and tearing the deforming body. Pin-on-disc tests between AA6082 aluminum alloy and G3500 cast iron were performed under step load change conditions. It has been found that contact load change will disequilibrate and rebuild the dynamic balance and high load will increase the generation and ejection rate of third body and vice versa. An interactive model was developed and presented to simulate the dynamic formation process of the aluminium third body layer under load change conditions, enabling multi-cycle simulations to model the galling distribution and friction variation [343].

In machining processes, tool wear should be controlled as low as possible. The laser surface texturing was applied to cemented carbide cutting tool, and focused ion beam (FIB)/FESEM was used to characterize the tool wear after testing against a steel workpiece in a workbench. It was demonstrated that: (1) Laser-surface texturing does not degrade the intrinsic surface integrity and tool effectiveness of hard metal pyramids; and (2) there exists a correlation between the wear and loading of shaped pyramids at the local level [344]. Adhesion of the workpiece material, such as nickel-based alloys, to the cutting tool is an important aspect that governs a number of physical parameters within the tool–chip interface. By using image processing of discrete gray intensities on backscatter images of uncoated tungsten carbide inserts, Alammari et al. [345] enabled the quantification of adhesion area for a variation of the cutting speed, based on a number of orthogonal machining trials carried out on nickel-based superalloy NiCr19–Fe19Nb5Mo3 (2.4668), using uncoated tungsten carbide inserts.

To make ultra-smooth and flatten surfaces, such as silicon wafers in semiconductor industry, the chemical-mechanical polishing (CMP) is widely used. Shear-induced chemical etching reactions of single-crystalline Si (100), Si (110), and Si (111) surfaces were studied in acidic, neutral, and basic aqueous solutions by Xiao et al. [346]. The critical activation volume and activation barrier were determined for mechanochemical etching of three crystallographic surfaces. The pH dependence of Si (110) etching is quite distinct from those of Si (100) and Si (111). The results suggested that the mechanochemical activation effect is larger for Si (100) and Si (111) than Si (110) which is chemically more reactive than the other two surfaces. CMP of precorroded $Lu_2O_3$ laser crystals [347], $CaF_2$ crystal [348], SiC substrate [349], and N-faced GaN surface [350] were researched experimentally. A new kind of electrical enhanced photocatalysis polishing slurry was developed and tested for polishing monocrystalline silicon carbide wafer [351].

### 3.3 Physical and digital modellings, monitoring, prediction, and assessment of wear processes

#### *3.3.1 Running-in and surface structure evolution*

Running-in, or break-in in other words, is the initial stage of operations in friction and wear, even a running-in like a pre-treatment before operation, usually lasting a short period of time, saying minutes or hours. It has been realized for a long time that this short running-in gives rise to great changes in surface topography, tribofilm generation, and microstructure evolutions of pristine interfaces, and thus affects friction, wear, stability, and life of tribosystems in the later stages of operations. A pair of ordinary rough contacting surfaces might become smoother either, or a pair of well-polished contacting surfaces might get roughened, after running-in, depending on the lubrication, load and speed conditions, and variations experienced in the running-in stage. Seeking for an optimal running-in scheme is always desired not only for better and longer operations of most of practical tribosystems, but also for the purpose of better understanding of friction and wear processes. Faller and Scherge [352] proposed a balanced strategy for optimal running-in in terms of stressing power (see Fig. 31), based on their systematic running-in experiments on a pair of iron-coated rough aluminum pin-on-DLC coated 100Cr smooth steel disk lubricated with SAE 0W20 engine oil for 4 h. They recommended high-power stressing, which induced tribochemical running-in, rather than low-power stressing which led to topographical running-in only. The judgements of good or not-good running-in included stability and sensitivity under varying operation conditions as well as the stable values of friction coefficient and wear rate. It should be noted that excessive stressing power during running-in might result in severe damages and even seizure failure. It does not means that the larger the stressing power, the better running-in. Pham-Ba and Molinari [353] investigated the evolution of surface roughness of smooth $SiO_2$-on-smooth $SiO_2$ disk under dry sliding condition experimentally and theoretically by molecular dynamics (MD) simulation. They confirmed that small spherical wear particles, whose minimum size was mainly dependent on the material properties was formed at initial wear stage, and they were the fundamental bricks in the formation of the third-body layer later. The wear particles grew into rolling cylinders and into larger aggregates, which took the form of flakes, creating a third-body layer and the macroscopic roughness. The monotonic decreasing in friction coefficient, monotonic or non-monotonic changes in surface roughness and other topographic features as well as gradual work hardening have also been reported during different types of tribopairs [354–357].

In addition to the changes in surface topography and hardness, microstructures of many tribopairs evolute during short running-in period under certain conditions. These changes include dynamic recrystallization of tribolayer [358], bainite, ferrite and martensite phase transformations of bainitic wheel steels [359], and grain coarsening of nanolaminated structure in martensite steels [360]. In severely deformed interface, material transfer from the plastically deformed softer part to the rigid surface might happen,

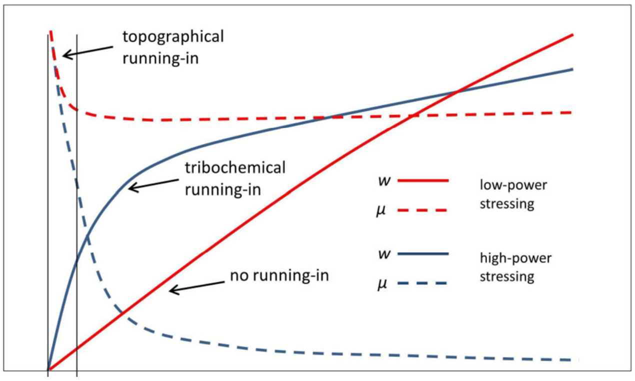

**Fig. 31** An illustrative depiction of the changes in friction coefficient $\mu$ and cumulative wear $W$ during running-in (horizontal axis) under low-power or high-power stressing [352]. Reproduced with permission from Ref. [352], © The authors, 2020.

which has been studied carefully by Heldin et al. [361]. The friction-induced microstructural evolutions are sometimes used as a pre-treatment of mechanical components to enhance their wear resistance, such as reported in Refs. [362, 363].

#### 3.3.2 *Physical modelling and testing*

There are abundant publications on physical modelling and testing of various tribopairs with various testing methods and conditions every year. It is not possible to comment every paper on this topic, instead only a limited number of papers are reviewed here. New friction and wear testers are demanded for evaluation of tribology performance in extreme environment and work conditions, which are not able to be simulated on conventional tribometers. A friction testing apparatus for simulating the ultra-high pressure environment of deep-ocean [364], a tribology cell attachable to a rheometer for simulation of grinding process of steel balls by coated sandpapers [365], and a whirling arm rain erosion rig for evaluation of wind turbine materials against raindrop impacts [366] have been built respectively. Accelerated testing of carburizable martensitic stainless steels for aerospace bearings in harsh environments has been performed by using electrochemical tests under elevated corrosion conditions [367]. Advanced sensing technologies, particularly *in-situ* measurements, are useful for characterizations of friction, wear, and lubrication in testing or practical operation. A time-frequency based approach for analysis of acoustic emission [368], *in-situ*-determination of tribologically induced hydrogen permeation by using electrochemistric methods [369], and a Tribo-Raman-spacer layer imaging method (SLIM) which is able to detect tribofilm formation process and tribofilm thickness on the sliding surface have been developed [370]. Besides of the evaluations of wear resistance of materials by using laboratory standard tribometers, system level or assembly level bench tests, such as full-size journal bearings, ball bearings, gears, are not replaceable in industry. The self-lubricating spherical plain bearings (type GE17ES) [371], full-size five-pad tilting-pad journal bearing [372], wet clutches [373] have been tested with bench testers. Full-scale testing of low adhesion effects with small amounts of water in the wheel/rail interface has been carried out on a tram wheel test rig [374]. Friction and wear tests of journal bearings in aircraft turbines under close-to-application conditions [375], of ball bearing under starved lubrication [376], of spur gears in simulated loss-of-lubrication condition [377] have been reported respectively. How to measure sliding wear in an efficient way and how to accelerate rolling contact fatigue have been discussed in Refs. [378, 379] respectively. Cousseau and Passos [380] discussed the methodology for wear mapping error quantification, and Kraus et al. [381] proposed a test method for characterization of the contact pressue-dependent friction by using a single sheet metal compression test. The wear performances of 3D printed spur gears, made of Nylon 618, Nylon 645, alloy 910 filaments, together with Onyx and Markforged nylon proprietary materials respectively, were measured on a gear test rig [382], and it revealed that gears 3D printed using Nylon 618 actually performed better than injection moulded nylon 66 gears when low to medium torque was applied. The wear resistance of Mo–Se–C transition metal dichalcogenide coatings was evaluated on a ball-on-disc tribometer against a 100Cr steel ball in ambient air. As shown in Fig. 32, the new solid lubricating coatings have relative high wear resistance in 100,000 cycles (wear rate ~ $10^{-7}$ $mm^3/(N\cdot m)$), and that of the counter steel ball is extremely low (wear rate ~ $10^{-10}$ $mm^3/(N\cdot m)$) at the same time [383].

#### 3.3.3 *Wear monitoring*

Wear is the loss of materials, a portion of which transports into flowing lubricant or emits airborne as tiny debris, from sliding or rolling interfaces. Detection, collection, and analysis of the wear debris *in-situ* or *ex-situ* are informative and the relevant techniques have been researched for several decades. Ferrography, a technique by which micro-size wear debris of ferrous metals are trapped at a zone under an electromagnetic field and optical images of the trapped debris are captured and analyzed. Fan et al. [384] proposed a deposition rate-based index of debris concentration (IDC) method to eliminate the influence of bubbles on debris identification for improvement of online image visual ferrography (OLVF), and applied it to online analysis of 1505 bubble-debris videos captured in a full-life accelerated gear wear

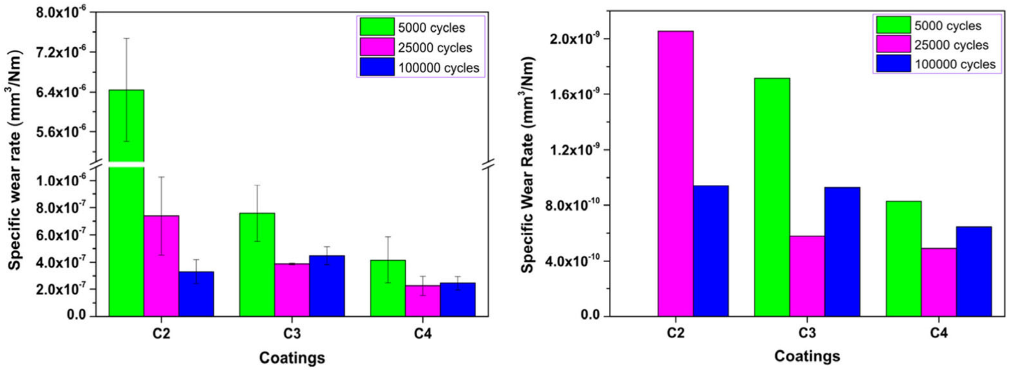

**Fig. 32** Comparisons of the wear rate of different coatings (left) and the counter steel ball (right) in different numbers of sliding cycles. C2: $MoSe_2$ doped 18%C; C3: $MoSe_2$ doped 22%C; C4: $MoSe_2$ doped 27%C [383]. Reproduced with permission from Ref. [383], © Elsevier B.V., 2020.

test. Wear debris were separated from used grease from 20 spherical rolling bearings taken out from urban passenger rolling stock during a routine maintenance programme. Spherical, laminar, and chunk wear debris were most frequently found among seven types of wear debris identified in the grease lubricated SRBs. Morphological examinations of wear debris/particles suggested the most active wear in grease lubricated SRBs is predominantly associated with the rolling/sliding contact and adhesive mechanisms. Moreover, wear debris naturally generated in grease SRBs experienced further deformation by interacting with other particles and/or the passage of a wear particles through a rolling contact, hence the complexity of surface features [385]. Wear particles emitted from an automobile brake under five driving cycles with a brake dynamometer were collected with 4 different instruments and compared the mass concentrations obtained by the different instruments. The density of brake wear particles was observed to be larger than 1.0 g/cm$^3$ because the optical mode diameter of the brake wear particles was smaller than the aerodynamic mode diameter. The brake wear particles emissions were shown to be proportional to the brake dissipation energy and brake initial velocity of each driving cycle. Although the same dissipation energy was applied by braking, more brake wear particles were found to be emitted at high initial velocity condition [386]. The wear particle analysis technique was used to the condition monitoring of worm gear wear tests done under 16 different lubrication conditions, together with other wear analysis methods [387]. *In-situ* optical imaging and thermography techniques are also useful tools to characterize wear degree of mechanical parts during laboratory testing. These monitoring methods have been used in silicone nitride ball sliding on steel disc under dry condition [388], *in-situ* determination of wear surface during tribocorrosion tests of ferrite stainless steel in sulphate solutions [389], and analysis of the role of the glaze layer generated in a cylinder-on-flat fretting wear test of 310S stainless steel [390]. Woski et al. [391, 392] discussed the automated classification of worn surfaces based on image analysis of wear scars.

Acoustic emission is another useful condition monitoring technique. It was used in a pendulum rig to model two-body abrasive wear [393], in monitoring friction and wear of journal bearings of geared turbofans [394], in state evolution identification of dry gas seal during repeated start–stop operation [395], in identification of damage and wear mechanisms in a steel/glass ball-on-disc sliding contact [396], in assessment of surface fatigue wear developed in the roller-bearing system [397, 398], in investigation of galling wear in a semi-industrial stamping test and scratch test [399]. Surface acoustic wave measurement was utilized in *in-situ* detection of lubricant thickness in rolling bearing [400], and the eddy current measurement was applied to monitor the wear depth development of elastomer materials in a tribologically equivalent system for radial shaft seals [401].

### 3.3.4 *Numerical predictions of wear profile and wear rate*

Numerical predictions of wear are as important as physical modelling and testing of wear, but more difficult because of the lack of sound wear failure criterions and laws. A number of assumptions, reasonable or non-reasonable, have to be made when numerically predicting wear failure, wear life, wear rate, and wear particle generation and transportation. Contact mechanics analysis of rough surfaces under dry or lubricated conditions is required as the first step for numerical predictions of wear events. Based on the continuous or discrete distributions of contact pressure and local frictional shear stress, volume of adhesive wear is usually predicted with the classical Archard's wear law along with experimentally obtained wear coefficient, while fretting wear is calculated by an energy dissipation model. Degradation of contacting surfaces during sliding and rolling is simulated with an assumption of damage evolution model, while the fatigue life is often estimated with a crack initiation and propagation model. In recent years, discrete modelling methods, including discrete element method, molecular dynamics simulation, discrete dislocation dynamics, peridynamic modelling, etc. for wear process predictions become more and more popular for the asperity scale and atomic scale wear, or multiscale simulations. Whatever the numerical modelling approaches, predictions of wear rate and wear failures in a reasonable accuracy without pre-modelling experiment-based parameters are unconfident, even not impossible, at present.

Numerical predictions of wear profiles of journal bearing with single- or multi-scale surface patterns [402], of misaligned and dynamically loaded engine bearings with roughness [403, 404], of piston-cylinder pair in axial piston pumps [405], of a thrust roller bearing [406], of a reciprocating rod step-seal [407], of spur gears considering contact temperature effect [408], and of aviation gear coupled with contact fatigue [409] have been performed by using the Archard's wear law along with a mixed lubrication and rough surface contact modelling. The Archard's wear equation was also used in wear calculations for the dry sliding/rolling contacts of spur gears [410], at a bimaterial sliding half-planes [411], of ball-end milling cutter–workpiece interface in forming of microstructured surface [412], of wheel in the motor and unpowered car of a high-speed train [413], of rail side wear in urban transit track [414], and of the wheel–rail at weld irregularity in metro lines [415]. Instead of the Archard's wear law, adhesive wear based on Usui equation was applied to predict wear on cutting face and flank coupled with the finite element method (XFEM)/multi-level-set simulation of cutting process [416]. Some of scientists regard adhesive wear as a result of progressive and accumulative damages in the subsurface layer, the evolution of which determined by the elastic/plastic strain. A so-called brick model was used in the thermo-mechanical coupled finite element analysis of rolling contact fatigue and wear of a rail steel under different slip ratios [417]. Fereidouni et al. [418] found a correlation between the depth of the maximum subsurface shear stress obtained from a dry point contact model and the measured wear rate in a series of running-in experiments. SEM images of all three friction-pair tested revealed that the size of the wear particles was directly related to the applied load. The finding hints wear rate, or wear coefficient in the Archard's equation would be probably estimated a priori, instead of measuring experimentally. Wear predictions based on frictional energy dissipation were performed for fretting wear [419, 420]. Terwey et al. [421] compared the adhesive wear in dry and mixed lubrication between the Archard's equation and an energy-based model, and they concluded that determining a critical number of load cycles for each asperity, a local wear coefficient, and local wear depth can be calculated with the energy-based model. According to the theory of Kragelskii, adhesive wear rate can be estimated by using a friction fatigue failure equation of asperities. This model was used in wear calculation for the slide guide of machine tool considering boundary lubrication [422]. In Ref. [423], a multiscale method based on quasi-continuum medium (QM) principle was proposed to describe the degradation process of guideway linear precision with an exponential wear model, which uses the wear exponent to describe the wear status based on linear precision measurement rather than tremendous wear volume tests. Discrete element method (DEM) and crystal plasticity model were used to predict cement tool wear in cutting Inconel 718 alloy [424] and the microscale abrasive wear of a polycrystalline high manganese austenitic steel block by a rigid semi-sphere [425], respectively. Continuous material damage and fatigue model were applied to predict the fretting wear life of selective laser melting (SLM) aluminum

AlSi10Mg [426] and the fatigue life of heavily loaded rolling/sliding EHL contacts in rolling bearings [427], respectively.

In many practical engineering applications, wear mode and mechanism change with operation time. In Refs. [428, 429], the transitions of wear mode and predominant wear equations were discussed for the cases of case-hardened 20MnCr5 under cyclic line rolling contact and the rail damage respectively. Li et al. [430] predicted erosion wear and lifespan of machine gun barrel under typical shooting conditions by using a slow erosion stage wear model and a thermochemical erosion stage wear model.

#### *3.3.5 Big data, machine learning (ML), and artificial neural network (ANN) application for wear monitoring and analysis of tribosystems*

At present, applications of ML and ANN are hot topics in a variety of engineering fields. ML is a sub-branch of Data Science and emphasizes on special techniques and methods aimed to provide an "intelligent final result" for a computing machine by utilizing specific algorithms for data analysis. ANN is a special set of mathematical algorithms based on representation how the human thinking can be mimiced by the computer. The effectiveness of ML and ANN depends on big data available for training the data-driven models. For wear monitoring and analysis of industrial tribosystems, *in-situ* detections of friction and wear of individual tribo-component involved in a machine in operation require to embed a number and different kinds of sensors into the system, and thus are not easy to realize in most of cases. Nowadays, vibration, temperature, ferrography (or other spectrum instruments), and acoustic emission data can be measured in real-time for some of important mechanical systems during operation. Inputting such *in-situ* data measured previously in a particular tribosystem, merged with previously obtained laboratory experiment data if available, to the input layer of a proper parameter-adjustable ANN model, and minimizing a suitable functional of the deviations between the outputs and the observed wear events, the optimal values of the model parameters can be obtained by using an appropriate optimization method, such as the Bayes's rule. In general, the ANN models available nowadays can be roughly separated to the three types: Static (ordinary) ANN, dynamic (recurrent) ANN, and "smart" (improved by internal algorithm for specific calculation) ANN. An ordinary ANN mainly used as a "universal approximator" to provide the nonlinear connection between input and output data. A dynamic (recurrent) ANN is used to learn the nonlinear behavior of variables as sequenced events in a time, where the events in a past have influenced on the upcoming event, and can be applied to describe the time-dependent behavior of friction and wear process. ANN based on specific internal calculation, as a convolutional NN, is used for estimation a "hidden features" of worn surface images and wear debris analysis, or coding/encoding nonlinear frequency dependent data (such as acoustic emission data), or classification problems.

Any type of ANN uses a randomly initialization of neuron's weight. The estimation of appropriate set of weights comprises the use a cost function, providing the minimum error between existing state and desired final result. This process is referred to as training mode, the data used in the training process is the training data, and ANN models are referred to as data-driven models. Generally speaking, the bigger the data set is, the better the trained model. Once a successful trained ANN model is established, it possesses the power of wear event predictions for that particular tribosystems, and thus is attractive for the purpose of wear monitoring and analysis. König et al. [431] established a data-driven wear monitoring recurrent neural network for wind turbine transmission sliding bearings by using acoustic emission signals, which are sensitive to the transient lubrication from mixed regime to hydrodynamic regime, as training data. They confirmed that the data-driven model had the capability to predict wear volume of a sliding bearing (see Fig. 33). Wear loss of plasma transfer arc (PTA) coatings on AISI 1020 steel was predicted by using an ordinary feedforward artificial neural network and basic, kernel-based, and weighted extreme learning machine trained with the data of microhardness measurements and laboratory dry sliding wear tests [432]. Pandiyan et al. [433] tried to identify abnormal tribology events, such as scuffing, which are difficult to predict using physics-based numerical modelling,

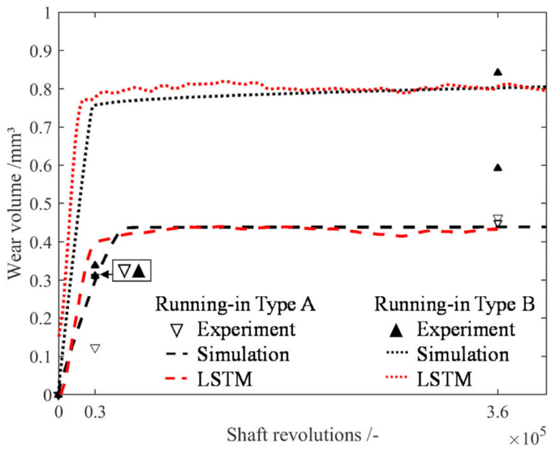

**Fig. 33** Comparisons of wear volume vs shaft revolutions between experiment, physical simulation, and the data-driven ANN (LSTM) for two types of running-in [431]. Reproduced with permission from Ref. [431], © Elsevier B.V., 2021.

by using a microphone and semi-supervised machine-learning algorithm implemented into recurrent NN. A simple ANN approach based on the "universal approximation" has also been applied to predictions of fretting wear life [434], of surfactant concentration effects in electroless Ni–B coating [435], of wear resistance of binary coatings of Cr3C237WC18M and WC20Cr3C27Ni [436], and identification of 3D ferrography wear particle images [437, 438]. Prost et al. [439] conducted semi-supervised classification of the state of operation in self-lubricating journal bearings using a random forest classifier.

Comparing with physics-based numerical prediction methods mentioned in Section 3.3.4, the data-driven models are less complicated, more robust, and faster to run. Its disadvantage is non-interpretable black-box, that provides non-linear regression between input and output data regardless of the real physical mechanisms could exist in certain wear process, giving the impression that the "hidden physics process" was learnt by ANN. Another disadvantage is of low degree of transferability between different tribosystems. Because a simple ANN trained with an experimental data set or from certain experiment's set-up and conditions is difficult to be extended to other experiment's set-ups.

### *3.3.6 Failure analysis, multi-dimensional assessment, and optimization of wear*

Failures due to severe wear are frequently encountered in fields. Determining the major failure causes and mechanisms is crucial to find a right solution. A case study of failure analysis of the main rotor joint bearing of the R44 helicopter in service was reported in Ref. [440]. Through a specially designed experimental scheme, it was found that severe wear damage to the fabric liner was the primary cause of the failure, which occurred non-uniformly in circumferential direction. The wear mechanisms involved in the case were fatigue wear, adhesive wear, and some abrasive wear and oxidation wear. Failure modes found even in laboratory tribology experiments are generally compound, and identifying the key factors needs repeated tests and thorough analysis. Wagner and Sundararajan [441] conducted 34 three-points rolling contact tests on a micro pitting rig (MPR) with variable rolling/sliding contact conditions and low lambda ratios, and they found there were three distinct failure modes: Micropitting, abrasive wear, and pitting. These results were used to evaluate three different mapping techniques: (1) Pmax vs. lambda ratio, (2) PV vs. lambda ratio, and (3) a modified Scm (line contact) vs. lambda ratio. A transition point existed, at which the primary failure mode switches from abrasive wear/pitting to micropitting, driven primarily by the lambda ratio. The experiments showed that (1) increasing film thickness, and thus reducing asperity contact, does not necessarily reduce the likelihood of micropitting in low lambda conditions, and (2) when the lambda ratio is less than 0.1, there exists a sensitivity where one cannot predict the resultant failure mode, most likely due to competing failure modes.

How to do laboratory experiments in conditions and test schemes as close as in field? How to transfer the data and findings obtained from laboratory tests to assessment of wear behaviors of real tribosystems? How to validate numerical predictions of wear processes, wear rate, and life by laboratory experiments and field data? How to improve and optimize the design of tribosystems for achieving better performance? These are questions asked from applications but no quick answers existing. For evaluation of the wear resistance of materials used in ships under ocean operating conditions, an experimental apparatus was developed, which is capable to perform several specimen tests involving parallel jet erosion, angle jet erosion, cavitation erosion, electrochemical corrosion, and their combinations under ocean operating conditions. The adjustable parameters in the experimental apparatus include the impact speed, impact angle, environmental pressure, etc. [442]. A series of unidirectional ball-on-disc tests were performed in synthetic seawater with the zero resistant ammeter technique. The differences on the currents registered for AISI 316 SS (passive) and a low-alloyed steel (non-passivating or active) evinced the high rate dissolution of the active steel even in the absence of sliding, while the AISI SS showed negligible electrochemical activity. The results showed little influence on the agitation for the passive material, but a considerable effect on the wear-accelerated corrosion in the unworn surface of the non-passivating steel [443]. Ayerdi et al. [444] presented a critical assessment of wear volume determination methods for ball-on-flat linear reciprocating sliding tribological tests. It revealed that the ASTM D7755-11 standard leads to the highest relative errors (up to 106%) and deviations (up to 27%) depending on the regularity of the wear track shape. They suggested improvements for the ASTM D7755-11 wear computation, which can reduce errors from 106% to 17% when analyzing irregularly shaped wear tracks. They also pointed out that the most used methods for wear determination are overall reported with incomplete procedural information for wear computation. By reviewing the papers published in tribology journals, Llavori et al. [445] criticized that 41% of the papers did not specify the derivation method used and 37% employed the least effective methods. They suggested to use the geometric independent coefficient of friction method (GICoF) for gross slip regime due to its robustness, followed by the energy method (ECoF). Zhang et al. [446] carried out life test of ultrasonic motors for 3,041.7 h with eight motors divided into four groups with different torque loads. Some start-up problems occurred during the early time in the life test, and some targeted suggestions are proposed to overcome the problems. The erosion damage to multiple target vessels operated at the Spallation Neutron Source was measured using a high-resolution laser scanning system, and a cavitation progression modelling procedure developed using empirical observations was compared to measurements of samples from target vessels after operation [447]. For assessment of gas power leakage and frictional losses from the top compression ring of internal combustion engines, a multi-physics integrated numerical prediction approach was presented, which accounts for flexible ring dynamics, ring-liner thermo-mixed hydrodynamics, and gas blow-by. The effects of transient ring elastodynamics, thermal gas flow through piston crevices upon chamber leakage pressure and parasitic frictional losses were investigated, and the predictions showed close conformance to frictional measurements under engine motored dynamometric conditions [448]. An experimental-computational approach for localized durability assessment of sliding contacts was proposed and applied to journal bearings [449]. Internal tribosystem interactions between the system components were obtained by the experimental model tester, while the consideration of different hydrodynamic and asperity contact pressure at the target system were managed in a computational way. To transfer tribology knowledge to better designs of rolling element bearings, Zhou and Lugt [450] discussed the applicability of the mechanical aging Master–Curve where the yield stress of greases is given as a function of the imposed energy corrected for temperature using an Arrhenius equation. They pointed out that it does not apply to those areas in the bearing where the centrifugal forces are high such as under the cage/inside the running track. However, it does apply to the grease that is aging in the un-swept area of the bearing, i.e., the area in the bearing where the grease undergoes moderate shear. This Master–Curve makes it possible to develop a remaining grease life model for bearings operating under moderate temperatures. Vrček et al. [451] studied the effect of hardness and heat treatment of different rolling bearing steels on micro-pitting and wear. It was demonstrated through rough-on-smooth two-disc tribo-testing that the faster, rough surface undergoes only mild wear and plastic deformation of asperities. However, three different damage modes were observed and assessed on the slower, smoother surface, where the damage mode showed a strong dependence on a surface hardness difference between the smooth and the rough surface. Furthermore, it was observed that a plain medium carbon steel (DIN C56E2) showed better surface fatigue resistance than a low alloy high carbon steel (DIN 100Cr6) at similar surface hardness levels if surface induction hardening is applied on the former. Correlation between the tribological behavior of polymer-steel contacts and the performance of real-scale gears was reported [452]. The results showed that tests at 50 °C in slide-roll ratio (SRR)-variable model tribotests can predict the SRR-dependent wear mechanisms along the meshing path in real gears, while the 80 °C tests are too severe, leading to thermal overload. An about 10-times-larger wear coefficient, compared to the gear tests, was measured during the SRR-variable tests, which is a better estimation compared to the common pin-on-disc test with 2–4 orders-of-magnitude deviation. Base on experimental investigations, four different methods and measures to protect a sealing system with radial shaft seals (RSS) from shaft wear were presented and discussed [453]. For improving assessment of fretting fatigue life, the theory of critical distances with mesh control was recommended in design [454], and a global-local fretting design methodology for the pressure armour layer of flexible marine risers was outlined [455].

### 3.4 Surface treatments and modifications

The terms of surface treatments and modifications used here mean a category of non-additive surface engineering, covering in-plane shearing/rolling, out-plane impacting, selective removal of materials, high-temperature or low-temperature heat treatments as well as external field impulsion applications. Such a treatment results in remarkable and beneficial changes in surface topography, physical and chemical properties of surface, residual stress and microstructures in the subsurface layer, which are as important as the pristine bulk properties of materials for tribology performance. The publications within the following four sub-categories will be reviewed one-by-one.

#### *3.4.1 Ultrasonic nanocrystalline surface modification (UNSM)*

UNSM is an effective surface nanocrystalline technique that transmits mechanical energy to the surface and subsurface layer through a free-rolling hard ball forced by an ultrasonic vibration. The UNSM treatment results in severe plastic deformation of the surface layer and yields low surface roughness, high surface hardness, and large depth of compressive residual stress distribution. UNSM is also regarded as ultrasonic surface rolling process (USRP) or surface mechanical attrition treatment (SMAT) in some literatures. It has been applied to various types of steels, including DZ2 axle shaft in high speed trains for improving the wear resistance [456], 17-4PH steel for enhancement of plain fatigue and fretting fatigue resistances [457], titanium alloy Ti5Al4Mo6V2Nb1Fe assisted by electric pulse for increasing fatigue properties [458], 300M steel for improving surface integrity and wear resistance [459], Inconel 690TT for increasing fretting wear resistance [460], selective laser melted 316L steel for enhancement of corrosion and wear resistances [461], polymer/composite coated 316L steel for enhanced sliding wear resistance [462], thermally-sprayed WC–Co coating [463], 1045 steel [464], cementitious materials for improving wind-blow and erosion resistance [465], boron-doped DLC coated 52100 steel [466], and 4150H steel [467].

#### *3.4.2 Particle shot peening, laser shock peening, and plasma jet hardening*

Surface peening leads to the similar effects to UNSM. Shot peening transmits high kinetic impacting energy of small particles onto solid surface, and produces multi-aspects beneficial results. By using shot peening, the fretting fatigue strength of 7075-T651 aluminum alloy has been improved [468], the dry sliding wear resistance of the additive manufactured alpha titanium has been increased [469], friction coefficient of the gear steel has been reduced [470]. Severe vibratory peening (SVP) is a variant technique of conventional shot peening, and it has been applied to harden hot rolled 1020 mild steel [471]. Laser shock peening is an advanced peening technique, using pulsed laser energy instead of kinetic mechanical energy. It has been used in a variety of applications, including surface modification of NiTi alloy [472], of 20Cr2Mn2Mo steel alloy [473], improvement of cavitation erosion of 420 stainless steel [474], of tribocorrosion properties of AZ31B Mg alloy [475], and of tribological properties of magnesium alloy ZK60 [476]. Plasma jet [477], and ultrasonic impacting [478] were used to harden P20 mold steel and 1045 steel, respectively.

#### 3.4.3 Reductive surface texturing

Reductive surface texturing is a surface finishing process to make regularly distributed micro/nanoscale dimples on surfaces by using dry or wet etching, high energy beam ablation, electrical discharging or machining. In fact, UNSM and surface peening mentioned above can also generate dimpled surfaces along with surface hardening. The reductive surface texturing described here is capable of producing more flexible shapes and distributions of dimples, grooves or other cavities, but keeping the hardness of the substrate almost un-changed. Surface texturing can also be fabricated by using additive manufacturing (see Section 3.5), or by plastic deformation, or molding. Here only some typical reductive surface texturing techniques and their tribological effects are selected and the relevant papers are reviewed. It has been well known that micro/nano-dimples can enhance load carrying capacity and reduce friction in full film hydrodynamic lubrication regime through micro-bearing effect, and serve as arrays of micro/nano-reservoirs for liquid lubricant, liquid/solid additives and wear debris in boundary lubrication regime and dry sliding. It is also helpful for heat transfer due to extended surface areas. However, little effect or even negative effects were also reported compared with texture-free surfaces, if the surface texture is not appropriately designed and/or fabricated in some cases. Laser surface texturing is most studied and a widely applied technology, which has been used in surface modification of piston ring [479], on stator surface in ultrasonic motors [480], on the Ti–6Al–4V biomedical materials combined with chemical assembly of a graphene oxide film [481], on a $Si_3N_4$ disc sliding in water against a $Si_3N_4$ ball [482], on UHMWP samples for bio-implants [483], on TC4 (Ti–6Al–4V) titanium alloy disc sliding against a UHMWP ball in simulated body fluid (SBF) [484], on a lead bronze coating surface [485], on a 42CrMo4 steel disc reciprocating under starved lubrication [486], and on the water-lubricated thrust ball bearing washer surface made of high density polyethylene (HDPE) specimens, manufactured using 3D printer technology [487]. Ding et al. [488] constructed 3 types of dimpled surfaces, type A: 'wide & deep' by pico-second laser ablation, type B: 'narrow & shallow' by one-step post-polishing type A, and type C: 'wide & shallow' by two-step post-polishing of type A, on a smooth AMS5630 440C stainless steel sample for investigation the effect of surface texturing on material transfer behavior of the X1 PTFE/glass fiber woven fabric composite (bonded by phenolic resin) when sliding against the textured steel samples. Tribological performances of textured surfaces, fabricated by photolithography-dry etching and molding processes [489], a maskless electrochemical texturing method [490], indentation with a hard hammerhead [491], and micromolding [492], have been respectively investigated. In Ref. [493], tribological performances of Al–Si alloy cylinder liner prepared by different surface shaping treatments were compared. Laser surface texturing was combined with other surface engineering, including selective laser melting and plasma nitriding [494], magnetic field [495], or hybridized with polymer solid lubricant [496]. Effects of surface texturing on hydrophobicity of coatings [497], slurry erosion resistance [498], and friction amplification [499] were also investigated.

#### 3.4.4 Heat treatment and cryogenic treatment

Traditionally the crystal structure and hardness of metals and alloys are adjusted by using proper heat treatments. Plasma selective quenching (PSQ) is an advanced heat treatment technique, in which high-energy plasma quickly heats the material surface to a point above its austenitizing temperature, and then the heated material surface will cool down quickly due to its good thermal conductivity, generating a hardened surface layer. The PSQ treated rail material was tested in laboratory and field. Results showed that the microhardness of the PSQ treated rail material increased significantly and the tribological properties were improved. Field test data indicated that the wear resistance of the switch rails is enhanced by approximately 3 times that of untreated rail materials [500]. The rolling contact fatigue (RCF) resistance of two ductile iron variants, chilled ductile iron (CDI), and quenched and tempered ductile iron (Q&T DI), was evaluated, and compared with that of AISI 1080 steel [501]. It was reported that Q&T DI exhibits a much lower RCF loading capacity (2.1 GPa) compared with CDI (3.6 GPa). Under the same loading condition, CDI demonstrated a significantly lower RCF resistance compared with Q&T 1080 steel. Cryogenic treatment can also alter the crystal structure and mechanical properties of steels and alloys. Effects of cryogenic treatment on wear resistance of D2 steel [502], aCN/TiAlN, TiAlN, and ncTiAlSiN hard coatings deposited on the D2 steel substrate [503] and Mg–Y–Nd–Zr alloy [504] were investigated. The optimal cryogenic process resulted in enhanced wear resistance index, minimum compressive residual stresses, and optimum impact toughness for the D2 steel and the coatings. For the magnesium alloy, a twofold reduction in the volumetric wear, a threefold reduction in the linear wear, and a considerable reduction in static and kinetic coefficients of friction were found, compared to its initial state.

## 3.5 Additive surface engineering

To change the properties and topography of an object, a direct and effective approach is to add a layer of different materials or form a layer of new materials on the surface of the object, while the increment of the dimensions of the object is very slight. There exist a variety of such coating technologies nowadays. The coating can be processed at atmosphere, in vacuum or in solutions under controlled different temperatures and speeds physically or chemically, and the components of the materials to be added can be in forms of particles, clusters of ions and/or atoms, the chemicals of the coating to be formed by chemical reactions can be liquids, gaseous or plasma generated under a strong field. The thickness, density, interface adhesion strength, residual stress, and compatibility of physicochemical properties with substrate are major concerns for the quality of the additive coatings.

### *3.5.1 Electro-depositions*

Chromium and nickel electroplatings are most widely used electrodeposited hard coatings. Because of the hazardous constituents generated during Cr-plating, Ni-plating, with some compounds, has become the major in industry. Ni–P multilayers were electrodeposited on brass and iron substrates in electrolyte Niphos 967, and their hardness, anti-corrosion, and anti-wear properties were measured in laboratory. The multilayers with individual layer thickness between 6 and 1 μm showed improved wear resistance even compared to the crystalline single layers, due to the decreased tensile stress within the crystalline Ni–P layer [505]. Reinforcement effect of Ni–P coating by electro-co-deposition of $ZrO_2$–$TiO_2$ was reported [506]. The properties of Ni–Co–P multilayers with various Co/P ratio were tested and compared [507], and it was found that the coatings with submicron/ nanometric layers exhibited lower mass loss and COF in the range of 0.34–0.4 against a steel ball. The presence of a transfer layer on the ball worn surface with various oxides was confirmed, which was responsible to the lowered COF for multilayer Ni–Co–P coating, especially under higher loads. Electroless deposition of a Ni–P coating was successfully deposited on diamond particles used for cutting of silicon wafers, but residual stress produced by the coalescing of crystallites during the deposition resulted in failures of fracture and peeling-off [508]. Ni–W coatings were electrodeposited on piston ring used in engines and their friction and wear properties were compared with those of hard chrome plating widely used in current automotive engines. Results showed a close wear index values and lower friction coefficient for the Ni–40.6W coating annealed at 600 °C high temperature compared with hard chrome plating. After 50 h of operation in an engine, the Ni–W alloy piston ring had no cracks on the surface or the cross section of the coating, indicating a promiseful application in engine parts [509]. Co-electrodeposited Ni–W–B/B coatings were prepared on low-carbon steel [510]. Dry sliding experiments showed the influence of B contents and post-treatments at different temperatures on friction coefficient and wear resistance of the coatings. TaC particles were incorporated into Ni–W coating, which increases the hardness, and results in a low friction coefficient (~ 0.1) in dry sliding after UNSM treatment [511]. Ni–Mo amorphous coatings were electrodeposited on 304 stainless steel in sulfate–citrate acid solutions [512], and wear tests showed an optimal Mo content of 16%. Ni–$MoS_2$ coatings were electrodeposited on Ni substrate in a mixture electrolyte of nickel sulfate + nickel chloride ($NiCl_2 \cdot 6H_2O$) + boric acid and a small amount of surfactant. A manifestation of Hall–Petch breakdown was found in the coating [513]. In Ref. [514], novel

Ni–B–TiN metal matrix composite (MMC) coatings were successfully deposited using a nickel Watts bath containing TiN nanoparticles by pulse electrodeposition (PED) method. The addition of TiN reinforcing particles to the Ni–B alloy matrix increased the anti-corrosion efficiency of the material by filling the gaps in the coating as well as the hardness and wear resistance. The mechanical and wear properties of the electrodeposited Fe–W/$Al_2O_3$ composite coatings, especially after annealing, were investigated, showing superior to the properties of hard chromium coatings [515].

#### 3.5.2 *Vacuum depositions*

Various kinds of wear and corrosion resistant coatings can be deposited on machine components at relative low temperatures with different PVD techniques. TiN and TiSiN multilayers are typical wear resistant coatings widely applied using PVD on manufacturing tools. Marchin and Ashrafizadeh [516] investigated the effect of carbon addition on tribological performance of TiSiN coatings produced by cathodic arc physical vapour deposition. Ball-on-disc wear tests indicated lower coefficient of friction of the coatings against a $ZrO_2$ ball as compared to the steel substrate; the lowest value of 0.2 was experienced by TiSiCN coated surface. The application of the coatings changed the prevailing wear mode from adhesive to abrasive, decreasing the wear rate to one third. Addition of carbon to TiSiN changed tribological behaviour of the coating, similar to a self-lubricted film. Field tests of TiSiCN coating on forming dies, for deep drawing operation of steel tubes, revealed 10 times increase of die lifetime. CrSiN/Cr multilayer coatings on Ti alloy by inserting ductile Cr layers were prepared by PVD, and tested in laboratory [517]. The results showed the Cr/CrSiN-10 multi-layer coating exhibited higher adhesive strength, and low friction coefficient (~0.44) and wear rate ($4.1\times10^{-10}$ $mm^3/(N\cdot m)$) due to the reduced probability of coating peels. The Cr/CrSiN-10 multilayer coating also exhibited lower erosion wear rate (0.05 $\mu m\cdot s^{-1}$) compared with Cr/CrSiN-1 coating (0.15 $\mu m\cdot s^{-1}$). Hard coatings of ZrC [518], ZrN [519], and $Ta_{1-x}W_xC_y$ [520] films were respectively deposited by sputtering. The ZrC nanoceramic coating showed higher corrosion resistance than the 316 stainless steel under cavitation erosion condition. Significant mitigation of fretting wear on samples with ZrN coatings has been demonstrated. Both low average friction coefficient of 0.28 and wear rate of $1.12 \times 10^{-6}$ $mm^3/(N\cdot m)$ was obtained for the $Ta_{0.69}W_{0.31}C_{0.75}$ film, which is consistent well with its super mechanical properties. Not only hard and wear resistant films but also soft solid lubricating films of transition metal dichalcogenides (TMD), including selenides, sulphides, tellurides of niobium, molybdenum, tungsten, etc., can be deposited by PVD. In Ref. [521], molybdenum oxide on AISI-316 steel was synthesized using cathodic cage plasma deposition at cathodic and floating potential. The samples treated at floating potential revealed better wear resistance and lowest/smooth friction coefficient. Mechanical and friction properties of sputtered Mo–Se–C film at low load were reported in Ref. [522]. Hardness, elastic modulus, hardness to elastic modulus ratio of the film was measured. The results showed that the ratio of hardness to elastic modulus increased with carbon content at the power two. These values decreased with increase in applied load except for very low load where slipping of indenter takes place. The friction coefficient and depth of scratch decreased with increase in carbon content up to 55 at% C. To develop good coatings operating in vacuum, Au–Ni multilayer films of varying interlayer spacing were deposited on cleaned Si wafer, and tested on an AFM and nanoindenter [523]. The results exhibited that there is a regime of high friction when the interlayer spacing is large and a regime of low friction when the spacing is small. The low friction regime is associated with a critical grain size below which grain boundary sliding is expected to be the dominant mechanism of deformation.

Organosilane films were synthesized on steel substrate 316 L by using PECVD, and characterized with Raman spectroscopy, XPS, infrared spectroscopy (IR), AFM, and profilometry [524]. The results showed that the films prepared by PECVD using hexamethyldisilane (HMDS) as a single precursor have high hardness, low surface roughness, and low friction coefficient. Textured alumina coatings were deposited on cutting tool by using CVD, and tested through machining experiments and ball-on-disc wear tests [525]. Workpiece materials containing hard particles cause wear of the coating mainly on the flank side of the tools, as expected. The ball bearing steel creates the most wear rate on the flank side due to its hard carbide particles. On the other hand, the stainless steel imposed the highest coating wear rate on the rake side of the tool.

*3.5.3 Friction surfacing (FS)*

FS is a solid-state joining process to coat a thin metal layer on similar or dissimilar substrates. In the FS process, a rotating consumable rod (referred to as "mechtrode") is pressed onto a metallic substrate causing the temperatures to rise because of friction-generated heat and the materials to be deformed at the contact surface. AISI 904L super austenitic stainless steel [526] and AISI 4340 steel [527] FS coatings were coated on a 304 ASS plate and a low carbon steel substrate respectively. Their microstructure, mechanical properties, and residual stresses were evaluated. Experimental investigation of FS was performed for varied process parameters and changes in substrate thickness as well as process environment, their influence on process temperature and deposit geometry was clarified [528]. In Ref. [529], the smoothed particle hydrodynamics method was applied to simulate the friction surfacing of aluminum alloys.

*3.5.4 Carbon films*

Owing to the tunability of carbon bond and doping compositions in a wide range, carbon films, covering from graphene, graphite, DLC to diamond, have been researched extensively for the past decades. The hardness and tribological properties of carbon films are adjustable, depending on coating technique and process parameters. Both CVD and PVD can be used for deposit carbon films. DLC coatings are mostly studied for various applications in dry and lubricated tribo-contacts. The effect of growth defects with irregular columnar boundaries (GDICB) on the mechanical and tribological behavior of a tungsten carbide/carbon (a-C:H:W) multilayer coating was subjected to multiscale tribological tests, including macroscale reciprocating tests and microscale scratch tests. Samples consisted of a commercial a-C:H:W coating deposited onto polished AISI H13 discs and AISI 52100 steel balls. The macroscale reciprocating tests revealed a variable GDICB mechanical behavior, including deformation and detachment of those regions inside the wear track in the beginning of the test. The microscale scratch test results indicated a load-dependent mechanical behavior of the GDICB, showing a transition from deformation to detachment mechanism with the increase in the normal load [530]. Friction and wear behaviors of bare and DLC/chromium (DLC/Cr bi-layer) coated SKH51 steel were assessed at low temperatures. The results revealed a significant increase in the wear rate of SKH51 due to changes in wear mechanism and the degradation of mechanical properties as the temperature decreased. The DLC/Cr bi-layer coating significantly reduced the wear of SKH51, particularly at low temperatures. The formation of a thin layer of frozen water at subzero temperatures played a major role in determining the wear behaviors of the specimens [531]. In Ref. [532], influence of Si- and W-doping on micro-scale reciprocating wear and impact performance of DLC coatings on hardened steel was performed. Micro-tribotests showed the lowest resistance to repetitive impact of the Si-doped DLC, whereas the W-doped DLC was the softest coating studied and had low wear resistance in reciprocating sliding, it was significantly more damage tolerant to repetitive impacting than the other coatings despite its low hardness and low wear resistance in reciprocating tests. A novel a-C/(WC/a-C) film compositing of a-C nano-layers and super-latticed WC/a-C nano-multilayers was fabricated on M50NiL substrate by an unbalanced magnetron sputtering system [533]. Tribology test at high temperatures showed that the a-C/ (WC/a-C) film has effectively overcome the poor tribological performance of a-C film at high temperatures (200 °C). While benefiting from the good toughness of a-C sublayers, the a-C/(WC/a-C) film showed significant better wear resistance than the WC/a-C film at the temperatures of 25–200 °C. AFM experiments indicated that the friction and wear behaviors of DLC film are not only related to the friction pairs but also related to the normal loads [534]. The self-lubricating, wear-resistance, anticorrosion, and antifouling properties of Ti/(Cu, $MoS_2$)-DLC coating in marine environment were tailored by controlling the content of Cu dopant [535].

The interplay between surface topography, DLC film and lubricating oils attracted a lot attention. Tribology tests showed that the strongest effect of the orientation of the topographic directions occurs in dry sliding DLC vs DLC contacts. A super-lubricious DLC surface layer was efficiently formed, exhibiting low friction coefficients of 0.04 for smooth surfaces, and rough surfaces with grooves oriented along the sliding direction. The process of surface layer formation was most severely disturbed for average surfaces roughness with grooves oriented at 45° to the sliding direction. In dry sliding steel contacts, the coefficient of friction decreased with increasing surface roughness. On the other hand, the coefficient of friction increased with surface roughness in oil lubricated steel contacts. Adding a synthetic oil into the DLC vs DLC sliding contact prevented the formation of the super-lubricious surface layer. In tests with rotational steel vs steel sliding the friction coefficient was about 10% lower than in test with linear reciprocal sliding but was higher in DLC vs DLC contacts [536]. The differences in the roles DLC film played in dry and lubricated conditions are illustrated in Fig. 34. Tribofilm formations on DLC film during sliding were investigated *in-situ* [537], and *ex-situ* [538]. Performance of DLC coatings in piston ring-cylinder bore was investigated in Refs. [539, 540]. Application of DLC film in methane environment [541] and the effect of DLC film on preventing ingress of hydrogen into bearing steel [542], durability and robustness of DLC coating for anti-icing in aircraft [543], and feasibility of DLC film as a self-lubricated dielectric coating [544] were also investigated. Design and fabrication of WC/DLC multilayer coatings with optimized interface characteristics and superior surface protection were proposed by Nemati et al [545]. A new DLC surface treatment method was developed with electric discharge for short running-in and friction reduction [546].

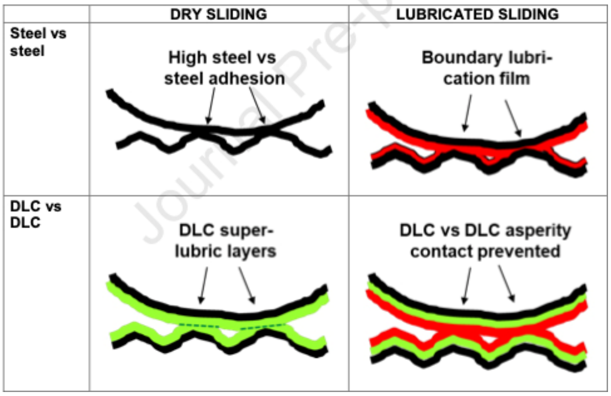

**Fig. 34** Illustrative differences between steel vs steel and DCL coated vs DLC contacts under dry and lubricated sliding conditions [536]. Reproduced with permission from Ref. [536], © Elsevier B.V., 2021.

Besides DLC, graphite-like film [547] and mirror-like surface finish ultrananocrystalline diamond films on tungsten carbide [548] were reported respectively. In addition, micro-textured diamond/$WS_x$ coatings were prepared by inductively coupled plasma etching techniques (ICP) on the silicon substrate, and followed by hot filament chemical vapor deposition (HFCVD) [549].

### 3.5.5 *Nitriding, carburizing, and other additive surface engineering technologies*

Nitriding and carburizing are surface hardening technologies widely used in industry. Carburizing and nitriding duplex treatments were carried out for 10 h at various nitriding temperatures in the range of 460–540 °C on M50NiL steel [550]. With increasing the nitriding temperature, the magnitude of the residual compressive stresses decreases, the surface roughness increases, and the proportion of $\gamma'$ phase increases. The results of the wear tests carried out at different loads demonstrate that the wear mechanism controlling the wear rate transits from oxidative wear to abrasive wear when the load increases. The sample nitrided at 500 °C shows the best wear resistance to abrasive wear under a high load. Tribological properties of medium carbon steel changed after a treatment of anodic plasma electrolytic nitrocarburising in a carbamide electrolyte [551]. The minimum wear rate was achieved after nitrocarburising of steel at 750 °C for 7 min and a sliding speed of 0.4–0.55 m/s. Anodic nitrocarburising increased the wear resistance of steel by 40% compared to quenching at a sliding speed of 0.9 m/s. The wear process in nitrocarburised samples was followed by plastic displacement of the metal in the friction contact zone according to the molecular-mechanical and fatigue theory of wear. Nitriding of industrially-pure titanium VT1-0 in the plasma formed by continuous electron beam created by a fore-vacuum plasma-cathode electron source was tested at different temperatures of the titanium sample surface (700–1,020 °C) and electron beam energy (4–6 keV), in medium vacuum (5 Pa) of nitrogen [552]. The results showed that the depth of nitrided layer, Vickers microhardness, and wear resistance grew with sample temperature and beam energy. Medium and high boron electroless nickel coatings were nitrided, and their tribological properties were characterized [553]. Results indicated that the highest microhardness value was achieved for the plasma nitrided high boron coating being equal 1459 HV0.1. The measured mean wear rate and coefficient of friction values of the plasma nitrided coatings were more than 14 and 2 times lower than that of the heat treated ones, respectively. Experiment results showed that hollow cathode assisted active screen plasma nitriding of titanium can produce titanium nitride surfaces with thicknesses far exceeding those typical in PVD coatings. Moreover, higher plasma processing intensities can generate same layer thicknesses at lower temperatures, minimizing the deleterious effect on grain size growth [554].

Research papers on other surface engineering technologies and applications include thermally sprayed coatings [555], gaseous surface hardening of Ti–6Al–4V fabricated by selective laser melting [556], ceramic based self-lubricating coating fabricated on plasma electrolytic oxidation (PEO) ceramic coating [557], laser surface alloying of a commercially pure copper with NiTi powder [558], manganese phosphate conversion coatings with different crystal sizes on AISI 52100 steel [559], high velocity oxy-fuel sprayed Inconel 625 coatings [560], thermo-reactive diffusion (TRD) Cr−V−C composite coatings grown on ductile iron [561] and liquid-phase hydrothermal deposition of $MoS_2$ into TiAl coatings on Ti6Al4V alloy [562].

### 3.6 New antiwear materials and friction materials

#### *3.6.1 High entropy alloys (HEAs)*

HEAs are a new class of alloys reported firstly in 2002, which are composed of at least five principal elements with the concentration of each element between 5 and 35 at% but having simple crystal structures. Because of the effects of high entropy, severe lattice distortion, sluggish diffusion, and the cocktail, HEAs exhibit distinct and excellent mechanical properties, corrosion resistance, and high-temperature softening resistance compared to pure metals and traditional alloys. The number of research papers on tribological properties of HEAs is growing fast in recent years. Numerous studies have reported improved microhardness and wear resistance by selectively adjusting multiple elements in HEAs (Fig. 35) [563]. Deng et al. [564] investigated the effects of normal load and velocity on the dry sliding tribological behaviour of $CoCrFeNiMo_{0.2}$ high entropy alloy. They found that increase in load led to decrease of both friction coefficient and specific wear rate, while increase in velocity resulted in decrease of friction coefficient but increase of specific wear rate. Geng et al. [565] measured tribological performances of CoCrFeNiAl high entropy alloy matrix solid-lubricating composites over a wide temperature range, and compared with those of Inconel high temperature materials. The results showed that the HEA without solid lubricant (HEAM) exhibited superior hardness over the temperature range from room temperature up to 800 °C compared with the HEA composited with 5 wt% h-BN (BNHC), composited with 5 wt% h-BN + 5 wt% Ag (HAHC), Inconel 718 superalloy and austenitic alloy, while the HAHC showed the lowest friction coefficient and wear depth. The atmospheric plasma sprayed AlCoCrFeNi high entropy alloy coatings composited with Ag and $BaF_2/CaF_2$ eutectic self-lubricating materials have also shown excellent wear-resistance, especially at high temperatures [566]. Addition of Si element to AlCoCrFeNi HEA [567], Ti element to AlCoCrFeNi HEA [568] or increase in the ratio of B/Si in the laser cladded $Fe_{25}Co_{25}Ni_{25}(B_xSi_{1-x})_{25}$ [569], reduced the friction coefficient and wear rate. Laser cladding HEA coatings of ternary CrNiTi on pure titanium [570], and nano-lamellar $Ni_{1.5}CrCoFe_{0.5}Mo_{0.1}Nb_x$ on stainless steel [571] were also reported. Other HEA coatings of CrNbSiTiZr by radio-frequency magnetron sputtering [572], of $Fe_{17}Cr_2Ni_{0.2}C$ by thermal spraying [573], of AlxCoCrFeNiSi by atmospheric plasma spraying and laser re-melting were fabricated and

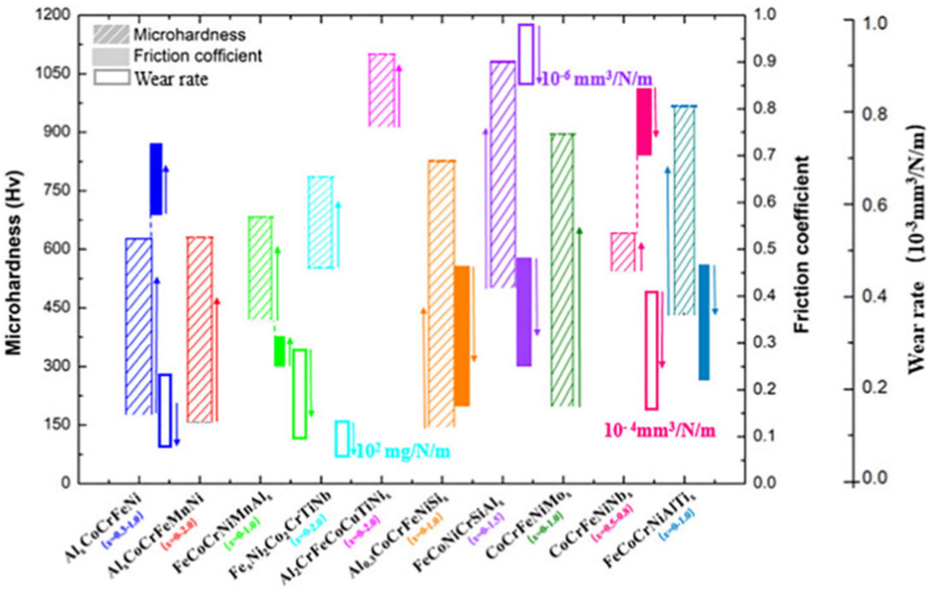

**Fig. 35** Microhardness, friction coefficient, and wear rate of different HEAs [563]. Reproduced with permission from Ref. [563], © Elsevier B.V., 2020.

characterized. Nitriding treatment of AlCrNbSiTiMo HEA coatings [574], boronizing treatment of Al0.1CoCrFeNi [575], and aging treatment of Co1.5CrFeNi1.5Ti0.5 [576] impoved the mechanical and tribological properties. Molecular dynamics simulation was used to study on the nanotribological behaviors of CrMnFeCoNi and CrFeCoNi HEAs under different conditions [577].

### *3.6.2 Metallic glasses (MGs) and superelastic metallic materials*

MGs are a relative new class of metals developed in the past four decades. Owing to the lack of long-range order, the absence of grain boundary, and in many cases, the retention of high alloying content in homogenous solid solution, MGs are of large elastic limit, high strength, excellent wear resistance, and corrosion resistance. MGs can be either casted into ingot as bulk metallic glasses (BMGs) or coated on objects as thin film metallic glasses (TFMGs). Tribological behaviors of both types of MGs have been investigated extensively in recent years. Effect of environmental oxygen concentration of the $Zr_{44}Cu_{10}Ni_{10}Ti_{11}Be_{25}$ MG were investigated [578]. Remarkably low and predictable friction was found when both oxidation and frictional heating were suppressed. Oxidation and the formation of a mixed metal-oxide layer increased the friction coefficient while significantly reducing wear rates, which ranged from values comparable to highly wear resistant materials like polymer and 2D solid lubricant nanocomposites to those that are found with soft, pure, high-wear metals, depending on the environment. These results revealed that the competition between material removal and the deformation-induced mixing of oxide particles can dramatically reduce wear rates, suggesting opportunities for optimization of tribological performance. Tribological properties of Pd-, Pt-, and Zr-based BMGs were compared under unlubricated conditions [579], showing that BMG-Pt exhibits a significantly higher COF (0.58 ± 0.08) when compared with BMG-Pd (0.30 ± 0.02) and BMG-Zr (0.20 ± 0.03). The effect of Fe addition on the structure, mechanical, and wear properties of Zr–Cu–Ni–Al BMGs was systematically investigated [580]. The results showed that a remarkable enhancement in plasticity occurred in the Zr58.6Cu18Ni10.4Al8Fe5 BMG, although compression strength and hardness did not change much. Hafnium-based alloys of two similar compositions were prepared by arc melting and suction casting to produce an amorphous alloy (Hf48Cu29Ni10Al13) and a crystalline one (Hf58Cu20Ni16Ti6) [581]. Pin-on-disc wear tests showed that there were no statistically significant differences in friction coefficient or wear-rates between the two materials. Four different BMGs have been studied in pure sliding and reciprocating contact conditions under two different initial contact pressures [582]. It is found that both friction and wear properties of these BMGs are quite different and scattered even for the same kind of BMGs (see Fig. 36). The wear, corrosion, and tribocorrosion resistance of ZrCuNiAl TFMGs were investigated by examining samples of similar global composition but with either homogenous or heterogeneous local composition [583]. The homogenous samples exhibited lower wear rates and friction coefficients than their heterogenous counterparts due to their higher hardness and lower stiffness. The heterogeneous samples exhibited higher corrosion pitting resistance than their homogenous counterparts by forming a protective passive layer with lower defect density.

The shape memory alloy NiTi possesses superior wear resistance owing to its superelasticity. The difference in wear behavior of superelastic NiTi and conventional Ti6Al4V alloys with similar elastic limits was investigated using reciprocating dry sliding ball-on-disc wear tests [584]. The wear resistance of the NiTi alloy showed up to 40 times higher than that of the Ti6Al4V alloy. Such a significant difference cannot be attributed only to the relatively small difference in hardness (*H*) or *H*/*E* ratios (*E* 1⁄4 elastic modulus) of the materials but, rather, to a difference in strain energy, which dissipates in the material and results from normal load of the sliding ball. NiTi superelastic archwire was used as the counterpart of a disc in a tribometer under the actual force magnitudes that the archwire receives during treatment and for different stress levels [585]. The results showed that the martensitic transformation induced by stress caused an increase in the coefficient of friction and in the wear rate by 50% and 30% respectively compared to the non-bent archwire.

### 3.6.3 *3D printed materials and sintered materials*

3D printing is an emerging manufacturing technology. Various kinds of polymeric materials and a few kinds of alloys can be printed into 3D mechanical components. Hardness, friction, and wear properties of poly lactic acid (PLA) polymer printed using fused deposition modelling (FDM) method [586] and 3D printed acrylonitrile butadiene styrene polymer [587] were characterized respectively. Effects of 3D-printing parameters and surface roughness on tribological behaviors were revealed. Laser powder bed fusion (LPBF) technique are used to print 316L stainless steel [588], 15-5 precipitation hardening stainless steel [589], 17-4 PH stainless steel [590], 316SS /bronze composites [591], and titanium alloy Ti–6Al–4V [592], and their behaviors of friction, abrasive wear, anticorrosion, and cavitation erosion were characterized.

Metallic powders are usually sintered into bearing parts with pregnant solid and/or liquid lubricants, referred to as powder metallurgy (P/M). The tribological behaviors of spark plasma sintered (SPS) niobium carbide [593] and nickel-aluminum bronze by P/M [594] method were characterized respectively.

### 3.6.4 *New polymer composites*

Compositing is the effective and major approach to

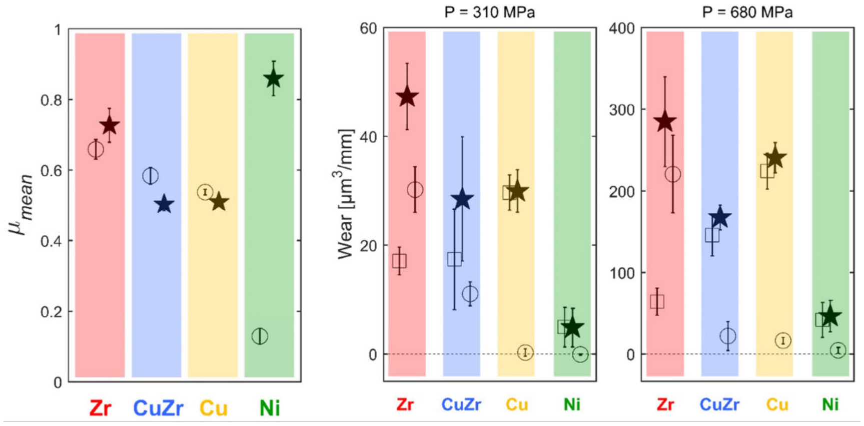

**Fig. 36** Mean friction coefficients and wear index of the tested four BMGs [582]. Reproduced with permission from Ref. [582], © Elsevier Ltd., 2020.

enhance the stiffness, strength, and wear resistance of polymers. UHMWPE composited with mulberry-like carbon spheres decorated with UiO-66-NH2 [595], boron phenolic resin-based composites using fluorine rubber micro powder as high-performance additive [596], graphene nano-plates (GNPs) reinforced basalt-epoxy multiscale composites [597], PTFE composites with different fillers [598], PAEK based composites [599], PA 66 polymer composite [600], short glass fiber reinforced PBT composites [601], and epoxy-based nanocomposites with graphene nanoplatelets–montmorillonite hybrid system [602] were prepared and characterized, respectively.

#### *3.6.5 Brake and friction materials*

Bakes are used to decelerate a moving vehicle or running/rotating parts in machine by dissipating and absorbing kinetic energy of the moving body with frictional work, most part of which transferred to heat finally. Brake materials are demanded to provide high and stable friction coefficient during interface slipping or sliding for a long operation period with low wear rate and high heat resistance. The high safety requirement of high speed railway transportation systems could not be guaranteed without reliable brakes. Friction blocks, with different additives including Cu-based powder metallurgy material, composite material, and a Mn–Cu damping alloy in high-speed train brake systems were tested and compared in terms of wear resistance and damping of friction-induced vibration and noise [603–605]. The results showed that the Mn–Cu damping alloy can suppress the friction-induced vibration, resulting in the lowest level of brake noise among all brake systems. However, the original friction block (i.e., the block without any filling material) can trap wear debris because of its perforated structure. The noise performance of the composite material block is superior to the noise performance of the block without any filling material (i.e., the original block) and the block with powder metallurgy material but inferior to that of the block filled with the Mn–Cu damping alloy. Cu-based brake pad [606], full-carbon/ ceramic braking pairs [607], and the P/M pad mated with C/C–SiC disc [608] for high speed trains were tested separately, and compared under same test conditions [609]. The cupper-free brake materials used in automobiles were also extensively tested for replacing traditional cupper-containing brake materials [610–613]. The airborne brake wear particulate emission problem was paid much attention [614–616]. Heat dissipation [617] and squeals [618] are also major concerns of brake performance. High friction brake shoe material was prepared for emergency braking in ultra-deep coal mine hoist [619].

For friction clutches, frictional torque limiters, and other frictional torque transmission parts, slipping is intermittent and transient event and thus sliding wear is mild compared to that in brake systems. Besides of dry friction, paper-based wet friction materials are widely used in automatic transmission systems. Friction and wear properties of paper-based composite friction material against 65Mn steel was investigated [620], and effects of the addition of rare earth on properties of paper-based friction materials was reported [621].

## 4 Biotribology

### 4.1 Introduction of biotribology

The research in the area of biotribology was immense and vast, covering a number of fundamental mechanisms and a wide range of practical applications in 2020–2021. Two search strategies were employed to identify the relevant references for review. The first one was based on the identification of the main journals in tribology, similar to other sections adopted in this manuscript and then selection of relevant papers related to medical and biological applications of tribological principles. The second one was based on the search in Web of Science using key words similar to a previous study listed in Table 1 [1]. In both cases, only papers published and accepted in 2020–2021 were included.

There were approximately 69 papers found from the first search, while a significantly more number of papers were returned from the second search (approximately 862). It was interesting to note the majority of biotriboloy papers were published in non-traditional tribology journals, implying the emphasis on applications and interdisciplinary nature.

**Table 1** Keywords and combinations for search in Web of Science, modified/taken from Ref. [1]. Reproduced with permission from Ref. [1], © The author(s), 2020.

| System | Keyword and combination | |
|---|---|---|
| | Medical and biological | Tribological |
| Joint | Articular cartilage<br>Synovial fluid or mucin<br>Joint and implant or prosthesis | Lubrication or friction or wear or tribology |
| Skin | Finger<br>Tatctile perception<br>Textile or fabric<br>Cosmetic<br>Prosthesis or implant | Skin and tribology or friction or wear |
| Oral | Enamel<br>Orthodontic | (Teeth or dental) lubrication or friction or wear or tribology |
| | Dental and implant<br>Saliva<br>Toungue<br>Oral perception | Lubrication or friction or wear or tribology |
| Other | Medical implant<br>Hair<br>Eye or contact lens<br>Vegetable oil | Lubrication or friction or wear or tribology |
| | Tissue | Cell and (tribology or friction or lubrication) |
| | Plasma protein<br>Gastric mucus | Lubrication |
| | Gecko<br>Pangolin<br>Fish<br>Shark<br>Bird<br>Water strider<br>Earthworm<br>Ants<br>Beetle<br>Butterfly<br>Seashell<br>Snail | Friction or tribology |

Therefore, the following analysis was mainly based on the number of studies from the second search, with additional studies from the first search. A density map and a network map, constructed from the keywords of research areas, were used to illustrated the focuses of these studies and the relationships (https://www.vosviewer.com/documentation/Manual_VOSviewer_1.6.5.pdf). The colour of each point in the density map indicated the density of that item, with red and blue showing the large and the small numbers in the neighborhood. The label and the size of the circle in the density map indicated the weight, with the colors and the lines showing the relevant cluster and relatedness respectively.

A wide range of topics were covered as analysed below. Figure 37 shows the number of references in different areas and number of review papers in the main area of research of biotribology.

Figure 38 shows the density map and the network map constructed from the keyword of biotribology. It is clear from Fig. 38 that the main areas of research in biotribology included synovial joints and artificial replacements, oral and skin, similar to those found in the previous review in 1999–2020 (Table 1). In addition, an increasing number of references was found in biolubricant.

The specific studies in the above areas were then analysed separately in details as follows.

### 4.2 Natural synovial joints and artificial joint replacements

Joint tribology covered both natural and artificial replacements as illustrated in Fig. 39 for the density and the network respectively. Both healthy and diseased natural joints (fluid, cartilage, osteoarthritis, etc.) as well replacement materials (hydrogel, uhmwpe, etc.) were covered, with respect to tribological studies (contact, wear, lubrication, etc.). The main findings were presented below in two aspects: Lubrication of natural synovial joints and cartilage repair/regeneration, and biotribology of total artificial joint replacements.

The lubrication mechanism in the natural synovial joints focused on the bearing surface of articular cartilage, the lubricant of synovial fluid and the effects with different treatment options [622]. Healthy, degenerated and diseased cartilages were considered as well as the biomaterials in articulations with these natural tissues and tissue engineered cartilages for

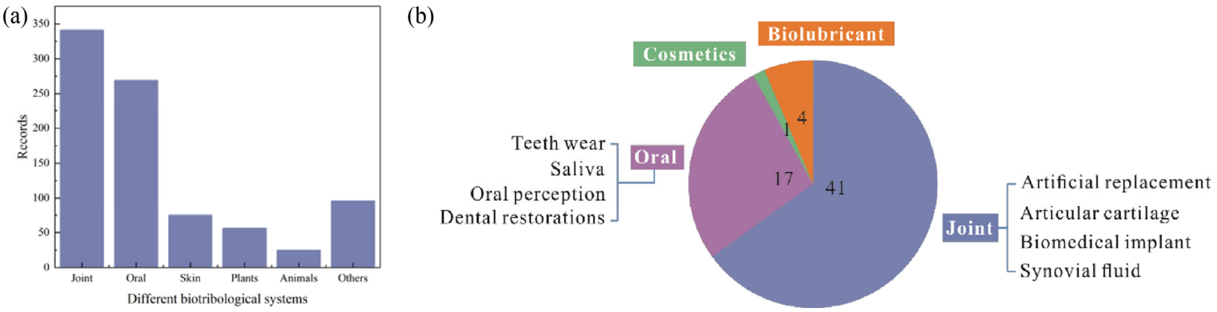

**Fig. 37** (a) Number of references and (b) review papers in different areas of biotribology.

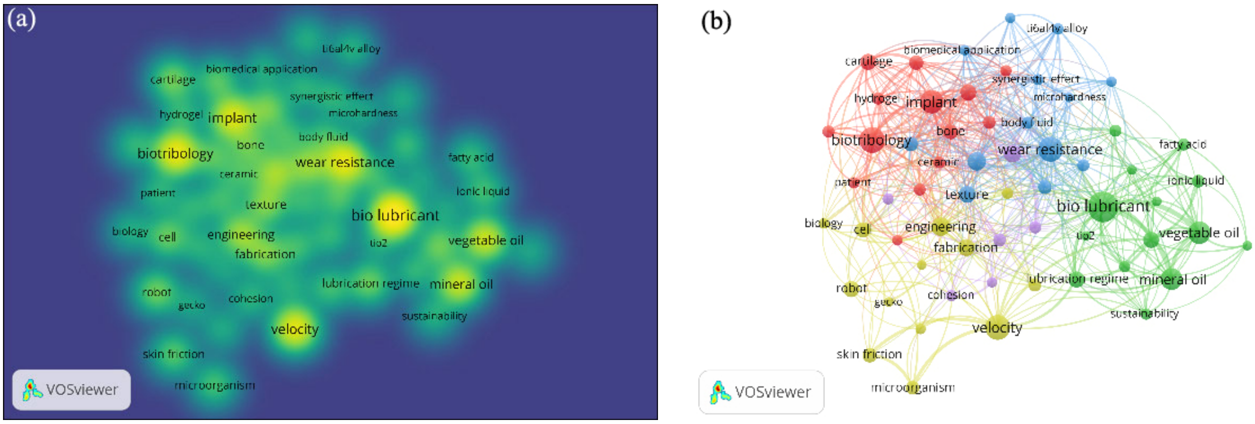

**Fig. 38** (a) Density and (b) network diagrammes of biotribology.

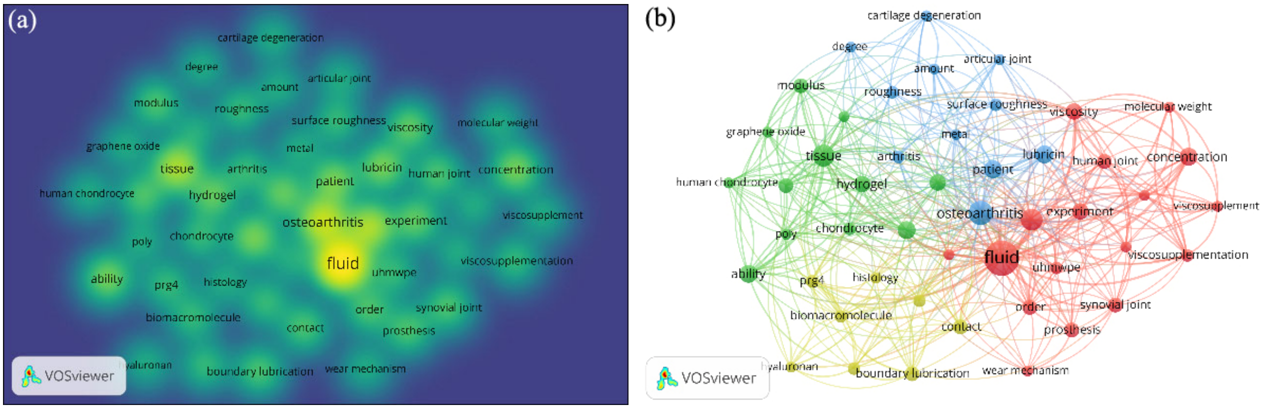

**Fig. 39** (a) Density and (b) network diagrammes of joints.

repair and regeneration in partial/hemi replacements. Most of these studies did not consider the living nature and live chondrocyte cells of articular cartilage addressed in Ref. [623]. Furthermore, a single, often simplified, loading and motion were applied, while the importance of considering more realistic combined inputs was recognized [624]. A number of studies discussed in this section investigated cartilage and synovial fluid separately, often with simplified configurations of cartilage samples which may not truly reflect the lubrication mechanism in the whole synovial joint system. For example, the viscosity of hyaluronic acid solutions was significantly increased in contact with cartilage compared with steel [625]. A custom-made whole joint system with realistic loading and motion inputs [626] or using robotics [627] was developed for evaluating potential clinical interventions of osteochondral repair. The tribological approach mainly focused on lubrication mechanisms generally through friction measurements. However, wear mechanisms and cartilage damage were equally important. Delamination of the articular surface, starting in the middle zone, was found in a reciprocal sliding of tibial cartilage strips against glass or cartilage, with immature cartilage showing significant less cycles [628]. A direct visualization of lubricating film and simultaneous measurement of the friction coefficient were developed in a novel setup [629]. Most of these studies were conducted experimentally, however analytical and computational approaches were also increasingly developed. A theoretical lubrication model for compliant-poroelastic cartilage-on-cartilage line contacts was developed [630]. An atomistic model for top layer cartilage–cartilage contact in unhydrated conditions was also attempted using molecular dynamics (MD) simulations [631]. Although a synergistic combination of different lubrication modes existed in the effective tribological performance of synovial joints such as hydrodynamic and biphasic [632], the current focus was on the boundary lubrication mechanism and the development of treatment options restoring this mechanism. The boundary lubrication mechanism was attributed to a combination of phosphatidylcholine lipids, hyaluronic acid, and lubricin, resulting in an effective hydration lubrication, with a number of important suggestions for future research [622]. A superficial free surface layer rich in glycoconjugates on bovine cartilage was directly observed with high resolution SEM and AFM [633]. The lubrication was depleted with increased friction in cartilage specimens with enhanced calcium centration which degraded the surface layer [634]. Lubrication of degraded cartilage with chondroitinase ABC was shown to be restored through re-establishing lamina splendens with a bioinspired mucoadhesive biopolymer chitosan catechol [635]. Furthermore, different tribological characteristics of articular cartilages were found in different joints and on different sites, implying the importance of physiological loading and motion input into the joints [636], in alternated biomechanical environments [637] and in joint injuries [638]. The importance of the understanding the lubrication mechanism was clearly recognized for the future development of tissue engineered cartilage [639] and alternative replacement materials such as hydrogels [640]. The role of tribological inputs and evaluation was considered in tissue engineered cartilages [641–645]. Different replacement biomaterials were shown to cause different levels of damage on cartilage in hemi-arthroplasty [646], polycarbonate urethane polymer being the best and ceramic better than CoCr. A number of studies were devoted to the restoration of the lubrication mechanism and development of bioinspired hydrogels mimicking the biphasic properties of cartilage with brush layers. The importance of the migrating contact in biphasic natural cartilage was shown to be equally important in hydrogels [647]. A number of bioinspired novel biomaterials and lubricants were developed with enhanced lubrication. A similar adaptive lubrication mechanism was observed in a novel thermo-responsive layered material, fabricated by grafting a poly(3-sulfopropylmethacrylate potassium salt) polyelectrolyte brush onto the subsurface of an initiator-embedded high strength hydrogel [poly(nisopropylacrylamide-co-acrylic acid-co-initiator/$Fe^{3+}$)] [P(NIPAAm-AA-iBr/$Fe^{3+}$)] [648]. A hydrogel-elastomer hybrid surface was fabricated with enhanced mechanical properties and lubrication [649]. Multilayer hydrogel material was grafted on ultra high molecular weight polyethylene (UHMWPE) substrate to simulate a bone–cartilage layered structure with improved lubrication [650]. Macroscale superlubricity was achieved between zwitterionic copolymer hydrogel and sapphire in water [651]. A novel thermoplastic polyurethane elastomer derivative filament for 3D printing was developed for cartilage tissue engineering [652]. In addition to these scaffolds and bearing surfaces, the role of synovial fluid was also considered [653]. Mesoporous silica nanoparticles grafted with sulfobetaine-based zwitterionic polymer were introduced with enhanced lubrication in a polytetrafluoroethylene sphere-in-a Ti6Al4V plate contact [654]. The rheological and frictional measurements of commercially available solutions for viscosupplementation were conducted in rotational rheometers in cone-plate and plate–plate configurations and a pin-on-plate tribometer using PVA hydrogel as a model of articular cartilage and the glass [655], with no significant benefits determined.

The research in biotribology of total artificial joint replacements ranged from optimization of biomaterials and implant designs to improving clinical functions [656]. Wear of the bearing surfaces remained one of the key issues, in terms of wear and wear debris induced biological reactions. Tribological approaches generally consisted of wear studies through laboratory and clinical means, while lubrication was mainly investigated through computational methods [657]. Wear of the contact surfaces was important, as well as the characterization of wear debris with novel imaging techniques [658]. A new wear measurement technique was developed through a chemical means [659], therefore capable of differentiating the top and the bottom wear of the tibial insert in the knee implant. In some studies, friction was measured, mainly for the purpose of understanding the wear and the lubrication mechanisms [660]. A fluorescent microscopy method was adopted to measure experimentally the lubricant film thickness and investigate the effects of synovial constituents [661]. Direct measurement of lubricant film thickness and validation of lubrication models were carried out for both the hip [662] and the knee [663]. Wear modelling was increasingly developed and refined for the hip [664, 665] and for the knee [666]. The advantage of computational wear modelling allowed the potential of integrated studies; for example, combination of wear and lubrication modelling, and coupling of tribological and biomechanics etc. towards more realistic applications. The advantage of the computational modelling was shown in parametric analyses for the design parameters of the hip [667] and the loading/motion in the knee [668]. Further applications of the computational modelling were made to investigate the effect of different daily activities of the hip [669] and wear of the lateral and medial compartments in the knee [670]. A wear model with the consideration of lubrication was developed in Ref. [671]. In addition to these in vitro laboratory and in silico modelling studies, instrumented prostheses with various sensors were developed [672]. For example, such a technique allowed the measurement of in-vivo friction and temperature rise, showing the effect of the surgical cup placement on the heating up of total joint hip replacements [673]. Other in vivo methods were also applied, including using acoustic emission for the early detection of the failure modes in hip implants [674]. With the advancement of these in vitro and in vivo methodologies, it was possible to investigate more realistic clinical problems. Importance of biomechanical inputs in terms of loading and motion was investigated for developing more realistic testing methodologies [675] or considering more realistic activities and extreme worse case scenario [676]. Implant position such as the knee was also shown to influence wear greatly [677]. Suboptimal neuromuscular control was shown to influence the loading and consequently wear greatly [678]. Small wear of ceramic-on-ceramic combinations with alumina matrix composite was found in the presence of third-body ceramic particles and under subluxation stresses [679]. Furthermore, adverse loading was shown to affect the tribo-corrosion of metal-on-metal hip implants [680]. Traditional biomaterials such as UHMWPEs in articulation against cobalt chromium alloys and ceramics as well as new potential biomaterials such as hydrogels and PEEK were investigated. Wear of artificial hip and knee implants with highly crossed-linked polyethelene was evaluated [681–683]. A new non-oxide silicon nitride $Si_3N_4$ against vitamin-E-diffused crosslinked polyethylene hip bearings was shown to be comparable to the gold standard oxide ceramic, zirconia ($ZrO_2$)-toughened alumina ($Al_2O_3$) [684]. Novel compliant polycarbonate urethane was demonstrated in a limited clinical and an experimental wear testing [685]. Friction and wear of alternative all polymer materials combinations were evaluated in Ref. [686]. Surface modifications of the traditional bearing surfaces included coating, texturing [687], and treatments, particularly when new technologies such as 3D printing was adopted. Coating of the knee implants was shown to be particularly important to reduce metallic ion release as demonstrated in a simulator study using titanium niobium nitride [688]. Potential applications of amorphous carbon coatings for both UHMWPE and CoCr in total knee replacements were demonstrated [689]. The potential problem of the coating substrates and manufacturing techniques remained an issue [690]. The fine microstructures and precipitates of laser-sintered Co–28Cr–6Mo alloy were attributed to a lower wear rate in a simulator study [691]. In addition to the wear at the main articulating bearing surfaces, tribology at other interfaces including the modular junctions (e.g. head and heck) between different biomaterials and the fixation with the bone. The fretting corrosion behaviours of three typical materials pairs were considered [692]. The fretting regime of $Al_2O_3$/Ti6Al4V and 316 L/Ti6Al4V pairs changed from slip regime to mixed fretting regime as the load increased, while the CoCrMo/Ti6Al4V pair was always in slip regime. Furthermore, the damage to the Ti6Al4V alloy was the smallest when paired with $Al_2O_3$, while it was more serious when paired with 316L and CoCrMo. Both the surface topography and pH levels influenced the tribo-corrosion in a CoCr–Ti contact, with machined groups exhibited increased the susceptibility to corrosion damage, and both polished and machined groups still exhibited a mechanically dominated degradation [693]. Alternative materials combinations were developed to reduce the fretting corrosion at the modular taper, including PEEK and polyethylene composites [694]. In addition to the modular junction, the tribological problems at the bone–implant interface were also evident. Friction and compliantness were shown to be important for the cementless fixation in the knee [695]. Laser textured Ti–6Al–4V alloy implants with bioinspired micro-overlapping structures were adopted to improve cell attachment, proliferation, and osteogenic differentiation as a result of the important role of friction [696]. Although most studies focused on the two main joints of the hip and the knee in the body as discussed above, other smaller joints were also received significant attention including the elbow [697], the wrist [68], the ankle [699], and the temporomandibular joint [700, 701]. The role of friction was considered in developing better robotics and exoskeletons [702] as well as neuroprosthesis [703]. It was also interesting to note the development of non-articulating compliant mechanisms with potential applications to the finger joint [704].

### 4.3 Oral

The density and the network for the tribological studies in oral tribology are shown in Fig. 40. Main keywords of research were identified as natural tissues of enamel, saliva, tongue, etc., biomaterials of zirconia etc., lubrication, wear, etc., as well as sensory sensory perception of mouthfeel, astringency, etc.

The wear mechanisms of both natural tooth and restoration biomaterials as well as tooth cleaning remained the major focus [705]. Despite a large number of wear studies in dental tribology, there was still a lack of standard testing methods. In order to tackle the complex tribological mechanism in oral environment, a number of new methodologies were developed, including friction [706], wear [707], etc. The wear mechanism of teeth was found to be a synergistic combination of acid-erosive and mechanical actions of attrition and abrasion [708]. Wear of different tribological pairs in dental tribology was investigated, including tooth–tooth, tooth–restorative material (tooth–ceramic, tooth–resin-based-materials, and tooth–metal), and restorative-restorative materials. The role of saliva in the wear of teeth was also recognized [709]. Both human and animal teeth were studied, particularly considering the effects of diets.

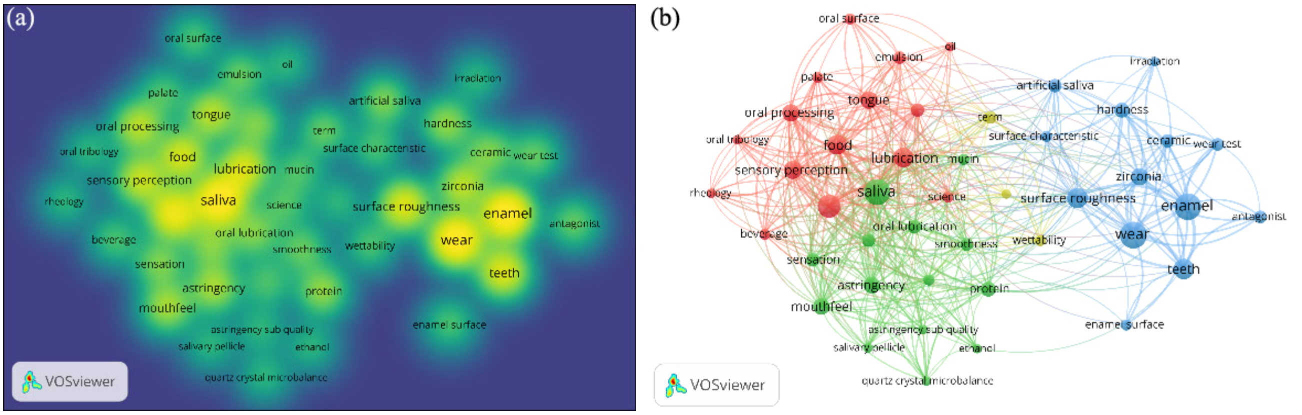

**Fig. 40** (a) Density and (b) network diagrammes of oral tribology.

The role of interfacial protein bonding was shown to be responsible for the wear mechanism of human tooth enamel [710]. A number of important factors on wear of dental enamel were identified, including remineralization [711], loading [712], pH of $H_2O_2$ solution [713], and tooth brushing [714]. Furthermore, the effect of desensitizing and whitening dentifrices on enamel loss was found to be negligible, compared with brushing with saliva [715]. The relationship between dental microwear textures and the diets of fossil mammals was reviewed in Ref. [716], in the context of evolution. Different biomaterials and technological were investigated in restorative dentistry using ceramics, metallic alloys, composite resins, and other polymers such as PEEK [717] and PEEK/PTFE coatings [718]. Surface texturing of 3Y-TZP zirconia ceramics was found to have a large effect on wear against $Si_3N_4$ ceramics and 304 stainless steels [719]. 3D printed zirconia, Ti6Al4V, and resin were all shown to be promising [720–722]. Similar to total artificial joints, both tribocorrosion and fretting corrosion were also found in dental implants [723]. Differences in the coefficients of friction were found between different materials combinations of pure titanium, titanium alloy, and yttria-stabilized zirconia for the taper joint of the dental implants, however, this did not cause a large change in the maximum von Mises stress in the constructs [724]. A chewing cycle loading was shown to be more appropriate to investigate the implant–abutment interface, as compared with a simple fatigue cycle [725]. The effects of connection type between abutments and implants and their friction on the fixation to bone and subsequent biomechanical and biological responses were examined [726]. In addition to these natural teeth and replacement materials, a number of studies were also carried out to develop better artificial saliva [727]. Furthermore, the role of tribology was increasingly recognized toward the study of food oral processing and sensory perception as reviewed in Refs. [728–730]. An artificial tongue substrate was developed, based on the topographic characteristics of human tongue and the morphological features of fungiform and filiform papillae and applied to investigate under different speeds and lubricants [731]. Two polyphenols were adopted to investigate the structure and lubrication of salivary pellicle, with a particular reference to astringency mechanism [732]. The effects of oral lubrication on satiety, satiation, and salivary biomarkers were investigated, as a potential novel strategy to reduce obesity [733]. The perception of a number of foods and drinks were investigated, including wine [734], milk [735], coffee [736], melted chocolate [737], etc. In addition to dental implants, tribological problems in other dental appliances also existed, including archwires and brackets [738], etc. Other interesting applications in oral tribology included evolution and archaeological studies [739].

### 4.4 Skin

The density and the network for skin tribology are shown in Fig. 41. Areas of focuses included skin friction, wear, and lubrication as well as the effect of wearing masks due to the current Covid-19 pandemic.

More and more sophisticated and complicated methodologies were developed to tackle the complex tribological problems of skin. These methodologies often combined different individual methods, such as

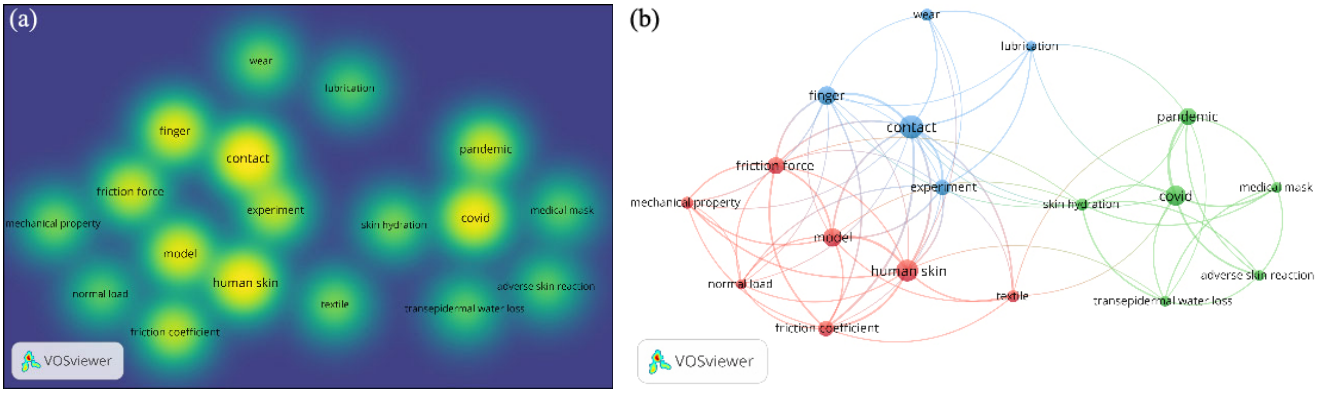

**Fig. 41** (a) Density and (b) network diagrammes of skin tribology.

friction and contact [740, 741], imaging (FTIR) and friction [742], and fluorescent imaging, contact, and lubrication [743]. Tactile perception remained as one of the hot topics in skin friction. Perception studies, including neuro pulses [744] and motor control [745] were developed alongside with friction-based studies. Time-dependent friction and vibration were recorded together with simultaneous visualization of the contact set-up and the contact area [746]. Novel sensors were developed for monitoring finger manipulation with minimal interference [747]. The stimulation of tactile mechanoreceptors was modelled to examine the effects of age-related morphological and mechanical skin changes [748]. Important considerations of skin included surface topography such as texture, roughness, coating, etc., structure (heterogenicity) and hardness. Different objects in contact with skin were considered with various materials and topographies, under appropriate loading and motion conditions in conjunction with the presence of appropriate environments. Finger pad friction was normalized due to personal and environmental differences [749]. The effect of sliding direction was shown to influence finger tactile perception, friction, and dynamics [750]. A wide range of applications were made, including in cosmetics [751], textile industry [752], virtual reality [753], and sports [754, 755]. The role of friction was examined in a brain–computer interface, for people with visual impairments [756] and limb reconstruction and comfort [757]. Novel artificial fingers were developed [758] and applied for robotics [759]. Novel sensors were developed for neuroprosthetics [760]. It was important to consider the effect of wearing protective respirators, medical, and fabric masks to prevent Covid-19 on skin contact, sweating, moisture, and friction [761].

### 4.5 Others

In addition to the tribological problems in joints, oral, and skin discussed above, a wide range of other applications were found in ocular system, hair, medical instruments, animal tribology, and biolubricant. The surface gel layer was found to be fundamental in reducing shear stress and damage to corneal epithelial cells [762]. A novel friction measurement system was developed to simulate the loading and motion conditions in a contact lens [763]. A simple cornea model was developed for the tribological assessment of different eye drops [764]. The resistance of hair to mechanical wear was correlated with hair mechanical properties and the effects of cosmetic treatments were evaluated [765]. An in vivo methodology was developed to investigate the tactile friction and sensory properties between human skin and hair, and effects of hair care products/shampoos were evaluated [766]. A wide range of tribological problems were examined, including in instruments [767], urinary catheter [768], rectal sexually transmitted infections [769], pleural mesothelial cells [770], gastroscope device-esophagus interface [771], cochlear [772], etc. The effective tribological mechanisms of animals and consequent bionics designs were investigated. A protein-based lubricating substance was found in reducing friction and wear in the femoro-tibial joint of an insect [773]. The normal and tangential peeling behaviour of gecko spatulae was investigated with a coupled adhesion-friction model [774]. Bionic cutting tool, wheel surface, and the tool surface of grinding were designed on the head microstructure of dung beetles [775], the frictional characteristics of ostrich planta [776], and bovine molar [777], respectively. The development of bio-lubricants such as based on vegetable oil were reviewed in Refs. [778, 779].

Many new potential biomaterials were developed. The tribological performances were considered in conjunction with biological effects such as anti-inflammation [780] and anti-microbial [781]. Various coatings were developed to improve the tribological properties [782]. The tribological characterization of 3D printed biomaterials was particularly focused [783].

## 5 High temperature tribology

### 5.1 Introduction of high temperature tribology

The authors in their previous review [1] had highlighted the importance, complexities, and rapidly growing research interest in high temperature tribology. A survey of research publications during 2020–2021 shows the increasing importance and continuing interest in high temperature tribology research. The published work in this field pertains to diverse tribological applications. However, the research efforts appear to devote greater efforts on controlling friction and wear at elevated temperatures. Therefore, this review accordingly covers the following aspects pertaining to high temperature tribology: (1) High temperature tribology in different applications; (2) control of friction and wear at elevated temperatures.

### 5.2 High temperature tribology in different applications

High temperature tribological studies reported during 2020–2021 span diverse applications. Some of these salient studies are reviewed below.

#### 5.2.1 *Manufacturing processes*

(1) Machining

Vereschaka et al. [784] investigated the tribological properties properties of a PVD deposited Cr, Mo–(Cr, Mo)N–(Cr, Mo, Al)N multilayer composite coating on carbide (WC+15% TiC+6% Co) inserts substrates against AISI321 steel at temperatures ranging from 500 to 1,000 °C in order to understand its cutting performance. These test temperatures are representative of those in the cutting zone of the tool in machining operation. The results showed that the adhesive component of friction ($f_{adh}$) increased almost linearly when the temperature increased from 500 to 700 °C. The increase of $f_{adh}$ slows down considerably at 800 °C. With further increase in temperature from 800 to 1,000 °C, $f_{adh}$ starts decreasing. The analysis of the wear pattern on the coating from cutting revealed a diffusion layer with the thickness of 20–40 nm formed due to diffusion of Fe and other steel components into the coating, as well as the diffusion of Mo from the coating into the steel being machined in combination with oxidation. During cutting with the coated tool, the thickness of the diffusion layer was 10–15 times lower compared to that in the uncoated tool.

Cui et al. [785] studied the tribological behavior of three different tool materials (high-speed steel, $Si_3N_4$, and SiC) against mild steel (MS) and high-chromium stainless steel using a ball-on-disc tribometer at 900 °C. This study showed that the two ceramic tool materials provide superior wear resistance compared to high speed steel (HSS) against mild steel counterface material. The HSS tool specimen suffered severe abrasive wear and damaged the glaze layer formed on the mild steel surface. In addition, HSS encountered sticking problem during rubbing against stainless steel. The use of ceramic tool materials promoted the formation of a thick (> 3 μm) tribo-oxide layer on stainless steel surfaces and reduced wear and transfer to the counterfce materials. ST surfaces, thereby reducing the wear of ST and its transfer to the counterface. However, the two ceramic tool materials against stainless steel suffered pull out wear due to their low toughness.

García-Martínez et al. [786] studied the tribological interaction of titanium aluminide Ti48Al2Cr2Nb against four carbide cutting tools, three with coatings (PVD TiAlN, CVD TiCN+$Al_2O_3$+TiN, PVD TiAlN+$AlCr_2O_3$) and one without coating for turning operations in a pin-on-disk test configuration over a wide range of temperatures from cryogenic temperature of –50 °C to high temperature of 600 °C with a view to select the most efficient cutting tool. The studies at subzero temperature were intended to explore the effectiveness of cryogenic lubrication using gaseous nitrogen. The measured friction coefficients (0.47 and 0.63) were much higher than that for conventional titanium alloys. The lowest values of friction coefficients were seen at 600 °C and it is mainly due to the formation of $TiO_2$ on the worn surface whereas the increase in friction at 300 °C was due to the presence of $Al_2O_3$ in the disk surface. In terms of wear performance, the coated inserts with PVD TiAlN and CVD TiCrN+$Al_2O_3$+TiN coatings, respectively, show poor performance during sliding against Ti48Al2Cr2Nb, mainly due to the high chemical affinity between the coatings and the aluminide. The uncoated tool performed better than the PVD TiAlN coated and CVD TiCN+$Al_2O_3$+TiN coated inserts. Moreover, it performed better than all other tools for temperatures up to 300 °C, but it was severely worn at 600 °C. The PVD TiAlN+$AlCr_2O_3$ coated tool showed superior performance at high temperatures in comparison to the other tools. The observed wear mechanisms up to 300 °C were ploughing and peeling-off but these change to adhesion and plastic deformation at 600 °C. At cryogenic temperature of –50 °C with gaseous nitrogen, the specific wear rates of Ti48Al2Cr2Nb alloy decreased due to the increased hardness.

Considering the critical aspect of transfer of material between workpiece and tool in machining operations is a critical aspect, Moghaddam et al. [787] investigated the effect of temperature on friction and wear of TiAlN PVD coated WC/Co sliding against stainless steel and carbide-free bainitic steel with special emphasis on coating degradation mechanisms and formation of tribolayers at different temperatures. During sliding between 316L stainless steel against TiAlN PVD coating, at a temperature of 800 °C, formation of a thin protective tribofilm consisting of aluminium oxide and oxidised transferred material could be effective in reducing wear of TiAlN coating. The generated tribofilm governs the tribological behaviour during the wear process. Removal of the tribofilm through abrasion or adhesion results in wear and reduction in thickness of the TiAlN coating at elevated temperatures as illustrated in Fig. 42.

(2) Hot forming

The problem of galling which essentially is a form of severe adhesive wear and material transfer is seen not only in machining of metals but it is also an acute problem in most metal forming processes. Dohda et al. [788] reviewed the actual galling conditions proposed by many researchers in order to better understand galling phenomena in metal forming. Further, the authors summarised the approaches for alleviating galling occurrence and methods for predicting galling as well as the studies to detect galling in metal forming processes.

The problem of material transfer and adhesion is particularly critical in forming Al alloys and Al coated steel. In this context, Cui et al. [789] conducted a fundamental study in order to explore the role of argon and air on the evolution of contact area (junction growth) in AA5083 alloy samples by *in-situ* observations through the contacting quartz glass counterface using an optical microscope incorporated in a high temperature pin-on-disc tribometer at 573 and 673 K, respectively. In argon atmosphere, junction growth occurred continuously and increased approximately by an order of magnitude for every 50 °C increase in temperature. However, in air, the junction growth was interrupted due to oxidation, resulting in stick-and-slip behavior due to formation and reformation of oxide layers after reaching a

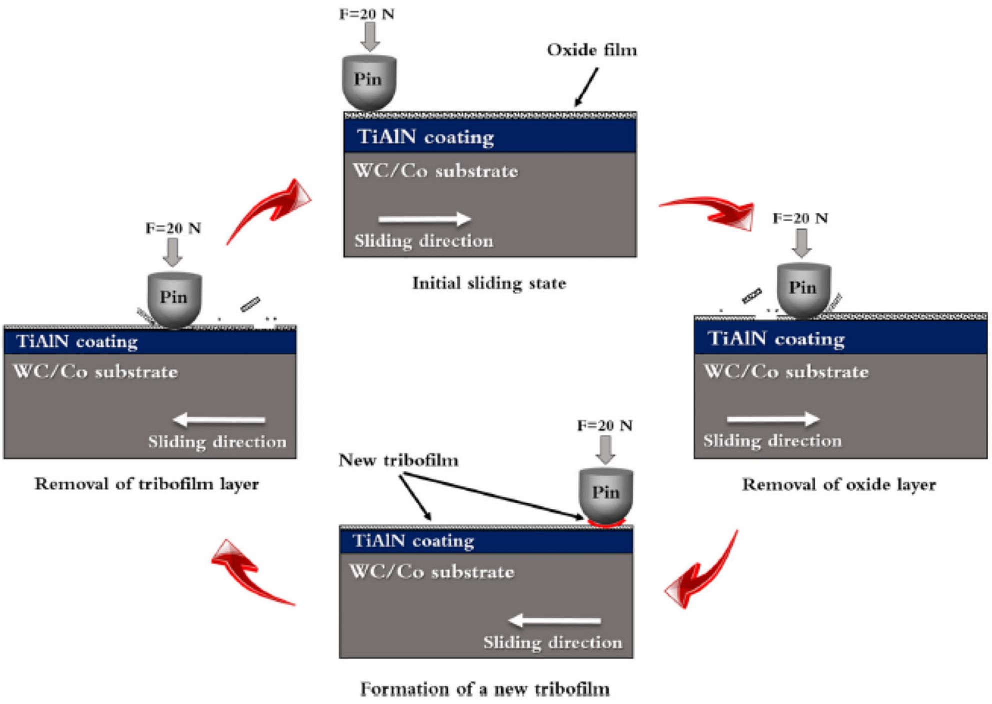

**Fig. 42** Schematic illustration of tribo-chemical wear mechanism of TiAlN coating tested against 316L stainless steel at 800 °C [787]. Reproduced with permission from Ref. [787], © Elsevier Ltd., 2021.

maximum tangential force. The tangential force at the onset of junction break decreased with increase in temperature. The authors concluded that the junction growth occurred because of thermally activated plastic deformation due to diffusion-controlled creep that required an activation energy similar to the self-diffusion of Al, and was accompanied by a grain boundary sliding.

Decrozant-Triquenaux et al. [790] studied the high temperature friction and wear behaviour of PVD coatings against aluminium under dry and lubricated reciprocating sliding conditions in a flat on flat contact configuration. In these studies, uncoated tool steel was used as reference and a warm forming polymer-based lubricant for lubrication during the tests. Friction was found to be particularly affected by the surface roughness of the PVD coatings and insignificant material transfer on the polished PVD coated surfaces occurred under lubricated conditions.

Decrozant-Triquenaux et al. [791] in another study investigated the effect of surface engineered hot work tool steel and lubrication on Al transfer during high temperature sliding against an Al alloy 6082 by using a hot strip drawing tribometer. The tool steel specimens were provided with two different PVD coatings ((PVD CrWN IonbondTM 35 and ta-C TetrabondTM Plus) and plasma nitriding. The lubricant used was a fully formulated potassium salt-based lubricant. The results showed that low friction (0.05) could be achieved when using properly selected PVD coated tool steel and a potassium salt-based lubricant. Low chemical affinity combined with optimized surface roughness to balance lubricant retention vs. mechanically initiated transfer is particularly important for high temperature sliding involving soft and reactive metals such as Al.

Pelcastre et al. [792] studied the tribological behaviour of Fe-based hardfacing materials deposited on a hot work tool steel tungsten inert gas (TIG) welding during sliding against Al–Si coated boron steel for press hardening in a flat on flat configuration at temperatures ranging from 600 to 750 °C. The results showed that the friction coefficient is not affected by the tool material, but its stability is affected by the temperature of the Al–Si coated steel. The wear of tool steels occurred as a result of abrasion due to the interaction with the hard Al–Si coating as well as progressive plastic deformation, grain refinement, and material flow.

Vikhareva et al. [793] investigated the high temperature tribological behaviour of additively manufactured maraging steel during sliding against Al–Si coated 22MnB5 boron steel. They found that the high temperature friction level and behaviour is similar for additively manufactured (AM) and conventionally produced tool steel whereas wear mechanisms involve formation of a material transfer layer with a layered structure resulting from intermixing between FeAlSi from the counter surface and the bulk tool steel.

Zhang et al. [794] carried out experimental investigations and FE simulations with a view to investigate the frictional behaviors and thermos-mechanical coupling characteristics of steel 22MnB5 against H13 steel at 800 °C in the context of hot forming. They observed that the coefficient of friction decreased and stabilized at higher load whereas the worn surface of the boron steel underwent large plastic deformation, resulting in scratches and furrows due to compression and shear by of die asperities. At high temperature and load, the plastically deformed surface layer of the boron steel adhered to the die surface leading to its peeling and delamination.

(3) Glass molding

The understanding of the tribological phenomena in the context of precision glass molding where optical components are formed in a single step process from heated glass by using molds typically made from tungsten carbide (WC) is of great importance since these affect the functional performance and lifetime of the molds. Different approaches such as surface coating or optimization of process parameters are used to control friction and wear. The understanding of the specifc tribological system comprising of the pressing tool and the heated glass in a highly rubbery state near the transition temperature is of particular interest. Chizhik et al. [795] developed a novel high-temperature tribometer to study the tribological properties of cemented WC/glass contacts at temperatures up to 700 °C using a pin-on-disk configuration. Two glass disk specimens were used in these studies; sodalime glass B270 as reference sample and acrylic glass PMMA as complementary sample. The results showed the efficacy of the newly developed tribometer for characterizing the friction of WC–glass interface near the glass transition. The coefficient of friction in case of soda lime glass B270 remained almost constant at temperatures well below $T_g$ (533 °C) and then it started increasing as the test temperature approached $T_g$. This increase has been attributed to diferent regimes of surface degradation including fracturing as well as plastic and viscoelastic deformation, as shown in Fig. 43. A similar behavior could be seen in case of complementary PMMA sample.

Currently, attempts are being made to reduce the mold-glass surface sticking and oxidation of the mold in order to ensure the desired surface quality. With this aim in mind, Li et al. [796] synthesized an amorphous carbon coating on the WC–8Co substrates by magnetron sputtering and conducted compression test by using a precision glass molding machine for evolution of friction under realistic glass molding conditions. The results showed that the friction of the coating/glass interface was highly dependent on the molding temperature, higher the molding temperature the smaller was the friction. They reported a coefficient of friction of about 0.3 at 680 °C. They also observed that the shear stress and friction between the coating and glass affected the service life of the mold. At the lower temperatures, the relatively higher shear stress resulted in wear and degradation of the coating. At the higher temperatures, the degradation of the coating was caused by oxidation and internal stresses.

(4) Hot dip galvanizing

Continuous hot dip galvanizing process is used for improving corrosion resistance of steel parts for wide ranging applications such as communication towers, ocean engineering, highway construction, and various other fields. However, damage caused by corrosion and erosive wear of equipment due to immersion in zinc solution for long periods is a major problem associated with hot dip galvanizing production. In this context, Zheng et al. [797] investigated the corrosion and erosive wear behavior of a directionally solidified Fe–B alloy in presence of high temperature zinc solution. In the liquid zinc erosive-wear system, the hard and corrosion resistant phase, $Fe_2B$, protects the ferrite matrix and the $\alpha$-Fe matrix supports the $Fe_2B$ in the Fe–B alloy with a "$Fe_2B\perp$interface" structure. It was observed that during the erosion-wear process, the ferrite matrix is preferentially eroded by the zinc liquid, and the $Fe_2B$ with different orientations protruding from the alloy surface resisted the wear damage.

### 5.2.2 *Nuclear power generation*

Nuclear energy is carbon-free, which makes it valuable for environmental protection. Coupled with the

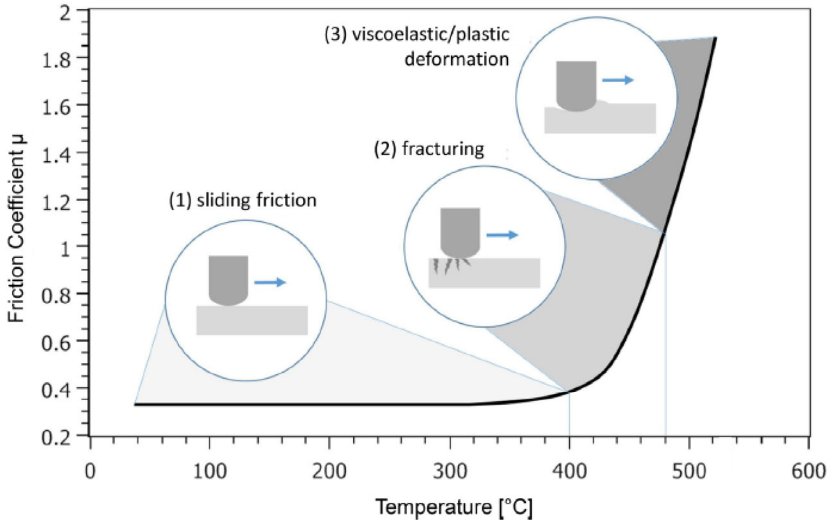

**Fig. 43** A representative friction coefficient vs. temperature curve for B270 glass obtained during heating indicating three different regimes of pin-glass interaction [795]. Reproduced with permission from Ref. [795], © The author(s), 2020.

depletion of fossil fuels, there is growing emphasis on nuclear power generation, especially in the developing countries. However, there are many high temperature tribological challenges pertaining to reactor components to be overcome in order to ensure safe and reliable operation of nuclear power generation.

Pauly et al. [798] investigated the high temperature tribological performance of Incoloy 88HT and Inconel 617 alloys used as structural materials in high temperature gas cooled reactors. The tribological performance of these alloys becomes particularly important since impurities such as $H_2O$ and $CH_4$ in the coolant lead to corrosion reactions that will affect tribological performance of the components. In this study, the alloys with different surface conditioning (high temperature $H_2O$ containing helium conditioned, carbon diffusion treated and conditioned after hardening) were evaluated for their tribological performance by using a pin-on-disk test configuration at high temperatures in ambient air, either 650 or 750 °C for alloy 800HT, and 850 or 900 °C for alloy 617. The wear resistance of alloy 617 in as-received condition was found to be superior to that of alloy 800HT overall due to its relatively higher hardness at the temperatures at which studies were conducted. Scuffing, glaze-oxide formation, and oxide-layer wear and break-through were identified as the wear mechanisms and these were influenced by the hardness, chemistry, and applied load.

Sodium cooled fast reactor utilizes molten sodium metal as the coolant. Its heat exchanger tubes are prone to impact wear damage due to flow-induced vibrations. The occurrence of impact wear can even lead to breakage of the tubes and lead to leakage that can lead to an aggressive sodium–water reaction with catastrophic consequences. Keeping this in mind, Chen et al. [799] investigated the impact wear behavior of 2.25Cr–1Mo steel against Gr5C12 alloy by using a controlled kinetic energy impact wear rig at temperature of 25, 225, and 450 °C. Highest impact wear occurred at 225 °C and lowest at 25 °C. The mechanism of impact wear reported were predominantly delamination at 225 °C, plastic deformation at 450 °C, and oxidative wear at and above 225 °C. A schematic of mechanisms of impact wear in the air is presented in Fig. 44.

#### 5.2.3 *Aero-engines*

Fretting is an important damage mode in case of

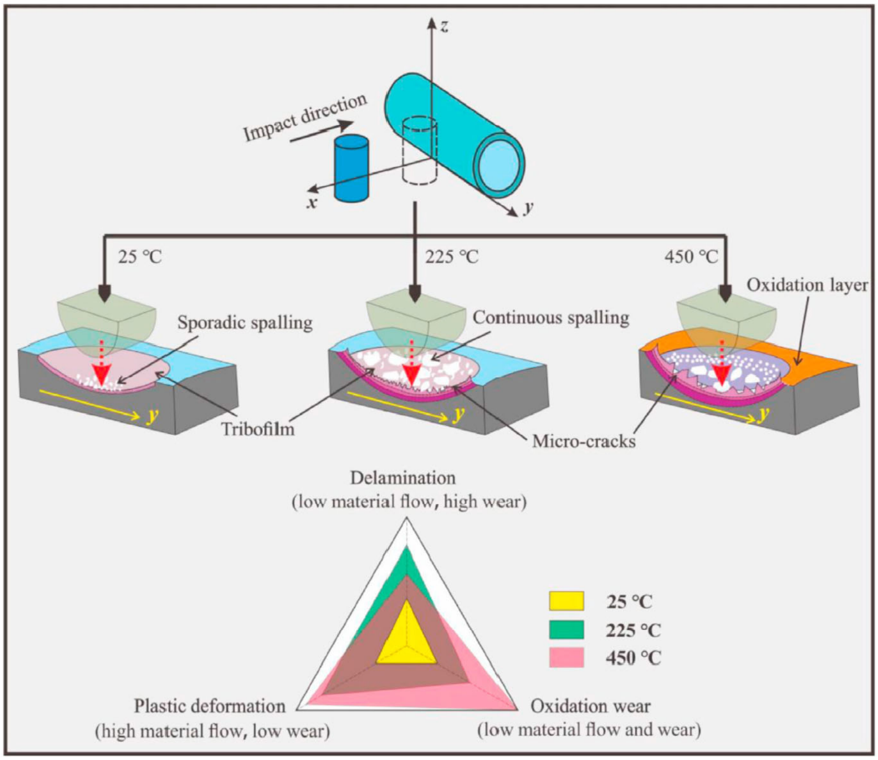

**Fig. 44** Schematic of mechanisms of impact wear [799]. Reproduced with permission from Ref. [799], © Elsevier B.V., 2021.

aero-engine turbines. In this context, Lavella and Botto [800] investigated the fretting behaviour of CoMoCrSi superalloy (Tribaloy®T800) coatings (deposited via plasma spray and TIG welding) under conditions representative of the dovetail or fir-tree type joints of turbine blade attachment to the rotor and the contact between adjacent shrouds placed on blades tips (also known as *z*-notch due to the shape of the contact between shrouds). The fretting studies were conducted by using point contact and flat-on-flat configurations at room temperature, 600, and 800 °C, respectively. The results from point contact configuration tests at room temperature showed stable contact properties independent of fretting cycles and the friction coefficient was independent of the normal load. On the other hand, nonlinearity was observed in wear volume at low and high dissipated energies but a linear volumes-energies trend, substantially independent of normal load for intermediate dissipated energy could be seen. More rapid wear of T800 occurred at room temperature compared to that at high temperature. At room temperature in point contact configuration, the fretted (worn) surface was fully covered by brittle cracks and the surface damage occurred due to abrasive ploughing action. In flat on flat configuration, the dual layer deposition in case of TIG welded T800 resulted in lower wear than that with than single layer at room temperature and 800 °C However, at 400 °C, the wear was slightly higher in dual layer deposition than that of single layer at 400 °C. Oxidation process at 800 °C (negative wear volumes) was more pronounced in single layer welding than in dual layer.

Keeping in view the potential of electron beam powder bed fusion (EBPBF) manufacturing process for manufacturing complex shaped parts, including those for aerospace applications, Alvi et al. [801] investigated the dry sliding wear behaviour of EBPBF produced Ti6Al4V alloy against steel and alumina counterpart balls in a ball on disc configuration from room temperature (RT) to 500 °C. The EBPBF produced Ti6Al4V alloy showed the lowest wear rate in tests against both the steel and alumina counterparts at 200 °C. In case of tests against steel, the wear mechanism changed from abrasive wear at room temperature to adhesive wear at 400 °C. At 500 °C, material transfer from steel ball to Ti6Al4V alloy and oxidative as well as mild abrasive wear mechanisms were seen. In the case of tests against alumina ball, oxide glaze layers were formed at all temperatures and the glaze layer thickness increased with increasing temperature.

### 5.3 Control of friction and wear at elevated temperatures

Broadly the three main approaches have been used in controlling friction and wear at elevated temperatures: (1) Use of lubricants; (2) choice of materials; and (3) use of surface modification technologies.

#### *5.3.1 High temperature lubricants*

Bi et al. [802] used two gas-phase monomers phthalic anhydride (PA) and p-phenylenediamine (PPD) through their introduction into the contact region for synthesis of solid lubricant film and its replenishment during on friction couples for effective lubrication at high temperature of 500 °C. The authors illustrated the effectiveness of generating solid lubricating film by polymerization of PA and PPD in a 38-min medium-term test using a two E52100 steel balls on W6Mo5Cr4V2 high speed steel disc test configuration as can be seen from Fig. 45.

Decrozant-Triquenaux et al. [803] investigated the behaviour of three different tool steels with different surface topographies during sliding against aluminium under dry and lubricated conditions. The tests were conducted by using tool steel a pin on Al disc contact configuration under reciprocating conditions. The lubricants used were a hexagonal boron nitride based lubricant (hBN) and a polymer-based aqueous emulsion (Pol.). The Al disc specimens for tests were used in as received (AR) condition as well as in mirror polished (MP) condition. The test temperature of 300 °C was used for tribological studies. The hBN lubricant was found to be ineffective as a result of poor adhesion to the interacting surfaces and easy removal from the sliding interface whereas the polymer-based aqueous emulsion could reduce both the friction as well as material transfer due to the formation of a carbon-rich tribolayer on the tool steel surface, as shown in Fig. 46. The tool steel surface roughness had also a significant impact on the

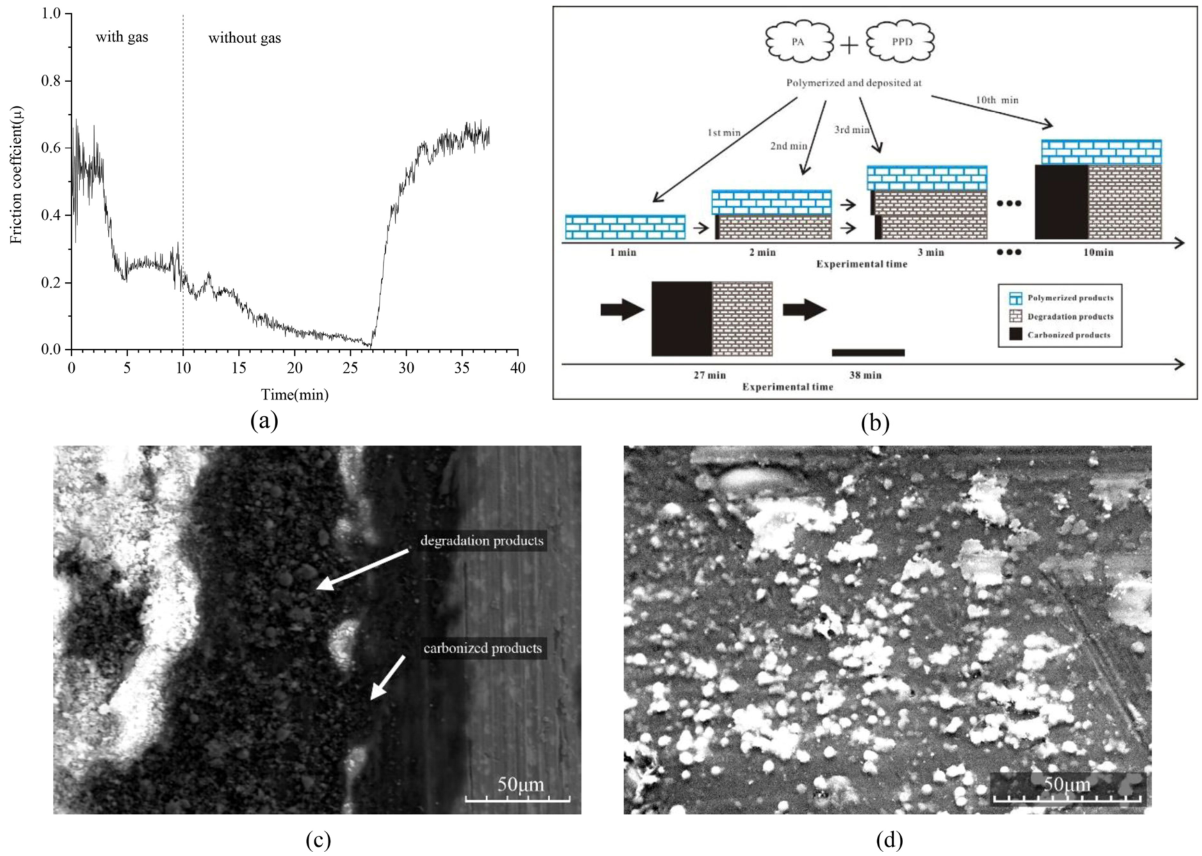

**Fig. 45** (a) Coefficient of friction curve for test PA+PPD-38; (b) change in composition of the solid lubricant film during the test; (c) SEM of the steel disc at 27 min; and (d) SEM of the steel ball at 27 min [802]. Reproduced with permission from Ref. [802], © Society of Tribologists and Lubrication Engineers, 2021.

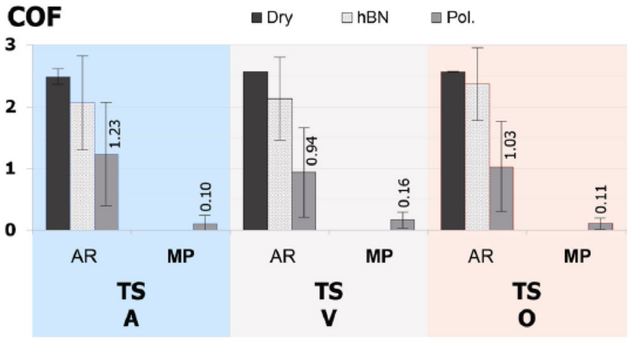

**Fig. 46** Average friction levels over the last 25 s of tribotests for all test configurations (load: 10 N, stroke length: 4 mm, frequency 12.5 Hz, temperature: 300 °C) [803]. Reproduced with permission from Ref. [803], © The author(s), 2021.

frictional behaviour and the severity of material transfer.

Torres et al. [804] explored the potential of graphene as a high temperature (HT) solid lubricant under conditions representative of Al forming and compared it with that of other commonly used solid lubricants such as graphite and hBN. The authors also investigated the compatibility of the solid lubricating materials with a novel self-lubricating wear resistant cladding. Tribological studies on tool steel and Ni-based self-lubricating cladding (NiCrSiB+Ag+$MoS_2$) against AA2014 aluminium were conducted by using a load scanner tribometer (single pass, load increases from 50 to 400 N) at 400 °C. Graphene (5 wt%) performed extremely well as a HT grease additive at 300 °C, with significantly lower friction than graphite or hBN. Also negligible material transfer could be observed on the tool steel samples.

### 5.3.2 *Materials*

While considering materials in the context of high temperature tribology, studies have been performed on diverse range of materials such as metallic materials, high entropy alloys, ceramics, polymers, and their composites. Salient findings of some of these studies pertaining to various materials are discussed below.

(1) Metallic materials

Bai et al. [805] studied the tribological characteristics of 40CrNiMoA steel and Inconel 718 alloy during sliding against $Si_3N_4$ counterparts in a ball on disc test configuration from room temperature to 800 °C. They reported that the friction coefficients and wear rates of both the alloys decreased at higher temperatures mainly due to the formation of tribo-oxide film. The authors hypothesized that at higher temperatures both the formation of surface oxides as well as thermal softening of the materials mainly determine the tribological performance and different materials show different tribological responses.

Philip et al. [806] studied the effects of electrical discharge machining assisted alloying (EDA) and electrical discharge machining assisted alloying (WEDA) on Ti6Al4V ( termed as Ti64) resulting surfaces and their friction and wear behaviour at temperatures of 200, 400, and 600 °C, respectively. The authors noted that the formation of recast layers (RL) during EDA and WEDA of Ti64 specimens is different considerably in terms of thickness and carbon content. The recast layers were also reported to significantly affect their wear at elevated temperatures. The EDATi64 specimens showed consistent wear at all test temperatures behaviour. The formation of oxides ($TiO_2$, $Ti_8O_{15}$, and $Fe_2O_3$) and carbides (TiC and $Ti_{24}C_{15}$) in the recast layer has been attributed to the superior tribological performance of EDA Ti64 specimens. The WEDATi64 specimens has excellent wear resistance at 200 and 400 °C but its wear resistance at 600 °C was similar to that of bare Ti64 specimen.

(2) Metal matrix composites (MMCs)

As regards MMCs, high temperature, tribological studies have been mainly conducted on Al and Mg alloy matrix composite materials [807, 808], mainly keeping in view the need for light weight construction in the context of aerospace and automobile applications.

Tan et al. [807] developed Al–Si alloy containing 30 wt% Sialon composite material by using spark plasma sintering (SPS) process and characterised its microstructure, mechanical properties, and tribological behavior from room temperature to 600 °C. The developed Al–Si/Sialon composite showed comparatively low and stable friction as well as low wear rate in the temperature range RT to 500 °C vis a vis that of Al–Si alloy. However, both the friction as well as wear performance deteriorated at temperatures above 500 °C is due to occurrence of severe adhesive wear.

In another study, Sharma et al. [808] fabricated Al alloy (LM30) matrix composites containing sillimanite mineral particles of different sizes and in different amounts. They conducted friction and wear tests of different composite material samples at temperatures ranging from 50 to 300 °C. The effects of different heat treatments (T4 and T6) of the Al alloy matrix composites on their friction and wear behaviour was also investigated. The developed composites with heat treatments showed improved friction and wear properties. The authors claimed that the Al alloy matrix composite containing 15 wt% of sillimanite mineral particles having fine to coarse particles ratio of 3:1 and subjected to T6 heat treatment could match the wear performance of commercial grey cast iron at operating temperature of 200 °C and contact pressure of 1 MPa.

Regarding Mg alloy matrix composites, Surendran and Gnanavelbabu [809] developed Mg alloy (AZ91D) based composites containing ultra high temperature ceramic particles of TiC, $TiB_2$, and TiN respectively by using stir-ultrasonic treatment-squeeze casting method and studied their tribological behaviour from room temperature to 200 °C against EN31 steel disc in a pin on disc configuration. All the composites showed superior wear and friction performance compared to unreinforced AZ91D Mg alloy at room and elevated temperatures.

(3) Ceramic materials

Contrary to expectation, the high temperature tribology research activities on ceramics have been limited. Keeping in view the harsh operating conditions in aerospace applications, ultra-high temperature ceramics (UHTCs) in view of their exceptional combination of properties such as high hardness, thermal resistance, and chemical stability have potential for high temperature tribological applications. With this as the key motivating factor, Lu et al. [810] developed polymer-derived $Ta_4HfC_5$ nanoceramics characterised their tribological behaviour by using a $Si_3N_4$ ball against nano ceramic disc rotational tribometer at room temperature, 300, 600, and 800 °C, respectively. The friction coefficients first increased from 0.3 at room temperature to about 0.7 at 300 °C, slightly decreased at 600 °C and then finally reaching the lowest value of 0.2 at 800 °C. The carbon phase has been reported to play an important role in the tribological behavior of polymer-derived ceramics and the low friction at room temperature has been attributed to the availability of free carbon. The lowest friction of friction of about 0.2 at 800 °C reported was mainly due to the formation of compacted oxidized layer through tribo-oxidation of $Ta_4HfC_5$ as well as the fine microstructure of the nanoceramic.

(4) Ceramic composites

Wang et al. [811] prepared *in situ* formed TiAl–$TiB_2$ composites containing containing 0, 10, 20, 30, and 40 vol% of $TiB_2$ and studied the friction and wear behaviour at RT, 200, 400, 600, and 800 °C temperatures during sliding against $Si_3N_4$ balls by using a ball-on-disc type vacuum friction tester at a low vacuum of 1 Pa. No clear trend in friction behaviour of different TiAl–$TiB_2$ composites could be seen when the temperature was increased from RT to 800 °C. The wear rates of all composites were low (about $10^{-5}$ $mm^3/(N\cdot m)$) with composite containing 20 vol% of $TiB_2$. Composites containing $TiB_2$ above 20% content resulted in higher wear owing to increased brittleness of the composites and consequent delamination in B-rich areas. The wear mechanisms of the composite containing 20% $TiB_2$ were found to be abrasive wear at low temperatures (RT–200 °C), slight abrasive wear and oxidation wear at moderate and high temperatures.

(5) Self-lubricating materials

Zhao et al. [812] investigated the tribological properties of non-impregnated and furan impregnated graphite as a function of temperature and load against tungsten carbide ball under reciprocating sliding conditions and elucidated the evolution of carbon based tribofilm. The results showed that impregnated graphite has considerably superior tribological performance compared to that of non-impregnated graphite. Both friction and wear decreased at temperature of 160 °C compared to those at 20 °C. The improvements of tribological properties was attributed to the formation of a uniform and stable friction induced carbon film that adsorbs at the rubbing interface at the higher temperature.

Wang et al. [813] developed $Ni_3Al$–Ag based self-lubricating alloys containing different amounts of Ag, Cr, Mo, Zr, and B through powder metallurgy route. The high temperature friction and wear behaviours of these different alloys were investigated by using a high temperature ball on disc test configuration from room temperature up to 900 °C. The counterface ball was made of silicon nitride whereas the disc specimens were made of different $Ni_3Al$ alloys. $Ni_3Al$ alloy containing 20% Ag was found to possess good self-lubricating performance in a wide temperature range. The authors proposed that at temperatures from RT to 400 °C, Ag provides effective lubrication whereas at high temperatures between 800 and 900 °C, the formation of a lubricious glaze film composed of $Ag_2MoO_4$ and NiO prevents direct metal to metal contact and enhances high temperature tribological performance as illustrated in Fig. 47.

In another study, Wang et al. [814] synthesized two-phase $Ni_3Al$–$Ni_3Nb$ alloys with relative weight ratios of 3:1 (designated as NLB60) and 1:3 (designated as NBL60) between $Ni_3Al$ and $Ni_3Nb$ and fixed amount (20%) of Ag through powder metallurgy process. The addition of Ag in these two alloys led to improvements in friction and wear performance with NLB60 showing superior performances compared to that of NBL60 at temperatures ranging from room

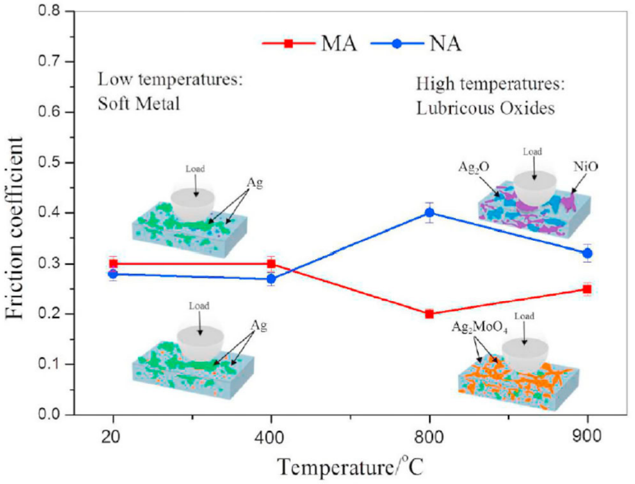

**Fig. 47** Illustration of the lubrication mechanism of $Ni_3Al$–Ag alloy (NA) and $Ni_3Al$–Ag alloy containing Cr, Mo, Zr, B (MA) [813]. Reproduced with permission from Ref. [813], © Elsevier B.V., 2021.

temperature to 900 °C. The improved tribological performance has been attributed to the synergetic effect of solid lubricantion by Ag at low-moderate temperatures and $AgNbO_3$ at high temperatures.

(6) High entropy alloys (HEA) and ceramics

Sadeghilaridjani et al. [815] investigated the deformation and wear behaviors of two refractory high-entropy alloys, HfNbTiZr and HfNbTaTiZr from room temperature to 573 K. HfNbTaTiZr HEA alloy showed higher hardness as well as strength and lower strain rate sensitivity compared to the HfNbTiZr HEA. Wear rate of both the alloys increased as the temperature was raised from room temperature to 423 K due to thermal softening but it decreased at 573 K due to formation of surface oxide layer. The wear mechanism was abrasive at room temperature, ploughing at intermediate temperature, and oxidative at high temperature.

Vo et al. [816] studied the effect of oxidation on the tribological properties of an as-cast AlFeCrCoNi2.1 eutectic high entropy alloy (EHEA) at temperatures ranging from 500–900 °C under reciprocating sliding conditions in a ($Si_3N_4$) ball on disc configuration. The EHEA comprising of five elements, plus oxygen and the dual FCC/$B_2$ lamellae structure constitutes a complex tribo-bed. The results showed that high temperature oxidation plays an important role in determining the tribological behaviour of the EHEA. The coefficients of friction varied around 0.45 at 500–600 °C and 800–900 °C. However, at 700 °C, the coefficient of reached the maximum value of about 0.7 with relatively long running-in duration. At temperatures above 700 °C, oxidative wear was main wear mechanism with small contributions from abrasive and adhesive wear mechanisms. On the other hand, abrasive wear was dominant below 700 °C.

Zeng et al. [817] applied an AlCoCrFeNiTi0.5 HEA coating on Ti–6Al–4V alloy by laser cladding and investigated its microstructure, micro-hardness, high-temperature wear, and oxidation resistance. This HEA coating was mainly composed of dendritic phase which was a FCC solid solution rich in Al–Co–Ni–Ti and interdendritic phases which were BCC solid solution, rich in Fe–Cr (IR1) and Fe–Cr–Ti–Co–Ni, respectively. The results revealed that the HEA coating has superior oxidation resistance than Ti–6Al–4V alloy. The hardness of the HEA coating was 706.3 HV which was about 2.32 times that of the substrate and its wear resistance was about 12 times that of the substrate. In terms of wear mechanisms at 600 °C, a combination of adhesive and oxidative wear mechanisms were identified in case of HEA coating whereas abrasive and oxidative wear mechanisms could be seen in case of Ti–6Al–4V alloy substrate.

Geng et al. [818] presented a strategy for designing (NiAl)$x$(FeCr)$y$Co($x$+$y$)/2 HEAs system with nano-coupled heterostructure based upon controlling the (Ni, Al)/(Fe, Cr) ratio for achieving good combination of mechanical and high temperature tribological properties. As the authors have mentioned, the AlCoCrFeNi HEAs are composed of FCC phase (Fe, Co-rich) at the grain boundaries and a BCC phase thus forming FCC/BCC heterogeneous structure. The BCC phase in the HEA is nano-coupled hetero-structure comprising of disordered A2 (Fe, Cr-rich) phase and ordered B2 (Ni, Al-rich) phase. For their investigations, the authors prepared three HEAs by varying the $x$:$y$ molar ratio value of 1:3, 1:1, and 1.3:1. Their findings showed that the mechanical properties of the (NiAl)$x$(FeCr)$y$Co($x$+$y$)/2 HEAs are determined by the evolution of the microstructure. The friction coefficients of the HEAs were about 0.39–0.80. These decreased from room temperature to 400 °C, increased slightly at 600 °C, and then decreased significantly at 800 °C. The wear rates of the HEAs were about (1.0–3.8)×$10^{-4}$ $mm^3$ from RT to 400 °C and thereafter decreased above 600 °C by an order of magnitude. Both the friction reduction as well as wear resistance of (NiAl)1.3(FeCr)1Co1.15 are superior to the other two HEAs.

Xin et al. [819] characterised the micro-hardness and tribological properties of the Al0.2Co1.5CrFeNi1.5Ti0.5+ $x$C$x$ ($x$ = 0, 1.0, and 2.0) HEAs from 25 to 800 °C. The tribological tests were conducted in $Si_3N_4$ a ball on HEA disc test configuration. There was significant increase in micro-hardness (from 295 to 525 HV) of HEAs at 800 °C when the molar content increased from 0 to 2.0. At temperatures below 600 °C, the wear resistance of the HEAs increased with increasing carbon content, but it was inversely proportional to carbon content above 600 °C. The higher carbon content in HEA led to severe oxidative wear and poor wear resistance of HEA at 800 °C.

Miao et al. [820] prepared a eutectic high entropy alloy (EHEA) AlCoCrFeNi2.1 by vacuum arc melting and studied its tribological behaviour against four difference counterface materials, namely $Al_2O_3$, $Si_3N_4$, SiC, and GCr15 steel respectively in ball against EHEA disc test configuration from RT to 900 °C under unidirectional sliding conditions. The EHEA showed excellent softening resistance and could retain high hardness over 197 HV up to a temperature of 900 °C which is superior to that of Inconel 718 super alloy. Of all the different tribopairs, the SiC–EHEA tribopair resulted in minimum friction coefficients and wear when sliding against SiC at room temperature. However, for friction coefficient gradually increased to over 0.7 in the temperature range 200–600 °C but then rapidly decreased above 600 °C due to the transition to an oxidative wear and formation of fine grained layer deformed layer below the worn surface. The wear rates of the EHEA as well as SIC ball however gradually increased when the temperature was increased from RT to 900 °C.

Sun et al. [821] developed a single phase (Hf0.2Mo0.2Nb0.2Ta0.2Ti0.2)C high entropy ceramics (HEC) by spark plasma sintering (SPS) process at different sintering temperatures (1,900, 1,950, 2,000, and 2,050 °C, respectively) and analysed as to how their microstructure and temperature affect its mechanical and tribological properties. Tribological tests were conducted using an $Al_2O_3$ ball on HEC disc configuration in the temperature range 25–900 °C under unidirectional sliding condition. All HEC ceramics showed excellent wear performance at high temperatures, especially, in the temperature range 25–300 °C where the lowest wear rate was $10^{-7}$ $mm^3/(N{\cdot}m)$ and it has been ascribed to the good mechanical properties of the HEC and to the formation of interfacial tribofilm.

### 5.4 Surface modification technologies

Huai et al. [822] prepared a composite bonded coating having thickness of about 80–120 μm and composed of a suitable mixture of amorphous $SiO_2$, solid lubricant graphite, and $Al(H_2PO_4)_3$ binder on titanium (TC4) discs and studied its tribological performance using a ($Si_3N_4$) ball on coated TC4 disc configuration at temperatures of 700, 800, and 900 °C, respectively. This coating provided effective lubrication at high temperatures, with very low friction coefficients of about 0.05 and negligible wear in air. The improved performance of graphite was attributed to the protection provided by $SiO_2$ to graphite against its oxidation and preventing its loss as $CO_2$ gas from the coating.

Torres et al. [823] explored the potential of graphene as a high temperature (HT) solid lubricant under conditions representative of Al forming and compared it with that of other commonly used solid lubricants such as graphite and hBN. The authors also investigated the compatibility of the solid lubricating materials with a novel self-lubricating wear resistant cladding. Tribological studies on tool steel and Ni-based self-lubricating cladding (NiCrSiB+Ag+$MoS_2$) against AA2014 aluminium were conducted by using a load scanner tribometer (single pass, load increases from 50 to 400 N) at 400 °C. Graphene (5 wt%) performed extremely well as a HT grease additive at 300 °C, with significantly lower friction than graphite or hBN. Also negligible material transfer could be observed on the tool steel samples.

Zhu et al. [824] prepared WSN coating by doping $WS_2$ with N using magnetron sputtering process and investigated their tribological performance at elevated temperatures of 200 to 500 °C against a $Si_3N_4$ ball counterpart. This WSN coating comprising of amorphous phases of $WS_2$, $WN_x$, and $WO_3$ as well as nano-crystalline phase of $WS_2$ showed extremely low friction coefficients: 0.008 at 200 °C, 0.009 at 400 °C, and 0.006 at 500 °C. The authors proposed a lubrication mechanism as illustrated in Fig. 48 and attributed the formation of a $WO_3$ transfer film with extremely low shear strength during sliding for low friction and wear at 500 °C.

In an attempt to expand the potential of MXenes in high temperature tribology field, Xue et al. [825] investigated the role of texturing coupled with the use of solid lubricants Sn–Ag–Cu incorporating MXene–$Ti_3C_2$ in improving the high temperature tribological performance of CSS-42L heat resistant bearing steel. The authors compared the tribological performance of CSS-42L bearing steel having smooth surface, textured surface, textured surface containing Sn–Ag–Cu solid lubricant and textured surface

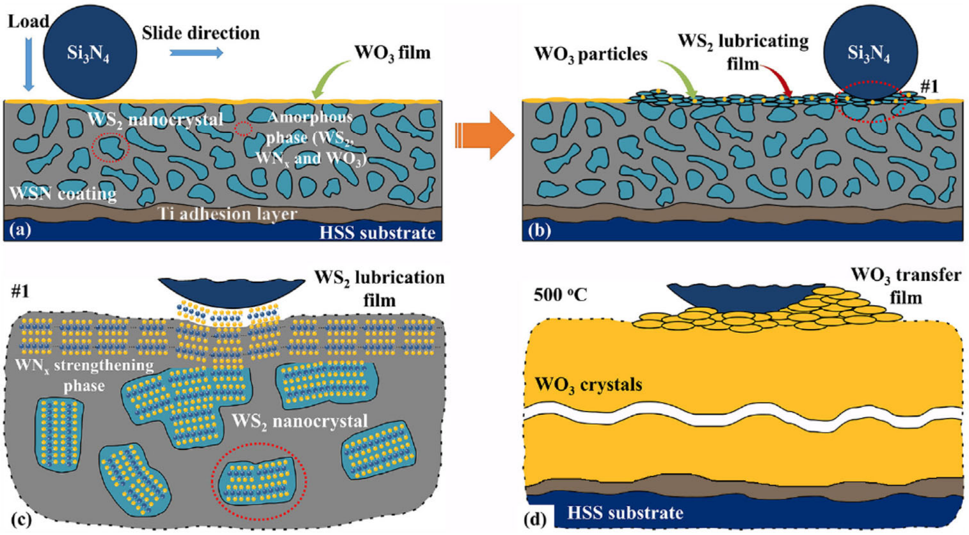

**Fig. 48** Schematic of lubrication mechanism. (a, b) Lubrication system; (c) partial enlarged view of friction pair contact area #1; (d) formation for $WO_3$ transfer film at 500 °C [824]. Reproduced with permission from Ref. [824], © Elsevier B.V., 2021.

containing Sn–Ag–Cu solid lubricant as well as MXene–$Ti_3C_2$ respectively from room temperature to 450 °C in a $Si_3N_4$ ball and disc configuration under unidirectional sliding conditions. The disc specimens were made of heat resistant bearing steel with different surface states such as polishing, texturing, and texturing filled with solid lubricants. The authors concluded that the CSS-42L bearing steel with surface texture grooves filled with Sn–Ag–Cu–$Ti_3C_2$ provided the best tribological performance compared to other surfaces in the entire test temperature range of 25 to 450 °C. This enhancement in tribological performance by Sn–Ag–Cu–$Ti_3C_2$ filled texture grooves at high temperatures has been attributed to the occurrence of tribochemical reactions resulting in formation of variety of intermetallic compounds and oxides.

Shi et al. [826] deposited Ni-based composite coatings incorporating $TiO_2$–ZnO–$MoO_3$ on GH4169 superalloy disc substrates and investigated their tribological performance in $Al_2O_3$ ball on disc contact configuration under unidirectional sliding conditions at temperatures of 25, 400, and 800 °C. These coatings were characterised by lamellar structure and the higher mass ratio of $MoO_3$ in the matrix led to increased porosity. The composite coatings containing multiple oxides displayed superior wear behaviour at 800 °C and this has been attributed to the formaion of a lubricious tribolayer composed of binary oxides (A–$TiO_2$, R–$TiO_2$, ZnO, and NiO) and ternary oxides ($ZnMoO_4$, $Zn_2TiO_4$, and $NiMoO_4$). The reduction of friction due to the addition of $MoO_3$ at 800 °C is due to the high ionic potentials of $MoO_3$ and large differences in the ionic potential of $ZnMoO_4$ and $NiMoO_4$.

Hao et al. [827] prepared HVOF NicrAlYTa/10Ag/5Mo coatings on Inconel 718 substrate and investigated its hot corrosion behaviour in molten salt with or without $V_2O_5$ as well its tribological performance against $Al_2O_3$ ball counterpart at 800 °C. The composite coating suffered more severe corrosion when exposed to $Na_2SO_4$–NaCl–$V_2O_5$ molten salt than that when exposed to $Na_2SO_4$–NaCl. The composite coating also showed superior wear resistance after hot corrosion in molten salt $Na_2SO_4$–NaCl–$V_2O_5$ than that in $Na_2SO_4$–NaCl. This has been attributed to the formation of the thicker oxidative scale and the better mechanical properties due to hot corrosion in molten salt $Na_2SO_4$–NaCl–$V_2O_5$.

Tian et al. [828] deposited graphite modified WC–12Co coatings on 304 stainless steel substrates by using the detonation gun spraying system and studied its friction and wear characteristics at temperatures ranging from room temperature to 400 °C. Friction and wear tests were conducted by using a (WC–steel alloy) ball on coated disc test configuration under reciprocating conditions. The modification of WC–12Co coating with the incorporation of graphene resulted in significant reduction in friction

(up to about 50%) at all temperatures compared to that of unmodified WC–12Co coating. The graphene modified coating also provided superior wear performance, specifically at temperatures of 200 and 400 °C respectively. The improvement in friction and wear of the graphene modified coating has been attributed to the presence of graphene film (of the order of nanometers) covering the wear tracks of coated surfaces that enables effective lubrication.

Plasma sprayed yttria-stabilized zirconia (YSZ) ceramic coatings in view of their several favourable properties have potential for high temperature tribological applications. However, they suffer from brittleness and are prone to internal cracks. Yang et al. [829] chemically modified the YSZ coatings by incorporating Ag and $Ag_2MoO_4$ nano particles into the pores (defect sites) by using vacuum impregnation and hydrothermal reaction. Prior to deposition of the YSZ coating, a NiCrAlY bond coating was applied on to the Inconel 718 alloy substrate in order to reduce the residual stress between the substrate and the ceramic coating. The authors demonstrated that the incorporation of Ag and $Ag_2MoO_4$ additives in the YSZ plasma sprayed coating remarkably reduced friction coefficients and wear rates in an $Al_2O_3$ ball on coated disc test configuration under reciprocating sliding at 800 °C. The authors attributed the improved friction and wear performance at 800 °C to the synergistic lubrication, i.e., liquid lubrication by Ag, $MoO_3$, $Ag_2MoO_4$ coupled with the formation of transfer film on the $Al_2O_3$ ball counter surface.

Tillman et al. [830] deposited stoichiometric $MoS_2$ and a sub-stoichiometric $MoS_{1.6}$ films of about 5.3 thickness on 16MnCr5 steel by high power impulse magnetron sputtering system with a view to understand the role of the structures of films on their high temperature tribological properties. The tribological tests were conducted by using a ball on disc tribometer test configuration against 100Cr6 steel counterpart at temperatures ranging from RT to 400 °C. Their results showed that the friction behaviour is strongly dependent on the temperature and a transition to low friction occurred at a temperature of 200 °C due to desorption of water molecules and an increasing intensity of the (002) basal plane. The sub-stoichiometric film showed superior tribological properties due to a passivation mechanism caused by the sulfur defect sites vis a vis the stoichiometric film. At temperatures above 200 °C, friction increased for both films and at 400 °C both films failed due to delamination during the tests. The authors concluded that the high temperature friction of molybdenum sulphide films is governed by complex interaction of crystallite re-orientation, desorption, and oxidation processes.

Tsigkis et al. [831] in an attempt to identify the most suitable mating (counterface) material for the latest version of plasma sprayed PS400 coating conducted tribological investigations at 25 and 500 °C by using a hemispherical ended pin against disc contact configuration in a high temperature tribometer. The titanium pin specimens were coated with plasma spray PS 400 coating and subsequently polished it, with the resultant coating thickness of about 200 μm. The counterface disc materials were 4130 steel in bulk form and DLC, poly-crystalline diamond (PCD) and molybdenum boride cobalt chrome (MoB/CoCr) coatings applied on to 4130 steel substrates respectively. The PS400 vs. DLC tribopair showed favourable tribological properties at 25 and 500 °C at low sliding speeds indicating its potential for space related applications such as bearing probes for venus missions.

Sun et al. [832] studied the effect of adding different amounts of $Bi_2O_3$ 20 to 50 wt% to Ni–5 wt% Al matrix composite coatings on their tribological performance in a broad range of temperature from room temperature to 800 °C. The coatings were deposited on S31008 high chromium–nickel austenitic stainless steel 06Cr25Ni20 substrates by an atmospheric plasma spraying system. The tribological tests were conducted by using a ball on disc contact configuration in which $Al_2O_3$ balls were used as counterparts against coated discs. The results showed that addition of $Bi_2O_3$ enhanced the lubricating effectiveness of the Ni–5 wt% Al matrix composite coatings that varied with operating temperature. Composite coatings containing 25 wt% and 33.3 wt% $Bi_2O_3$ showed significant reduction in friction and wear at 800 °C. This was attributed to the enhanced lubricating effectiveness due to the diffusion of $Bi_2O_3$ under the combined effects of shearing and temperature as well as simultaneous oxidation of Ni. The NiAl–25 wt% $Bi_2O_3$ composite coating especially showed superior friction reducing ability resulting in very low friction coefficient of 0.13 at 800 °C. On the other hand, the composite coatings containing 42.9 wt% and 50 wt% $Bi_2O_3$ showed higher friction coefficients at 800 °C owing to excessive amount of $Bi_2O_3$ solid lubricant and consequently higher plastic deformation.

Sun et al. [833] in another study applied NiAl–$25Bi_2O_3$–15Ag–$6Cr_2O_3$ composite coating on S31008 high chromium–nickel austenitic stainless steel 06Cr25Ni20 substrates by an atmospheric plasma spraying process. The tribological response of the composite coating was studied using a ball on disc tribometer by using an $Al_2O_3$ ball as counterpart under unidirectional sliding conditions. The experiments were conducted at a set of fixed temperatures, room temperature, 400 and 800 °C respectively as well as in thermal cycling tests in which temperature was varied in steps, room temperature, 400 °C, 800°C, 400°C and room temperature respectively. The results showed that the friction and wear behaviour differed significantly between these two types of tests. The composite coating showed superior friction and wear at 800 °C compared to that at ambient temperatures.

Wang et al. [834] deposited $TiB_2$–50Ni coatings on 304 stainless steel substrates by using 'shell and core' type $TiB_2$ powder coated with Ni in order to prevent oxidation of $TiB_2$ powder during HVOF spraying process. The tribological studies were conducted by using a ball on disc contact configuration by employing SiC balls as counterparts from room temperature up to 600 °C. The friction and wear results of the composite coatings were compared with that of the substrate 304 stainless steel without coating. The results showed that the HVOF sprayed $TiB_2$–50Ni composite coating provides much superior tribological performance compared to that of 304 stainless steel at high temperatures. As the test temperature increased from room temperature to 600 °C, the friction coefficients showed large fluctuations and wear rates increased rapidly in case of 304 stainless steel. On the other hand, the composite coating displayed relatively stable friction and significantly low wear rates ((about $2.73\times10^{-5}$ mm$^3$) compared to that of 304 stainless steel (about $11.07\times10^{-5}$ mm$^3$/(N·m)). The improved high temperature tribological performance of the composite coating was attributed to the formation of a continuous and stable oxide film.

Shi et al. [835] incorporated mixed $TiO_2$/ZnO powders during deposition of Ni- and NiCrAlY composite coatings on GH4169 alloy by atmospheric plasma spraying process. The idea of adding oxides was essentially to enhance the oxidation resistance of the composite coatings. Subsequently, the authors conducted tribological studies on the composite coatings in $Al_2O_3$ ball on coated disc contact configuration at 25, 400, and 800 °C respectively. The presence of $TiO_2$/ZnO to the composite coatings was detrimental to their tribological performance at 25 °C but greatly enhanced it at 800 °C. Especially, the NiCrAlY composite coating containing $TiO_2$–ZnO resulted in excellent friction and wear performance at 800 °C. This was attributed to the formation of multiple oxides on both coatings as well as $Al_2O_3$ ball specimens during high temperature sliding.

Lu et al. [836] applied laser cladded Inconel 718 coating in conjunction with V and Cr transition layers with a view to minimise the large differences of Ti and Ni thermal properties and also to avoid undesirable intermetallic compounds on Ti–6Al–4V substrates. The friction and wear characteristics of the coating as well as that of the substrate material were evaluated by using a $Si_3N_4$ ball on coated disc contact configuration at temperatures in the range 30–800 °C under reciprocating sliding condition. The laser cladded Inconel 718 coating showed superior friction and wear behaviour compared to that with the substrate material in the temperature range 30–800 °C. The wear rates of the Inconel coating at temperatures from 600 to 800 °C were almost an order of magnitude lower than that of the substrate material. The authors attributed the decrease of friction in case of coating to the strengthening effect of intermetallics and solid solutions at low and middle temperature and a synergistic lubricating effect among vanadates and other oxides at high temperature. The improved wear performance was ascribed to the formation of interfacial glazed layer at high temperature.

Zhou and Kong [837] applied laser cladded Co-based and $Ti_3AlC_2$ reinforced Co-based coatings on

H13 substrates. The friction and wear performances of the lased cladded coatings were evaluated at 600 °C. The nature of the counterpart material has not been mentioned in the paper. The Co-based coating containing 5% $Ti_3AlC_2$ showed superior friction and wear performance compared to other coatings studied by the authors. The wear was due to oxidative wear, adhesive wear, and abrasive wear mechanisms.

Liu et al. [838] deposited self-lubricating nickel-based coatings on SUS304 by laser cladding and investigated their tribological behaviour in a $Si_3N_4$ ball on coated disc configuration under unidirectional sliding condition at 25 and 600 °C respectively. The application of Ni60–$Ti_3SiC_2$ composite coatings greatly enhanced the tribological performance of 304 stainless steel. The enhancement in tribologial performance at high temperature of 600 °C has been due to the synergistic effect of solid lubricant $Ti_3SiC_2$ with Ni60 oxide films.

Chakraborty et al. [839] applied NiCr based hardfacings having 3 different compositions with Cr content of 10.9, 12.7, and 14.3 wt% on to 316LN stainless steel using gas tungsten arc (GTA) process. Their friction and wear performance was evaluated by using a high temperature high vaccum tribometer. The tests in high vacuum were done with a view to assess the suitability of hardfacings in oxygen-free environment since the hardfacings were intended for use in sodium cooled fast breeder reactor. The NiCr based hardfacing having 12.7% Cr showed superior wear performance compared to the other two under ambient and high temperature-high vaccum conditions.

Günen et al. [840] employed pack boriding treatment at different temperatures and durations with a view to enhance the high temperature wear and oxidation behaviour of Nimonic 80A type Ni based superalloy. The wear performance of both, the untreated and pack borided samples was evaluated at room temperature and 500 °C in a ball on disc configuration using WC ball as the counterpart. The application of boriding treatment to Nimonic 80A type Ni based superalloy considerably improved the wear resistance at both RT as well as at 500 °C. The improvement in wear resistance was more significant at RT and it was due to softening of the surface at higher temperature. The improved wear resistance was attributed to the hard boride phases formed on the borided samples.

Wang et al. [841] applied NiCoCrAlTa/TaC metal matrix composite coating on to a single crystal superalloy (Ni–12Cr–9Co–2Mo–4W–5Ta–4Al–4Ti) by elektrospark deposition and investigated its wear performance at room temperature and 600 °C in $Si_3N_4$ ball and coated disc configuration under unidirectional sliding condition. The presence of TaC particles in the composite coating remarkably improved the microhardness as well as wear resistance of the coating at high temperature.

Wang et al. [842] deposited AlCrVSiN coatings with varying amount of V by using a hybrid process combining arc ion plating and pulsed DC magnetron sputtering and characterised their microstructure, mechanical, oxidation, and tribological properties. The tribological characteristics of the coatings were evaluated by using an $Al_2O_3$ ball on coated disc tests configuration from room temperature to 800 °C. The results revealed that the addition of V into AlCrSiN coatings led the formation of lubricious oxides (e.g. $V_2O_5$) which resulted in sharp decrease in friction coefficient at 600 °C (from 1.0 to 0.56) and at 800 °C (from 0.86 to 0.21).

Wang et al. [843] deposited (Cr, V)N solid solution coatings containing 14 at% V by a cathodic arc evaporation system. Subsequently, the oxidation and tribological behaviours of the coatings were studied with a view to understand the impact of oxidation on the tribological behaviour. The tribological studies were conducted by using an $Al_2O_3$ ball on coated disc contact configuration in the temperature range 500–900 °C under unidirectional conditions. The results showed that the coating started to get oxidised at 600 °C. The oxidation rate increased with an increase in temperature and the mechanical properties deteriorated as the temperature increased. The tribological behaviour in the temperature range 500–600 °C was termed as a dynamic abrasive grain generation, powdering and self-aggregation process resulting in high coefficient of friction (0.57–0.63), and the damage of the adhesion strength at 600 °C giving rise to reduced wear resistance of the coating. In the temperature range 700–900 °C, the increased oxidation led to the formation soft V–O phase that significantly reduced COF (0.28–0.37). This reduction in friction was however accompanied by increased wear because of the depletion of V element in the coating and led to its catastrophic failure at 900 °C.

Liu et al. [844] deposited $Hf_{1-x}Mo_xN_y$ films on 718 Ni alloy plate by using the DC-magnetron sputtering and studied the effect of Mo content on the structural and mechanical properties of the coatings, especially the effect of Mo content on tribological properties at different temperatures. The tribological tests were conducted in a $Si_3N_4$ ball on coated disc test configuration at temperatures ranging from room temperature to 600 °C under unidirectional conditions. The deposited films were characterised by a FCC single solid solution phase structure. This study showed that the COF and wear-rate of $Hf_xMo_{1-x}N_y$ films are almost inversely proportional to its $H/E$ value at RT and elevated temperatures. The film $Hf_{0.44}Mo_{0.56}N$, characterised by higher $H/E$ (0.12) and $H^3/E^2$ (0.60) values showed lower wear-rate ($<1.17\times10^{-5}$ $mm^3/(N\cdot m)$) and COF (0.4–0.6) in the temperature range of RT–600 °C. The formation of friction-induced compact molybdenum oxides gave rise to superior tribological performance of $Hf_{0.44}Mo_{0.56}N$ films at high temperatures (400 and 600 °C).

Muhaffel et al. [845] used multi arc oxidation (MAO) process with and without monoclinic $ZrO_2$ particles in an aluminate-based electrolyte to form hard and adherent $Al_2O_3$ coating on 7075 Al alloy and evaluated their wear performance in a WC ball on coated test configuration at room temperature and 300 °C respectively. The MAO coating synthesized without $ZrO_2$ particles was monolithic $Al_2O_3$ whereas the one synthesized with $ZrO_2$ particles has two distinct oxide layers, the outer layer having $ZrO_2$ particles in the $Al_2O_3$ and an inner layer of monolithic $Al_2O_3$. At room temperature, the MAO–Zr coating showed remarkably lower wear compared to the MAO coating. However, at 300 °C both the coatings got detached from the surface after certain number of cycles depending on the load, with MAO–Zr coating exhibiting superior overall performance as compared to the MAO coating.

Kulkarni et al. [846] explored the suitability of laser-assisted nitriding in open-air with an option of $N_2$ gas purging with a view to enable selective nitriding at a localized sites large and complex shaped components. In their study laser nitriding of an A356 cast alloy (in T6 heat treated condition) was carried out and friction and wear studies were conducted by using a pin on disc configuration with AISI 316L stainless steel pins at different loads. The laser nitriding resulted in the formation of an AlN phase in the laser nitride surface layer which enhanced both the hardness as well as the high temperature fretting wear performance of the A356 alloy.

## 6 Computer simulations in tribology

### 6.1 Introduction of computer simulations in tribology

Computer simulation plays an increasingly important role in tribology research. A search of the literature reveals that a large number of studies related to computer simulation have been published in the last two years. This section reviews the development of computer simulation from both macro and micro levels. From the aspect of macro level computer simulation, the modelling progress and simulation in rough surface, EHL and mixed lubrication, contact problem, wear are mainly involved. From aspect of the atomistic level, five topics are involved including micro-friction and superlubricity of two-dimensional materials, friction and wear behavior of polymeric composites, tribochemical reaction at friction interface, fundamentals of EHL and lubricant design and fundamentals of friction & wear behavior. The studies reviewed in this section are mainly from a search of literatures in the period of 2020–2021. However, due to the huge amount of important contributions in this area, only parts of them have been reviewed.

### 6.2 Simulation of EHL and mixed lubrication

#### *6.2.1 Point contact lubrication problem*

In real engineering products, mixed lubrication may prevail due to the surface roughness, and variation of operating conditions. Many efforts have been made to model the mixed lubrication problem. There are basically two catalogs for mixed lubrication models: Stochastic and deterministic. Stochastic models use stochastic parameters to represent the surface roughness and its effect on lubrication performance is evaluated with the aid of flow factors, which are obtained by separate deterministic sub-models. Deterministic models directly use rough surface data and can obtain the detailed information in lubricated contact area which plays a key role in the design of products. There are several successful stochastic models [847] and deterministic model of mixed lubrication [848]. In mixed lubrication, there are basically two sub-interfaces between solids in direct solid contacts, and the sub-interface between the lubricant and the surfaces in lubricated zones, and between them, namely, the lubrication–contact interface, the pressure should be continuous. The reduced Reynolds equation proposed by Hu and Zhu [848] achieves unification between boundary lubrication and EHL in the physical meaning, when the local film thickness is less than the threshold, the Poiseuille flow of the left-hand terms will disappear and the reduced Reynolds equation is solved for contact pressure. For numerical implementation, the pressure term is extracted from the film thickness and forms an iterative equation. The mathematical understanding of the reduced Reynolds equation is to set film thickness constant by changing pressure. However, the convergence and accuracy of this differential scheme are not ideal. In order to improve the accuracy and efficiency of unified Reynolds equation method proposed by Hu and Zhu [848], Zhang and Zhang [849] put forward a straightforward but robust approach to solve pressure in the boundary lubrication region. They directly take $h = 0$ as the governing equation of contact area and form the iteration equation for local contact node. Instead of using continuous pressure boundary condition, they use zero pressure conditions at the border between the EHL region and asperity contact region, which is proven efficient and robust to simulate mixed lubrication with a complicated rough surface, for both oil-based lubrication and water-based lubrication. However, in mixed lubrication, the lubricant may be entrapped in valleys surrounding by asperities and still withstand a tiny fraction of load. The reasonable boundary condition for the pressure between lubricated area and solid–contact area should be that the lubricant pressure there should not surpass the pressure at the solid–contact side. Otherwise, the lubricant would be able to penetrate and open the contact junction before reaching equilibrium.

Liu et al. [850] revisited the lubrication–contact interface conditions (LCICs) from the flow continuum point of view and described the interface conditions explicitly with mathematical equations and inequalities, focusing on the flow continuum. By careful investigation, they found that LCICs were ignored in most related models, and therefore, the flow continuum cannot be guaranteed. In order to make sure that there is no net flow entering into the solid-contact zones, nor net flow coming out from these zones, they present the expressions for the Couette flows entering or leaving a control volume with different differential schemes. A set of LCICs, including nullifying flows and pressure inequalities, are presented, and a new method to determine solid-contact pressure is proposed with the detailed treatment of Couette flows around the interface. Based on these derived LCICs and their corresponding treatments, the extra-flow problems in solid-contact zones are eliminated and the flow continuity is confirmed, therefore, more precise mixed lubrication results can be obtained as shown in Fig. 49.

Another issue in lubrication problems is flow continuity. The foundation of hydrodynamic lubrication theory is Reynolds equation which is derived based on flow continuity and has to be numerically solved by iterative methods for most cases. Two convergence criteria are commonly used to end the iteration process, one is an error control for pressures, and the other is load balance. In fact, as stated by Liu et al. [851] that there is no work to explicitly consider flow continuity in its iteration process, and they found that the flow continuity is not well guaranteed in most solutions, specially, the degree of flow continuity in the inlet region is very poor and the entire iteration process is slowed down due to the consistent use of under-relaxation iteration in the whole computation domain. They proposed a novel approach by using different relaxation factors in inlet, transition, and high pressure/exit regions and introducing the error control of flow continuity, as a result shown in Fig. 50, not only flow continuity is guaranteed, but also the accuracy and convergence speed are improved.

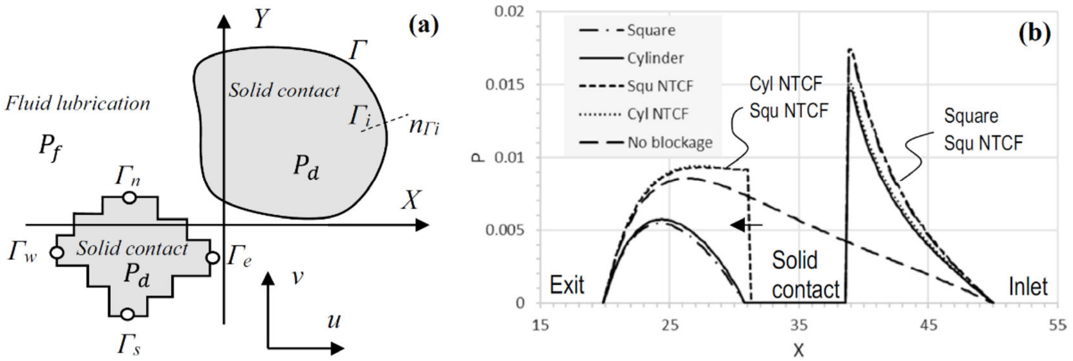

**Fig. 49** A point-contact lubrication problem. (a) Simulation region with lubrication–contact interfaces and (b) comparison of pressures. The "NTCF" with the 1B scheme allows the Couette flow exchange (see arrows) when the fluid lubrication zone is on the left-hand side of the solid-contact zone [850]. Reproduced with permission from Ref. [850], © The author(s), 2021.

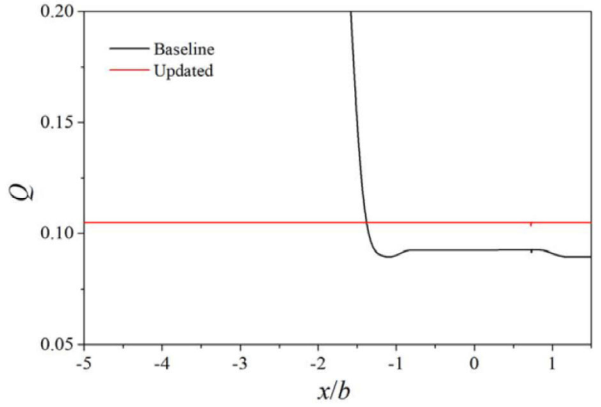

**Fig. 50** Comparing results with and without iteration update process including the error control of flow continuity [851]. Reproduced with permission from Ref. [851], © American Society of Mechnaical Engineers, 2021.

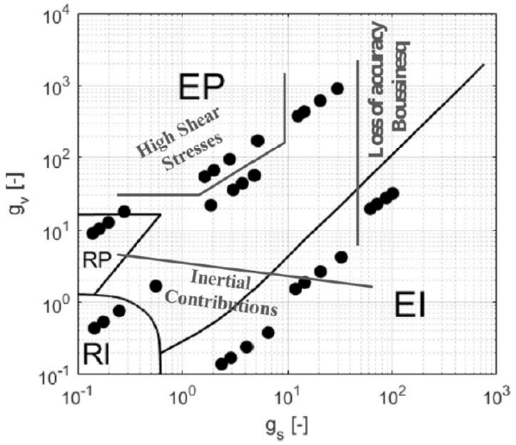

**Fig. 51** Main causes of the discrepancy between the RBM and the NS approach [852]. Reproduced with permission from Ref. [852], © American Society of Mechnaical Engineers, 2021.

Reynolds and Navier–Stokes based modelling approaches are the two main approaches to predict the pressure and fluid film thickness distribution throughout the contact. Scurria et al. [852] assessed both methods for different lubrication conditions in a wide EHL conditions and quantified their differences to understand advantages and limitations of both methods. They conclude that as shown in Fig. 51, for the cases with low loads and relatively high velocity of the surfaces, loss of accuracy is exhibited due to the lack of inertial contributions in the Reynolds equation; for quasi-rigid EHL cases with high loads, high shear stresses approach the value of the singular shear stress criterion in the central area of the contact and have a mild impact with respect to accuracy; for highly loaded soft EHL cases, the Reynolds based method has the loss of accuracy due to loss of accuracy of the Boussinesq equation due to large deformations, while it becomes less important, but still evident, for hard EHL.

To explore the mechanism of abnormal pressure distribution that the groove pressure can be higher than the ridge pressure when the longitudinal roughness of both contacting bodies has the same wavelength and ridges come into the in-phase contact, Kaneta et al. [853] conducted the thermal EHL analysis in point contacts composed of different thermal and mechanical materials with the longitudinal roughness, they found that this abnormal phenomenon may occur when the speed of the material having low thermal conductivity is higher than that of the material having high thermal conductivity and also the overall film thickness is neither thick nor thin, and caused when the fluid flowing from the ridge into the groove in the entrance region is suppressed by that in the exit region.

Liu et al. [854] proposed a simplified traction force prediction model for highly loaded rolling/sliding EHL contacts that considers the rheological characteristics of lubricants without solving the Reynolds equation and elastic deformation. The model uses the Hertz pressure distribution as the pressure distribution of the contact zone, and the film thickness is calculated by the Hamrock Dowson (HD) formula, avoiding the numerical solution of the Reynolds equation. The temperature field of the contact zone is obtained by solving a simplified energy equation. In order to consider the shear thinning effect of lubricating oil, the power law Carreau equation is adopted. Figure 52 shows the model prediction results and experimental results under different working conditions. At low speed, the model prediction results fit well with the experimental results, and deviate greatly from the experimental results when the speed is higher. In addition, for heavy-load cases, there is always a large deviation between the model prediction results and the experimental results.

Wu [855] presented a multi-scale method to explore the tribological behaviors of a mono- or multi-layer molecular thin fluid film confined by two solid substrates, which uses an atomistic description for the near region and a coarse-grained description for the far region of the solid substrate. The multi-scale method has the potential to be directly applied to some realistic models such as the lubricated sliding of two metal substrates.

Bearing steels are strongly non-homogeneous and anisotropic within a sufficiently small range. However, existing EHL models mostly treat solids as homogeneous materials with three-dimensional isotropics. In order to be able to assess the criticality of specific aspects of microstructure, Zhang et al. [856] established the EHL lubrication model of three-dimensional anisotropic heterogeneous materials as shown in Fig. 53. The lubrication problem of three-dimensional anisotropic materials can be solved based on the Navier–Cauchy equation and the Reynolds equation. From the simulation results, the three-dimensional anisotropic material will not affect the thickness profile and size of the film, but will affect the pressure distribution.

### 6.2.2 *Line contact lubrication problem*

Habchi [857] presents a finite element model for the solution of thermal elastohydrodynamic lubrication in finite line contacts, in which the surface deformations are evaluated by finite element method and the finite length in the axial direction for both solids is considered. They investigated the effect of roller-end axial profiling on the frictional behavior and found that roller-end axial profiling has a negative effect on friction but positive on minimum film thickness. It concludes that the improvement in fatigue life through end profiling comes at the expense of increased friction. As the functionally graded material (FGM) coating has received considerable attention for improving the surface contact damage, Su and Ke [858] presented an FGM coated EHL line contact model, in which the inhomogeneous characteristics of the coating are modeled in the thickness direction by an exponential function. It is found that the distributions of the fluid pressure can be changed by

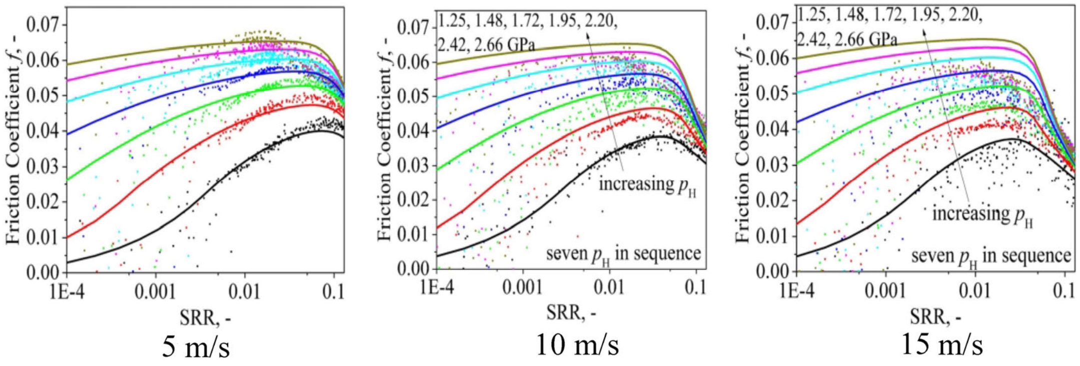

**Fig. 52** Traction curve comparison between simplified traction calculation and twin-disc measurements [854]. Reproduced with permission from Ref. [854], © Elsevier Ltd., 2020.

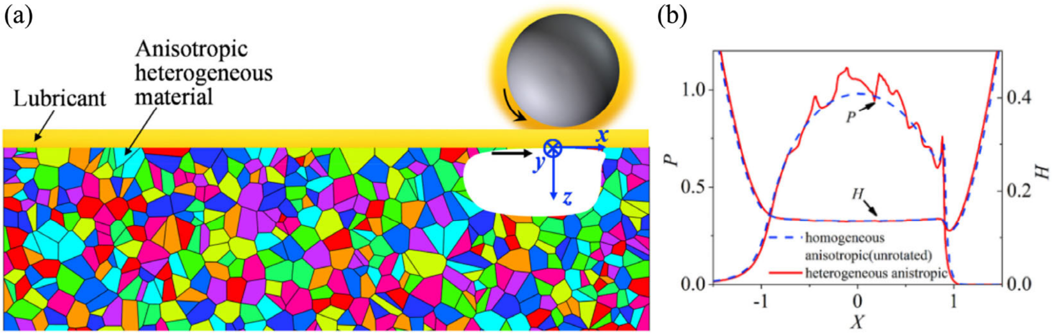

**Fig. 53** Schematic representation of (a) an elastohydrodynamically lubricated contact between a sphere and a polycrystalline anisotropic material and (b) comparison between isotropic and anisotropic materials [856]. Reproduced with permission from Ref. [856], © Elsevier Ltd., 2020.

modifying the stiffness ratio of the FGM coating.

The COF valley zone of the Stribeck curve obtained on smooth surfaces suggests that the variations of COF closely depended on the physical and chemical properties of the confined lubricant layer. Zhang et al. [859] proposed a thermodynamics model to analyze the Stribeck curve of a lubrication system, for which an entropy production model was introduced by combining with interfacial disjoining pressure function and interfacial free energy. It reveals that as the film thickness of the lubricant decreases to nanometers, the friction force resulted from interfacial interaction becomes significant and dominant. They conclude that smaller friction coefficient within the valley zone on the Stribeck curve may be resulted from a stronger interfacial interaction, and may be tailored by modifying the interfacial interaction as shown in Fig. 54.

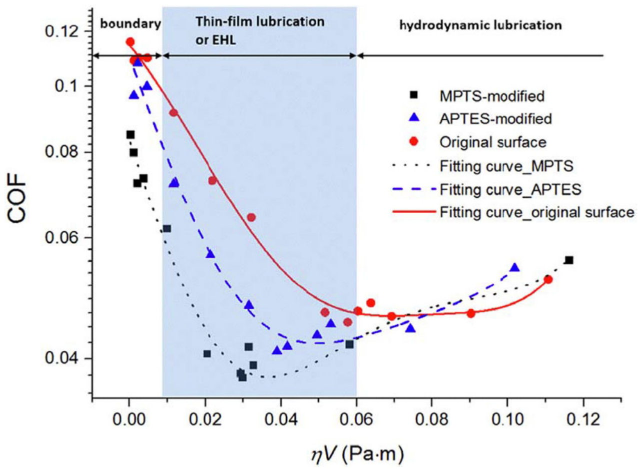

**Fig. 54** Stribeck curves on different substrate surfaces [859]. Reproduced with permission from Ref. [859], © American Society of Mechnaical Engineers, 2020.

Peterson et al. [860] present a partitioned strongly coupled fluid–solid interaction (FSI) model to solve the 2D elastohydrodynamic (EHD) lubrication problem, coupling a special FE domain in ABAQUS with the control volume finite-difference formulation of the Reynolds equation, in an effort to make up for the shortcomings of classical EHD lubrication models lacking the necessary flexibility to fully characterize the lubricated contacts, as shown in Fig. 55. The model has the potential to conveniently consider plasticity, contacting surfaces, material microstructure and damage. In addition, Singh et al. [861] also used N–S equations to describe the fluid flow to avoid the requirement of Reynolds assumptions being applicable, therefore the model can provide a detailed solution of the fluid flow behavior (from CFD results) and the structural response (from FEM results) simultaneously for a wide range of contact conditions with reasonable computational efficiency.

Lampaert and van Ostayen [862] proposed an effective numerical simulation method to describe the pressure in a Bingham plastic fluid lubrication film. The derivation uses the 'exact' Bingham plastic fluid rheology model in combination with only the approximations required for the general Reynolds equation. In addition, since the regularization technique is no longer required, the method is computationally efficient and can be applied to lubricating films of any geometry. Figure 56 compares the simulation results of the model, the results of the CFD simulation, and the results obtained by the calculation using regularization techniques, the accuracy of the model

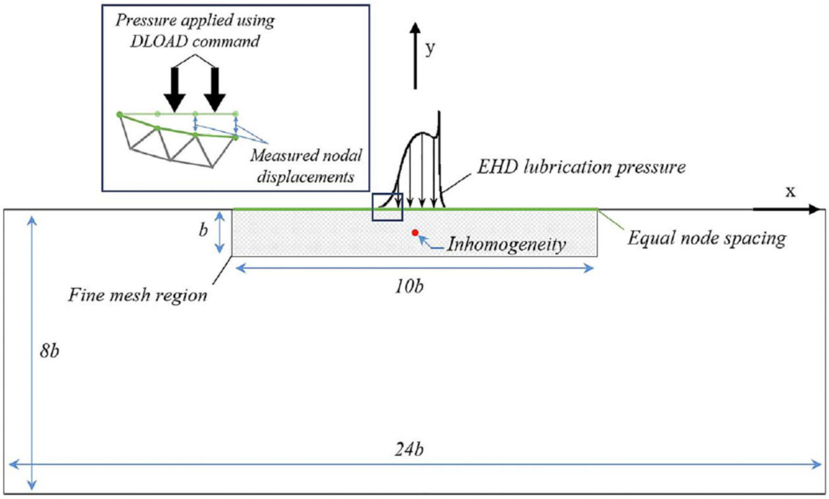

**Fig. 55** 2D FE domain schematic with dimensions, load application, and surface deflection measurement (EHD lubrication pressure half width may vary based on the prescribed load) [860]. Reproduced with permission from Ref. [860], © American Society of Mechnaical Engineers, 2020.

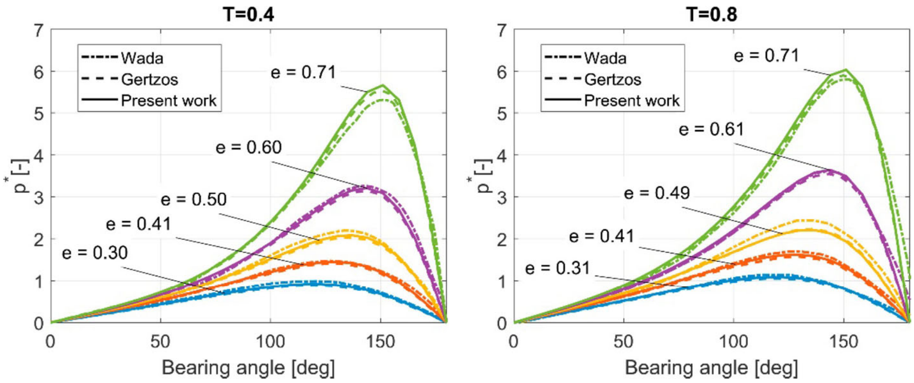

**Fig. 56** Press curve comparison between present model and other two models [862]. Reproduced with permission from Ref. [862], © Elsevier Ltd., 2020.

is close to that of the other two methods, but the efficiency is higher than them.

Spiral groove thrust bearings (SGTBs) have been applied in high-speed rotating machinery and high-precision instruments due to their significant hydrodynamic and self-pumping effects. When the SGTB is running at high speed, the lubricant will change from laminar flow state to turbulent state, there will be severe cavitation. In addition, excessive centrifugal force at high speed will make the lubricant throw out and affect its performance. For the water-lubricated SGTB operating at a high speed, a heterogeneous two-phase flow model should be established to describe its complex cavitation behavior. Based on the gas-liquid two-phase flow, Zhang et al. [863] established a water lubrication hydrodynamic SGTB turbulent lubrication model that considers the influence of cavitation and centrifugal forces. They assumed that the cavitation flow consisted of a continuous liquid phase and discrete spherical bubbles filled only with steam. For the liquid phase part, considering the centrifugal effect of the fluid and the circumferential interface effect, the equivalent Reynolds equation was derived using the finite volume method. It was found that the lubrication model considering cavitation and centrifugal forces was closer to the experimental results as shown in Fig. 57.

To conduct accurate and efficient analysis of the nonlinear dynamics of high-speed tilting-pad journal bearings (TPJBs) and low-viscosity-fluid-lubricated TPJBs, Jin et al. [864, 865] modified the database method (DM) to rapidly determine the nonlinear

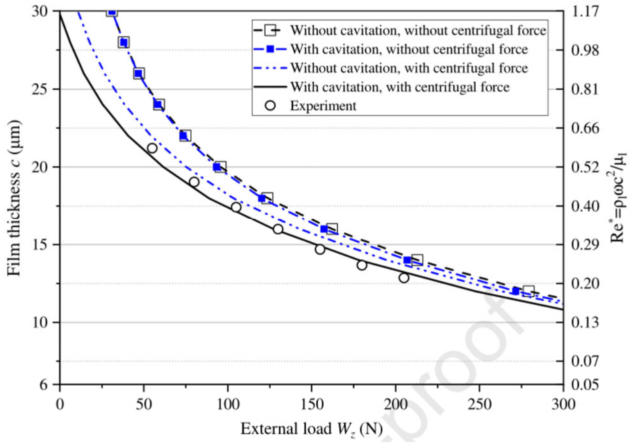

**Fig. 57** Film thickness curve comparison between the model calculation and experiments [863]. Reproduced with permission from Ref. [863], © Elsevier Ltd., 2020.

fluid film force (NFFF) of a TPJB while considering turbulent and thermal effects. An NFFF database with high accuracy and large capacity is compiled, and a combined linear and parabolic interpolation polynomial of the NFFF for a single pad is constructed. As an example, the DM is applied to perform nonlinear dynamic calculations of a water lubricated TPJB–rotor system.

Gu et al. [866] studied the performance of a journal bearing subjected to impulse loading based on transient mixed lubrication model considering mass conservation and the effects of asperities on flow and contact, and developed a novel journal bearing surface design methodology. Also, the soft elastohydrodynamic lubrication model was applied to evaluate the effect of microtexture design parameters and bearing operating conditions on the resulting lubricant film thickness and performed the optimal design [867]. Thielen et al. [868] presented a transient thermo-elastohydrodynamic lubrication (TEHL) model for the contact of radial shaft seals in order to consider the roughness or microstructure of both contact surfaces with transient effects. In contrast to most other EHL models for radial shaft seals, the deformation calculation is based on the Boussinesq theory, not on FE. Yang and Palazzolo [869, 870] presented a computational fluid dynamics (CFD)-based approach to model the TPJB, which can consider thermo-elastohydrodynamic (TEHD) effects with multi-mode pad flexibility. They demonstrated that the conventional approach of disregarding the three-dimensional flow physics between pads (BP) can generate significantly different pressure, temperature, heat flux, dynamic viscosity, and film thickness distributions, then they presented the Reynolds based TEHD TPJB model with mixing coefficient (MC) obtained based on machine learning, which has an accuracy nearly the same as the complete CFD model, but with better computational economy.

In actual engineering, multi-directional mechanical vibration of the transmission system is universal and inevitable. Predicting and understanding the dynamic contact stiffness evolution of the transmission mechanism is of great significance for the design and dynamic simulation of the transmission mechanism. In order to study the lubrication dynamic contact stiffness of spiral bevel gears (SBG) under periodic fluctuating loads and speeds, Wang et al. [871] established a transient hybrid lubrication contact stiffness model. Through a series of studies, the dynamic contact stiffness of point contact under the action of periodic fluctuating loads, coupling periodic fluctuating loads, and velocities were revealed.

Also, the calculation of the stiffness and damping coefficients of hydrodynamic journal bearings is an important issue and the widely used approach for approximating the stiffness and damping is the so-called infinitesimal perturbation (IP) method, whose boundary condition usually is to set the perturbation pressure to zero at boundary curves of oil film zone. However, this boundary condition ignores the moving of oil film zone after the perturbation applied. Chen et al. [872] proposed an improved IP method with modified boundary condition (IPMMB) is to calculate the stiffness and damping with considering the fluid film boundary migration (FFBM). They found that FFBM has a significant influence on dynamic coefficients of full circular journal bearing but little impact on journal bearing with axial grooves. Furthermore, it has great effect on the stiffness and damping for full circle bearing with large length-to-diameter ratios or small eccentricity ratios.

### 6.2.3 *Lubricant theory and design*

In EHL, lubricants are subjected to pressures of more than 500 MPa and strain rates of more than $10^5$ $s^{-1}$ [873]. High pressure results in a sharp rise in Newtonian viscosity, while high velocity results in large shear stress and significant shear thinning, which bring challenges to the design of engineering moving parts working in this regime. In the past two years, molecular dynamics simulation continued to contribute in this field, both independently and by coupling with macroscale EHL models.

Kadupitiya and Jadhao [873] used non-equilibrium molecular dynamics (NEMD) simulations to extract accurate rheological properties of lubricants under EHL conditions, and innovatively used machine learning to analyze and visualize the high-dimensional results generated in output data of typical NEMD simulations (Fig. 58(a)). After dimensionality reduction of NEMD simulation data for squalene shear flow, they found that shear flow transitions from Newtonian to non-Newtonian shear thinning with increasing shear rate, providing a correlation between shear thinning and evolution of molecular orientation. One of the key issues in EHL is the model that best describes the non-Newtonian response of lubricants at high shear rates. Santak and Conduit [874] developed a sampling algorithm based on NEMD simulations. They modified the Carreau model by weighted least squares regression and tested the Newtonian viscosities of 74 alkanes with 5–10 carbon atoms at high shear rates ($10^9$–$10^{12}$ $s^{-1}$) whose results were estimated in good agreement with the experiment. Ewen et al. [875] systematically reviewed friction and lubrication studies under EHL conditions based on MD simulations, mainly including the progresses in shear thinning, high pressure Newtonian viscosity, etc. The review outlined the fundamental understanding of molecular behavior under EHL condition that was gained via molecular dynamics simulations, as well as how models were improved to better predict macroscopic behavior.

In addition to the above research contributing to the lubrication theory, atomic simulation also has many explorations in the research and design of lubricants. Albina et al. [876] performed coarse-grained molecular dynamics (CGMD) simulations to study the friction properties of lubricant molecules on nanostructured metal surfaces. By acting as attraction centers for lubricant molecules, the fine grain boundary structure was thought to prevent oil film delamination, thereby improving friction and wear performance. Kajita et al. [877] proposed an

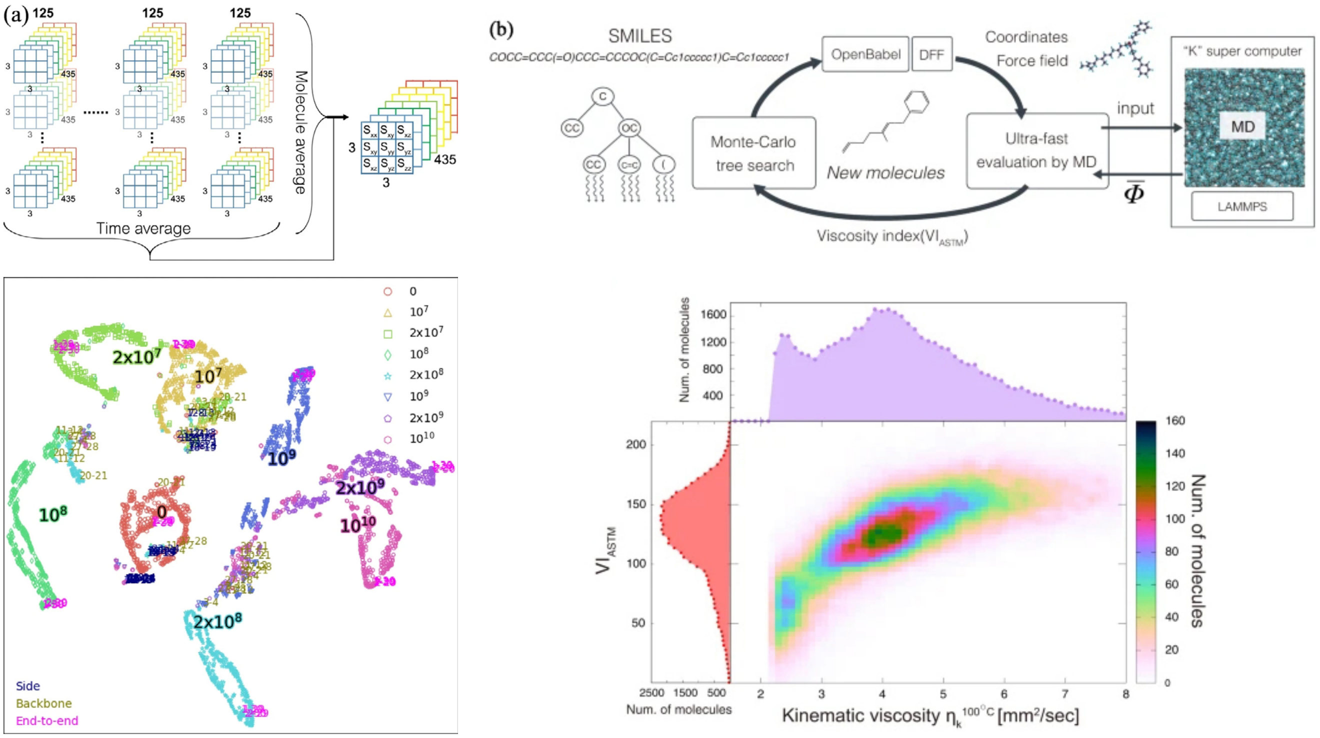

**Fig. 58** Molecular dynamics and machine learning methods in EHL simulation [873, 877]. Reproduced with permission from Ref. [873, 877], © The author(s), 2021 and The author(s), 2020, respecitively.

autonomous molecular design system based on Monte Carlo Tree Search (MCTS) and MD simulations (Fig. 58(b)). They used stress tensor correlation to significantly speed up viscosity evaluation (by three orders of magnitude) and screened 55,000 lubricant-sized molecules for their viscosity–temperature behavior. The combination of simulation and physical models in this study provided new ideas for the design of high viscosity index lubricating oil molecules. There is a trend of machine learning in combination of molecular dynamics and other simulation methods in tribology area to accelerate materials selection and design, which should continue to play an important role in the future.

The hydro-dynamically lubrication can help to increase the separation between the sliding surfaces and reduce friction and wear. The shape optimization and introduction of surface textures are efficient methods to improve the lubrication of tribological devices. Hence, Codrignani et al. [878] present the implementation of the adjoint method for the optimization of surface textures in hydrodynamically lubricated contacts with cavitation. This method allows to fine-tune each single texture element individually and carry out free-form optimizations in a computationally efficient manner. The performance of the adjoint optimization for different constraints is shown in Fig. 59.

## 6.3 Simulation in contact problem

The working of mechanical equipment is accompanied by the mechanical contact of parts, thus, the research on the contact problem is essential in the safety design of machine structure and energy-saving design. With the rapid development of computer technology, computer simulation has become an effective method to study complex contact problems. The simulation study of material contact seeks the numerical solutions of the contact area, contact pressure, deformation, and stress of two contact bodies, and these obtained results can be used to predict the failure or guide the structural design of the part. The material of the contact bodies may be various, homogeneous or non-homogeneous; isotropic or anisotropic; elastic, elastoplastic or viscoelastic; etc. The form of contact may be flat contact, line contact, or point contact. Furthermore, the contact of parts may involve the effects of multi-physical fields, including elastic, thermal, electric, magnetic, and so on. Many researchers have conducted simulation studies on the contact problems of parts under various materials, working conditions, and contact forms.

### 6.3.1 *Extension of contact model*

At present, the contact models are developed to be closer to engineering practice. In soft contact and

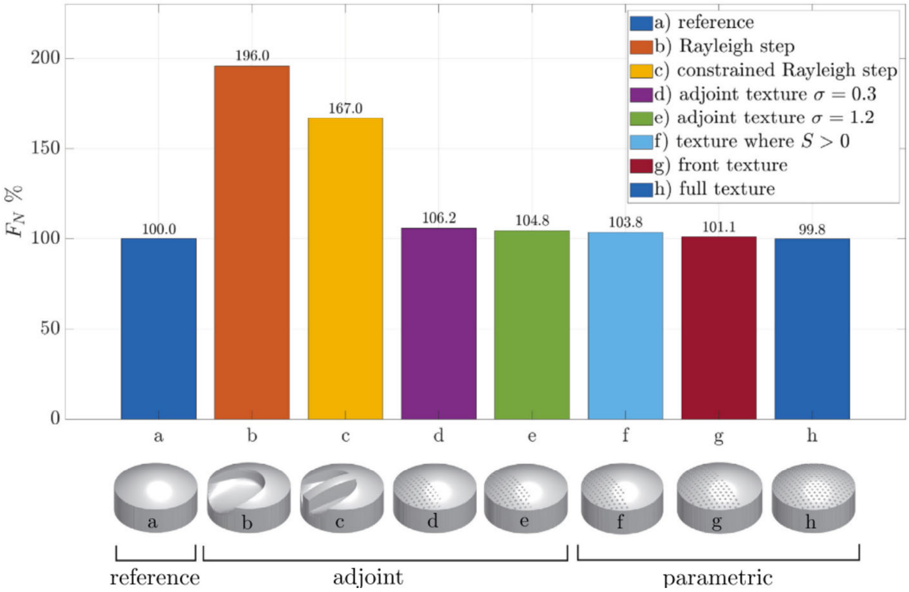

**Fig. 59** Performance of the adjoint optimization for different constraints [878]. Reproduced with permission from Ref. [878], © The author(s), 2020.

conformal contact, the classical Hertzian contact theory has certain inapplicability resulting in inaccurate calculation results. The Kelvin–Voigt model is another method but suffers from a discontinuity existing in its constitutive law. Askari [879] proposed a concept to simulate either soft or conformal contacts and developed mathematically closed-form contact models, which were nonlinear and easy-to-implement while resolving the discontinuity issue with the Kelvin–Voigt model. Chauda and Segalman [880] presented a novel contact algorithm, the method of first violation (MFV), based on the principle of first contact status violation, shown in Fig. 60. In this method, the role of contact history is emphasized and the capability of the MFV to capture and accommodate that history dependence is discussed. The MFV algorithm predictions compared well with both analytic solutions and conventional algorithms. By considering the dry contact problem as a static equilibrium where a dynamic interaction process takes place before the static equilibrium is reached, Zhang et al. [881] proposed a semi-analytical approach for the elastic loading behavior of rough surfaces, the contact pressure is obtained based on determining the geometric overlap during loading as shown in Fig. 61 and analytical relationship between deformation and pressure obtained by introducing the concept of "boundary node". Yuan et al. [882] introduced a model for tangential contact problems with surface stress. The effects of surface stress on the spherical contact problem under combined normal and lateral loads were investigated and the contact analysis with partial slip was conducted. These works can provide a theoretical basis for the study of contact and friction of some soft materials and biological systems.

The contact between an elastic ball and a flat is a simple case of mechanical contact problem, which is often used in model studies of adhesion and friction. Many experimental studies have been conducted for the adhesive contact between an elastic ball and a flat,

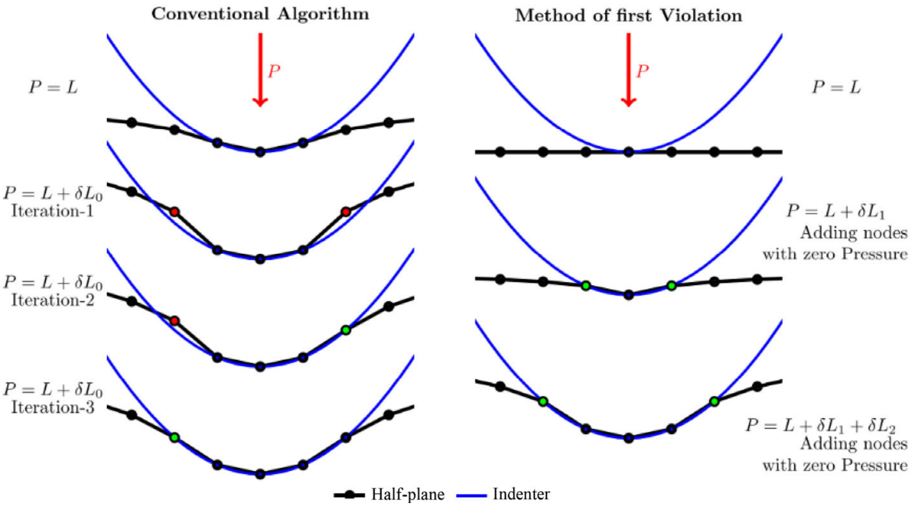

**Fig. 60** Difference between the conventional algorithm and method of the first violation. With conventional contact algorithms, a load step is selected and an iterative procedure is used to find an admissible set of loads and displacements consistent with that load step. With the MFV, the load step is computed to be exactly that necessary to bring the first node to change status (contact, non-contact, stick, and slip). Reproduced with permission from Ref. [880], © Elsevier Ltd., 2021.

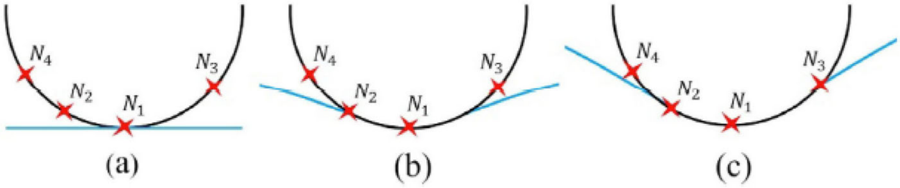

**Fig. 61** Contact process of a rigid rough surface on an elastic half-space. (a) Before the load is applied, (b) two nodes in contact, and (c) three nodes in contact [881]. Reproduced with permission from Ref. [881], © The author(s), 2020.

however, the relevant theoretical studies are lacking especially for the dependency of the contact area on the sliding speed. Hence, Wang et al. [883] used molecular dynamics to study the contact between an elastic slab with a flat surface and a rigid, cylinder corrugated substrate, as shown in Fig. 62(a). Through molecular dynamic simulation, the dependency of the contact width on the sliding speed is discussed, as shown in Fig. 62(b).

Resolving atomic scale details while capturing long-range elastic deformation is the principal difficulty when solving contact mechanics problems with computer simulation. Monti et al. [884] presented a numerically efficient dynamic Green' function technique to treat realistic time-evolving, elastic solids, which can substantially accelerate molecular dynamic simulations of large elastic substrate.

The half-space assumption is very common in the study of contact problems, however, the contact body has a finite length in fact. The half-space assumption will lead to calculation errors, especially in the vicinity of the edge. The quarter-space model was proposed to solve this problem well, but the original quarter-space model only had one free-end face. Constructing two or four free end faces by overlapping quarter-space model would require a lot of complex calculations [885]. Liu et al. [886] proposed a mixed discrete-continuous convolution with duplicated padding and fast Fourier transform (DCD-FFT) method which is modified from discrete convolution and fast Fourier transform (DC-FFT) method with duplicated padding is utilized to deal with the potential free-end-surface effect. This method agrees well with the overlapping method [885] and does not require a lot of complex calculations. The difference between DCD-FFT and the overlapping model is shown in Fig. 63 [886].

Zhang and Etsion [887] developed a coupled Eulerian–Lagrangian (CEL) model with explicit dynamic analysis and power-law hardening to resolve elastic–plastic spherical contact problem. The CEL model avoids convergence problem caused by excessive distortion of elements in Lagrangian models with much improved efficiency. It may provide alternative method for yet unsolved simulation problems in contact mechanics.

Riva et al. [888] presented a simulation methodology based on the FEA that involves computing the global coefficient of friction (COF) of an entire brake system starting from the local COF determined from a pv-map for each local sliding velocity and contact pressure determined by the FEA; as a result, the frictional properties of the macroscale-level rough surface are determined by the mechanical behaviors of nanoasperity contact pairs under shear. Hu et al. [889] presented a multiscale study of the dynamic friction coefficient due to asperity plowing, they first used molecular dynamics simulations to study the non-adhesive shear between single contact pairs; then, by integrating the frictional behavior of a single contact pair into a Greenwood–Williamson–type statistical model, the friction coefficient of rough surfaces is estimated.

With the rapid development of artificial intelligence (AI), its application in tribology becomes prevailed. To overcome the limitations of currently available

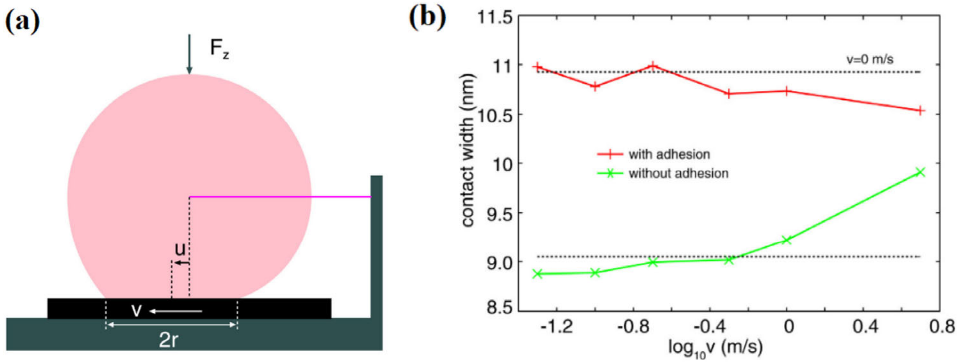

**Fig. 62** (a) An elastic ball squeezed against a flat and rigid substrate. (b) the contact width as a function of the logarithm of the sliding speed [883]. Reproduced with permission from Ref. [883], © Elsevier Ltd., 2020.

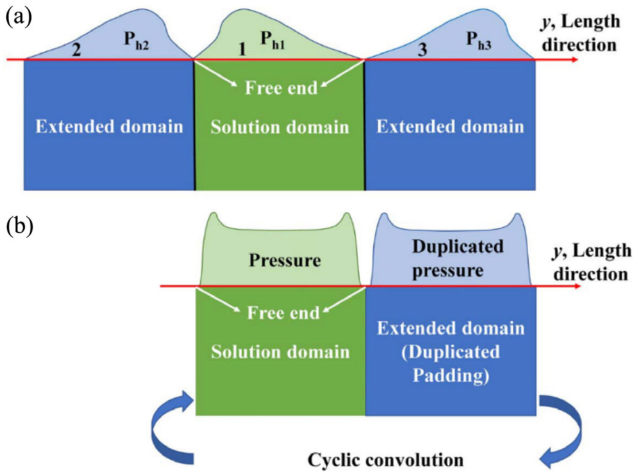

**Fig. 63** Modelling pressure-induced horizontal-surface normal deformation of a finite roller. (a) Overlapping model and (b) DCD-FFT method [886]. Reproduced with permission from Ref. [886], © American Society of Mechanical Engineers, 2020.

models in ascertaining the effects of the physicochemical processes and phenomena involved in nanoscale contacts, based on the acquired experimental data, Perčić et al. [890] used data mining, incorporating various state-of-the-art machine learning (ML) numerical regression algorithms, to model friction in the nanometric domain. They concluded that the black box ML methods provided effective predictions with good accuracy levels, but the intrinsic nature of such algorithms prevented their usage in most practical applications, while the AI-based symbolic regression models allowed attaining an excellent predictive performance with the respective prediction accuracy, based on which an extremely simple functional description of the multidimensional dependence of nanoscale friction was obtained. Xie et al. [891] presents an integrated finite element and artificial neural network (ANN) method to predict friction coefficients during scratch. They used the results from finite element analysis of scratch process to train the ANN and the outputs of network were in agreement with the finite element results as shown in Fig. 64. This model provides assistance for the prediction and analysis of complex relationships between scratch process parameters and variables related to material deformation, and between the plowing friction coefficient and the relevant parameters.

Ozaki et al. [892] phenomenologically derived the evolution law of friction coefficient (real contact area) based on the consistency condition, and then formulated a rate-, state-, and pressure-dependent friction model based on the elastoplastic theory with introducing a nonlinear sliding surface. The model can represent various frictional sliding behaviors, including stick–slip motion, however, the hysteresis friction owing to the viscoelastic property is not considered.

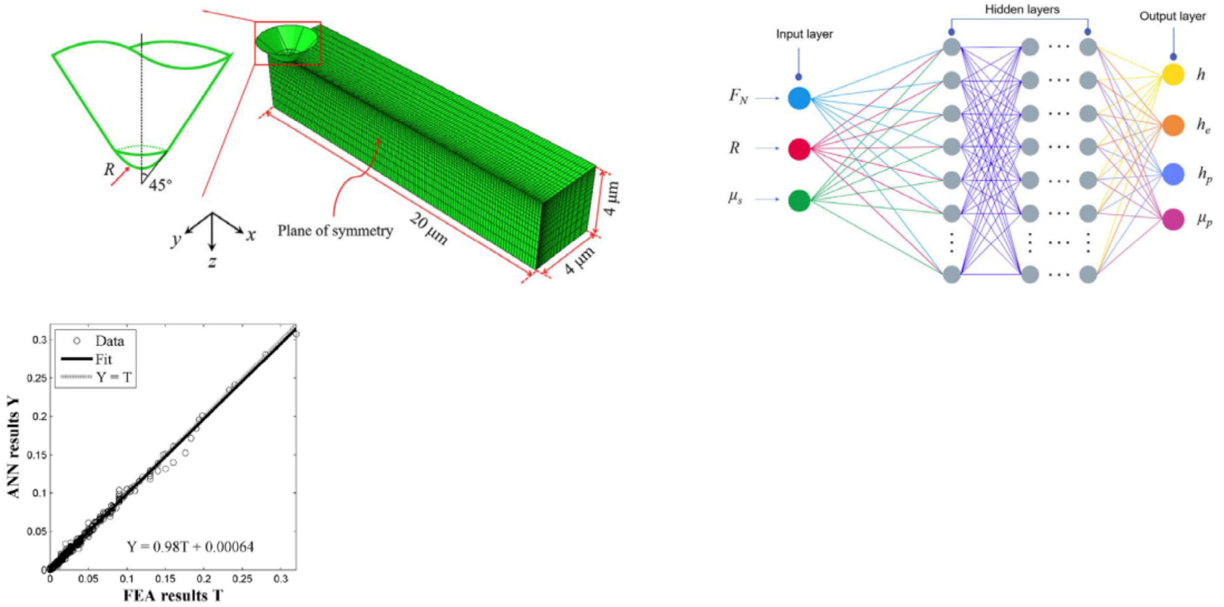

**Fig. 64** FE model of scratch, ANN model for scratch simulations, and ANN training regression analysis [891]. Reproduced with permission from Ref. [891], © American Society of Mechanical Engineers, 2019.

### 6.3.2 *Contact problems of inhomogeneous and layered materials*

#### 6.3.2.1 Inhomogeneous materials

In engineering practice, most of the materials are inhomogeneous, and part materials may be mixed and doped inclusions to meet certain requirements. Therefore, the research on the contact problem of inhomogeneous materials is important for engineering applications and materials design. Zhao et al. [893] derived the analytical solution for the elastic field due to a cuboidal inclusion in a transversely isotropic full-space material and proposed a numerical method to model inclusions of any arbitrary shapes. Sun et al. [894] proposed a novel numerical model for simulating the contact of machined cylindrical components containing inhomogeneity via extending the concept of the 3D line-contact fast Fourier transform (FFT) algorithms as shown in Fig. 65. This model can efficiently solve the three-dimensional axial periodic contact problems of inhomogeneous materials, whose structural features in contact are confined in a finite domain in one direction but extended infinitely in the other direction. Further, Sun et al. [895] proposed a novel numerical model for simulating the elastoplastic contact between nominally flat surfaces of materials containing inhomogeneity as shown in Fig. 66. The model is developed via extending the concept of the DC-FFT algorithm with double superposition of influence coefficients, which is named the DCSS-FFT algorithm.

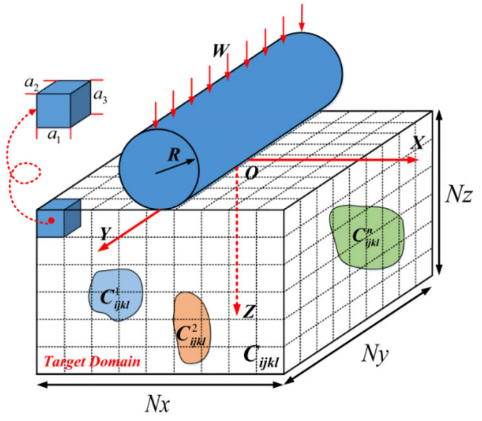

**Fig. 65** A line-contact model of inhomogeneous material subjected to line-contact loading [894]. Reproduced with permission from Ref. [894], © Springer-Verlag GmbH Germany, part of Springer Nature, 2020.

Based on the elastoplastic contact model, Li et al. [896] investigated the relationships between contact yield initiation and case-hardening parameters, for circular, rectangular, and elliptical contacts. A group of formulas relating contact yield initiation, in terms of the critical pressure, location of the first yield, and plasticity index (transition to plasticity) to case-hardening parameters are derived to facilitate contact element designs using case-hardened materials. Guo et al. [897] presented an explicit matrix algorithm to solve the problem of an elastic wedge with three loaded surfaces through converting original surface loads to equivalent loads in half-space, as shown in Fig. 67. Correction factors for the deformation at the edge and the center of Hertzian point load for different distances of the load from the free edge are expressed with empirical formulae.

#### 6.3.2.2 Layered materials

Various coatings are increasingly used to protect the contact surfaces of engineering components. Thus, the contact problems of layered materials have attracted much attention.

Zhang and Wang [898] investigated the thermoelastic frictional contact problems of an elastic sphere sliding on an elastic layer–substrate system. Dislocation-like imperfect interfaces are considered and significant effects on contact pressure, temperature, and stress are observed as shown in Fig. 68. Further, Zhang et al. [899] also investigated the transient and steady-state viscoelastic contact responses of layer-substrate systems with interfacial imperfections. Çömez et al. [900] investigated the continuous and discontinuous contact problems of a homogeneous piezoelectric layer pressed by a conducting rigid flat punch, as shown in Fig. 69. They also investigated the frictional receding contact problem of a functionally graded (FG) layer resting on a homogeneously coated half-plane [901], and then on a Pasternak foundation [902]. In order to improve the uniformity of the contact pressure, Lin et al. [903] developed a contact interface material stiffness optimization design algorithm based on the modified solid isotropic material with the penalization (SIMP) method, and a node-to-node frictionless elastic contact theory was adopted to perform the contact analysis. Wang et al. [904] simulate the flattening process of a coated single asperity by a rigid flat based

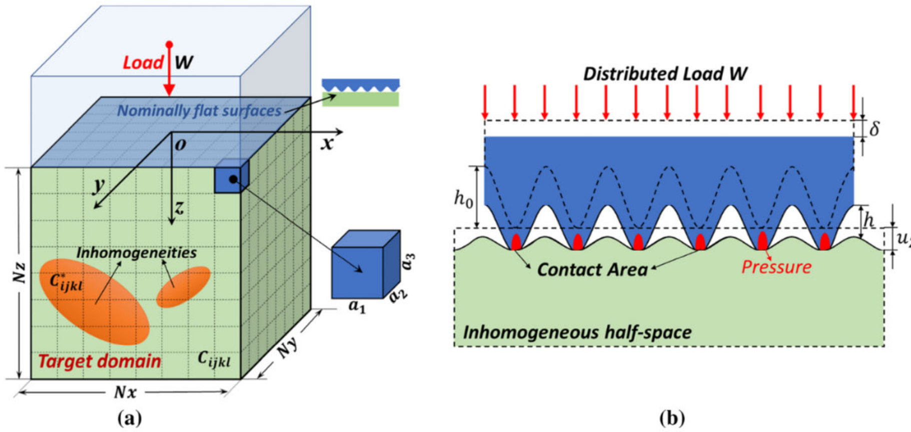

**Fig. 66** A surface-contact model of inhomogeneous material subjected normal load [895]. Reproduced with permission from Ref. [895], © Springer-Verlag GmbH Germany, part of Springer Nature, 2021.

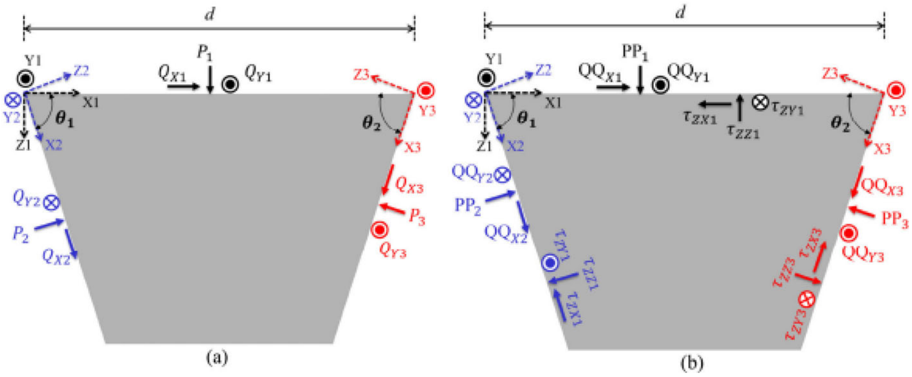

**Fig. 67** An elastic wedge with three loaded surfaces [897]. Reproduced with permission from Ref. [897], © The author(s), 2020.

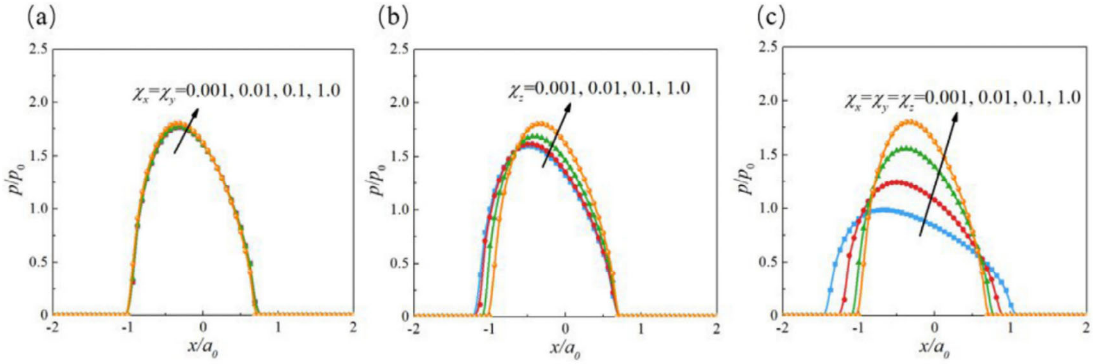

**Fig. 68** Pressure distributions due to different interfacial imperfection indices. (a) Individual lateral imperfection with $\chi_z$ = 1.0, $\chi_x$ = $\chi_y$ = 0.001, 0.01, 0.1, 1.0; (b) individual vertical imperfection with $\chi_x$ = $\chi_y$ = 1.0, $\chi_z$ = 0.001, 0.01, 0.1, 1.0; and (c) combined lateral and vertical imperfections with $\chi_x$ = $\chi_y$ = $\chi_z$ = 0.001, 0.01, 0.1, 1.0 [898]. Reproduced with permission from Ref. [898], © Springer-Verlag GmbH Germany, part of Springer Nature, 2020.

on a two-dimensional finite element model. The soft coating and hard substrate materials of the coated asperity followed the power-law hardening elastic–plastic properties. For both the loading and unloading processes, the effects of geometrical and material properties on contact behaviors were explored, and the fitting formulas were obtained. Combining the topographical-dependent Zhao–Maietta–Chang (ZMC) model with the physical-related Iwan model, Li et al. [905] built the nonlinear constitutive

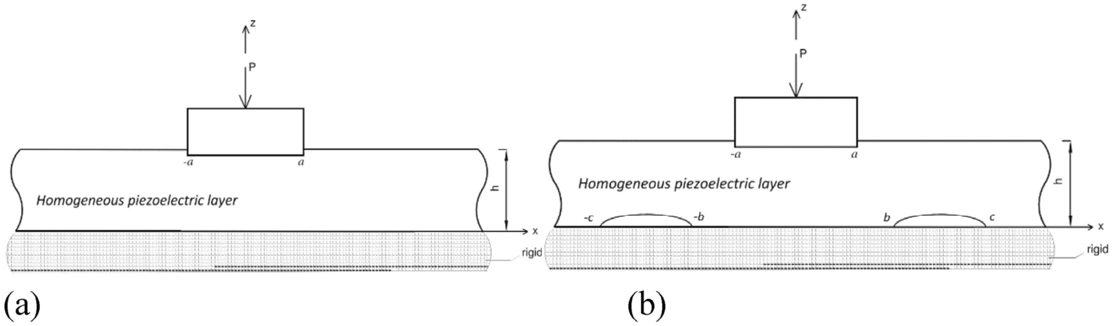

**Fig. 69** Geometry of the (a) continuous contact problem and (b) discontinuous contact problem [898]. Reproduced with permission from Ref. [900], © Springer-Verlag GmbH Austria, part of Springer Nature, 2020.

relationship of a lap interface by using Masing hypothesis, and established a general contact model for a lap joint interface based on non-Gaussian surfaces, which may provide an access to research microslip behavior between two contact surfaces of identical or different materials.

### 6.3.3 *Contact problems involving multipysical fields*

#### 6.3.3.1 Thermal contact

During the working process of the machine, the contact surface of the parts generates a lot of heat due to friction, which causes the temperature to rise. Meanwhile, the change of temperature will also have an impact on the contact performance of the parts, so the research on the contact problem considering the thermal effect is very important in engineering applications. The thermo-elastic or thermo-elastoplastic contact problem of smooth surfaces has been extensively studied, and various contact models have been established. But in engineering practice, the contact surface is rough, and the contact occurs in a limited area. This makes it difficult for the heat flow to pass through the contact surface, and the normal flow of heat is limited by the contact thermal resistance, which affects the heat dissipation of the part. Therefore, the contact thermal resistance between rough surfaces is a key scientific issue. Liu et al. [906] studied the contact thermal resistance between the curved surface and the spherical surface shown in Fig. 70 and established a contact thermal resistance prediction model considering the rough surface of the ball bearing under actual working conditions. The fractal contact model is constructed by considering the elastic, elastic-plastic, and plastic deformations of micro-protrusions, and then an effective contact coefficient was built to modify the fractal contact parameters. The contact thermal resistance prediction is very important not only in the mechanical industry such as bearings but also in the electronics industry. In a microelectronic package, the heat of the microprocessor must be dissipated, thus the heat flow between the microprocessor and the heat sink must have a large conductivity. Vallepuga-Espinosa et al. [907] used the boundary element method to study the contact thermal resistance between 3D microchips and heat sinks in microelectronic packaging. The values of the final thermal and mechanical variables of the boundary of the microelectronic packaging and the thermal contact resistance are obtained by a double iterative schema. However, many prediction studies on contact thermal resistance ignore the influence of thermal expansion, a complete thermal-mechanical coupling simulation costs a large number of calculation time. Dai et al. [908] proposed

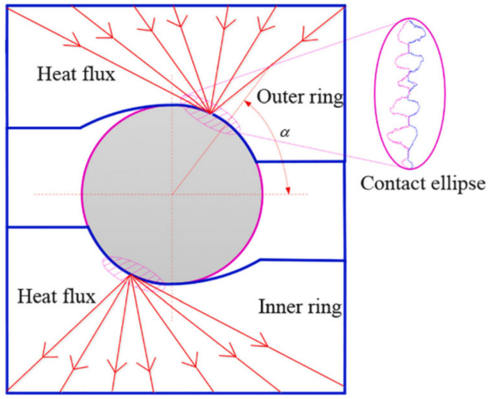

**Fig. 70** The heat transfer mechanism of ball bearings [906]. Reproduced with permission from Ref. [906], © Elsevier Ltd., 2020.

a dual-iterative thermal-mechanical coupling method for predicting the contact thermal resistance, which considered the effect of thermal expansion on the contact thermal resistance and showed the difference up to 91.4% in comparison with that of without thermal expansion, indicating the importance of considering the influence of thermal expansion. In addition, researchers have also paid attention to the change of the contact surface temperature and stress when considering the thermal influence of the parts in contact. Zhang et al. [909] extended the method of solving the three-dimensional frictional contact problem of multilayer materials to the heat transfer and temperature analysis of the tool tip–plate interface in incremental forming as shown in Fig. 71.

#### 6.3.3.2 Electrical contact

The electrical connection is an indispensable and abundant link in power electronic equipment and system, and electrical contact is the core of electrical connection. In electric contact, current flows through two contact bodies, meanwhile, a large amount of heat will be generated on the contact surface due to the hindering effect of the contact resistance. Therefore, electrical contact is a complex electric-thermal-mechanical coupling problem.

The solution of contact resistance and its influence on the temperature and stress of the contact surface are the key to understanding the mechanism of electrical contact. Ren et al. [910] systematically studied the contact resistance of arc-surface contacts by combining analytical solutions and finite element methods. Two typical arc-surface contact models, namely sphere–flat contact and cylinder–flat contact, are established. The current and potential distributions of the two contact forms are compared, as shown in Fig. 72, and the variation of contact resistance with the mechanical load of the two contact models is fitted. However, the thermal effects of the contact and current on the rough surface are not taken into account.

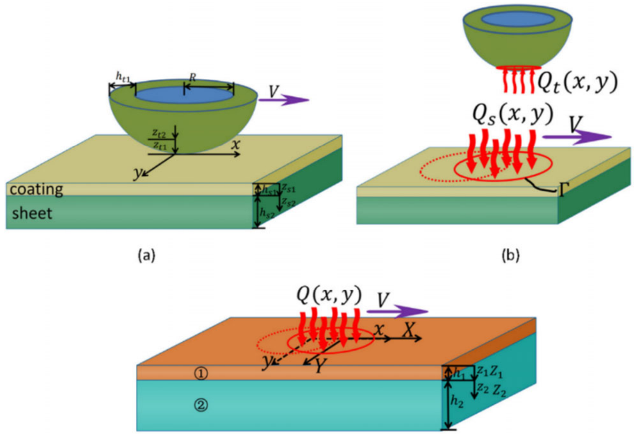

**Fig. 71** Heat transfer at the tool tip–sheet interface. (a) A tool tip moving on a thin sheet with constant velocity $V$ along the $x$-direction; (b) heat generated in the interface between the tool tip and sheet surfaces and its partition, note that the sheet is subjected to a moving heat source; and (c) problem generalized to a coating–substrate system under a moving surface flux, $Q$ [909]. Reproduced with permission from Ref. [909], © Elsevier Ltd., 2019.

The calculation of the contact resistance considering rough surfaces and the thermal effect of current are still the main concerns at present. Based on fractal theory, Hertzian contact theory and fractal network, Liu et al. [911] proposed a prediction model of the electric resistance for rough interface considering the modified hypothesis, 3D interface topography, and elastic-plastic deformation. Using the engineering data of material, the influences of fractal dimension, fractal roughness, loading force, and friction coefficient on the electric resistance were analyzed. Ta et al. [912] proposed a volumetric contact theory for studying electrical contact properties between random rough surfaces as shown in Fig. 73, which did not need to presuppose a certain contact pattern and was not limited to the assumption that the real contact area was much smaller than the nominal contact area. Zhang and Ren [913] proposed an improved peak identification method for numerical reconstruction of rough surface topography, then, a three-dimensional finite element model for analyzing the electric-mechanical coupled contact problem was built. This model can accurately calculate the contact area and facilitate the calculation of contact resistance.

The above studies have tried to consider the influence of rough surfaces on electrical contact, especially the contact resistance, but still did not consider the thermal effect of current. Komvopoulos [914] established a thermal-electrical-mechanical contact model for the electrical contact of the parting rough surface. The elastic, elastic-plastic and full-plastic deformation, heat conduction, temperature rise, and contact resistance were theoretically treated for the contact interface of the parting geometric feature.

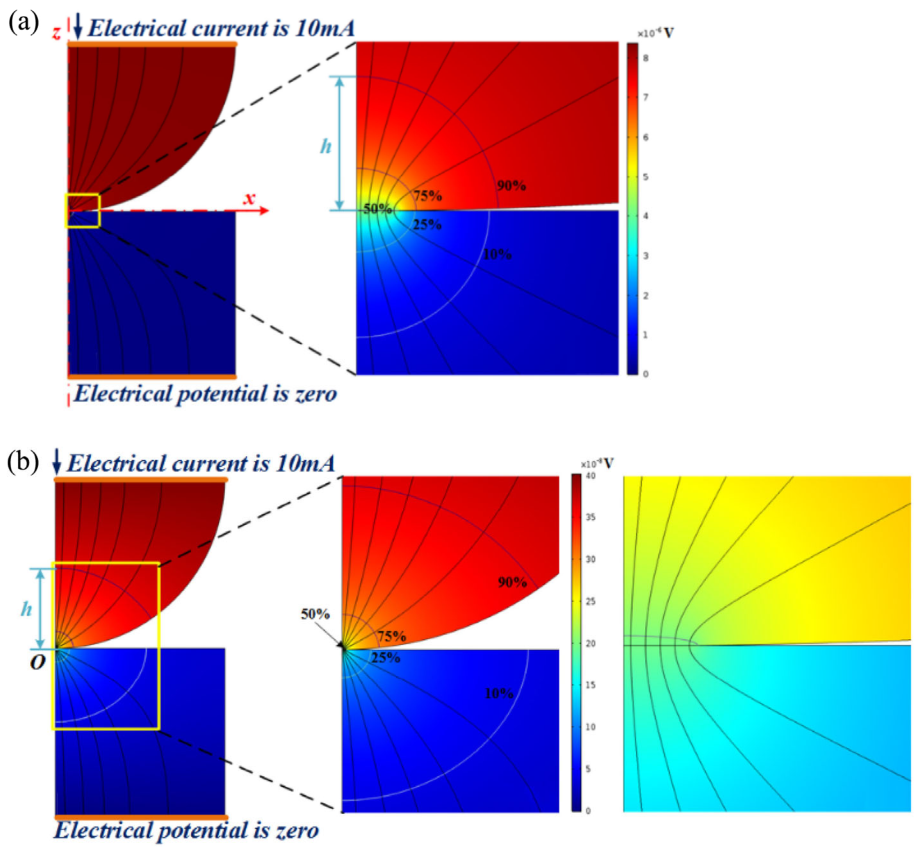

**Fig. 72** Distributions of electrical current lines and isopotential. (a) Sphere–plane contact and (b) cylinder–plane contact [910]. Reproduced with permission from Ref. [910], © IEEE, 2020.

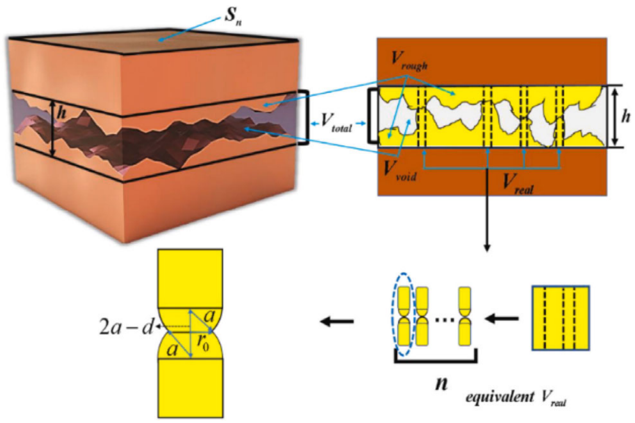

**Fig. 73** Schematic diagram of the contact volumes and the equivalent real contact volume between rough surfaces [912]. Reproduced with permission from Ref. [912], © Elsevier Ltd., 2021.

It provided a basis for the development of multi-scale electro-thermo-dynamic analysis methods for contacting solids. Shen and Ke [915] developed a numerical approach to investigate the electro-thermal-mechanical-wear coupling behavior of electric contacts based on finite element modelling as shown in Fig. 74. An electrical contact conductance law is used with the current conservation model to evaluate the electrical behavior. A transient heat transfer model, including the Joule heating behavior and a thermal contact conductance law, is employed to calculate the temperature field. Both contact conductance laws are related to the contact pressure distribution obtained by the contact stress analysis. The electrical contact calculation model has been applied in various engineering practices, such as electromagnetic emission. The contact resistance between the armature and the guide rail is a very important indicator that affects the contact characteristics and the emission effect. Zhu and Li [916] analyzed the contact characteristics of the reinforced railgun by combining the contact resistance and the sliding friction coefficient and established a theoretical calculation model of the contact resistance of the reinforced railgun.

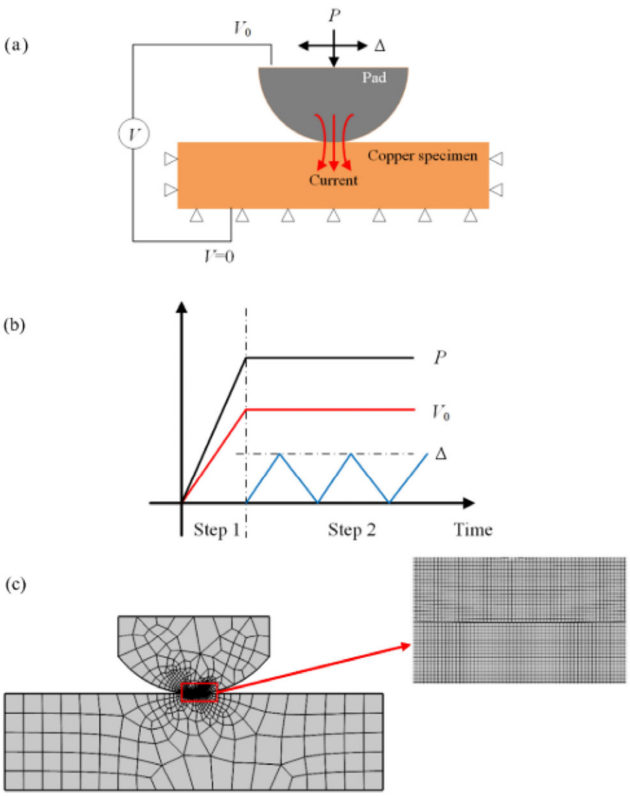

**Fig. 74** (a) Schematic of electrical contact; (b) histories of applied loads; and (c) finite element mesh of electrical contact [915]. Reproduced with permission from Ref. [915], © The auther(s), 2021.

6.3.3.3 Magneto-electro-elastic contact

Magneto-electro-elastic (MEE) material composites of piezoelectric and piezomagnetic phase, have attracted much attention in recent years due to their strong coupled effects between elastic, electric, and magnetic fields. With these effects, MEE materials can be used to realize the conversion between mechanical, electric, and magnetic energy. Çömez [917] solved the 2D frictional sliding contact problem between an MEE half-plane and conducting punches with given total electric and magnetic charges, and investigated the effects of sliding velocity on contact width and stresses. Sui et al. [918] solved the 3D frictional contact between a MEE material and a perfectly conducting rigid spherical punch under electromagnetic fields based on the semi-analytical method (SAM), in which the frequency response functions (FRFs) with respect to unit mechanical, electric, and magnetic loads were derived and the surface density (closed circuit) or total amount (open circuit) of electric and magnetic charges were considered. It is found that the change of electric field presents significant effects on the contact behavior comparing with magnetic field, as shown in Fig. 75. Wu and Li [919] introduced the classical Johnson–Kendall–Roberts (JKR) and Maugis–Dugdale (MD) models into the contact of MEE materials and established the theory of MEE adhesive contact. They study the influence of the electric and magnetic quantities on reversible adhesion. Further,

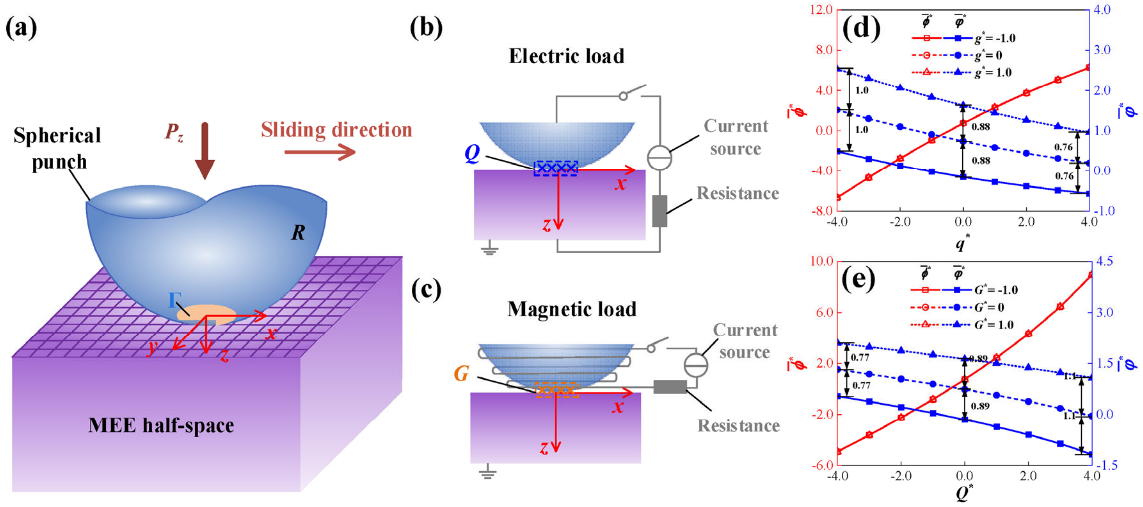

**Fig. 75** Illustration of frictional contact of a perfectly conducting spherical punch with MEE half-space under the electromagnetic fields. (a) 3D sliding contact between spherical punch and the MEE half-space, (b) the circuit diagram of applying electric load $Q$ and (c) magnetic load $G$ in contact region, (d) effects of dimensionless surface electric charge density $q^*$ and magnetic charge density $g^*$, (e) effects of dimensionless total electric charge amount $Q^*$ and total magnetic charge amount $G^*$ [918]. Reproduced with permission from Ref. [918], © Elsevier Ltd., 2022.

Wu and Li [920] concerned partial slip contact problem of an MEE half-space punched by a spherical indenter. The indenter is subjected to the normal indentation force, electric and magnetic loads, and then exerted by a progressively increasing tangential load. The tangential load, which is not sufficient to cause complete sliding, results in the contact region split into the slip region and the stick region. The study found that suitable electric and magnetic loads may eliminate the stress concentration.

As the composition of MEE materials, the piezoelectric phase is easily obtained and more widely used in engineering. Therefore, it is also very important to study the contact problem of piezoelectric materials. Hou and Zhang [921] presented a general theory of the piezo-electric contact mechanics for a transversely isotropic piezo-electric elasticity coated system pressed by a charged spherical. Three-dimensional exact solutions for the stresses, displacements, electric displacements, and electric potential are derived for predicting some transient electrical effects. Huang et al. [922] investigated the frictional problem of the one-dimensional hexagonal piezoelectric quasicrystals layer, which can provide a reference basis for experimental analysis and material development of the piezoelectric devices.

## 6.4 Simulation in wear problem

Accurately modelling the wear processes is a substantial challenge due to the formidable complexity emerging from a variety of physical and chemical mechanisms at disparate time and length scales. Mechanics embedded in this problem are generally complicated by geometrical and material nonlinearities, and analytical solutions are cumbersome or even impossible. The development of computer simulation technology, computational science, and many fields related to surface science allow in accurately simulating the wear process by considering more realistic conditions, which provides insight into physical behavior that is barely visible in experiments.

### *6.4.1 Finite element method*

Wu et al. [923] extended their previous 2D FE model of adhesive wear in asperity in Ref. [924] to metallic materials containing inclusions which are randomly distributed in subsurface. The damage of matrix and the matrix–inclusion interface were considered and the crack initiation and crack propagation during normal and tangential contact loading were simulated. During the tangential loading, crack aggregation and crack branching are predicted and the formation of sheet-like wear particles for matrix-inclusion solids was found. The simulation results demonstrated that the subsurface micro-cracks, particularly those near inclusions, and their subsequent evolution played a major role in the generation of flake-like wear particles. Zhang and Etsion [925] presented a 3D FE model for the evolution of fracture and friction in elastic-plastic adhesive spherical contact with full stick contact condition. The model is based on a realistic criterion of ductile fracture in a three-dimensional contact problem and the failed elements are deleted, allowing the process of material removal in the form of a wear particle. It was found that with increasing tangential loading, fracture first occurs near the trailing edge of the contact area and extends along the contact boundary to the leading edge. Then a second fracture initiates below the contact interface near the leading edge of the contact area and with further sliding, the first and second fractures propagate and link forming a ring-like fractured zone below the contact interface. Eventually, the central un-failed regions vanish and a wear particle, floating on a slip interface, forms between that interface and the flat. Zhang and Etsion [926] then advanced their model with submodel technique to shorten the computing time. It consists of a global model, showing the potential location of fracture and a submodel covering only the region near the potential fracture with refined mesh. Damage initiation and evolution in the submodel are considered and failed elements are deleted from the mesh, allowing to more accurately simulate the wear particle formation. For different values of dimensionless normal load, flake-like wear particles were observed and the volume increased with increasing load. Dhaka and Prakash [927] carried out a two-dimensional finite element analysis for a cylinder-on-plate configuration, to explore the effect of dissimilar materials on fretting wear using frictional dissipation energy density approach. It was discovered that interchanging the material of the mating pairs can significantly alter the normal stress in the fretting direction. This is because similar materials with lower elastic modulus and yield strength are likely to sustain less fretting wear damage because of more plastic deformation and stress redistribution. Lorenz et al. [928] presented a FEM to investigate the effect of prior austenite grain refinement on rolling contact fatigue (RCF), which was determined using continuum damage mechanics (CDM) due to the cycle. They obtained the grain size-dependent CDM critical material parameters ($\sigma_r$, $m$) from open literature torsion S-N data. RCF life displayed an inclination to improve as grain size was refined, and a predictive fatigue life equation was constructed as a function of the relations between grain size, contact pressure, and the Weibull shape ($\beta$) and scale parameters ($\eta$).

To understand the complexity of contact loads or formation of wear particles in abrasion, Lindroos et al. [929] established 3D finite element based micromechanical model to study single contact abrasion on a high manganese austenitic steel, in which crystal plasticity framework was utilized and the microstructure of the material was included. As shown in Fig. 76. It presents a potential micromechanically inspired modelling approach to investigate the local effects of microstructure, link deformation mechanisms, and hardening to the material's behavior under abrasive contact.

### 6.4.2 *Atomic simulations*

#### 6.4.2.1 Fundamentals of solid friction and wear

Friction is a multiscale phenomenon, which remains largely unknown at both macroscale and microscale. At microscale, an in-depth understanding of friction induced materials structural evolution, and friction tuning mechanism by external field will be beneficial to materials selection and design for an improved performance. Also there is a trend for multiscale modelling to combine macroscale and microscale laws without gap, which, however, still remains a big challenge.

Zhu et al. [930] investigated the nano-friction process of Ni-based polycrystalline superalloy coatings using MD simulations. The results showed that the friction force is related to the dislocation slip direction, and the effect of grain boundaries and grain size on tribological behaviors were revealed: the grain boundary structure showed a strengthening effect, which hindered propagation of stacking fault; as the grain size decreased, the material softened, resulting in a decrease in friction coefficient and increase of wear depth. Furthermore, in order to better capture

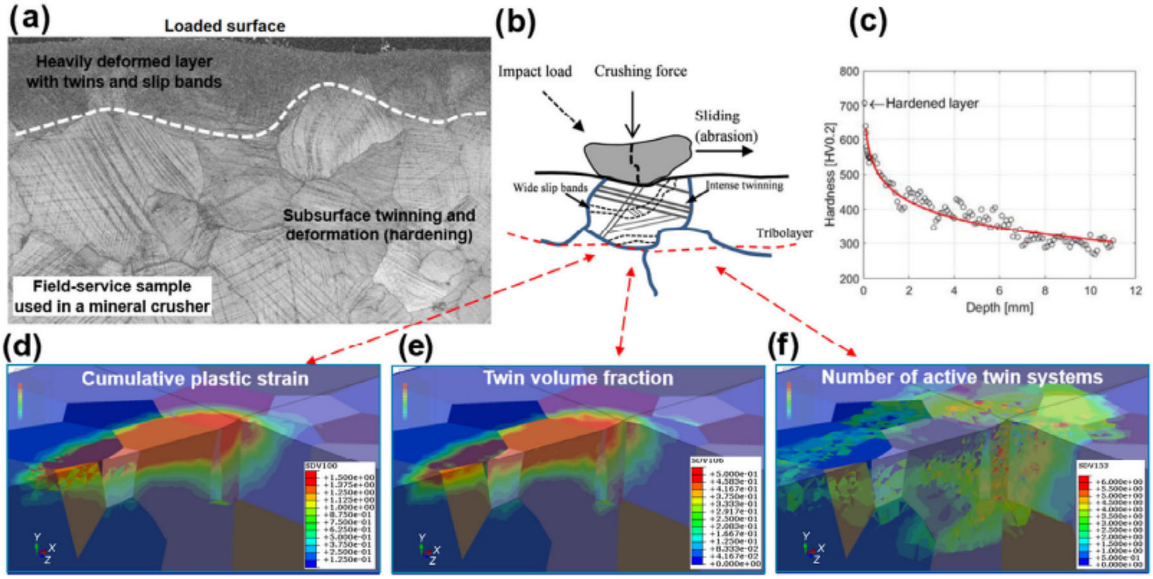

**Fig. 76** Simulation results from micromechanical modelling of polycrystalline high manganese austenitic steel subjected to abrasive contact. (a) Cross-section of field-service sample; (b) schematic of surface deformation; (c) cross-section hardness gradient; (d) cumulative plastic strain; (e) twin volume fraction; and (f) number of currently active twin systems [929]. Reproduced with permission from Ref. [929], © The author(s), 2020.

the materials microstructural evolution during friction, Eder et al. [931] innovatively proposed the "differential computed orientation tomography" (dCOT) method, based on large-scale MD simulations (Fig. 77(a)). By using this method, near-surface deformation, e.g. twinning, lattice rotation, and grain boundary dominated processes, can be clearly observed, which helps design and optimize materials for improved tribological performance.

To investigate the dependence of macroscopic friction behavior on the contact properties of nanoroughness, Hu et al. [932] first used MD simulations to study non-adherent shear between individual asperity contact and then the friction behavior was implemented into the Greenwood–Williamson model. Then the effects of asperity size, velocity, and crystal orientation derived at the nanoscale were adopted to determine the macroscopic friction coefficient through the statistical model and system parameters. This work presented a step forward to understanding of macroscopic tribological phenomena from the nanoscale behaviors.

Traditionally, wear has been described by empirical laws, such as Archard's Law. However, empirical laws often do not have clear physical meaning and are sometimes difficult to apply in nanoscale contacts. Wang et al. [933] proposed a non-empirical and universal wear law applicable to the nanoscale contact

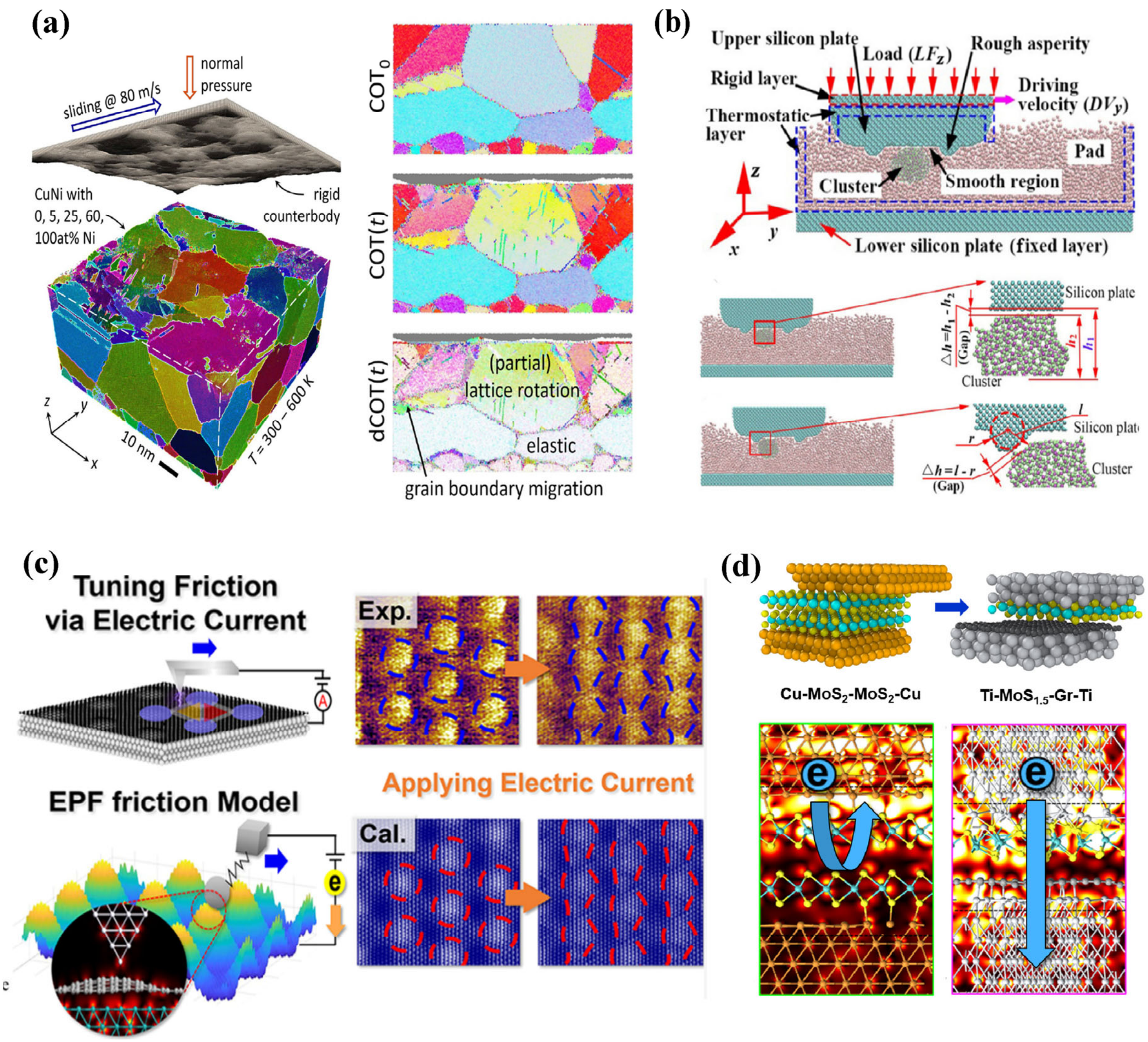

**Fig. 77** Mechanism of friction and wear at atomistic scale [931, 934, 935, 937]. Reproduced with permission from Ref. [931], © The author(s), 2021; Reproduced with permission from Ref. [934], © The author(s), 2022; Reproduced with permission from Ref. [935], © American Chemical Society, 2022; Reproduced with permission from Ref. [937], © The author(s), 2020.

situation, successfully explained why the nanoscale wear behavior does not conform to the description of Archard's law, and verified it by atomic simulations. This work provides a theoretically robust method for calculating nanoscale contact wear and will contribute to develop design principles for wear reduction.

Chen and Li [934] investigated the three-body nano-abrasive wear process during a polishing process by molecular dynamics simulations (Fig. 77(b)). They found that the suspension sliding of abrasive in smooth regions and rolling impact at asperity regions of the silicon surface, leading to damage-free monoatomic layer removal. These findings provide a new microscopic understanding of the three-body abrasive wear induced material removal mechanism in ultra-precision manufacturing, such as CMP.

The friction tuning by external electric field or electric current has been experimentally observed for decades, but the microscopic mechanism still remains elusive. Recently, Song et al. [935] revealed the tuning effect of electronic properties fluctuation (EPF), i.e., the electric field-induced electron redistribution and current-induced electron transfer fluctuations, on friction (Fig. 77(c)), based on which a friction model considering the effect of electronic properties fluctuation on potential energy corrugation during friction was proposed. Vazirisereshk et al. [936] investigated nanoscale time-dependent electrical contact resistance (ECR) by combining conducting atomic force microscopy and MD simulations, and unraveled the important role of contact aging in interfacial electronic conduction. The contact aging will lead to the increase of actual contact area and the decrease of ECR, which is a thermally activated process and can be described by Arrhenius law. Song et al. [937] put forward the atomic-scale contact quality (ACQ) model unraveling the impact of interfacial atomic stacking on the contact conductance of 2D interfaces. Furthermore, a design strategy to obtain low friction and high conductivity in sliding electrical contacts via 2D heterointerface were proposed (Fig. 77(d)).

Wear significantly affects the service life of the devices, however, wear is always described by empirical laws such as the Archard's law. The moreover empirical laws do not always hold for nanoscale contact. Thus, Wang et al. [933] proposed a non-empirical and robustly applicable wear law using molecular dynamics simulation. The proposed wear law successfully reveals the reason why the nanoscale wear results in certain experiments behaves obeying the description by Archard's law while does not in others.

SiC, an important ceramic material in industries, is widely used as a protective coating on sliding or rolling contact surface to reduce friction and wear. Thus, Wang et al. [938] studied the atomic-scale friction/wear mechanisms of water-lubricated SiC in the rolling contact state by applying molecular dynamics simulation as shown in Fig. 78. It can be found from the study that an interesting selective wear of silicon atoms from the SiC surface during the rolling friction state, instead of almost identical wear of silicon and carbon atoms in sliding friction case.

Friction contacts cause the formulation of wear particles whose behavior and properties at the nanoscale can significantly affected the macroscopic tribology performance. To study how wear particles evolve with time and influence the surface topography, Brink et al. [939] presented molecular dynamics computer simulations with a multiparticle, three-dimensional setup with fully plastically deformable, rough surfaces. From molecular dynamics computer simulations, it can be found that the wear particles

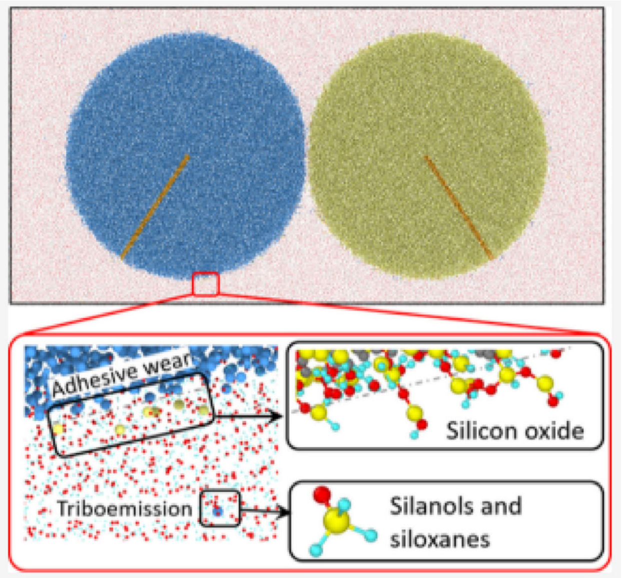

**Fig. 78** Snapshots from the rolling friction simulation of SiC in a water environment [938]. Reproduced with permission from Ref. [938], © American Chemical Society, 2021.

evolve from spherical particles to cylindrical rollers and then to shear-band-like state, as shown in Fig. 79.

6.4.2.2 Friction and wear of polymer matrix composites

Self-lubricating polymer matrix composite coatings with customizable tribological and mechanical properties have been widely used in mechanical parts to reduce friction and wear, promoting lifetime and performance. Due to the high molecular weight of the molecular chains of polymer-based composites, there is a cross-scale effect in the simulation research on the molecular scale. In molecular dynamics simulations, the friction and wear behavior of polymer matrix composites is often studied by establishing all-atom friction systems or coarse-grained friction systems. In the past two years, there have been many advances in the researches of polymer matrix composites using molecular dynamics.

In order to explore the lubrication mechanism of polymer molecular chains in the friction process, all-atom simulations are used to study the friction and wear behavior of polymer molecules. Song and Zhao [940] established a tribological model of PTFE sliding on a Cu layer under dry friction and water lubrication conditions through molecular dynamics simulations. After water lubrication, the steady-state average friction coefficient of PTFE decreased from 0.189 to 0.064. Compared to dry friction, water lubrication greatly reduced the wear of PTFE. The radius distribution function (RDF) values indicated that water molecules have strong interactions with Cu atoms and PTFE molecules during wear. Water molecules gradually accumulated on the interface between the top Cu layer and PTFE as lubricant, finally forming a lubricating layer with enhanced wear resistance. The wear mechanism of PTFE under water lubrication also changed from mixed lubrication to elastohydrodynamic lubrication. Savio et al. [941] elucidated friction in poly-ether-ether-ketone (PEEK) sliding contact by the classical molecular dynamics simulations of dry and water-lubricated amorphous PEEK–PEEK interfaces at the nanoscale (Fig. 80(a)). A linear pressure–shear stress dependence was revealed, and this dependence was summarized as a nanoscale friction law, which bridged between molecular

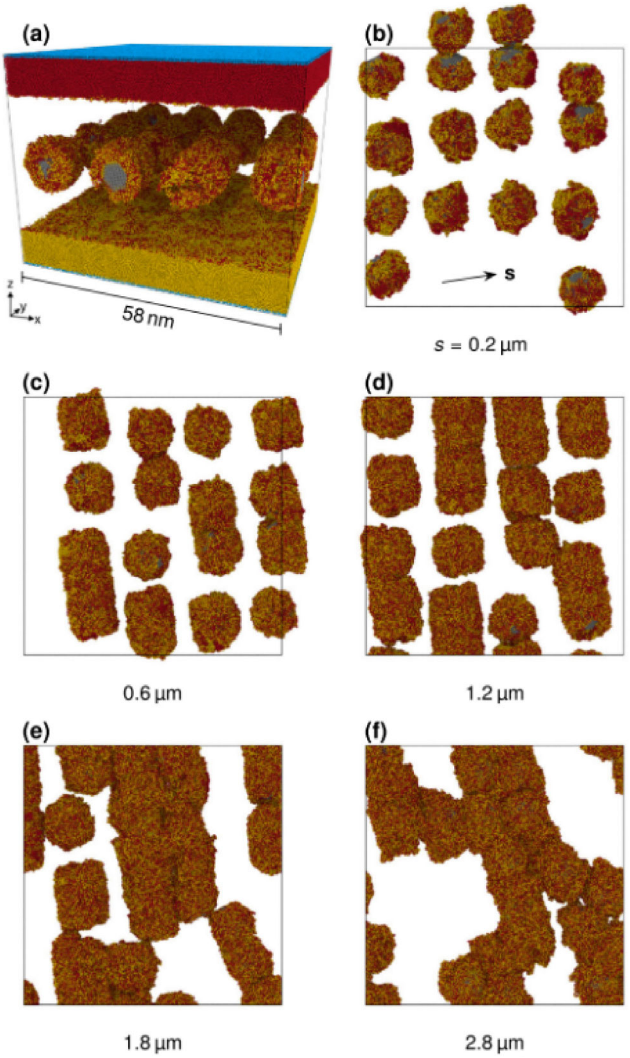

**Fig. 79** Evolution of wear particles. (a) Particles were introduced between two surfaces and subjected to sliding motion. (b)–(f) Wear particles evolve from spherical particles in (b) to cylindrical rollers (c) and (d), to a porous, shear-band-like state in (e) and (f) [939]. Reproduced with permission from Ref. [939], © American Physcial Society, 2022.

dynamics and elastic-plastic contact mechanics calculations. The nanoscale friction law together with the real contact area was adopted to estimate the macroscopic friction coefficient which was further verified by experiments. To elucidate the experimentally observed film transfer and friction mechanisms between polymer and steel counterface, van Goeldel et al. [942] performed sliding MD simulations on PTFE-lubricated iron oxide surfaces using a density functional-based non-reactive force field, explaining the interaction of PTFE chains with iron oxide. Strong adhesion promotes the formation of transfer films, while shear-induced chain alignment within PTFE reduces friction. Xu et al. [943] studied the tribochemical reaction behavior of the iron-oxygen-water-PTFE system by training and extending the ReaxFF reactive force field (C/H/O/F/Fe). The research revealed the tribochemical behavior of PTFE molecules which were chemically bonded and anchored to the Fe or $Fe_2O_3$ counterface to form a transfer film and friction-induced molecular orientation mechanism for its solid lubrication behavior (Fig. 80(b)). It also showed that the changing behavior of molecular mobility and chain stiffness with temperature dominates the non-monotonic variation of friction. Besides, there are many research progresses on the reinforcement

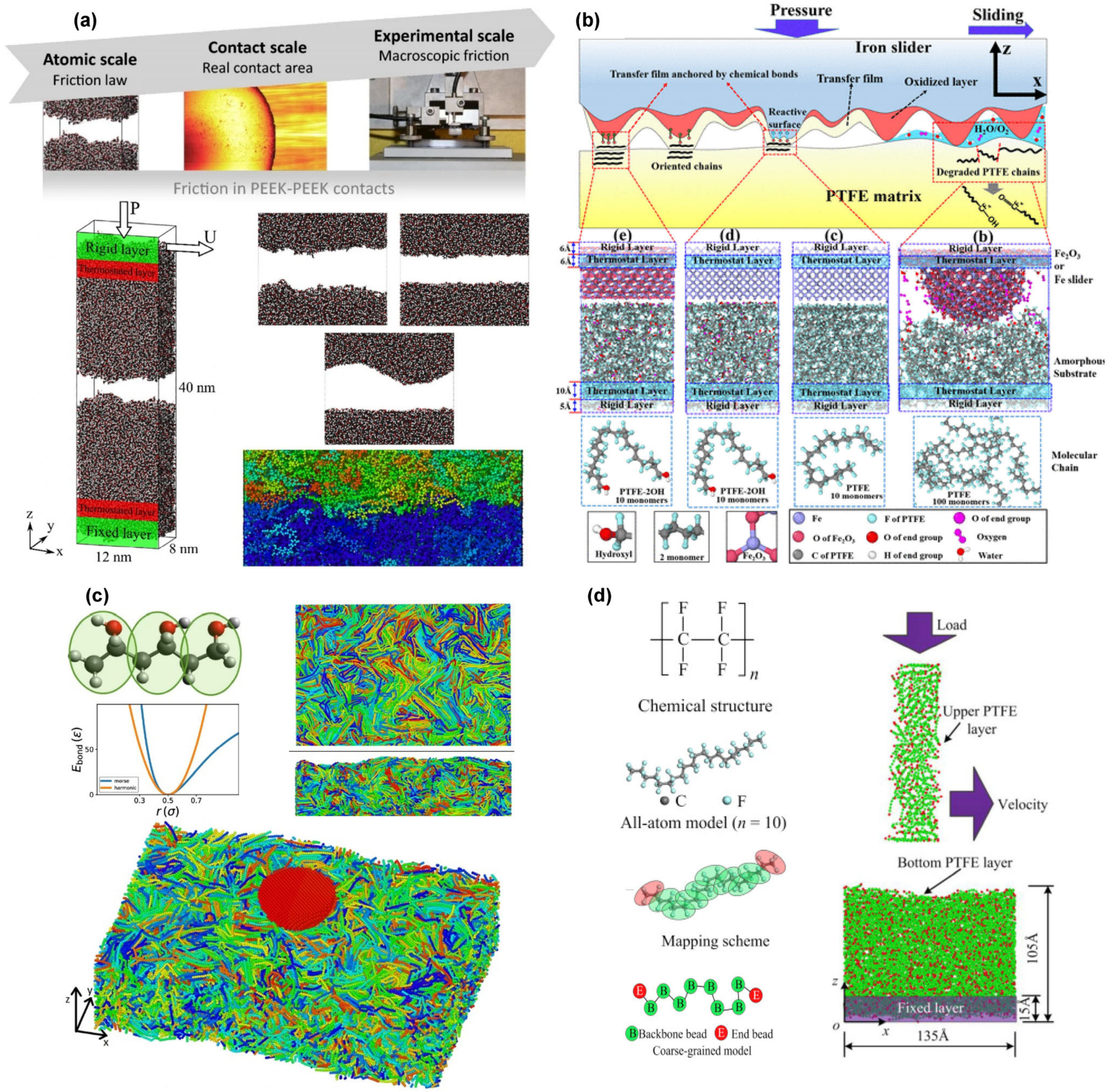

**Fig. 80** Atomistic or coarse-grained molecular dynamics simulations of tribology of polymeric materials [941, 943, 948, 949]. Reproduced with permission from Ref. [941], © The Author(s), under exclusive licence to Springer Science+Business Media, LLC, part of Springer Nature, 2021; Reproduced with permission from Ref. [943], © American Chemical Society, 2022; Reproduced with permission from Ref. [948], © The author(s), 2021; Reproduced with permission from Ref. [949], © Elsevier Ltd., 2019.

phase of composite materials. Xu et al. [944] further investigated the tribological behavior of PTFE composites filled with graphene (Gr) sheets and carbon nanotubes (CNTs) at the nanoscale. They found that the CNT- and Gr-filled composites exhibited better wear resistance than pure PTFE, with 76.2% and 85.7% lower wear amounts, respectively. Due to the stronger interaction at the Gr/PTFE interface, the Gr fillers have a better reinforcement effect than CNTs. Talapatra and Datta [945] investigated the mechanical and tribological properties of pure thermoplastic polyurethane and functionalized graphene-reinforced thermoplastic polyurethane polymer nanocomposites by molecular dynamics simulation. It was found that 3 wt% addition of functionalized monolayer graphene can effectively enhance Young’s modulus and reduce friction coefficient and abrasion rate. Fan et al. [946] studied the mechanical properties and failure mechanism of graphene/epoxy composite under uniaxial tension and shear loading. It was found that the Young's modulus and tensile strength increase, yet shear modulus and shear strength are reduced due to the interfacial slipping between epoxy and graphene. Liu et al. [947] studied the mechanism of improving the mechanical and tribological properties of nitrile rubber (NBR) by adding nano-$SiO_2$ from the molecular scale. They found that hydrogen bonding and interfacial interactions between nano-$SiO_2$ and NBR molecular chains reduced the free volume fraction of NBR nanocomposites during the shear process and thus improved the tribological properties of NBR.

In order to further study the effects of molecular features on a larger scale, for example, to describe chain entanglement and crystalline structures, coarse-graining molecular dynamics are commonly used by neglecting some atomic-scale details. Vacher and de Wijn [948] investigated the underlying mechanism of running-in process of semi-crystalline polymers using a coarse-grained method (Fig. 80(c)). Semi-crystalline structure of polyvinyl alcohol was constructed, which was sliding against a semispherical tip mimicking the tip of atomic force microscopy. They found the alignment of molecules along the sliding direction under the shearing action of the tip. Both plastic and elastic deformation were identified using this scheme. Pan et al. [949] developed a method to simulate the tribology of PTFE by estimating molecular interaction parameters (Fig. 80(d)). They used an iterative Boltzmann inversion method and a multicenter Gaussian-based potential to fit the molecular interaction parameters between coarse-grained PTFE beads, and validated the matching parameters by comparing the all-atom PTFE model with its corresponding coarse-grained model. It was found that with the increase of normal load, the friction coefficient decreases, and the wear depth increases, which was verified by their experiments.

#### 6.4.2.3 Friction and wear of adhesive contact

The work of Aghababaei et al. [950] in 2016 for adhesive wear mechanisms with coarse-grained atomistic simulations triggered the extensive following studies. For the interface adhesion, Brink and Molinari [951] further investigated the adhesive wear particle formation for sliding interfaces in dry contact in the presence of weak interfaces. They found that colliding surface asperities can either deform plastically, form wear particles, or slip along the contact junction surface without significant damage, depending on the material and interface properties and the local slopes of the surfaces. A mechanism map as a function of material properties and local geometry was proposed with confirmation by quasi-two-dimensional and three-dimensional molecular dynamics and also finite-element simulations. An analytical expression of a critical length scale incorporating interface properties and roughness parameters was provided, which indicated debris formation for the contact junctions of sizes above the critical length scale. Milanese et al. [952] studied the role of interfacial adhesion on minimum wear particle size and roughness evolution. They found that at short timescales, it is the surface morphology and not the interfacial adhesion strength, that dictated the minimum size of wear particles, while at longer timescales, adhesion altered the particle motion and thus the wear rate and the surface morphology.

Aghababaei and Zhao [953] conducted systematic long-timescale asperity-level wear simulations of material detachment during adhesive wear numerical for assessment of Archard's wear relation, i.e. wear volume varied linearly with the normal load and sliding distance. They found that a linear wear relation could be recovered only when the material removal progresses dominated by plastic deformation at the asperity tip, confirming the long-standing theoretical hypothesis made by Archard. However, this linearity breaks down when cleavage cracking dominates the material removal.

#### 6.4.3 Discrete element method

Dimaki et al. [954] proposed a 2D discrete-element based model of adhesive wear for elastic-plastic materials with non-ideal plasticity and with an account of both cohesive and adhesive interactions inside the material. A detailed study of factors controlling the modes of adhesive wear was performed and three main modes of wear, i.e., slipping, plastic grinding, cleavage and breakaway were found, depending on the material and loading parameters. They found that the occurrence of a particular mode was determined by the combination of two dimensionless material parameters: The ratio of the adhesive stress to the pure shear strength of the material, and sensitivity parameter of material shear strength to local pressure. The case study map of asperity wear modes in the space of these parameters was also constructed.

Pointing out the high computational cost and a narrow window of time and length scales in atomic simulations, Pham-Ba and Molinari [955] recently reproduced the key mechanisms observed with atomic simulations by DEM having particle diameters and system sizes an order of magnitude higher, validating the coarse-graining procedure.

#### 6.4.4 Phase-field approach

Collet et al. [956] proposed a brittle formulation of the variational phase-field approach to fracture for study of the physical processes of adhesive wear at the scale of the surface roughness. They found that the failure mechanisms of an adhesive junction could be linked to its geometry. By imposing specific couplings between the damage and the elastic energy, they found that a large debris formation was mostly triggered by tensile stresses while shear stresses led to small or no particle formation. With the study for groups of junctions, a classification in terms of macroscopic wear rate was proposed. Although based on a continuum approach, the phase-field calculations were able to effectively capture the failure of adhesive junctions.

#### 6.4.5 Wear simulation of rough surface

Due to the long computing time in other methods, boundary element method (BEM) and semi-analytic method are the proper ways to model wear of rough surface. Based on their previous elastic simulation with BEM in Ref. [957], Frérot et al. [958] investigated the consequences of plastic deformation on the macro-scale wear response using novel elastoplastic contact simulations. The crack nucleation process at a rough contact interface was analyzed in a comparative study with a classical J2 plasticity approach and a saturation plasticity model. They found that the plastic residual deformations in the J2 model heightened the surface tensile stresses, leading to a higher crack nucleation likelihood for contacts. The elastic interactions between contacts can increase the likelihood of individual contacts nucleating cracks, irrespective of the contact constitutive model.

Cao et al. [261] proposed a novel fracture-induced adhesive wear criterion with semi-analytical method to predict local wear of material. The effects of work of adhesion, surface energy degradation and also the stochastic distributions of these physical properties of materials on adhesive wear were considered into the criterion. Coupled with the deterministic mixed lubrication theory, the wear criterion was applied to predict the wear process of mixed lubricated point contact, showing that morphology evolution of surfaces and wear particles formation during wear process can be simulated by using the proposed wear criterion and simulation procedure. The comparison of simulation results with experiment measurements showed reasonable agreement between them.

Tan and Ma [959] presented an analytical wear model for the slide guide under the boundary lubrication condition based on the relationship of friction fatigue failure of asperities, which is applicable for the wear calculation of the sliding pairs with the dominant wear behavior of friction fatigue under dry friction or boundary lubrication condition. Xiang et al. [960] presents a dynamic Archard wear model for micro-grooved water-lubricated bearings considering the transient mixed elastohydrodynamic lubrication (mixed-EHL) condition, the dynamic wear behavior of textured water lubricated bearing was investigated, and rapid and slight wear stage could be identified.

Liu et al. [961] developed a numerical model incorporating the lubrication state, tooth surface roughness, residual stress, and mechanical properties of the material to determine the contact fatigue behavior of a megawatt level wind turbine carburized gear, in which the variations of the hardness and residual stress along the depth were characterized by the Vickers hardness measurement and X-ray diffraction test, respectively; modified Dang Van diagrams under different working conditions were determined to estimate the contact fatigue failure risk.

*6.4.6 Wear prediction with machine learning algorithms*

Utilizing experimental data to predict wear behaviors of materials has been an increasing need based on machine learning algorithms. Altay et al. and Ulas et al. [962, 963] presented a machine learning methods including the interaction linear regression (LR), the kernel function cubic in the support vector machine (SVM) method, and the rational quadratic in the Gaussian process regression (GPR) method or artificial neural network and basic, kernel-based, and weighted extreme learning machine to predict the wear amounts of the surface coatings FeCrC, FeW, and FeB ferro-alloys, It was observed that a more efficient model could be created by using the GPR algorithm when the amount of wear was predicted.

## 6.5 Simulation in rough surface

Zhang et al. [964] proposed a multiscale model to quantitatively predict the engine cylinder bore surface generated from the honing process at the macroscale and microscale simultaneously, which consisted of three submodels including an abrasive distribution model of honing stone, a kinematic model of the honing process, and initial topography model for the cylinder bore surface evaluation. The model comprehensively evaluated the effects of abrasives distribution, abrasive wear process, the initial surface topography of the cylinder bore, and honing trajectory contacting with the cylinder bore on the dimensional accuracies of the honed cylinder bore at multiscale levels. The research provided an effective approach to predict the honed quality of the cylinder bore in terms of the dimensional accuracy at the macroscale level and the surface texture/topography of cylinder bore at the microscale level simultaneously.

Asperity plays an important role for rough surface contact and lubrication performance, it is vital to accurately define asperity. In order to accurately characterize the whole 3D rough surface, Wen et al. [965] presented a reconstruction and parameter calculation method by fitting the asperity on the 3D rough surface with ellipsoid. They applied watershed algorithm in image segmentation field to segment the region corresponding to asperity, instead of traditional five-point or eight-point methods, etc. As stated, it can retain the true texture structure and height characteristics of the real surface, and the constructed asperities are closer to the real contacted asperities, while the computation of asperity parameters has better stability under different sampling intervals.

Due to the scale-dependence of rough surface, roughness parameters hardly accurately predict the surface properties. Sanner et al. [966] developed a new method to characterize the rough surface: the Scale-Dependent Roughness Parameters (SDRP) analysis, which yields slope, curvature, and high-order derivatives of surface topography at many scales, even for a single topography measurement. The basic idea behind the scale-dependence roughness parameters is shown in Fig. 81.

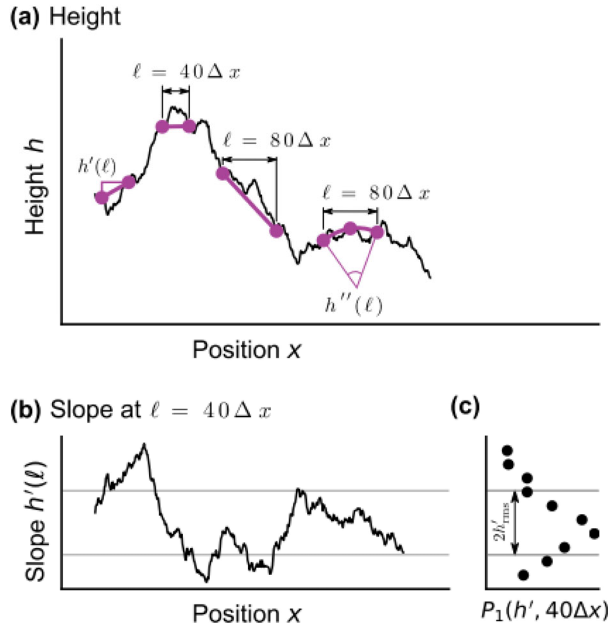

**Fig. 81** Illustration of the basic idea behind the scale-dependence roughness parameters [966]. Reproduced with permission from Ref. [966], © The author(s), 2022.

### 6.6 Simulation in micro-friction and superlubricity

2D materials with layered structures are excellent candidates for the field of lubrication due to their unique physical and chemical properties, such as interlayer van der Waals interaction, chemical inertness, low surface energy, etc. The excellent properties were explored firstly at the microscale, which shows potential usage in tribological applications. In the past two years, studies focused on micro-friction and superlubricity behavior of 2D materials, as will be described in detail below.

Zhao and Duan [967] investigated the friction process of DLC over nanoparticles supported by amorphous silica (a-$SiO_2$) plates and the effect of wrapping graphene sheets on the friction properties of carbon nanodiamond (CND) by non-equilibrium molecular dynamics simulations (Fig. 82(a)). They found that wrapping graphene sheets around CND to form carbon nanoscrolls (CNS) reduced the COF by 72% relative to CND and achieved superlubricity (COF $\leqslant$ 0.01), which was consistent with experimental observations. It was found that due to the existence of two-dimensional graphene sheets, the contact area

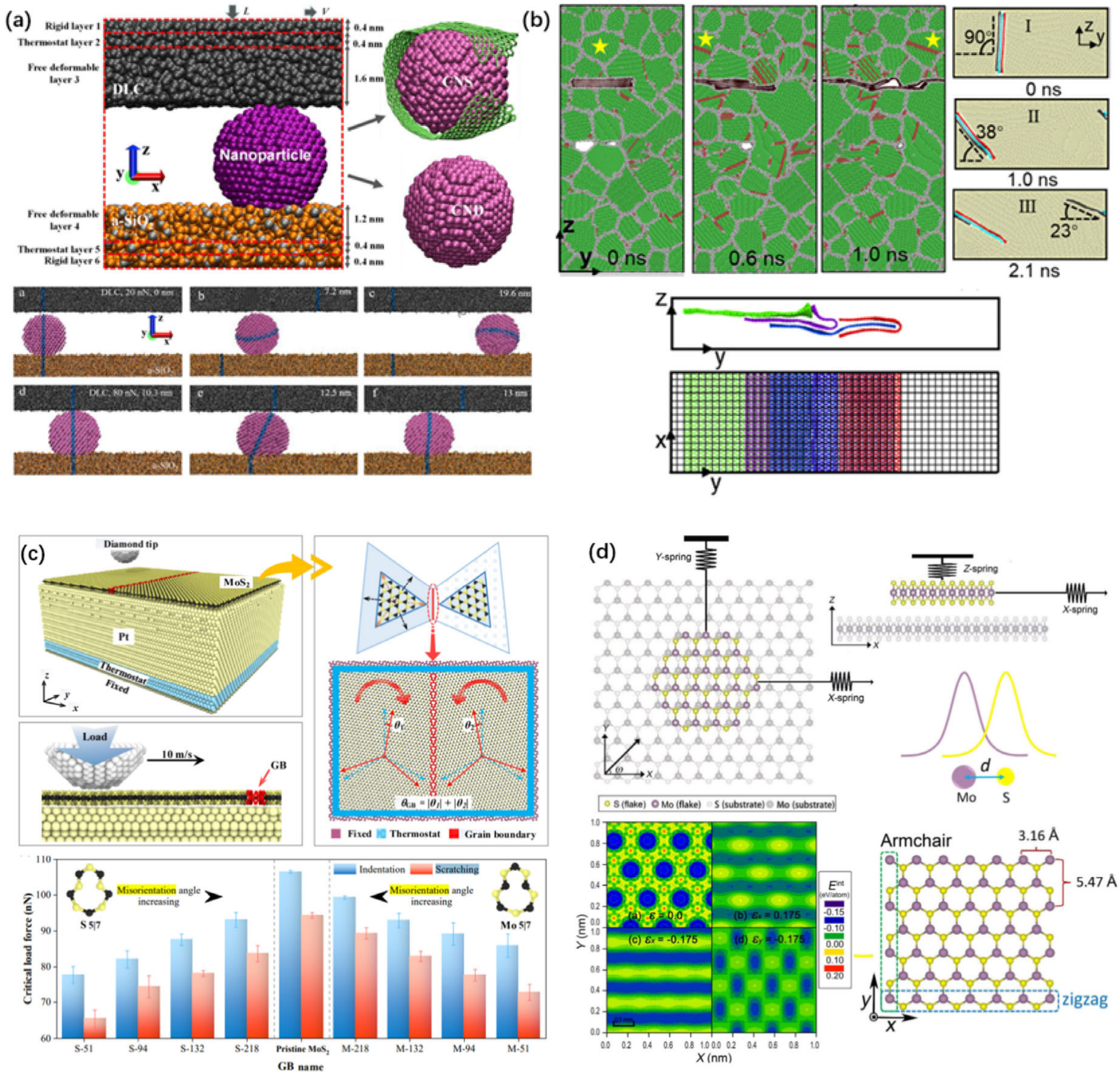

Fig. 82 Effects of grain boundary or in-plain strain on wear or superlubricity of 2D materials [967, 968, 971, 973]. Reproduced with permission from Ref. [967], © Elsevier Ltd., 2022; Reproduced with permission from Ref. [968], © Elsevier B.V., 2020; Reproduced with permission from Ref. [971], © The author(s), 2021; Reproduced with permission from Ref. [973], © The author(s), 2022.

of DLC–CNS is smaller than that of DLC–CND, which inhibited the movement of CNS, thereby reducing the frictional dissipation of the system and effectively improving the lubricity of CND. The excellent lubricity obtained by using 2D graphene sheets as the additive phase of composites has also aroused the interest of researchers. Zhang et al. [968] investigated the self-lubricating behavior of copper-based nanocomposites embedded with graphene nanoflakes (GNFs) by large-scale molecular dynamics simulations (Fig. 82(b)). The simulation results showed that in the polycrystalline copper matrix, the friction reduction is achieved through reorientation of GNF, and the friction coefficient is closely related to the coverage of GNF on the sliding interface. When GNF is distributed over the entire sliding interface, the minimum friction coefficient can be obtained. Compared with the conventional view of extrusion mechanism, they found that the van der Waals gap formed in GNF and between GNF and Cu matrix is the main factor to reduce sliding resistance, which enriched the understanding of the lubrication mechanism of metal matrix composites (MMCs). In addition, many studies have been carried out on the friction and wear behavior of graphene under the influence of hydrogen. For example, aiming at the wear behavior of graphene layer in the hydrogen environment, the presence of hydrogen adsorption may promote the fracture of the stretched carbon chain at the edge of the wear cavity, or change the propagation direction of the wear cavity, resulting in the early failure of the graphene layer [969]. Furthermore, it was found that hydrogenated graphene exhibited superior tribological behavior under low or intermediate loads and even for rough substrates with small periodic asperities, which provided a guideline to achieve superlubricity by chemical modifications [970].

Besides, the effect of defects on the friction and wear behavior of 2D materials has also been studied. Wei et al. [971] investigated the effect of grain boundary (GB) defects on the tribological behavior of $MoS_2$ by MD simulations (Fig. 82(c)). It is found that the wear resistance and load-bearing capacity of $MoS_2$ with GB defects are related to misorientation angles. Compared with the pristine single-layer $MoS_2$ (SL-$MoS_2$), the critical fracture load of $MoS_2$ with GB defects is significantly reduced, and the critical load is smaller with the smaller misorientation angle. When friction occurs at the grain boundary, the dislocated GB structure will undergo massive bond breakage. The Mo–S bond length near the low-angle GB is significantly larger than that of ordinary $MoS_2$, namely "long bond", the accumulation of which leads to wear failure at the GB. These results have important implications for understanding and improving the wear resistance of SL-$MoS_2$ with GB defects. Gao et al. [972] investigated the effect of corrugated GB on the tribological properties of extended planar graphite contacts containing polycrystalline surfaces through molecular dynamics simulations. It is found that dynamic friction is mainly affected by shear-induced buckling and debuckling of corrugated GB dislocations, resulting in non-monotonic behavior of friction under normal loads and temperatures. The process involves two competing effects of an increase in the dislocation buckling probability and a reduction in the energy dissipated per buckling event. The two effects are well captured by the phenomenological two-state model, enabling the characterization of the tribological properties of any large polycrystalline layered interface while avoiding the need for harsh atomic simulations. It also opens a way to achieve large-scale superlubricity by reducing the excess friction associated with each individual GB.

On the basis of two-dimensional materials, researchers have also carried out many explorations on the theoretical research and realization of superlubricity. Wu et al. [973] analyzed the friction between $MoS_2$ nanoflakes and $MoS_2$ substrates using an atomistic force fields-based modified Tomlinson model (Fig. 84(d)). Researches have shown that large deformation in the substrate can lead to a sharp drop in the friction force between the nanoflakes and the substrate, resulting in superlubricity. This friction reduction is strongly anisotropic, the coefficient of friction is the lowest when high tensile strain is applied in the $x$ direction or high compressive strain is applied in the $y$ direction. From the strain induced potential energy surface (PES), it can be found that a sliding pathway with the lowest energy corrugation will be formed by applying a compressive strain in

*x* direction, thereby reducing friction. Similar studies have also been conducted on graphene and hydrogenated graphene systems. The strain can significantly reduce the friction force, and the effect of biaxial strain on friction reduction is more obvious than that of uniaxial strain [974]. Two-dimensional material heterojunctions are also potential structures for achieving superlubricity. Ru et al. [975] studied two-dimensional tellurene isomers (α-Te/β-Te) as well as two-dimensional tellurene and graphene (G/α-Te, G/β-Te). It was found that the G/α-Te system can obtain superlubricity in a wide range of interlayer angles, and is almost independent on the sliding direction, velocity, and normal load. Furthermore, superlubricity has also been found in the friction process of two-dimensional colloidal particles with strong repulsion or weak attraction [976], which is valuable in understanding the mechanism of sliding friction in mesoscopic particle systems and for controlling their self-organization.

### 6.7 Tribochemistry

Tribochemistry is the study of chemical reactions at tribo-interfaces and plays a key role in determining friction and wear behavior. In recent years, reactive molecular dynamics (RMD) simulations have been extensively used to explore tribochemical processes. As compared to conventional non-reactive forcefield, reactive MD is able to describe chemical bond formation and breakage. As compared to first-principles calculation, reactive MD can handle large simulation size of thousands atoms or even more. Tribochemistry is ubiquitous in the fields of liquid lubrication, solid friction, and wear, processing and manufacturing. Great efforts have been made to optimize friction and reduce wear based on tribochemical mechanisms. In the past two years, several advances and discoveries have been made in the exploration of tribochemical processes using RMD.

The use of diamond-like carbon (DLC) for engine wear reduction can be compromised by zinc-dialkyldithiophosphate (ZDDP), which are widely used in engine oils. Ruiz et al. [977] found that the tribological behavior of DLC in ZDDP-added oil can be optimized by tuning its stiffness, surface nanotopography, and hydrogen content. Contact mechanics and quantum chemical simulations show that the shear force combined with the high local contact pressure caused by the contact stiffness and the mean surface slope of the hard ta-Cs favors ZDDP fragmentation and sulfur release. In the absence of hydrogen, localized surface cold welding and sub-surface mechanical mixing of sulfur followed, resulting in lower yield stress and wear.

Based on the material characterization results of contaminated head surfaces in actual hard disk drives (HDD), Rahman et al. [978] used reactive MD simulations with ReaxFF potential to study the effect of $SiO_2$ particles as contaminants on the friction behavior and lubrication performance of PFPE lubricants (Fig. 83(a)). They found that in contrast to water molecules as contaminants (i.e., increasing the desorption rate of PFPE lubricants), $SiO_2$ particles strongly bond to the lubricant and increase the stiffness of PFPE lubricants; at the same time, the adsorbed $SiO_2$ particles increase the frictional force and lubricant transfer during sliding contact with the tip surface. This research provided theoretical guidance for design of head–disk interface during HDD operation. Pominov et al. [979] tested and revised the traditional molecular dynamics model for the organic friction modifer (OFM) used in lubricating oil, which reliably described the interaction and reactivity between OFM and steel. Furthermore, combined ultrahigh vacuum tribotests and first-principles density functional theory calculations, it was found that acetic acid adsorbed on copper surfaces can form a self-healing film during friction, which is replenishing by shear-induced decomposition of the adsorbed species [980].

MD simulation has also made many important contributions to the identification of chemical changes in friction and wear processes between solid interfaces. Gao et al. [981] found that friction can induce the structural conversion of GO to graphene perfectly on a macroscale sliding interface. They used ReaxFF MD simulations to study the friction and wear process of GO (Fig. 83(b)). Driven by shear force, the tribochemical reactions occur between the –OH group of GO and active bond of the counterpart, as well as the –OH groups of adjacent GO sheets, leading to the breakage of the C–OH bond. This leads to the transformation

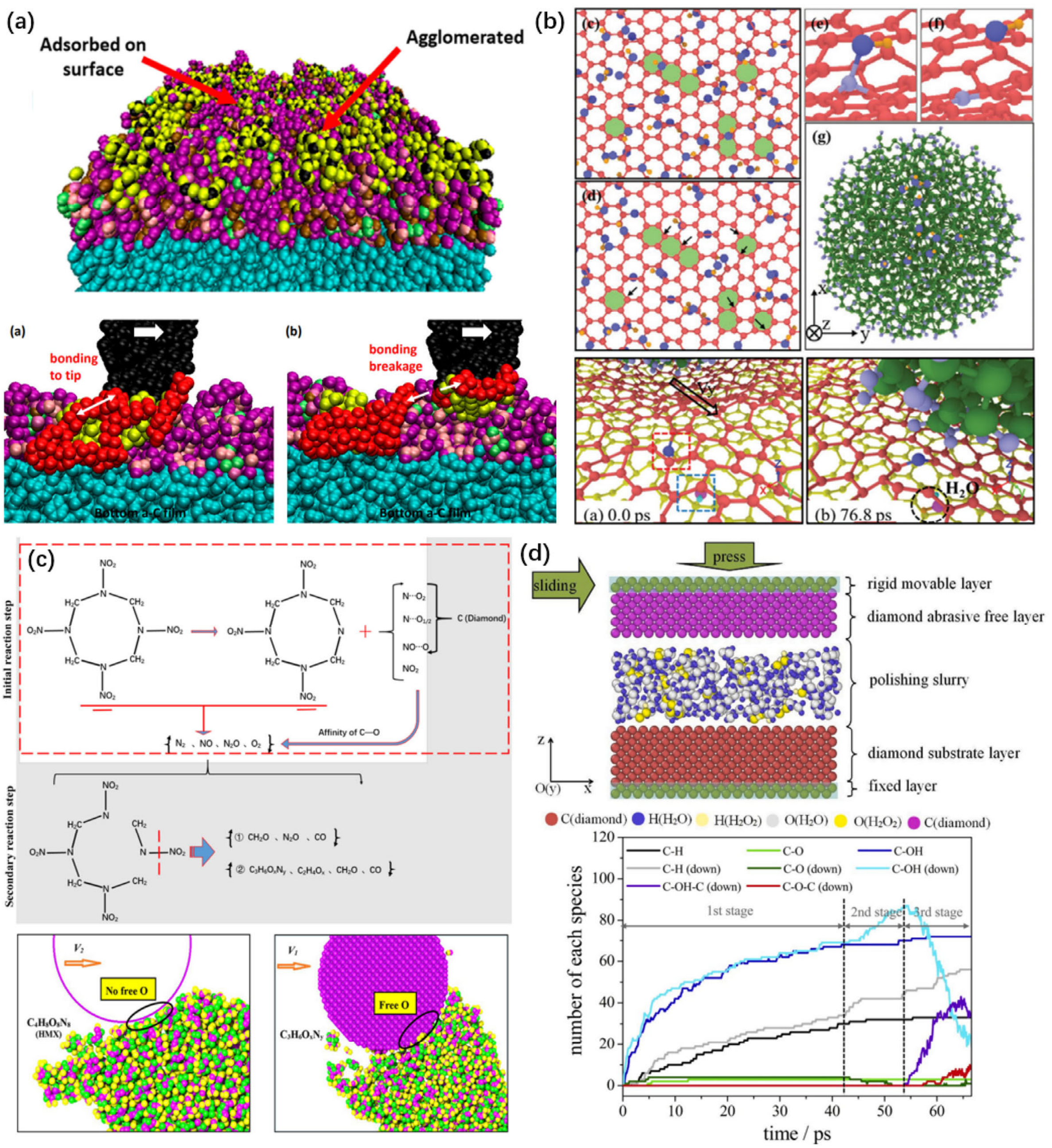

**Fig. 83** Reactive molecular dynamics simulations of tribochemistry at sliding interface [978, 981, 984, 985]. Reproduced with permission from Ref. [978], © The author(s), 2021; Reproduced with permission from Ref. [981], © Wiley-VCH GmbH, 2020; Reproduced with permission from Ref. [984], © Emerald Publishing Limited, 2021; Reproduced with permission from Ref. [985], © Elsevier Ltd., 2020.

of the sp$^3$ C to sp$^2$ C, thereby forming a perfect six-membered ring. Schall et al. [982] investigated the contact between nanoscale hydrogen-terminated monocrystalline silicon asperities and surfaces. They emphasized the important role of the concentration of terminating hydrogen atoms in the wear, adhesion, and friction behavior of nanoscale silicon surfaces, which explained the experimentally observed reversible adhesion phenomena [983].

In addition, some efforts have been done to provide theoretical support for the highly complex tribochemical process in the manufacturing. Cao et al. [984] used ReaxFF MD simulation to analyze the tribochemical behavior, reaction kinetics, decomposition products during nano-cutting of octahydro-1,3,5,7-tetranitro-1,3,5,7-tetrazocine (HMX) crystal by using diamond tool (Fig. 83(c)). The detailed tribocatalytic decomposition processes of HMX crystals had been revealed during machining with diamond, including the breakage of $–NO_2$ functional groups, activation of free oxygen atoms, etc., due to the C–O affinity between the diamond tool and HMX crystals. Yuan et al. [985] studied the friction mechanism of diamond in CMP process and found that its friction behavior is controlled by the synergistic control of the polishing fluid pressure and flow: High flow and high pressure will accelerate surface passivation and thus reduce friction, but at the same time higher pressure will also lead to more C–O–C and C–C bridges between the abrasive and the diamond matrix, resulting in increased friction and coefficient of friction (Fig. 83(d)). This work systematically revealed atomic friction details during the diamond CMP process, providing theoretical support for ultra-precise, low-damage machining of diamond. Shi et al. [986] investigated the atomic-level surface flatness and roughness removal behavior of nanoscale process in ultra-thin water environment by molecular dynamics simulation. It was found that nanoscale processing is governed by interatomic adhesion, during which the water film transmits the loading force to the copper surface, resulting in the migration and removal of surface atoms. These works deepen the understanding of nanoscale removal of materials for the development of ultra-precision manufacturing techniques.

It should be noted that recent comprehensive reviews by Martini et al. [987] and by Ta et al. [988] systematically summarize perspectives on the contribution of reactive molecular dynamics simulations as well as first principles calculations to the current understanding of tribochemistry, reveal how they help to move tribology from a "trial and error" approach to knowledge-based design of materials and lubricants.

### 6.8 New simulation methods in tribology—Peridynamics (PD)

PD is a novel continuum mechanics theory established by Silling in 2000 [989]. In PD, the continuum is subdivided into points of material occupying a volume in space. PD theory analyzes the interactions between material points in a given neighborhood. Unlike classical continuum media mechanics (CCM), the governing equation of PD is an integral–differential equation, which makes it applicable to problems involving body discontinuities such as cracks. PD can model discontinuities naturally and spontaneously without any extra technical remedies that are adopted in other conventional continuum numerical methods. Figure 84 shows the similarities and differences between CCM, PD, and molecular dynamics (MD) [990].

In Silling's initial work [989], a bond-based PD theory was proposed in which material points within the horizon exchange intrinsic information by applying forces of equal but opposite magnitude to each other.

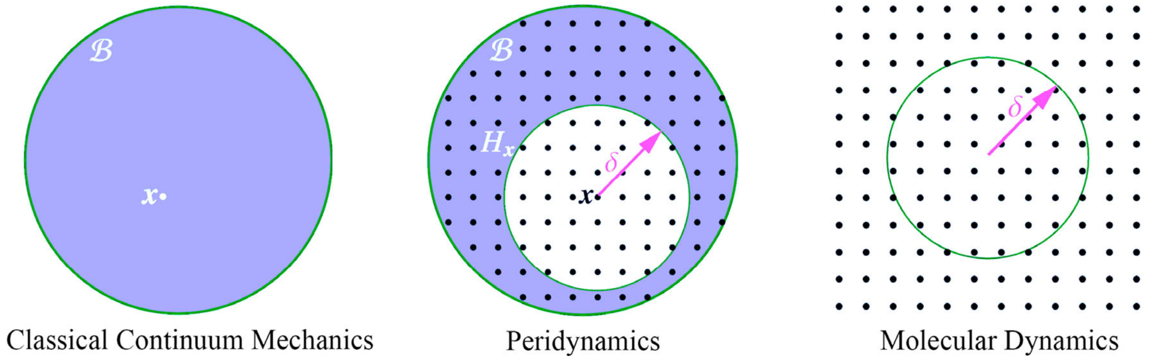

**Fig. 84** Comparison of CCM, PD, and MD. CCM and PD are formulas defined on continuum B. PD is divided into points with finite volume. In CCM, the state of a point is only affected by its immediate neighbors. In PD, a particle $x$ interacts with other particles within a neighborhood $H_x$ bounded by the horizon $\delta$. MD analyzes the interactions between discrete atoms whose interactions are governed by the cutoff radius $\delta$. PD is similar to CCM in that it is still a continuous formulation, and similar to MD in that it is nonlocal and borrows some concepts from MD, such as the cutoff radius $\delta$ [990]. Reproduced with permission from Ref. [990], © The author(s), 2019.

However, the bond-based PD model has significant limitations in simulating the mechanical behavior of materials. For example, the Poisson's ratio can only be 1/4 for isotropic materials, which cannot discern the volume expansion and shape change of solids and is not suitable for capturing plastic incompressibility conditions [991]. To overcome the above shortcomings of bond-based PD theory, in 2007, Silling et al. [992] proposed the state-based PD model. The state-based PD theory is based on the introduction of PD states, which are infinite dimensional arrays containing the bond interaction information. The state-based PD theory can be further divided into ordinary state-based theory and non-ordinary state-based theory.

PD theory has been widely used in various fields including impact [994]], tribology [995–997], cutting [998], quasi-static fracture problems [999], dynamic fracture of brittle materials [1000], etc. In fact, PD is a powerful tool to model the contact and wear process, especially involving the discontinuity problems such as cracks and material removal. For example, Cao et al. [995] used PD simulations to describe the nanoindentation process of a soda-lime silicate window glass. Kamensky et al. [1001] introduced a new state-based nonlocal friction formulation. Ebrahimi et al. [996] adopted a two-dimensional state-based PD model to study the nanoscale friction and wear behaviors of thin amorphous carbon films. Although PD has been employed to investigate the contact and wear process, related studies remain limited. In the future more work needs to be done to promote further applications of PD in tribology.

## 7 Concluding remarks and prospectives

Despite the adverse global impact of COVID-19 pandemic, substantial progress in tribology research has been accomplished during 2020–2021. The reported research has covered almost all salient aspects of tribology. It is seen that the research and development activities reported herein are mainly driven by demands for high performance and sustainable tribosystems operating under extreme operating conditions. There have been considerable advances related to novel lubricants, materials, surface modification technologies to combat friction and wear in diverse tribological situations. Increasingly more sophisticated tribological approaches were developed to tackle the complex mechanisms in medical and biological systems. These novel approaches generally consisted of integrated and coupled individual methods, such as contact, friction, lubrication and wear, experimental measurements and numerical simulations, tribology and biomechanics, etc., so that more and more realistic physiological conditions were considered. Significant progress in developing sophisticated experimental as well as computational techniques has also been reported.

## Acknowledgements

This work was financially supported by the National Natural Science Foundation of China (Nos. 52225502, 51922058, 52035012, and U2141243).

**Open Access** This article is licensed under a Creative Commons Attribution 4.0 International License, which permits use, sharing, adaptation, distribution and reproduction in any medium or format, as long as you give appropriate credit to the original author(s) and the source, provide a link to the Creative Commons licence, and indicate if changes were made.

The images or other third party material in this article are included in the article's Creative Commons licence, unless indicated otherwise in a credit line to the material. If material is not included in the article's Creative Commons licence and your intended use is not permitted by statutory regulation or exceeds the permitted use, you will need to obtain permission directly from the copyright holder.

To view a copy of this licence, visit http://creativecommons.org/licenses/ by/4.0/.
